# Analysis of sinusoidal post-buckling deformation of horizontal coiled tubing with initial residual bending

**DOI:** 10.1371/journal.pone.0301610

**Published:** 2024-05-14

**Authors:** Zhuang Li, Liangyu Chen, Fei Yuan, Lei Zhao

**Affiliations:** School of Mechanical Engineering and Automation, Northeastern University, Shenyang, Liaoning Province, China; China University of Mining and Technology, CHINA

## Abstract

In the process of horizontal well construction, the working environment in the well is very complicated. It is difficult to determine the actual deformation and force of the coiled tubing in the wellbore from the load change at the wellhead along. In addition, the coiled tubing wrapped around the reel and entering the wellbore through the injection head may cause an initial residual bending. In this paper, the mathematical model of the coiled tubing under axial load during buckling deformation is established, and the analytical solution with the time term is derived by reasonable simplification of the model. The effect of residual bending on the post-sinusoidal buckling of the coiled tubing in the horizontal well was investigated using the separation variable method. The effect of residual bending variations on the well wall contact forces is analyzed. The research shows that the initial parameter Г is the main factor influencing the post-sinusoidal buckling of the coiled tubing with residual bending. The larger the parameter value Г, the greater the effect of the residual bending on the post-sinusoidal buckling deformation of the coiled tubing, and the earlier the critical point of the mixed sinusoidal-helical buckling of the coiled tubing appears. The compression velocity of the coiled tubing has a significant effect on the well wall contact force. The faster the compression, the greater the contact force. By introducing the time term and the separation variable, this paper provides a new method and theoretical basis for further study of the process of entering sinusoidal-helical buckling of the coiled tubing with initial residual bending.

## 1. Introduction

Coiled tubing (CT) has the advantages of fast and efficient operation, low formation damage, and low cost, and is now widely used in well workovers, cleanups, sidetracks, and even drilling operations. However, due to the unique characteristics of the CT, buckling behavior usually occurs when working in complex downhole environments [1, 2], causing significant increases in downhole frictional resistance and torque, even locking, or causing failure of the tubular string connections [3–5]. Since Rubinsky pioneered study of the deformation of strings during operation and provided the differential equations for strings buckling for the first time, many scholars have also conducted research on the buckling of the tubular string [6]. Mitchell derived the differential equations for helical buckling based on the principle of virtual work, and used the Lunger-Kutta method to find the analytical solution [7]. Paslay and Dawson obtained the lateral stability conditions for inclined straight wells [8, 9]. Chen et al. developed a spiral buckling stability criterion [10], and Mitchell investigated the effect of wellbore curvature on the buckling stability of the tubular string [11]. Huang and Pattillo, Hishida, and other scholars have conducted in-depth research on helical buckling [12, 13]. Sun et al. proposed an improved helical buckling model that includes both helical and non-helical segments to reduce the error [14].

With further research, many scholars have found that the deformation of the tubular string in complex wellbores is affected by various conditions [15–17]. Mitchell, Graphics, and Miska, Gao, and Di et al. studied the deformation state of the tubular string with joints during operation [18, 19]. Liu et al. studied the sinusoidal buckling and helical buckling of the tubular string in the wellbore by using the inextensible and extensible rod theories [20], and pointed out that sinusoidal buckling deformation occurs firstly in the tubular string compression process, and then it enters the mixed sinusoidal-helical buckling deformation, and finally becomes the stabilized helical buckling deformation. However, so far there is no consistent mathematical model to represent the conversion from sinusoidal to helical buckling of the tubular string [21]. Gao studied the velocity variation during deformation of the tubular string [22]. Gao also pointed out that the velocity of movement of the tubular string has little effect on the critical load of the tubular string buckling [23, 24]. However, the important feature of the tubular string during the conversion morphology is the movement pattern of the micro segments of the tubular string.

In production, the coiled tubing is wound on a reel. The plastic deformation that occurs after a long period of time remains within the metal structure of the coiled tubing. Even if the CT is straightened, the plastic deformation remains for a long time, and it is called "residual bending". The CT is lowered into the wellbore by the rotation of the reel. Without the centralizer, the CT entering the wellbore may have an initial curvature. Qiu and Miska, and Zhu et al. have studied the buckling behavior of the CT with initial curvature, and it was shown that the initial curvature has a significant effect on the load in sinusoidal buckling and helical buckling [25, 26]. Subsequently, Qin and Gao investigated the buckling deformation of the tubular string with two initial configurations, sine and cosine, and found that the critical load values of sinusoidal buckling for the two initial configurations are basically the same [27]. Zheng and Adnan assumed the buckling deformation of the tubular string when initially helical [28]. Huang and Gao considered the effects of having both initial curvature and joints on the critical buckling load of the tubular column [29–31].

In this paper, a new method is used to intensively study the sinusoidal post-buckling deformation of the coiled tubing with residual bending in horizontal well. Firstly, a mathematical model of the bending deformation of a horizontal section of the coiled tubing, including a dynamic term, was established. The equations are reasonably simplified, and the separation variable is introduced to obtain an analytical solution for sinusoidal buckling with a time term. Secondly, when the coiled tubing with initial residual bending undergoes sinusoidal post-buckling deformation, the variation rule of the separation variable is obtained by using the energy method. The formulae for the compressive displacement and axial load of the coiled tubing containing the separation variable and time term are derived. Finally, contact force change rule analyzed for coiled tubing undergoing sinusoidal post-buckling with residual bending and motion speeds.

## 2. Theoretical model

### 2.1. Mathematical model

Before constructing the mathematical model, the following assumptions must be made to simplify the analysis process and ensure the accuracy of the results.

1. The CT cannot be compressed;
2. The mechanical effects of external factors such as downhole fluids on the CT are ignored;
3. The cross sections of the wellbore and the CT are circular;
4. The CT is always in contact with the well wall when deformation occurs;
5. The sinusoidal buckling of the CT satisfies the small deformation theory;
6. The effect of the CT rotation and torsion is ignored.
7. Both ends of the CT are connected in a hinged mode.

**Fig 1:**
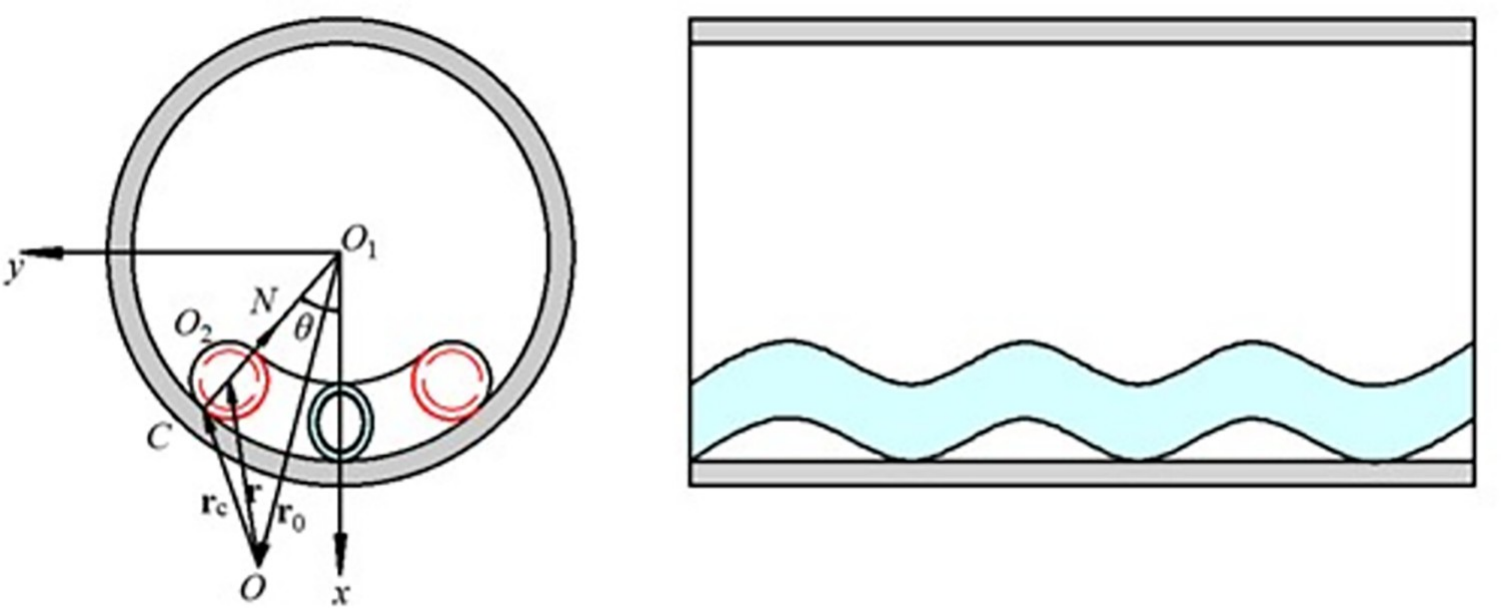
Coordinate parameters of the CT cross section.

The establishment of the coordinate system is shown in Fig 1.

where *θ* is the angular displacement of the CT when buckling deformation occurs, and *N* is the contact force between the well wall and the CT. In the deformation process of the horizontal coiled tubing, any microsegment of the CT is taken as the research object, and the motion balance equation of the CT per unit length is established as follows:

$$\frac{\partial V}{\partial s}+F=\frac{m}{\Delta s}\frac{\partial^{2}r}{\partial t^{2}}$$ (1)

According to the momentum theorem:

$$\frac{\partial \left(mv\right)}{\partial t}=\left(V+\Delta V\right)-V+F\Delta s$$ (2)

where **V** is the internal force vector of the microsegment, *m* is the mass of the microsegment, **F** is the vector of external forces per unit length of the CT, Δ*s* is the microsegment arc length, **v** is the motion velocity vector of the microsegment center point *O*_2_.

The moment of momentum theorem of the microsegment for wellbore center *O*_1_ can be expressed as:

$$\begin{array}{l} \frac{\partial \left(H\Delta s\right)}{\partial t}=M\Delta s+\left(\psi +\Delta \psi \right)-\psi +\left[\left(r+\Delta r\right)-r_{o}\right]\\ \times \left(V+\Delta V\right)-\left(r-r_{o}\right)\times V+\left(r-r_{o}\right)\times \left(F\Delta s-m\frac{\partial^{2}r}{\partial t^{2}}\right) \end{array}$$ (3)

where **r**_*o*_ is the vector diameter from the origin *O* to the center of the wellbore *O*_1_, **M** is the external torque of the external force on the CT per unit length to the center *O*_2_ of the CT, **ψ** is the internal moment on the microsegment of the CT, **r** is the vector diameter from the origin *O* to the center of the element *O*_2_, **H** is the moment of momentum per unit length of the CT concerning wellbore center *O*_1_.

From Eq (1)–(3), and ignoring small quantities, we can obtain Eq (4) as follows:

$$\begin{array}{l} \frac{\partial \left(H\Delta s\right)}{\partial t}=M\Delta s+\left(\psi +\Delta \psi \right)-\psi +\Delta r\times \left(V+\Delta V\right)+\left(r-r_{o}\right)\times \left(\Delta V-\frac{\partial V}{\partial s}\Delta s\right)\\ =M\Delta s+\left(\psi +\Delta \psi \right)-\psi +\Delta r\times V+\left(r-r_{o}\right)\times \left(\Delta V-\frac{\partial V}{\partial s}\Delta s\right) \end{array}$$ (4)

Assuming that **e** is a tangential unit vector, the two ends of the equal sign in Eq (4) are divided by Δ*s* and taken as infinitesimal, and then:

$$\frac{\partial H}{\partial t}=M+\frac{\partial \psi }{\partial s}+e\times V$$ (5)

The angular momentum of the microsegment moving along the well wall is given by Eq (6).

$$H=\rho A\left(r-r_{o}\right)\times \left[\Omega \times \left(r-r_{o}\right)+v_{z}k\right]+I_{c}\omega$$ (6)

where *I*_*c*_ is the moment of inertia per unit length of the CT around its own axis, **ω** is the angular velocity vector of the rotation of the CT on its axis, **Ω** is the angular velocity vector of the centroid of the unit length of the tubular string rotating in the XY-plane about the center of the wellbore. The vector parameters are represented by the subscripts X, Y, and Z, corresponding to components along the X-axis, Y-axis, and Z-axis, respectively.

When the CT is buckled and deformed, Gao and Mitchell believe that the movement direction of the contact point between the CT and the well wall can be divided into two directions: the movement direction along the Z-axis and the well wall rotation along the XY-plane [16, 33]. It is assumed that the dynamic friction coefficient along the Z-axis is *μ*_*z*_ and the dynamic friction coefficient in the XY-plane is *μ*_*xy*_. The expressions of the friction vector **f** and the external force vector **F** per unit length of the CT string in continuous contact with the well wall are as follows respectively:

$$f=sign\left(\theta \right)\left(\mu_{xy}N\sin\theta \cdot i-\mu_{xy}N\cos\theta \cdot j\right)-\mu_{z}Nk$$ (7)

$$\begin{array}{l} F=\left[-N\cos\theta +sign\left(\theta \right)\mu_{xy}N\sin\theta +\rho Ag\right]i\\ +\left[-N\sin\theta -sign\left(\theta \right)\mu_{xy}N\cos\theta \right]j-\mu_{z}Nk \end{array}$$ (8)

where sign (*θ*) = 1 when the CT microsegment rotates clockwise, and sign(*θ*) = -1 when the CT microsegment rotates counterclockwise.

Assuming **M** is the external torque of the external force on the unit-length tubular string to the center *O*_2_ of the CT, and **r**_*o*2*c*_ is the vector from the contact point *C* to the center *O*_2_ of the tubular string microsegment, as shown in Fig 1. From Eq (5), (6) and (9), it can be obtained:

$$M=r_{o2c}\times \left[sign\left(\theta \right)\mu_{xy}N\sin\theta \cdot i-sign\left(\theta \right)\mu_{xy}N\cos\theta \cdot j-\mu_{z}Nk\right]$$ (9)

If the radius is *r*, and *r*_*p*_ is the CT radius, and *r*_*s*_ is the wellbore radius, then: *r = r*_*s*_*—r*_*p*_. The following conditions are satisfied.


$$\left\{ \begin{array}{l} x=r\cos\theta \\ y=r\sin\theta \end{array}\right.$$ (10)

Substitute Eq (8) into (1), and satisfy *m = ρA*Δ*s*, we can get Eq (11) after expansion.


$$\left\{ \begin{array}{l} \frac{\partial V_{x}}{\partial s}=\rho A\frac{\partial^{2}x}{\partial t^{2}}+\left(N\cos\theta -sign\left(\theta \right)\mu_{xy}N\sin\theta -\rho Ag\right)\\ \frac{\partial V_{y}}{\partial s}=\rho A\frac{\partial^{2}y}{\partial t^{2}}+\left(N\sin\theta +sign\left(\theta \right)\mu_{xy}N\cos\theta \right)\\ \frac{\partial V_{z}}{\partial s}=\rho A\frac{\partial^{2}z}{\partial t^{2}}+\mu_{z}N \end{array}\right.$$ (11)

The symbol ()’ is the derivative of the arc length *s*, and the component of the external force vector on the Z-axis is **F**. Eq (1)–(6) are substituted into the constitutive equation of Gao for derivation [21], and ignoring torque, we can then substitute Eq (7)–(11) and use the Lagrange formula to expand the derivation and obtain:

$$F^{\prime }x^{\prime }+Fx^{\prime \prime }+EI\left(\begin{array}{l} 3\theta^{\prime \prime \prime }{\theta^{\prime }}^{3}r^{3}\cos\theta +9{\theta^{\prime }}^{2}{\theta^{\prime \prime }}^{2}r^{3}\cos\theta -3{\theta^{\prime }}^{4}\theta^{\prime \prime }r^{3}\sin\theta -2rz^{\prime }z^{\prime \prime }{\theta^{\prime }}^{3}\sin\theta \\ +6rz^{\prime }z^{\prime \prime }\theta^{\prime }\theta^{\prime \prime }\cos\theta +2rz^{\prime }z^{\prime \prime }\theta^{\prime \prime \prime }\sin\theta -rz^{\prime \prime }z^{\prime \prime \prime }\theta^{\prime }\sin\theta -r{z^{\prime }}^{2}{\theta^{\prime }}^{4}\cos\theta \\ +3r{z^{\prime }}^{2}{\theta^{\prime \prime }}^{2}\cos\theta +4r{z^{\prime }}^{2}\theta^{\prime }\theta^{\prime \prime \prime }\cos\theta -6r{z^{\prime }}^{2}{\theta^{\prime }}^{2}\theta^{\prime \prime }\sin\theta +r{z^{\prime }}^{2}\theta^{\prime \prime \prime \prime }\sin\theta \\ -rz^{\prime }z^{\prime \prime \prime }{\theta^{\prime }}^{2}\cos\theta -rz^{\prime }z^{\prime \prime \prime }\theta^{\prime \prime }\sin\theta -rz^{\prime }z^{\prime \prime \prime \prime }\theta^{\prime }\sin\theta \end{array}\right)=\chi_{1}$$ (12)

$$F^{\prime }y^{\prime }+Fy^{\prime \prime }+EI\left(\begin{array}{l} 3\theta^{\prime \prime \prime }{\theta^{\prime }}^{3}r^{3}\sin\theta +9{\theta^{\prime }}^{2}{\theta^{\prime \prime }}^{2}r^{3}\sin\theta +3{\theta^{\prime }}^{4}\theta^{\prime \prime }r^{3}\cos\theta +2rz^{\prime }z^{\prime \prime }{\theta^{\prime }}^{3}\cos\theta \\ +6rz^{\prime }z^{\prime \prime }\theta^{\prime }\theta^{\prime \prime }\sin\theta -2rz^{\prime }z^{\prime \prime }\theta^{\prime \prime \prime }\cos\theta +rz^{\prime \prime }z^{\prime \prime \prime }\theta^{\prime }\cos\theta -r{z^{\prime }}^{2}{\theta^{\prime }}^{4}\sin\theta \\ +3r{z^{\prime }}^{2}{\theta^{\prime \prime }}^{2}\sin\theta +4r{z^{\prime }}^{2}\theta^{\prime }\theta^{\prime \prime \prime }\sin\theta +6r{z^{\prime }}^{2}{\theta^{\prime }}^{2}\theta^{\prime \prime }\cos\theta -r{z^{\prime }}^{2}\theta^{\prime \prime \prime \prime }\cos\theta \\ -rz^{\prime }z^{\prime \prime \prime }{\theta^{\prime }}^{2}\sin\theta +rz^{\prime }z^{\prime \prime \prime }\theta^{\prime \prime }\cos\theta +rz^{\prime }z^{\prime \prime \prime \prime }\theta^{\prime }\cos\theta \end{array}\right)=\chi_{2}$$ (13)

where:

$$\zeta_{1}=\left(y^{\prime }M_{z}-z^{\prime }M_{y}\right)-y^{\prime }\rho A\left[x\left(\frac{\partial^{2}\theta }{\partial t^{2}}y+\frac{\partial \theta }{\partial t}\frac{\partial y}{\partial t}\right)-y\left(\frac{\partial^{2}\theta }{\partial t^{2}}x+\frac{\partial \theta }{\partial t}\frac{\partial x}{\partial t}\right)\right]-z^{\prime }\rho A\left(x\frac{\partial^{2}z}{\partial t^{2}}+\frac{\partial z}{\partial t}\frac{\partial x}{\partial t}\right),$$

$$\zeta_{2}=\left(z^{\prime }M_{x}-x^{\prime }M_{z}\right)-z^{\prime }\rho A\left(y\frac{\partial^{2}z}{\partial t^{2}}+\frac{\partial z}{\partial t}\frac{\partial y}{\partial t}\right)+x^{\prime }\rho A\left[x\left(\frac{\partial^{2}\theta }{\partial t^{2}}y+\frac{\partial \theta }{\partial t}\frac{\partial y}{\partial t}\right)-y\left(\frac{\partial^{2}\theta }{\partial t^{2}}x+\frac{\partial \theta }{\partial t}\frac{\partial x}{\partial t}\right)\right],$$

$$\chi_{1}=\rho A\frac{\partial^{2}x}{\partial t^{2}}+\left(N\cos\theta -sign\left(\theta \right)\mu_{xy}N\sin\theta -\rho Ag\right)-{\zeta_{1}}^{\prime },$$

$$\chi_{2}=\rho A\frac{\partial^{2}y}{\partial t^{2}}+\left(N\sin\theta +sign\left(\theta \right)\mu_{xy}N\cos\theta \right)-{\zeta_{2}}^{\prime }.$$

where *E* is the elastic modulus, and *I* is the moment of inertia. According to the hypothesis of Li [34], *ds*≈*dz*. From Eqs (12) and (13), we can get the fourth-order nonlinear partial differential equations including the dynamic effect when the coiled tubing in the horizontal well is compressed and bent by axial load:

$$\left\{ \begin{array}{l} EIr\left[6{\left(\frac{\partial \theta }{\partial z}\right)}^{2}\frac{\partial^{2}\theta }{\partial z^{2}}-\frac{\partial^{4}\theta }{\partial z^{4}}\right]+r\frac{\partial }{\partial z}\left(F\frac{\partial \theta }{\partial z}\right)-sign\left(\theta \right)\mu_{xy}N+\mu_{z}r_{p}N\frac{\partial \theta }{\partial z}-\rho Ag\sin\theta \\ =\rho Ar\left[\frac{\partial^{2}z}{\partial t^{2}}\frac{\partial \theta }{\partial z}+\frac{\partial }{\partial z}\left(\frac{\partial z}{\partial t}\frac{\partial \theta }{\partial t}\right)+\frac{\partial^{2}\theta }{\partial t^{2}}\right]\\ EIr\left[4\frac{\partial \theta }{\partial z}\frac{\partial^{3}\theta }{\partial z^{3}}-{\left(\frac{\partial \theta }{\partial z}\right)}^{4}+3{\left(\frac{\partial^{2}\theta }{\partial z^{2}}\right)}^{2}\right]-rF{\left(\frac{\partial \theta }{\partial z}\right)}^{2}-N+\mu_{z}r_{p}\frac{\partial N}{\partial z}+\rho Ag\cos\theta \\ =\rho Ar\left[\frac{\partial }{\partial z}\left(\frac{\partial^{2}z}{\partial t^{2}}\right)-\frac{\partial \theta }{\partial t}\left(\frac{\partial z}{\partial t}\frac{\partial \theta }{\partial z}+\frac{\partial \theta }{\partial t}\right)\right] \end{array}\right.$$ (14)

Eq (14) are the same as the conclusion of Li [35]. If the CT is stationary, equations become the form of inclination angle *β* = 0, according to Mitchell’s conclusion. Because the CT is in continuous contact with the well wall, the contact force changes little with the axial coordinate z, and the friction coefficient *μ*_*z*_ is less than 1. Additionally, because the contact force on the well wall at the beginning of the buckling deformation of the CT is approximately equal to the gravity of the CT, the values of *μ*_*z*_*Nr*_*p*_*θ*′ and *μ*_*z*_*r*_*p*_*N*′ in (14) are very small. It shows that the influence of axial friction on the tubular string deformation is far less than that of radial friction at the beginning of the tubular string deformation, which is the same as that of Gao and Miska [23, 33].

### 2.2. Dimensionless

The following variables are defined:

$$\kappa_{1}={\left(\frac{EIr}{q}\right)}^{\frac{1}{4}},\;\kappa_{2}=2{\left(\frac{EIq}{r}\right)}^{\frac{1}{2}},\;\kappa_{3}={\left(\frac{I}{r^{3}g}\right)}^{\frac{1}{2}}.$$

The dimensionless parameters are:

$$\bar{z}=\frac{z}{\kappa_{1}},\;\bar{F}=\frac{F}{\kappa_{2}},\;\bar{t}=\frac{t}{\kappa_{3}},\;\bar{N}=\frac{N}{q}.$$

where *q* is gravity per unit length of the CT. Because the initial deformation is small, for the convenience of solving, ignoring the influence of friction and high-order small quantity [22], Eqs (15) is a non-dimensional partial differential equation set for the deformation of horizontal coiled tubing, taking into account dynamic effects.


$$\left\{ \begin{array}{l} \frac{\partial^{4}\theta }{\partial {\bar{z}}^{4}}-2\bar{F}\frac{\partial^{2}\theta }{\partial {\bar{z}}^{2}}+\theta =-2\frac{r^{4}}{I}\frac{\partial^{2}\theta }{\partial {\bar{t}}^{2}}\\ 4\frac{\partial \theta }{\partial \bar{z}}\frac{\partial^{3}\theta }{\partial {\bar{z}}^{3}}+3{\left(\frac{\partial^{2}\theta }{\partial {\bar{z}}^{2}}\right)}^{2}-2\bar{F}{\left(\frac{\partial \theta }{\partial \bar{z}}\right)}^{2}-\bar{N}+1-\frac{\theta^{2}}{2}+\frac{\theta^{4}}{24}=-2\frac{r^{4}}{I}{\left(\frac{\partial \theta }{\partial \bar{t}}\right)}^{2} \end{array}\right.$$ (15)

## 3. Results and discussions

### 3.1. Energy analysis

The two ends of the horizontal coiled tubing are connected as hinged, then the boundary conditions are:

$${\frac{\partial^{2}\theta }{\partial {\bar{z}}^{2}}|}_{\bar{z}=0}={\frac{\partial^{2}\theta }{\partial {\bar{z}}^{2}}|}_{\bar{z}=\bar{L}}=0,\;\theta \left(0,\bar{t}\right)=\theta \left(\bar{L},\bar{t}\right)=0.$$

In the boundary condition of the equation, the axial dimensionless length of the horizontal string after deformation is $\bar{L}$. Assuming that the starting end of the coiled tubing is hinged and the tail is moving, an analytical solution to the Eq (15) is obtained by substituting boundary conditions and introducing a separation variable, as shown in Eq (16).

$$\theta \left(\bar{z},\bar{t}\right)=\left(C_{1}e^{\bar{t}\sqrt{-\frac{\lambda }{\beta }}}+C_{2}e^{-\bar{t}\sqrt{-\frac{\lambda }{\beta }}}\right)\sin\left(\frac{\pi n}{\bar{L}}\bar{z}\right)$$ (16)

where *λ* is the separation variable, *C*_1_ and *C*_2_ are arbitrary constants, and *β* = 2*r*4 / *I*. The necessary and sufficient conditions to satisfy the above equation are

$$\bar{F}=-\frac{1}{2}\left[\frac{n^{2}\pi^{2}}{{\bar{L}}^{2}}+\frac{{\bar{L}}^{2}}{n^{2}\pi^{2}}\left(1-\lambda \right)\right]$$ (17)

When *λ* = 1, Eq (17) becomes the Euler critical load hinged at both ends. When the CT is in the static state or uniform motion state, it satisfies: ∂^2^*θ* / ∂ ${\bar{t}}^{2}$ = 0. The definition of the separation variable, *λ* = 0, satisfies this requirement. The expression for axial load excludes the velocity term, which is the same as the conclusion of Gao [22]. According to the conclusion of Haberman [37], when solving the equation with a small angle, that is, sin (*θ*) ≈ *θ*, the separation variable must satisfy *λ* < 0 when the CT is sinusoidal buckling. Eq (16) can be simplified as:

$$\theta \left(\bar{z},\bar{t}\right)=A_{n}\sin\left(\frac{n\pi }{\bar{L}}\bar{z}\right)$$ (18)

where: $A_{n}=C_{1}e^{\bar{t}\sqrt{-\frac{\lambda }{\beta }}}+C_{2}e^{-\bar{t}\sqrt{-\frac{\lambda }{\beta }}}$.

For the CT with initial residual bending can be expressed by Eq (19).

$$\theta_{0}=A_{0}\sin\left(\frac{m\pi }{L_{0}}z\right)$$ (19)

where *A*_0_ is the initial deflection of the CT, and *m* is the initial half-wave number, and *L*_0_ is the initial axial length of the CT. The conditions are satisfied at this time.


$$\left\{ \begin{array}{l} x_{0}=r\cos\theta_{0}\\ y_{0}=r\sin\theta_{0} \end{array}\right.$$

The *x*_0_ and *y*_0_ are the horizontal and vertical coordinates values corresponding to the initial state of the CT, respectively, and *θ*_0_ is the initial angular displacement of the cross-section.

The energy of the CT with buckling deformation are as follows [10, 38, 39].

$$\Delta U=\frac{EI}{2}\int_{0}^{L}\left[{\left(\frac{d^{2}x}{dz^{2}}-\frac{d^{2}x_{0}}{dz^{2}}\right)}^{2}+{\left(\frac{d^{2}y}{dz^{2}}-\frac{d^{2}y_{0}}{dz^{2}}\right)}^{2}\right]dz$$ (20)

$$\Delta W_{q}=\int_{0}^{L}q\left(r_{c}\cos\theta_{0}-r_{c}\cos\theta \right)dz$$ (21)

$$\Delta W_{F}=-F\left\{\int_{0}^{L}\frac{1}{2}\left[{\left(\frac{dx}{dz}\right)}^{2}+{\left(\frac{dy}{dz}\right)}^{2}\right]dz-\int_{0}^{L_{0}}\frac{1}{2}\left[{\left(\frac{dx_{0}}{dz}\right)}^{2}+{\left(\frac{dy_{0}}{dz}\right)}^{2}\right]dz\right\}$$ (22)

where Δ*U* is the bending strain energy, Δ*W*_*q*_ is the gravitational potential energy, Δ*W*_*F*_ is the axial load work. Because the integral term appears to transcend functions sin(*A*sin *θ*) and cos(*A*sin *θ*). To simplify the calculation, the integral term is expanded using a power series as shown in Eq (23).


$$\left\{ \begin{array}{l} \cos\left(A\sin\theta \right)=1-\frac{A^{2}\sin^{2}\theta }{2}\\ \sin\left(A\sin\theta \right)=A\sin\theta -\frac{A^{3}\sin^{3}\theta }{6} \end{array}\right.$$ (23)

Using the potential energy principle, we get:

$$\Pi =\Delta U+\Delta W_{q}-\Delta W_{F}$$

By substituting Eq (20)–(23) respectively, and dimensionless, we can get

$$\Pi =\frac{1}{2}qrL\left[\begin{array}{l} \frac{3}{8}{A_{0}}^{4}\frac{\pi^{4}m^{4}}{{{\bar{L}}_{0}}^{4}}+\frac{1}{2}{A_{n}}^{2}\frac{\pi^{4}n^{4}}{{\bar{L}}^{4}}+\frac{1}{2}{A_{0}}^{2}\frac{\pi^{4}m^{4}}{{{\bar{L}}_{0}}^{4}}+\frac{3}{8}{A_{n}}^{4}\frac{\pi^{4}n^{4}}{{\bar{L}}^{4}}-\frac{1}{16}{A_{0}}^{4}{A_{n}}^{2}\frac{m^{2}\pi^{2}}{{{\bar{L}}_{0}}^{2}}\frac{\pi^{2}n^{2}}{{\bar{L}}^{2}}\\ -\frac{1}{16}{A_{0}}^{2}{A_{n}}^{4}\frac{m^{2}\pi^{2}}{{{\bar{L}}_{0}}^{2}}\frac{\pi^{2}n^{2}}{{\bar{L}}^{2}}+\frac{1}{2}\left({A_{n}}^{2}-{A_{0}}^{2}\right)+\bar{F}\left(\frac{{A_{n}}^{2}\pi^{2}n^{2}}{{\bar{L}}^{2}}-\frac{{A_{0}}^{2}\pi^{2}m^{2}}{\bar{L}{\bar{L}}_{0}}\right) \end{array}\right]$$ (24)

According to the principle of the minimum potential energy of a system, there is

$$\delta \Pi =\frac{\partial \Pi }{\partial A_{n}}\delta A_{n}=0$$

Due to the arbitrariness of *δA*_*n*_, the relationship between the dimensionless axial load and the amplitude *A*_*n*_ of the horizontal coiled tubing is obtained after ∂Π / ∂*A*_*n*_ = 0 is satisfied Eq (25).


$$\bar{F}=\frac{1}{16}{A_{0}}^{4}\frac{m^{2}\pi^{2}}{{{\bar{L}}_{0}}^{2}}+\frac{1}{8}{A_{0}}^{2}{A_{n}}^{2}\frac{m^{2}\pi^{2}}{{{\bar{L}}_{0}}^{2}}-\frac{1}{2}\frac{\pi^{2}n^{2}}{{\bar{L}}^{2}}-\frac{3}{4}{A_{n}}^{2}\frac{\pi^{2}n^{2}}{{\bar{L}}^{2}}-\frac{{\bar{L}}^{2}}{2\pi^{2}n^{2}}$$ (25)

Taking the derivative of $\bar{L}$ at both ends of the Eq (25) and setting it equal to zero, we can get

$$\frac{\pi^{2}n^{2}}{{\bar{L}}^{2}}={\left(\frac{2}{3{A_{n}}^{2}+2}\right)}^{\frac{1}{2}}$$ (26)

By substituting Eq (26) into (25), the expression of minimum dimensionless axial load required for buckling deformation of a horizontal coiled tubing with initial deflection *A*_0_ and initial half-wave number *m* can be obtained.


$$\bar{F}=\frac{1}{16}{A_{0}}^{4}\frac{m^{2}\pi^{2}}{{{\bar{L}}_{0}}^{2}}+\frac{1}{8}{A_{0}}^{2}{A_{n}}^{2}\frac{m^{2}\pi^{2}}{{{\bar{L}}_{0}}^{2}}-{\left(\frac{3{A_{n}}^{2}+2}{2}\right)}^{\frac{1}{2}}$$ (27)

If the initial mode of the CT is a straight-line shape, then there is *A*_0_ = 0. Eq (27) becomes:

$$\bar{F}=-{\left(\frac{3{A_{n}}^{2}+2}{2}\right)}^{\frac{1}{2}}$$

At this point, the CT just deformed can be considered *A*_*n*_ = 0, the axial load becomes:

$$F=\kappa_{2}\bar{F}=-2{\left(\frac{EIq}{r}\right)}^{\frac{1}{2}}$$

This result is the same as the conclusion of Palsay, and the conclusion in Miska when *α* = 90° [3, 32].

The dimensionless expression for the compression displacement of a horizontal coiled tubing is as follows:

$${\bar{L}}_{z}=\frac{r^{2}}{4{\kappa_{1}}^{2}}\left({A_{n}}^{2}\frac{\pi^{2}n^{2}}{\bar{L}}-{A_{0}}^{2}\frac{\pi^{2}m^{2}}{{\bar{L}}_{0}}\right)$$ (28)

Substituting Eq (26) into (28) yields an expression for the amplitude *A*_*n*_ and the dimensionless compression displacement ${\bar{L}}_{z}$ as shown in Eq (29).

$${A_{n}}^{2}=\frac{3{\left(\Psi {\bar{L}}_{z}+\Gamma \right)}^{2}+\left(\Psi {\bar{L}}_{z}+\Gamma \right)\sqrt{9{\left(\Psi {\bar{L}}_{z}+\Gamma \right)}^{2}+16{\bar{L}}^{2}}}{4{\bar{L}}^{2}}$$ (29)

where: $\Psi =\frac{4{k_{1}}^{2}}{{r_{c}}^{2}}>0$, $\Gamma ={A_{0}}^{2}\frac{\pi^{2}m^{2}}{{\bar{L}}_{0}}>0$.

### 3.2. Numerical analysis

**Fig 2:**
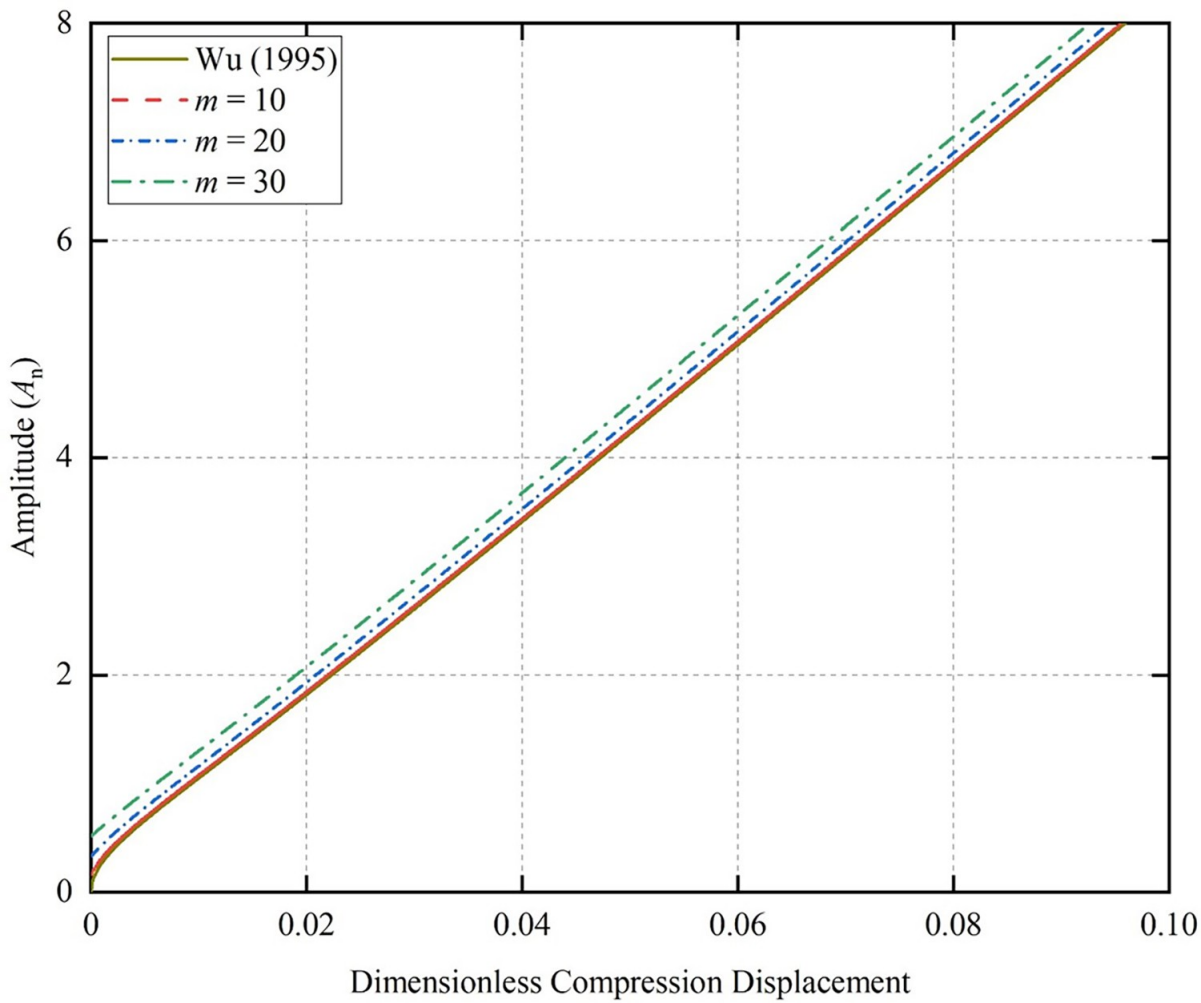
Relationship between compression displacement and amplitude.

Assuming the outsider diameter of the coiled tubing is 2 inches, the wall thickness of the CT is 0.165 inches, the radius of the wellbore is 3.5 inches, the density of the coiled tubing is 489 lb/ft^3^, and the modulus of elasticity is *E* = 2.988×10^7^ PSI. The relationship between the compression displacement and the generated amplitude at different initial amplitudes can be obtained from Eq (29), as shown in Fig 2 (*A*_0_ = 0.5, ${\bar{L}}_{0}$ = 100).

The change of the initial half-wave number has little effect on the change of amplitude *A*_*n*_, which is basically consistent with the results of Wu [40]. The slopes of the amplitudes are all the same, and the increase of *A*_*n*_ under the same displacement of compression is the same. From Eq (29) we can see that the initial variable Г is the main factor influencing the amplitude *A*_*n*_. Changing the initial half-wave number and changing the initial amplitude both change the Г value. If the change of Г value is small, the effect on the change of amplitude *A*_*n*_ is small.

**Fig 3:**
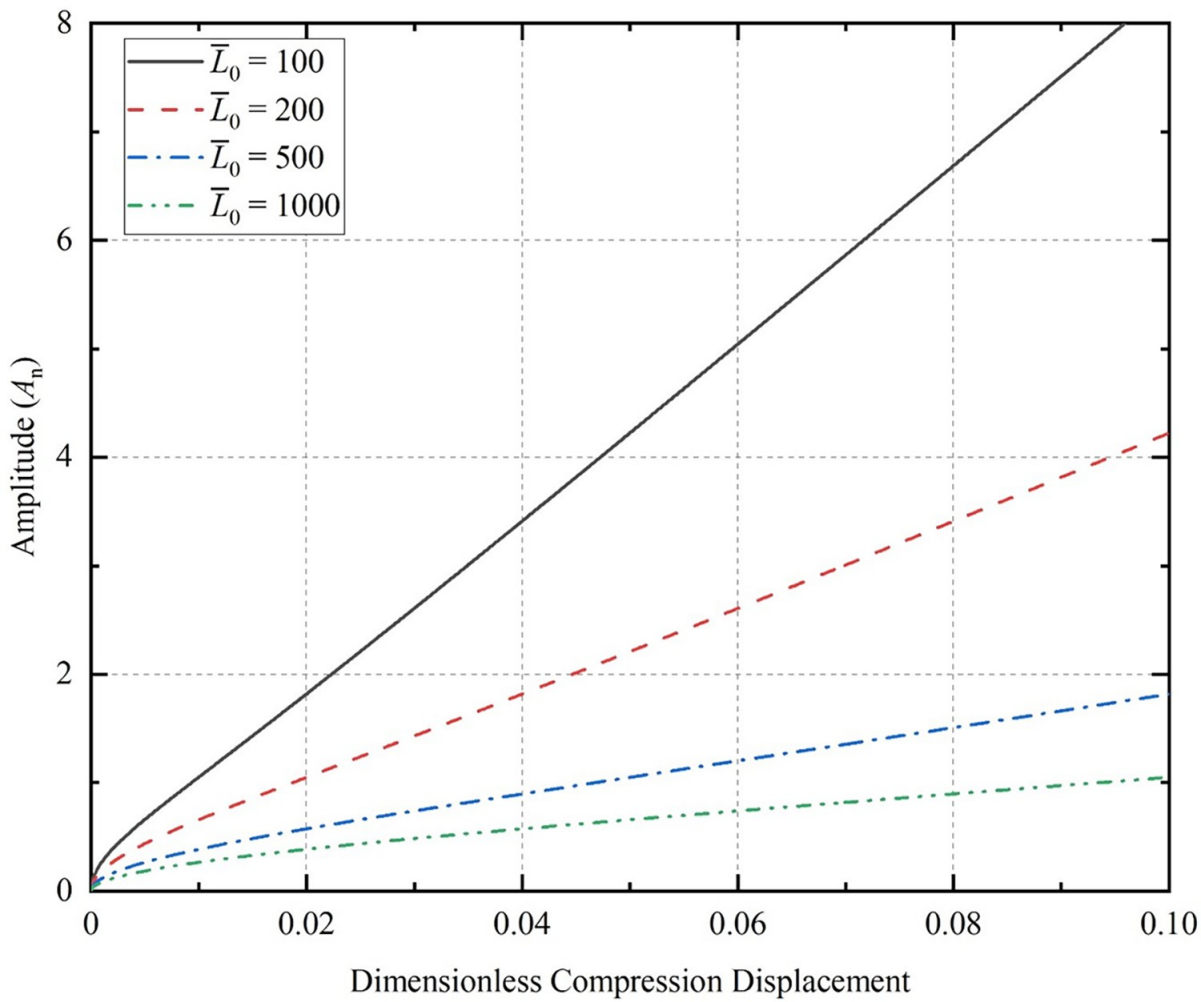
Relationship between compression displacement and amplitude.

Fig 3 (*A*_0_ = 0.5, *m* = 2) shows the amplitude change curves of columns with different initial lengths during compression. It can be seen that the longer the initial length of the CT, the smaller the amplitude under compression for the same displacement. This is because the longer CT generates more half-waves in compression [35], causing the amplitude to decrease.

**Fig 4:**
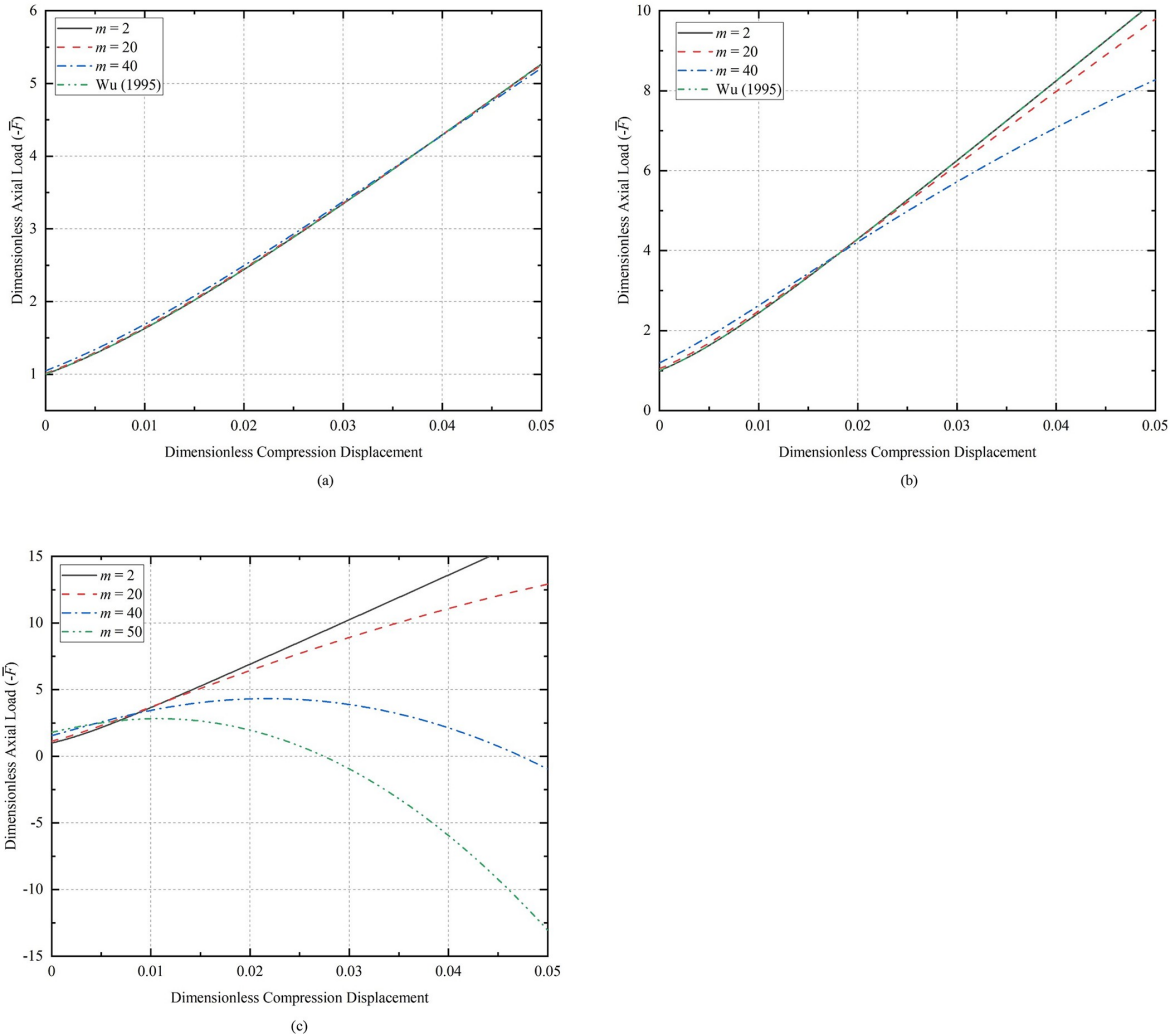
Relationship between compression displacement and axial load for the CT; (a) dimensionless length 100 of the CT; (b) dimensionless length 50 of the CT; (c) dimensionless length 30 of the CT.

By substituting Eq (29) into (25), we can obtain the dimensionless axial load $\bar{F}$ versus compression displacement ${\bar{L}}_{z}$ required for bending of horizontal coiled tubing with different initial half-wave numbers, as shown in Fig 4(A) (${\bar{L}}_{0}$ = 100). The axial load necessary for the CT to experience buckling deformation increases with the number of half-waves, however, the increase is relatively minor. It is evident that the axial load is less affected by the change in the initial half-wave number for smaller Г values. If we continue to increase the value of Г and shorten the dimensionless length of the CT to 50, the relationship between the dimensionless axial load and the compression displacement is shown in Fig 4(B).

The initial half-wave number increases, the less axial load is required to compress the same displacement. However, the critical load for sinusoidal buckling deformation of the CT with initial deflection exceeds the critical load value for sinusoidal buckling of the initially straight CT. The relationship between axial load and compression displacement at varying initial amplitudes as the Г value increase is depicted in Fig 4(C) (${\bar{L}}_{0}$ = 30). The extreme value of axial load occurs at around 0.0221 of dimensionless compression displacement for the CT with the initial half-wave number *m* = 40. For the CT with *m* = 50, the dimensionless compression displacement is approximately 0.11 when the axial load is extreme. This means that the larger the initial half-wave number is, the earlier the axial load extreme occurs.

Actually, Eq (27) takes the derivative of the amplitude *A*_*n*_ at both ends and sets it equal to zero, we can get:

$${A_{n}}^{2}\left(\frac{1}{8}{A_{0}}^{4}\frac{m^{4}\pi^{4}}{{{\bar{L}}_{0}}^{4}}-\frac{9}{3{A_{n}}^{2}+2}\right)=0$$

Since the CT has residual bending, the solution for the amplitude is obtained as follows:

$${A_{n}}^{2}=\frac{24}{{A_{0}}^{4}}\frac{{{\bar{L}}_{0}}^{4}}{m^{4}\pi^{4}}-\frac{2}{3}$$

It can be seen that the axial load $\bar{F}$ will have an extreme point when Г is taken at the appropriate value as shown in Eq (30).


$$\bar{F}=\frac{{A_{0}}^{2}\left(3{A_{0}}^{2}-4\right)}{48}\frac{\pi^{2}m^{2}}{{{\bar{L}}_{0}}^{2}}-\frac{3{{\bar{L}}_{0}}^{2}}{{A_{0}}^{2}\pi^{2}m^{2}}$$ (30)

Li shows that the critical point where sinusoidal buckling transforms into sinusoidal-helical buckling is the extreme point of Eq (30) [35]. As the value of Г increases, the axial load will appear earlier at the critical point, and the sinusoidal-helical shape will appear earlier as well. As compression continues, the angular displacement *θ* increases, and causing the error resulting from Eq (14) with approximate values to becomes larger. The curve behind the extreme point of the axial load in Fig 4(C) has a large error and is not practically relevant.

**Fig 5:**
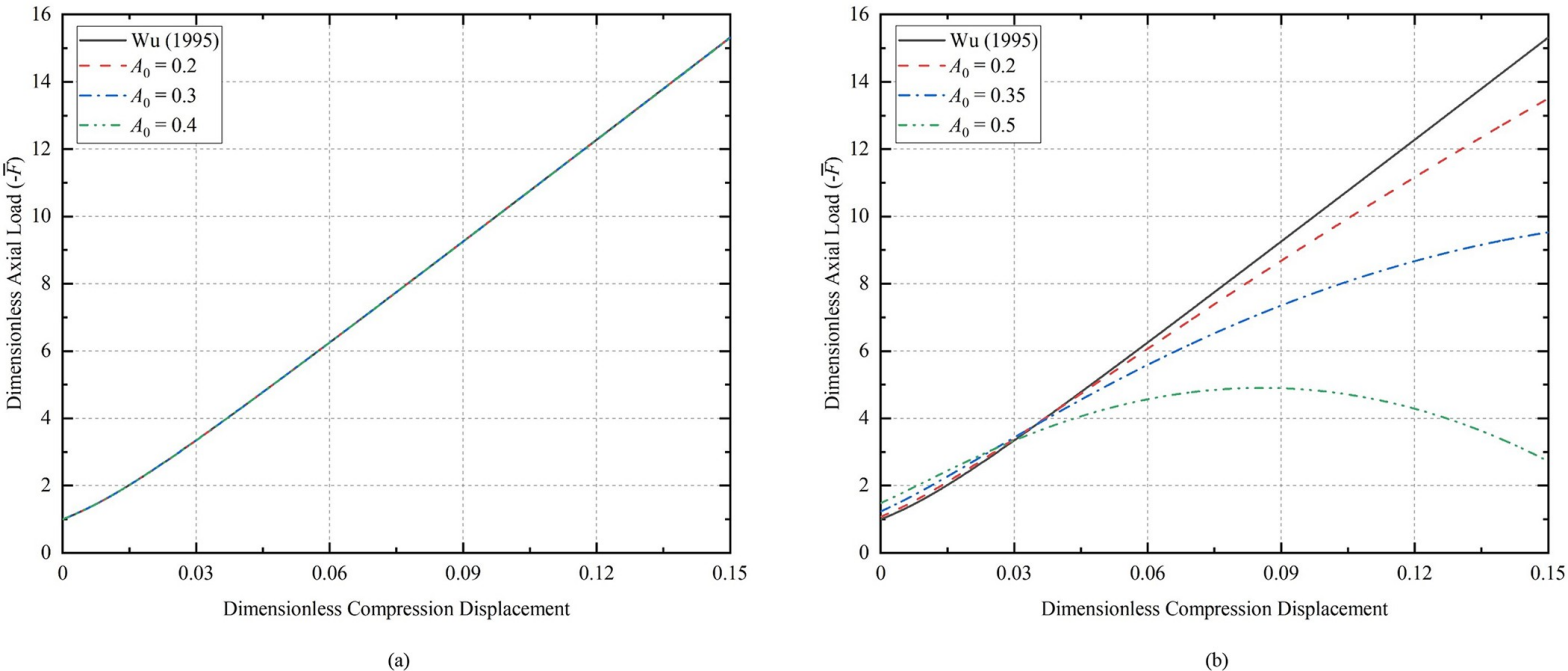
Relationship between compression displacement and axial load of the CT; (a) the initial half-wave number of 2; (b) the initial half-wave number of 50.

Fig 5(A) illustrates the relationship between axial load and compression displacement for sinusoidal buckling of the CT with various initial amplitudes, where the initial half-wave number *m* = 2 and dimensionless length ${\bar{L}}_{0}$ = 100.

**Fig 6:**
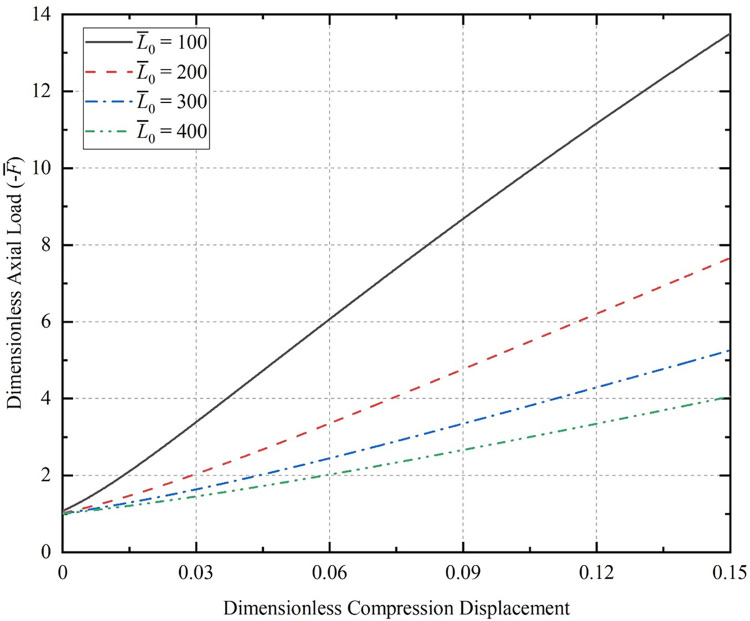
Relation between axial load and compression displacement.

Fig 6 shows the same initial conditions (*m* = 2, *A*_0_ = 0.2), the relationship between axial load and compression displacement of different initial lengths of the CT during compression.

The larger the length of the CT with initial deflection, the smaller the axial load is required to compress the same displacement when buckling. This conclusion is the same as the rule of sinusoidal buckling deformation of horizontal coiled tubing with straight initial shape considered by Li [35].

### 3.3. Influence of separation variable

**Fig 7:**
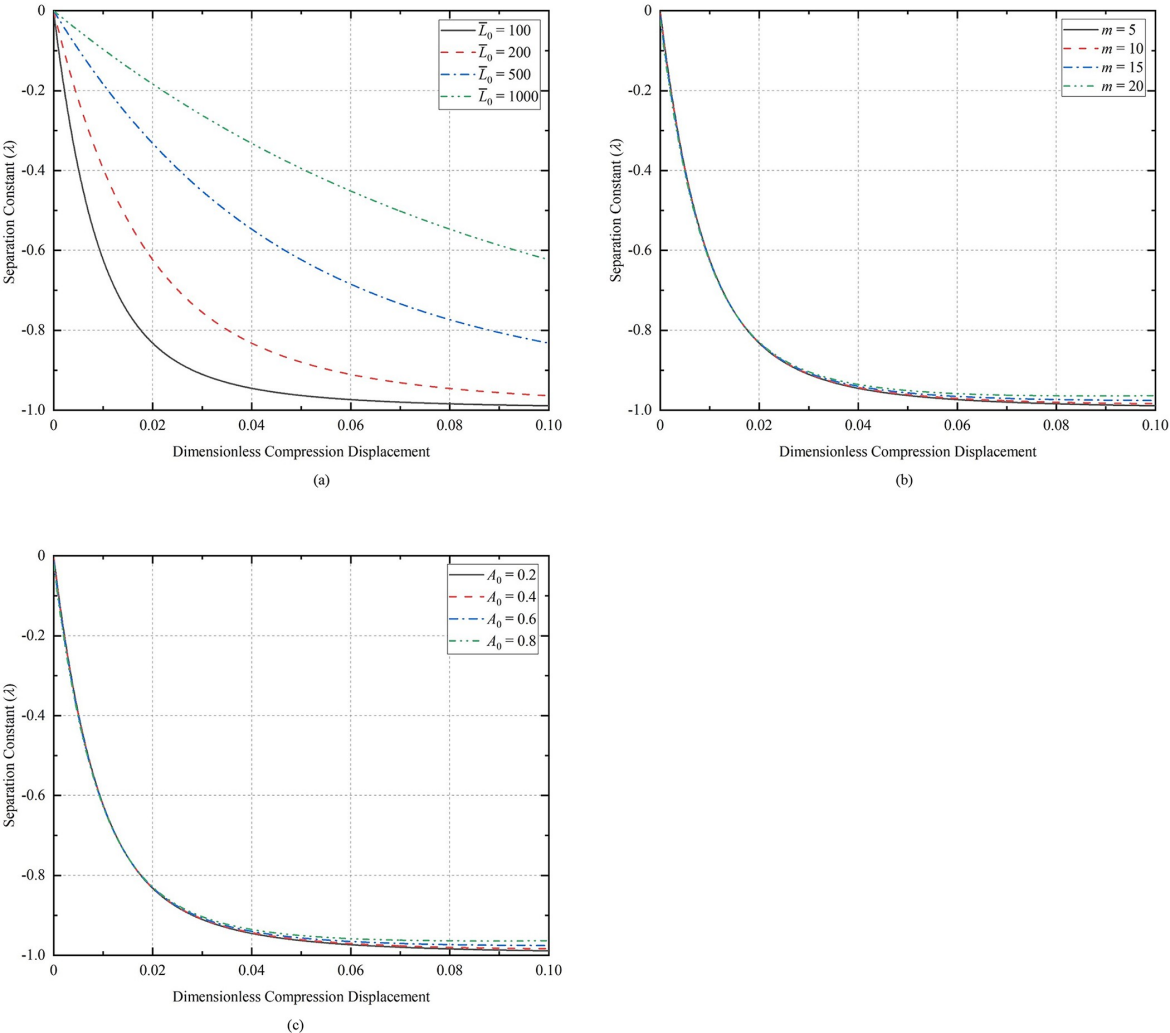
The relationship between the separation variable and the amplitude: (a) *m* = 5, *A*_0_ = 0.2; (b) ${\bar{L}}_{0}$ = 100, *A*_0_ = 0.2; (c) ${\bar{L}}_{0}$ = 100, *m* = 5.

Substituting Eqs (25), (26) and (29) into Eq (17) gives the relationship between the separation variable and the amplitude. Fig 7(A)-7(C) show the relationship between separation variable and amplitudes at different lengths, different initial half-wave numbers, and different initial deflections, respectively. The value range of the separation variable is *λ* > -1. The initial length change has a large effect on *λ*, but the initial half-wave number and the initial amplitude have a small effect on *λ*.

**Fig 8:**
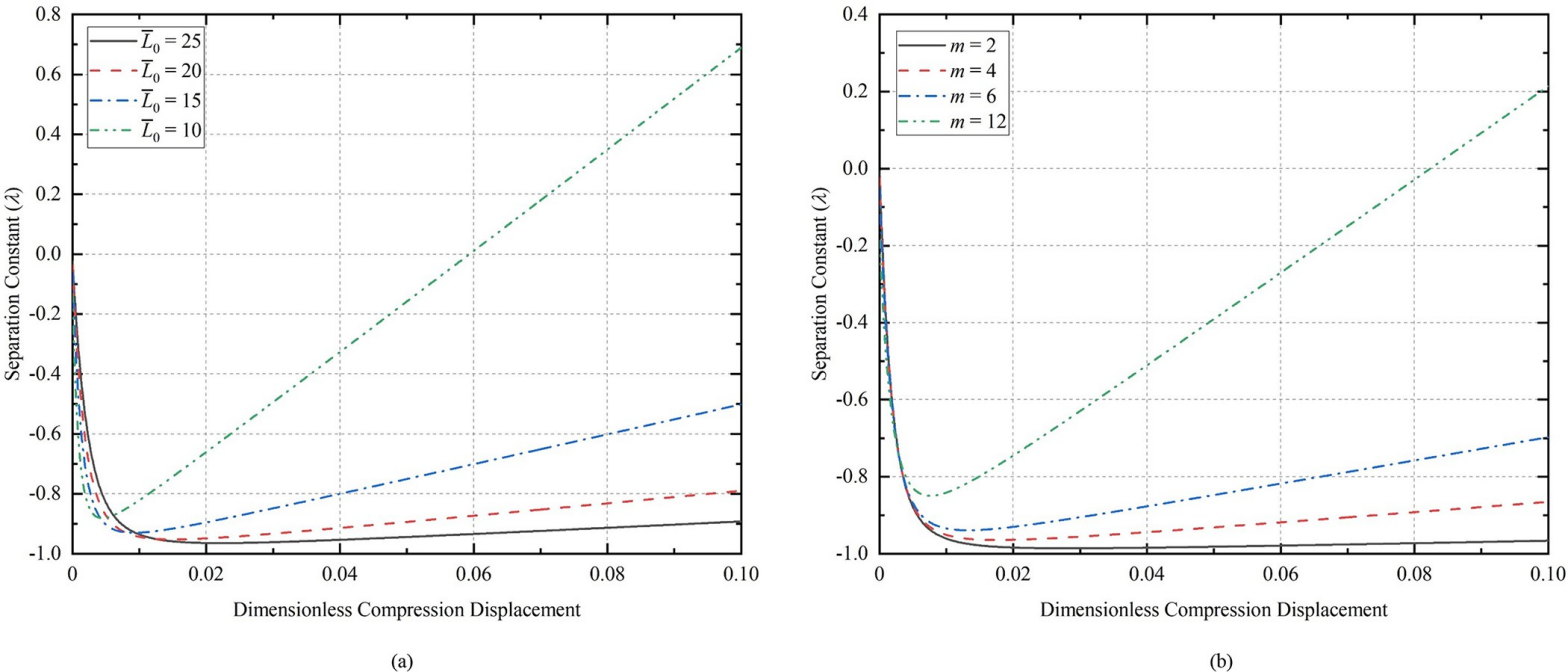
Relationship between separation variable and compression displacement: (a) *m* = 5, *A*_0_ = 0.2; (b) ${\bar{L}}_{0}$ = 25, *A*_0_ = 0.2.

**Fig 9:**
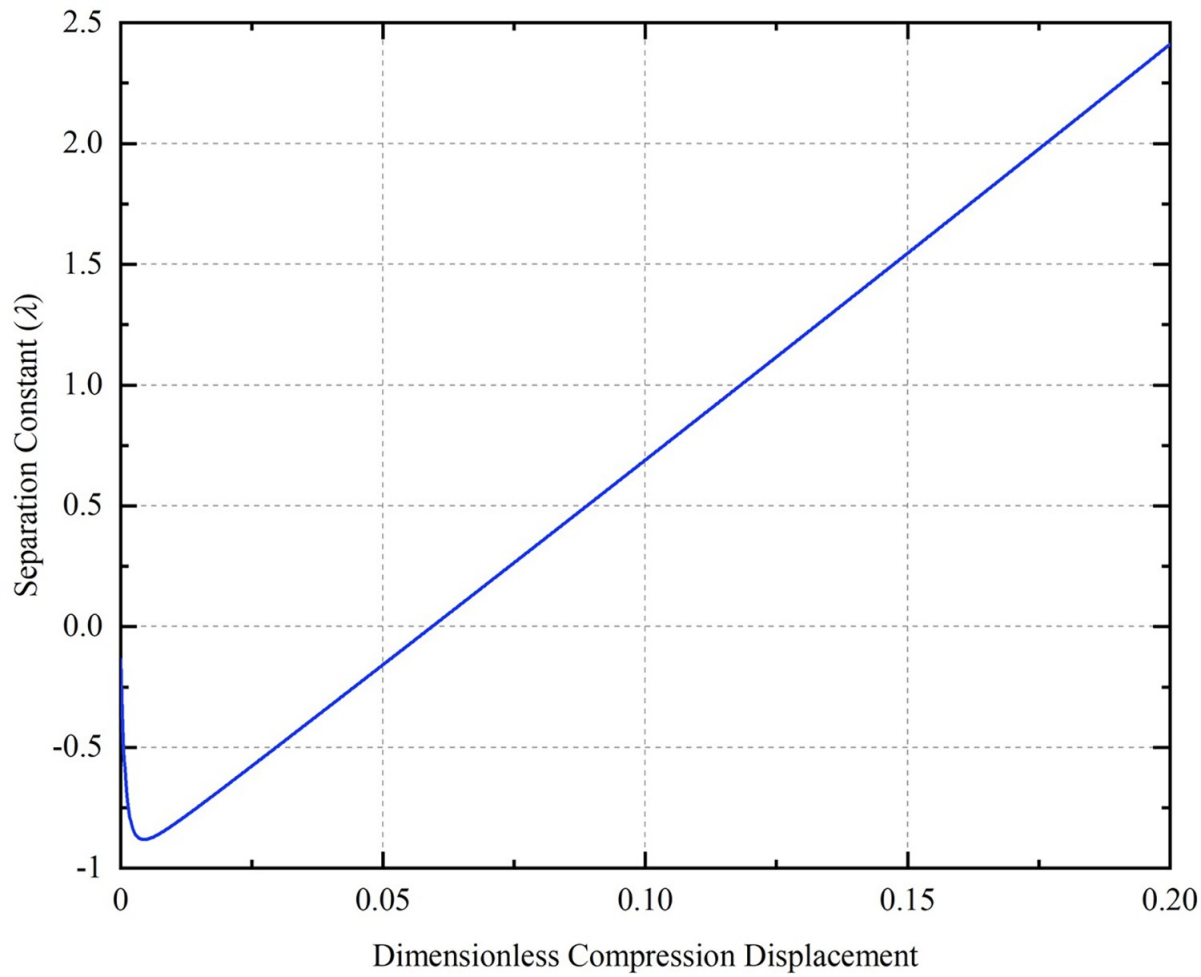
Relationship between separation variable and compression displacement.

Fig 8(A) and 8(B) show the relationship curves of the separation variable with the compression displacement for the conditions of *m* = 5, *A*_0_ = 0.2 and ${\bar{L}}_{0}$ = 25, *A*_0_ = 0.2, respectively. The range of values of *λ* appears to be greater than zero after compression to certain displacements for the CT with ${\bar{L}}_{0}$ = 10 in Fig 8(A), and for the CT with *m* = 12 in Fig 8(B). Under the conditions of *m* = 5, *A*_0_ = 0.2, ${\bar{L}}_{0}$ = 10, the curve of compression displacement versus separation constant during compression of dimensionless displacement to 0.2 is shown in Fig 9. According to the analytical results of Timoshenko [36], the deformation of the CT occurs due to the presence of the basic reaction force, so the separation variable is satisfied: *λ* ≤ 1. If *λ* > 1, the contact force of the well wall on the buckling coiled tubing is negative, and at this time, the CT is separated from the contact with the well wall, and even more severe plastic deformation occurs, and it is obvious that the calculation using the formula is no longer meaningful. The larger the value of the initial parameter Г, the earlier the extremes of *λ* will occur.

### 3.4. Numerical calculation of contact force

The expression for the dimensionless contact force $\bar{N}$ on the well wall when sinusoidal buckling occurs in coiled tubing with residual bending can be obtawined from the coiled tubing deformation Eq (16) and the coiled tubing initial configuration Eq (19) by substituting them into Eq (15) as shown in Eq (31). The symbol definitions in the expressions are consistent with the settings in the paper.


$$\begin{array}{l} \bar{N}=3{A_{n}}^{2}\frac{\pi^{4}n^{4}}{{\bar{L}}^{4}}\sin^{2}\left(\frac{\pi n}{\bar{L}}\bar{z}\right)-4{A_{n}}^{2}\frac{\pi^{4}n^{4}}{{\bar{L}}^{4}}\cos^{2}\left(\frac{\pi n}{\bar{L}}\bar{z}\right)-2\bar{F}{A_{n}}^{2}\frac{\pi^{2}n^{2}}{{\bar{L}}^{2}}\cos^{2}\left(\frac{\pi n}{\bar{L}}\bar{z}\right)+1\\ -\frac{{A_{n}}^{2}\sin^{2}\left(\frac{\pi n}{\bar{L}}\bar{z}\right)}{2}+\frac{{A_{n}}^{4}\sin^{4}\left(\frac{\pi n}{\bar{L}}\bar{z}\right)}{24}\\ -\lambda {\left[\frac{\left(A_{n}-A_{0}e^{-\bar{t}\sqrt{-\frac{\lambda }{Q}}}\right)\left(e^{\bar{t}\sqrt{-\frac{\lambda }{Q}}}+e^{-\bar{t}\sqrt{-\frac{\lambda }{Q}}}\right)}{e^{\bar{t}\sqrt{-\frac{\lambda }{Q}}}-e^{-\bar{t}\sqrt{-\frac{\lambda }{Q}}}}-A_{0}e^{-\bar{t}\sqrt{-\frac{\lambda }{Q}}}\right]}^{2}\sin^{2}\left(\frac{\pi n}{\bar{L}}\bar{z}\right) \end{array}$$ (31)

**Fig 10:**
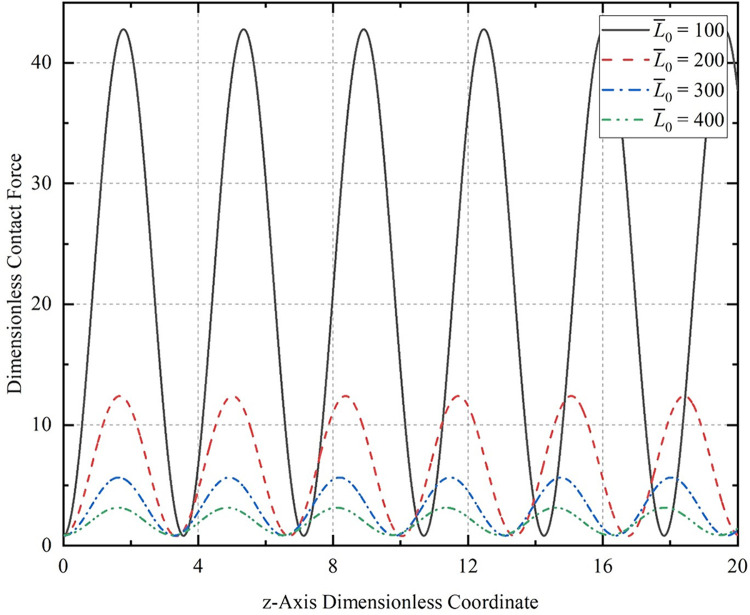
Contact force curves of the CT with different lengths on the horizontal well wall.

The relationship between the time term $\bar{t}$ and the compression displacement ${\bar{L}}_{z}$ affects the compression speed of the CT. Assuming that the end of the CT moves with a dimensionless velocity of uniform ${\bar{v}}_{0}$, we have: ${\bar{v}}_{0}$ = ${\bar{L}}_{z}$ / $\bar{t}$. The contact force curves of different lengths of the CT on the horizontal well wall when the dimensionless compression displacement ${\bar{L}}_{z}$ = 0.005, the dimensionless time $\bar{t}$ = 1, the initial amplitude *A*_0_ = 0.2, and the initial half-wave number *m* = 2 are taken as shown in Fig 10, and in Fig 10 for convenience of presentation, the horizontal coordinate is taken only for the length of the tube column: $\bar{z}$= 20.

It can be seen that the contact force of the CT on the well wall changes periodically with the change in length and is greater than zero. This result is the same as the conclusion of Gao [21]. By comparing the contact force curve of different lengths of the tubular string on the well wall, it can be seen that other parameters being equal, the larger the length of the CT with residual bending, the larger the maximum contact force on the wellbore under the same displacement, while the minimum contact force is almost not affected by the length of the CT.

**Fig 11:**
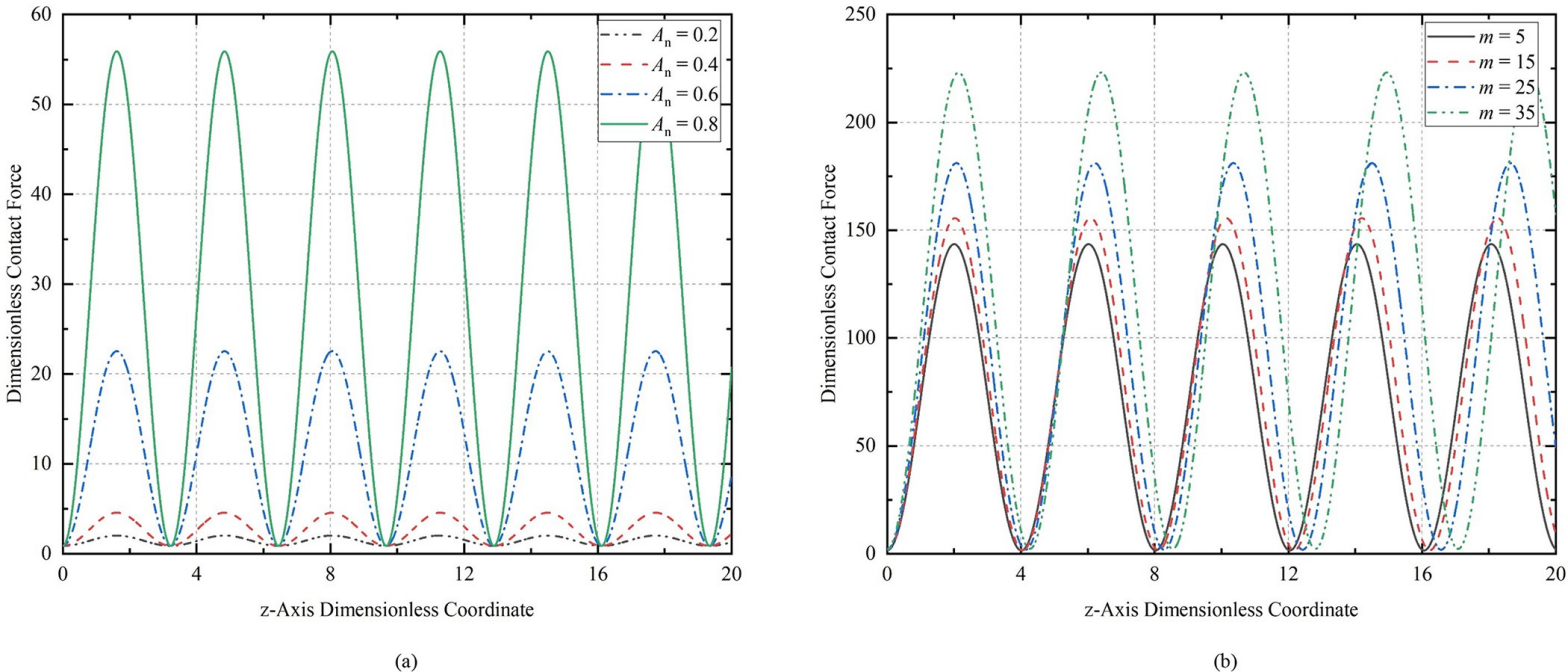
Contact force curves for the CT: (a) different initial amplitudes; (b) different initial half-wave numbers.

Fig 11(A) and 11(B) show the relationship curves of the contact force of the CT on the well wall when compressed with the same displacement under different initial amplitudes *A*_0_ (${\bar{L}}_{0}$ = 50, *m* = 5, ${\bar{L}}_{z}$ = 0.005, $\bar{t}$ = 1) and different initial half-wave numbers *m* (${\bar{L}}_{0}$ = 50, *A*_0_ = 2, ${\bar{L}}_{z}$ = 0.005, $\bar{t}$ = 1) at the same initial length, respectively.

The maximum value of the contact force on the well wall of a buckling tubular column increases as the initial amplitude increases, and the increase is greater. When *A*_0_ = 0.4, $\bar{N}$ = 4.57, when *A*_0_ = 0.6, $\bar{N}$ = 22.54, when *A*_0_ = 0.8, $\bar{N}$ = 4.57. When *A*_0_ = 0.8, the maximum contact force is reached ${\bar{N}}_{\max}$ = 55.9. Therefore, the maximum contact force on the well wall when the CT with residual bending is buckled is greater than that when the CT with a straight initial shape is bent. The minimum contact forces are: ${\bar{N}}_{\min}$ = 0.8852, ${\bar{N}}_{\min}$ = 0.8851, ${\bar{N}}_{\min}$ = 0.8846, and ${\bar{N}}_{\min}$ = 0.8842, respectively. The minimum contact force of the buckling coiled tubing to the well wall is not affected by the change in initial amplitude. As the number of initial half-wave increases, the maximum value of the contact force of the buckled CT on the well wall increases, while the minimum value is essentially unaffected. However, a larger number of initial half-waves has less effect on the magnitude of the maximum contact force change than a larger initial amplitude has on the magnitude of the maximum contact force change.

**Fig 12:**
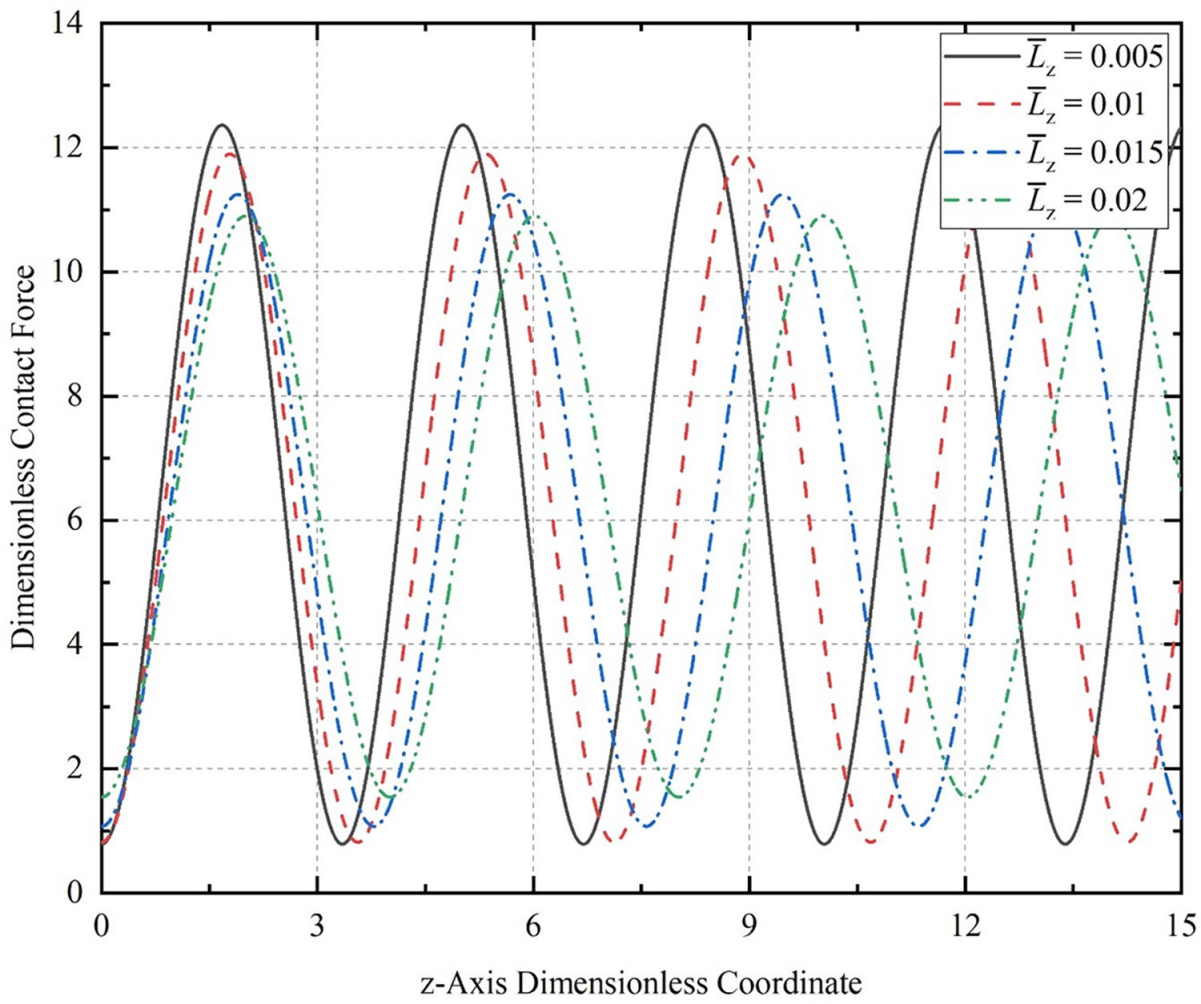
Contact force curves for the CT with different compression displacements.

Initial amplitude *A*_*n*_ = 0.2, initial half-wave number *m* = 2, dimensionless length ${\bar{L}}_{0}$ = 200 of the CT, in the dimensionless speed of 0.005 conditions, respectively, compression of the dimensionless displacement: 0.005, 0.01, 0.015, 0.02, when the contact force generated by the change rule of the cycle as shown in Fig 12.

The greater the compression displacement, the smaller the value of the maximum contact force of the CT on the well wall and the larger the value of the minimum contact force. The compression displacement becomes larger, the axial load required necessarily increases, and the contact force on the CT located at the bottom of the wellbore increases. However, the CT tends to return to its initial form at this point, causing the contact force of the sinusoidal peak to gradually decrease. The contact force period decreases over time, indicating a reduction in the number of sinusoidal half-waves generated by the buckled coiled tubing as compression progresses. Eventually, the CT transforms into a mixed sinusoidal-helical buckling deformation pattern.

**Fig 13:**
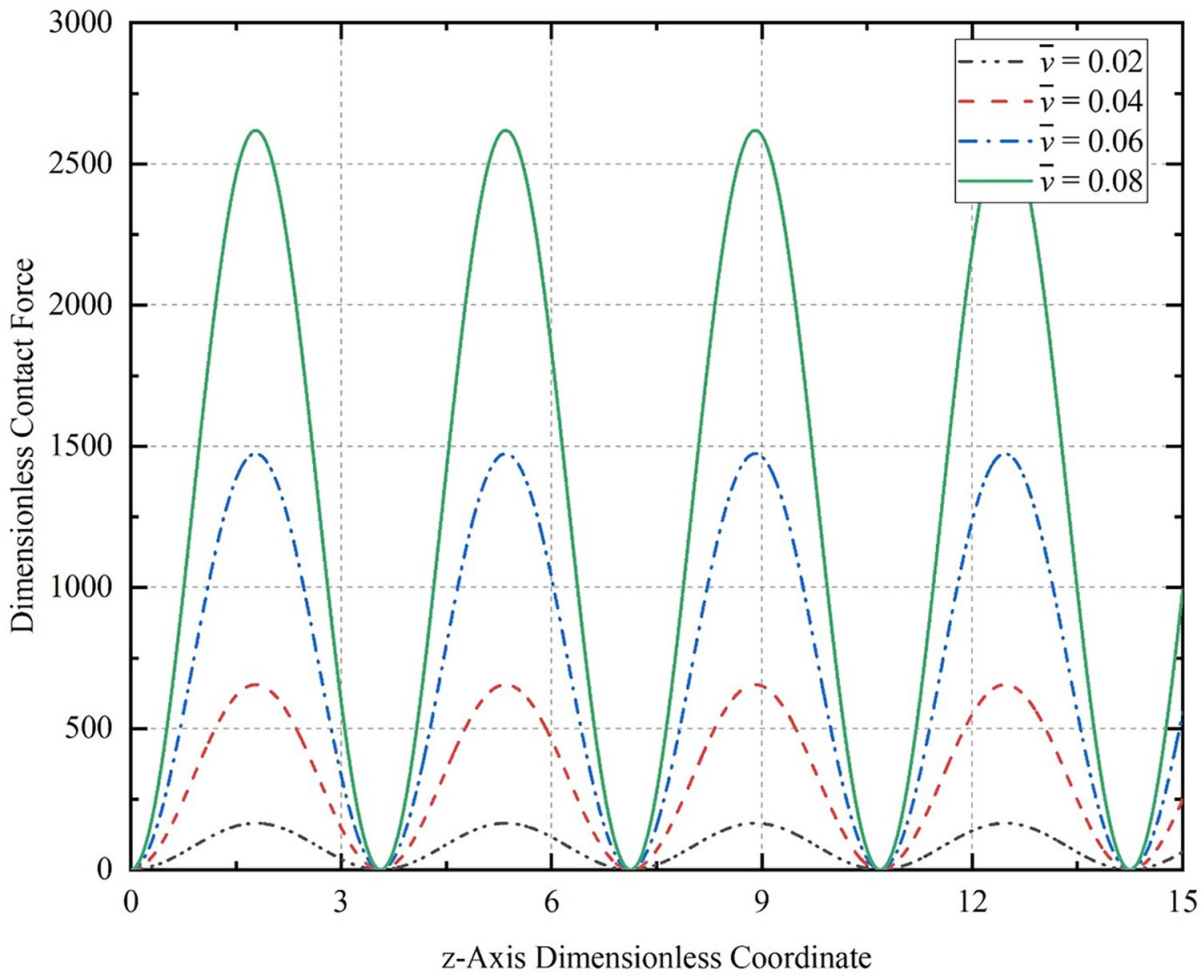
Contact force curves for the CT at different speeds.

Fig 13 shows the variation of the contact force at a compression of 0.01 dimensionless displacement for the CT with dimensionless length ${\bar{L}}_{0}$ = 200, initial amplitude *A*_0_ = 0.2, and initial half-wave number *m* = 2. The dimensionless velocities $\bar{v}$ at the end is taken as 0.02, 0.04, 0.06, and 0.08, respectively.

Fig 13 shows that the greater the coiled tubing lowering speed, the greater the maximum contact force generated by the deformation of the coiled tubing on the well wall. The minimum value remains largely unchanged and is estimated as $\bar{N}$ = 0.8171. The maximum value of dimensionless contact force is $\bar{N}$ = 165.3 when $\bar{v}$ = 0.02. However, when the dimensionless velocity $\bar{v}$ = 0.08, the maximum value of dimensionless contact force increases to $\bar{N}$ = 2619. If the velocity is further increased to $\bar{v}$ = 0.1, the maximum value of dimensionless contact force reaches $\bar{N}$ = 4092. Therefore, if the speed at which the CT is lowered exceeds a certain value during actual production, the contact force of the buckling coiled tubing on the well wall increases rapidly. This is one of the reasons why the coiled tubing lowering speed cannot be too fast.

## 4. Conclusions

This paper presents a generalized mathematical model for the buckling deformation of a horizontal coiled tubing during its motion. The model is established using the momentum theorem and the momentum moment theorem. The mathematical model includes the time term for the sinusoidal buckling deformation of a coiled tubing with residual bending. The equations are simplified and a separation variable is introduced. The residual bending of the coiled tubing is considered a sinusoidal shape. The amplitude, axial load, and contact force variation of the coiled tubing after sinusoidal buckling are analyzed using the energy method. This lays the foundation for further research on the process of the horizontal coiled tubing with initial deflection transforming from sinusoidal buckling to helical buckling, and the following conclusions are obtained.

1. Theoretical analysis was conducted on the emergence conditions of sinusoidal buckling of horizontal coiled tubing with initial deflection to mixed sinusoidal-helical buckling critical point. The results indicate that as the initial parameter Γ of the coiled tubing increases, the transition from sinusoidal buckling to sinusoidal-helical buckling critical point occurs earlier during compression. If the initial parameter Γ is very small, the sinusoidal-helical buckling deformation of the coiled tubing is not easy to occur.
2. The effects of compression displacement on the amplitude, axial load, and separation variable of the coiled tubing with different initial amplitudes and initial half-wave numbers are analyzed. The deformation press is similar to that calculated by Wu when the Γ value is small, and the initial half-wave number and initial amplitude have little influence on the deformation of the coiled tubing. As the value of Γ increase, the critical load of sinusoidal buckling of the coiled tubing also increases with the initial amplitude. However, the change of the initial half-wave number has little effect on the critical load of sinusoidal buckling.
3. A mathematical model was developed to calculate the contact force of a horizontal coiled tubing with residual bending subjected to sinusoidal post-buckling, which includes a time term. The contact force changes on the well wall during sinusoidal post-buckling of the coiled tubing under different conditions were calculated. According to the calculation results, when the coiled tubing with residual bending undergoes buckling deformation, the maximum contact force on the well wall is greater than that calculated by Gao. The longer the coiled tubing, the greater the maximum contact force on the well wall at the same distance of compression. The more the initial half-wave number, the greater the maximum contact force. The larger the initial amplitude, the greater the maximum value of contact force. However, the minimum value of contact force under the above conditions is not affected. Finally, it was proven theoretically that the lowering speed of the coiled tubing has a significant impact on the contact force. and the faster the lowering speed, the greater the contact force.

Mathematically, the set of differential equations for the buckling deformation of the coiled tubing is a system of high-order strongly nonlinear coupled system of partial differential equations, so the process of solving the equations uses a more idealized case, ignoring some boundary conditions such as friction, torque, and some small values. Although the compression distance at which the CT undergoes sinusoidal buckling is small, its deformation increases when the CT continues to be compressed, which may result in large errors if the small angle approximation in the paper continues to be used for analytical calculations. Therefore, the analysis process in this paper is limited to the process of sinusoidal buckling deformation of the CT.

The future research will focus on the deformation state of the CT when the spiral buckling occurs using the mathematical model established in this paper, the effect of increasing the friction, torque, and other boundary conditions on the mathematical model, and how the coiled tubing is converted from sinusoidal buckling to spiral buckling in the process of motion.

## Supporting information

S1 File (ZIP)
