# Supplementary material for: Analysis of sinusoidal post-buckling deformation of horizontal coiled tubing with initial residual bending
Source: PLoS One. 2024 May 14;19(5):e0301610. doi: 10.1371/journal.pone.0301610 (PMC11093391; doi:10.1371/journal.pone.0301610)
Supplement: S1 File — (ZIP) [file pone.0301610.s001.zip › The values used to build graphs - Fig 9.docx]

## The values used to build graphs

The minimal data set of the original data for plotting curves in Fig 9 is as follows:

| x-axis | Separation variable |
| --- | --- |
| 0 | -0.1346 |
| 0.001 | -0.6497 |
| 0.002 | -0.8124 |
| 0.003 | -0.8648 |
| 0.004 | -0.8798 |
| 0.005 | -0.8798 |
| 0.006 | -0.8728 |
| 0.007 | -0.8622 |
| 0.008 | -0.8496 |
| 0.009 | -0.8358 |
| 0.01 | -0.8211 |
| 0.011 | -0.806 |
| 0.012 | -0.7904 |
| 0.013 | -0.7746 |
| 0.014 | -0.7586 |
| 0.015 | -0.7425 |
| 0.016 | -0.7262 |
| 0.017 | -0.7099 |
| 0.018 | -0.6935 |
| 0.019 | -0.677 |
| 0.02 | -0.6605 |
| 0.021 | -0.644 |
| 0.022 | -0.6274 |
| 0.023 | -0.6108 |
| 0.024 | -0.5941 |
| 0.025 | -0.5775 |
| 0.026 | -0.5608 |
| 0.027 | -0.5441 |
| 0.028 | -0.5274 |
| 0.029 | -0.5107 |
| 0.03 | -0.494 |
| 0.031 | -0.4773 |
| 0.032 | -0.4605 |
| 0.033 | -0.4438 |
| 0.034 | -0.427 |
| 0.035 | -0.4102 |
| 0.036 | -0.3935 |
| 0.037 | -0.3767 |
| 0.038 | -0.3599 |
| 0.039 | -0.3431 |
| 0.04 | -0.3263 |
| 0.041 | -0.3095 |
| 0.042 | -0.2927 |
| 0.043 | -0.2759 |
| 0.044 | -0.259 |
| 0.045 | -0.2422 |
| 0.046 | -0.2254 |
| 0.047 | -0.2085 |
| 0.048 | -0.1917 |
| 0.049 | -0.1748 |
| 0.05 | -0.158 |
| 0.051 | -0.1411 |
| 0.052 | -0.1243 |
| 0.053 | -0.1074 |
| 0.054 | -0.0906 |
| 0.055 | -0.0737 |
| 0.056 | -0.0568 |
| 0.057 | -0.0399 |
| 0.058 | -0.023 |
| 0.059 | -0.0062 |
| 0.06 | 0.0107 |
| 0.061 | 0.0276 |
| 0.062 | 0.0445 |
| 0.063 | 0.0614 |
| 0.064 | 0.0783 |
| 0.065 | 0.0952 |
| 0.066 | 0.1122 |
| 0.067 | 0.1291 |
| 0.068 | 0.146 |
| 0.069 | 0.1629 |
| 0.07 | 0.1798 |
| 0.071 | 0.1968 |
| 0.072 | 0.2137 |
| 0.073 | 0.2307 |
| 0.074 | 0.2476 |
| 0.075 | 0.2645 |
| 0.076 | 0.2815 |
| 0.077 | 0.2984 |
| 0.078 | 0.3154 |
| 0.079 | 0.3324 |
| 0.08 | 0.3493 |
| 0.081 | 0.3663 |
| 0.082 | 0.3833 |
| 0.083 | 0.4002 |
| 0.084 | 0.4172 |
| 0.085 | 0.4342 |
| 0.086 | 0.4512 |
| 0.087 | 0.4682 |
| 0.088 | 0.4852 |
| 0.089 | 0.5022 |
| 0.09 | 0.5192 |
| 0.091 | 0.5362 |
| 0.092 | 0.5532 |
| 0.093 | 0.5702 |
| 0.094 | 0.5872 |
| 0.095 | 0.6042 |
| 0.096 | 0.6212 |
| 0.097 | 0.6383 |
| 0.098 | 0.6553 |
| 0.099 | 0.6723 |
| 0.1 | 0.6894 |
| 0.101 | 0.7064 |
| 0.102 | 0.7234 |
| 0.103 | 0.7405 |
| 0.104 | 0.7575 |
| 0.105 | 0.7746 |
| 0.106 | 0.7916 |
| 0.107 | 0.8087 |
| 0.108 | 0.8258 |
| 0.109 | 0.8428 |
| 0.11 | 0.8599 |
| 0.111 | 0.877 |
| 0.112 | 0.8941 |
| 0.113 | 0.9111 |
| 0.114 | 0.9282 |
| 0.115 | 0.9453 |
| 0.116 | 0.9624 |
| 0.117 | 0.9795 |
| 0.118 | 0.9966 |
| 0.119 | 1.0137 |
| 0.12 | 1.0308 |
| 0.121 | 1.0479 |
| 0.122 | 1.065 |
| 0.123 | 1.0821 |
| 0.124 | 1.0992 |
| 0.125 | 1.1164 |
| 0.126 | 1.1335 |
| 0.127 | 1.1506 |
| 0.128 | 1.1678 |
| 0.129 | 1.1849 |
| 0.13 | 1.202 |
| 0.131 | 1.2192 |
| 0.132 | 1.2363 |
| 0.133 | 1.2535 |
| 0.134 | 1.2706 |
| 0.135 | 1.2878 |
| 0.136 | 1.3049 |
| 0.137 | 1.3221 |
| 0.138 | 1.3393 |
| 0.139 | 1.3564 |
| 0.14 | 1.3736 |
| 0.141 | 1.3908 |
| 0.142 | 1.408 |
| 0.143 | 1.4252 |
| 0.144 | 1.4424 |
| 0.145 | 1.4595 |
| 0.146 | 1.4767 |
| 0.147 | 1.4939 |
| 0.148 | 1.5111 |
| 0.149 | 1.5284 |
| 0.15 | 1.5456 |
| 0.151 | 1.5628 |
| 0.152 | 1.58 |
| 0.153 | 1.5972 |
| 0.154 | 1.6144 |
| 0.155 | 1.6317 |
| 0.156 | 1.6489 |
| 0.157 | 1.6661 |
| 0.158 | 1.6834 |
| 0.159 | 1.7006 |
| 0.16 | 1.7179 |
| 0.161 | 1.7351 |
| 0.162 | 1.7524 |
| 0.163 | 1.7696 |
| 0.164 | 1.7869 |
| 0.165 | 1.8041 |
| 0.166 | 1.8214 |
| 0.167 | 1.8387 |
| 0.168 | 1.8559 |
| 0.169 | 1.8732 |
| 0.17 | 1.8905 |
| 0.171 | 1.9078 |
| 0.172 | 1.9251 |
| 0.173 | 1.9424 |
| 0.174 | 1.9596 |
| 0.175 | 1.9769 |
| 0.176 | 1.9942 |
| 0.177 | 2.0116 |
| 0.178 | 2.0289 |
| 0.179 | 2.0462 |
| 0.18 | 2.0635 |
| 0.181 | 2.0808 |
| 0.182 | 2.0981 |
| 0.183 | 2.1155 |
| 0.184 | 2.1328 |
| 0.185 | 2.1501 |
| 0.186 | 2.1675 |
| 0.187 | 2.1848 |
| 0.188 | 2.2021 |
| 0.189 | 2.2195 |
| 0.19 | 2.2368 |
| 0.191 | 2.2542 |
| 0.192 | 2.2715 |
| 0.193 | 2.2889 |
| 0.194 | 2.3063 |
| 0.195 | 2.3236 |
| 0.196 | 2.341 |
| 0.197 | 2.3584 |
| 0.198 | 2.3758 |
| 0.199 | 2.3931 |
| 0.2 | 2.4105 |
