# Supplementary material for: Analysis of sinusoidal post-buckling deformation of horizontal coiled tubing with initial residual bending
Source: PLoS One. 2024 May 14;19(5):e0301610. doi: 10.1371/journal.pone.0301610 (PMC11093391; doi:10.1371/journal.pone.0301610)
Supplement: S1 File — (ZIP) [file pone.0301610.s001.zip › The values used to build graphs - Fig 8 (b).docx]

## The values used to build graphs

The minimal data set of the original data for plotting curves in Fig 8 (b) is as follows:

| x-axis | m=2 | m=4 | m=6 | m=12 |
| --- | --- | --- | --- | --- |
| 0 | -0.0059 | -0.0233 | -0.0514 | -0.186 |
| 0.0005 | -0.228 | -0.2409 | -0.2616 | -0.3589 |
| 0.001 | -0.398 | -0.4071 | -0.4218 | -0.4888 |
| 0.0015 | -0.527 | -0.5331 | -0.5429 | -0.5857 |
| 0.002 | -0.6245 | -0.6283 | -0.6342 | -0.6576 |
| 0.0025 | -0.6984 | -0.7003 | -0.703 | -0.711 |
| 0.003 | -0.7547 | -0.755 | -0.7553 | -0.7505 |
| 0.0035 | -0.7979 | -0.797 | -0.7951 | -0.7798 |
| 0.004 | -0.8315 | -0.8294 | -0.8258 | -0.8014 |
| 0.0045 | -0.8578 | -0.8548 | -0.8497 | -0.8174 |
| 0.005 | -0.8786 | -0.8748 | -0.8683 | -0.829 |
| 0.0055 | -0.8954 | -0.8908 | -0.8831 | -0.8372 |
| 0.006 | -0.9089 | -0.9037 | -0.8948 | -0.8429 |
| 0.0065 | -0.92 | -0.9141 | -0.9042 | -0.8465 |
| 0.007 | -0.9292 | -0.9227 | -0.9117 | -0.8486 |
| 0.0075 | -0.9368 | -0.9297 | -0.9178 | -0.8494 |
| 0.008 | -0.9433 | -0.9356 | -0.9226 | -0.8491 |
| 0.0085 | -0.9487 | -0.9405 | -0.9266 | -0.8481 |
| 0.009 | -0.9533 | -0.9445 | -0.9297 | -0.8463 |
| 0.0095 | -0.9573 | -0.948 | -0.9323 | -0.844 |
| 0.01 | -0.9607 | -0.9508 | -0.9342 | -0.8412 |
| 0.0105 | -0.9637 | -0.9533 | -0.9358 | -0.838 |
| 0.011 | -0.9662 | -0.9553 | -0.937 | -0.8344 |
| 0.0115 | -0.9685 | -0.9571 | -0.9378 | -0.8306 |
| 0.012 | -0.9705 | -0.9585 | -0.9384 | -0.8265 |
| 0.0125 | -0.9722 | -0.9597 | -0.9388 | -0.8223 |
| 0.013 | -0.9737 | -0.9608 | -0.939 | -0.8178 |
| 0.0135 | -0.9751 | -0.9616 | -0.9389 | -0.8132 |
| 0.014 | -0.9763 | -0.9623 | -0.9388 | -0.8084 |
| 0.0145 | -0.9774 | -0.9629 | -0.9385 | -0.8036 |
| 0.015 | -0.9784 | -0.9633 | -0.9381 | -0.7986 |
| 0.0155 | -0.9792 | -0.9637 | -0.9376 | -0.7935 |
| 0.016 | -0.98 | -0.9639 | -0.937 | -0.7884 |
| 0.0165 | -0.9807 | -0.9641 | -0.9364 | -0.7831 |
| 0.017 | -0.9813 | -0.9642 | -0.9356 | -0.7778 |
| 0.0175 | -0.9818 | -0.9643 | -0.9348 | -0.7725 |
| 0.018 | -0.9823 | -0.9642 | -0.934 | -0.7671 |
| 0.0185 | -0.9828 | -0.9642 | -0.9331 | -0.7616 |
| 0.019 | -0.9831 | -0.9641 | -0.9321 | -0.7561 |
| 0.0195 | -0.9835 | -0.9639 | -0.9311 | -0.7506 |
| 0.02 | -0.9838 | -0.9637 | -0.9301 | -0.745 |
| 0.0205 | -0.9841 | -0.9635 | -0.929 | -0.7394 |
| 0.021 | -0.9844 | -0.9633 | -0.9279 | -0.7338 |
| 0.0215 | -0.9846 | -0.963 | -0.9268 | -0.7282 |
| 0.022 | -0.9848 | -0.9627 | -0.9257 | -0.7225 |
| 0.0225 | -0.9849 | -0.9623 | -0.9245 | -0.7168 |
| 0.023 | -0.9851 | -0.962 | -0.9233 | -0.7111 |
| 0.0235 | -0.9852 | -0.9616 | -0.9221 | -0.7053 |
| 0.024 | -0.9853 | -0.9612 | -0.9209 | -0.6996 |
| 0.0245 | -0.9854 | -0.9608 | -0.9196 | -0.6938 |
| 0.025 | -0.9855 | -0.9604 | -0.9184 | -0.688 |
| 0.0255 | -0.9856 | -0.96 | -0.9171 | -0.6822 |
| 0.026 | -0.9856 | -0.9595 | -0.9158 | -0.6764 |
| 0.0265 | -0.9857 | -0.959 | -0.9145 | -0.6706 |
| 0.027 | -0.9857 | -0.9586 | -0.9132 | -0.6647 |
| 0.0275 | -0.9857 | -0.9581 | -0.9119 | -0.6589 |
| 0.028 | -0.9857 | -0.9576 | -0.9105 | -0.653 |
| 0.0285 | -0.9857 | -0.9571 | -0.9092 | -0.6472 |
| 0.029 | -0.9857 | -0.9566 | -0.9078 | -0.6413 |
| 0.0295 | -0.9857 | -0.9561 | -0.9065 | -0.6354 |
| 0.03 | -0.9857 | -0.9555 | -0.9051 | -0.6295 |
| 0.0305 | -0.9857 | -0.955 | -0.9038 | -0.6236 |
| 0.031 | -0.9856 | -0.9545 | -0.9024 | -0.6177 |
| 0.0315 | -0.9856 | -0.9539 | -0.901 | -0.6118 |
| 0.032 | -0.9855 | -0.9534 | -0.8996 | -0.6059 |
| 0.0325 | -0.9855 | -0.9528 | -0.8982 | -0.6 |
| 0.033 | -0.9854 | -0.9523 | -0.8968 | -0.594 |
| 0.0335 | -0.9854 | -0.9517 | -0.8954 | -0.5881 |
| 0.034 | -0.9853 | -0.9511 | -0.894 | -0.5822 |
| 0.0345 | -0.9852 | -0.9505 | -0.8926 | -0.5762 |
| 0.035 | -0.9851 | -0.95 | -0.8912 | -0.5703 |
| 0.0355 | -0.9851 | -0.9494 | -0.8897 | -0.5643 |
| 0.036 | -0.985 | -0.9488 | -0.8883 | -0.5584 |
| 0.0365 | -0.9849 | -0.9482 | -0.8869 | -0.5524 |
| 0.037 | -0.9848 | -0.9476 | -0.8855 | -0.5465 |
| 0.0375 | -0.9847 | -0.947 | -0.884 | -0.5405 |
| 0.038 | -0.9846 | -0.9464 | -0.8826 | -0.5345 |
| 0.0385 | -0.9845 | -0.9458 | -0.8811 | -0.5286 |
| 0.039 | -0.9844 | -0.9452 | -0.8797 | -0.5226 |
| 0.0395 | -0.9843 | -0.9446 | -0.8783 | -0.5166 |
| 0.04 | -0.9842 | -0.944 | -0.8768 | -0.5106 |
| 0.0405 | -0.9841 | -0.9434 | -0.8754 | -0.5047 |
| 0.041 | -0.984 | -0.9428 | -0.8739 | -0.4987 |
| 0.0415 | -0.9839 | -0.9421 | -0.8724 | -0.4927 |
| 0.042 | -0.9838 | -0.9415 | -0.871 | -0.4867 |
| 0.0425 | -0.9836 | -0.9409 | -0.8695 | -0.4807 |
| 0.043 | -0.9835 | -0.9403 | -0.8681 | -0.4747 |
| 0.0435 | -0.9834 | -0.9397 | -0.8666 | -0.4687 |
| 0.044 | -0.9833 | -0.939 | -0.8651 | -0.4628 |
| 0.0445 | -0.9832 | -0.9384 | -0.8637 | -0.4568 |
| 0.045 | -0.983 | -0.9378 | -0.8622 | -0.4508 |
| 0.0455 | -0.9829 | -0.9372 | -0.8607 | -0.4448 |
| 0.046 | -0.9828 | -0.9365 | -0.8593 | -0.4388 |
| 0.0465 | -0.9826 | -0.9359 | -0.8578 | -0.4328 |
| 0.047 | -0.9825 | -0.9353 | -0.8563 | -0.4268 |
| 0.0475 | -0.9824 | -0.9346 | -0.8549 | -0.4208 |
| 0.048 | -0.9823 | -0.934 | -0.8534 | -0.4148 |
| 0.0485 | -0.9821 | -0.9333 | -0.8519 | -0.4087 |
| 0.049 | -0.982 | -0.9327 | -0.8504 | -0.4027 |
| 0.0495 | -0.9819 | -0.9321 | -0.849 | -0.3967 |
| 0.05 | -0.9817 | -0.9314 | -0.8475 | -0.3907 |
| 0.0505 | -0.9816 | -0.9308 | -0.846 | -0.3847 |
| 0.051 | -0.9814 | -0.9301 | -0.8445 | -0.3787 |
| 0.0515 | -0.9813 | -0.9295 | -0.843 | -0.3727 |
| 0.052 | -0.9812 | -0.9289 | -0.8415 | -0.3667 |
| 0.0525 | -0.981 | -0.9282 | -0.8401 | -0.3606 |
| 0.053 | -0.9809 | -0.9276 | -0.8386 | -0.3546 |
| 0.0535 | -0.9807 | -0.9269 | -0.8371 | -0.3486 |
| 0.054 | -0.9806 | -0.9263 | -0.8356 | -0.3426 |
| 0.0545 | -0.9804 | -0.9256 | -0.8341 | -0.3366 |
| 0.055 | -0.9803 | -0.925 | -0.8326 | -0.3305 |
| 0.0555 | -0.9802 | -0.9243 | -0.8311 | -0.3245 |
| 0.056 | -0.98 | -0.9237 | -0.8297 | -0.3185 |
| 0.0565 | -0.9799 | -0.923 | -0.8282 | -0.3125 |
| 0.057 | -0.9797 | -0.9224 | -0.8267 | -0.3064 |
| 0.0575 | -0.9796 | -0.9217 | -0.8252 | -0.3004 |
| 0.058 | -0.9794 | -0.9211 | -0.8237 | -0.2944 |
| 0.0585 | -0.9793 | -0.9204 | -0.8222 | -0.2884 |
| 0.059 | -0.9791 | -0.9198 | -0.8207 | -0.2823 |
| 0.0595 | -0.979 | -0.9191 | -0.8192 | -0.2763 |
| 0.06 | -0.9788 | -0.9185 | -0.8177 | -0.2703 |
| 0.0605 | -0.9787 | -0.9178 | -0.8162 | -0.2642 |
| 0.061 | -0.9785 | -0.9172 | -0.8147 | -0.2582 |
| 0.0615 | -0.9784 | -0.9165 | -0.8132 | -0.2522 |
| 0.062 | -0.9782 | -0.9158 | -0.8117 | -0.2461 |
| 0.0625 | -0.9781 | -0.9152 | -0.8102 | -0.2401 |
| 0.063 | -0.9779 | -0.9145 | -0.8087 | -0.2341 |
| 0.0635 | -0.9778 | -0.9139 | -0.8072 | -0.228 |
| 0.064 | -0.9776 | -0.9132 | -0.8057 | -0.222 |
| 0.0645 | -0.9775 | -0.9126 | -0.8042 | -0.216 |
| 0.065 | -0.9773 | -0.9119 | -0.8028 | -0.2099 |
| 0.0655 | -0.9772 | -0.9112 | -0.8013 | -0.2039 |
| 0.066 | -0.977 | -0.9106 | -0.7998 | -0.1979 |
| 0.0665 | -0.9768 | -0.9099 | -0.7983 | -0.1918 |
| 0.067 | -0.9767 | -0.9093 | -0.7968 | -0.1858 |
| 0.0675 | -0.9765 | -0.9086 | -0.7953 | -0.1797 |
| 0.068 | -0.9764 | -0.9079 | -0.7938 | -0.1737 |
| 0.0685 | -0.9762 | -0.9073 | -0.7923 | -0.1677 |
| 0.069 | -0.9761 | -0.9066 | -0.7907 | -0.1616 |
| 0.0695 | -0.9759 | -0.906 | -0.7892 | -0.1556 |
| 0.07 | -0.9757 | -0.9053 | -0.7877 | -0.1495 |
| 0.0705 | -0.9756 | -0.9046 | -0.7862 | -0.1435 |
| 0.071 | -0.9754 | -0.904 | -0.7847 | -0.1374 |
| 0.0715 | -0.9753 | -0.9033 | -0.7832 | -0.1314 |
| 0.072 | -0.9751 | -0.9027 | -0.7817 | -0.1254 |
| 0.0725 | -0.975 | -0.902 | -0.7802 | -0.1193 |
| 0.073 | -0.9748 | -0.9013 | -0.7787 | -0.1133 |
| 0.0735 | -0.9746 | -0.9007 | -0.7772 | -0.1072 |
| 0.074 | -0.9745 | -0.9 | -0.7757 | -0.1012 |
| 0.0745 | -0.9743 | -0.8993 | -0.7742 | -0.0951 |
| 0.075 | -0.9742 | -0.8987 | -0.7727 | -0.0891 |
| 0.0755 | -0.974 | -0.898 | -0.7712 | -0.083 |
| 0.076 | -0.9739 | -0.8974 | -0.7697 | -0.077 |
| 0.0765 | -0.9737 | -0.8967 | -0.7682 | -0.0709 |
| 0.077 | -0.9735 | -0.896 | -0.7667 | -0.0649 |
| 0.0775 | -0.9734 | -0.8954 | -0.7652 | -0.0588 |
| 0.078 | -0.9732 | -0.8947 | -0.7637 | -0.0528 |
| 0.0785 | -0.9731 | -0.894 | -0.7622 | -0.0467 |
| 0.079 | -0.9729 | -0.8934 | -0.7607 | -0.0407 |
| 0.0795 | -0.9727 | -0.8927 | -0.7592 | -0.0346 |
| 0.08 | -0.9726 | -0.892 | -0.7576 | -0.0286 |
| 0.0805 | -0.9724 | -0.8914 | -0.7561 | -0.0225 |
| 0.081 | -0.9722 | -0.8907 | -0.7546 | -0.0165 |
| 0.0815 | -0.9721 | -0.89 | -0.7531 | -0.0104 |
| 0.082 | -0.9719 | -0.8894 | -0.7516 | -0.0044 |
| 0.0825 | -0.9718 | -0.8887 | -0.7501 | 0.0017 |
| 0.083 | -0.9716 | -0.888 | -0.7486 | 0.0077 |
| 0.0835 | -0.9714 | -0.8874 | -0.7471 | 0.0138 |
| 0.084 | -0.9713 | -0.8867 | -0.7456 | 0.0199 |
| 0.0845 | -0.9711 | -0.886 | -0.7441 | 0.0259 |
| 0.085 | -0.971 | -0.8854 | -0.7426 | 0.032 |
| 0.0855 | -0.9708 | -0.8847 | -0.741 | 0.038 |
| 0.086 | -0.9706 | -0.884 | -0.7395 | 0.0441 |
| 0.0865 | -0.9705 | -0.8834 | -0.738 | 0.0501 |
| 0.087 | -0.9703 | -0.8827 | -0.7365 | 0.0562 |
| 0.0875 | -0.9701 | -0.882 | -0.735 | 0.0623 |
| 0.088 | -0.97 | -0.8814 | -0.7335 | 0.0683 |
| 0.0885 | -0.9698 | -0.8807 | -0.732 | 0.0744 |
| 0.089 | -0.9697 | -0.88 | -0.7305 | 0.0804 |
| 0.0895 | -0.9695 | -0.8794 | -0.729 | 0.0865 |
| 0.09 | -0.9693 | -0.8787 | -0.7275 | 0.0926 |
| 0.0905 | -0.9692 | -0.878 | -0.7259 | 0.0986 |
| 0.091 | -0.969 | -0.8773 | -0.7244 | 0.1047 |
| 0.0915 | -0.9688 | -0.8767 | -0.7229 | 0.1107 |
| 0.092 | -0.9687 | -0.876 | -0.7214 | 0.1168 |
| 0.0925 | -0.9685 | -0.8753 | -0.7199 | 0.1229 |
| 0.093 | -0.9683 | -0.8747 | -0.7184 | 0.1289 |
| 0.0935 | -0.9682 | -0.874 | -0.7169 | 0.135 |
| 0.094 | -0.968 | -0.8733 | -0.7154 | 0.1411 |
| 0.0945 | -0.9679 | -0.8727 | -0.7138 | 0.1471 |
| 0.095 | -0.9677 | -0.872 | -0.7123 | 0.1532 |
| 0.0955 | -0.9675 | -0.8713 | -0.7108 | 0.1593 |
| 0.096 | -0.9674 | -0.8707 | -0.7093 | 0.1653 |
| 0.0965 | -0.9672 | -0.87 | -0.7078 | 0.1714 |
| 0.097 | -0.967 | -0.8693 | -0.7063 | 0.1775 |
| 0.0975 | -0.9669 | -0.8686 | -0.7048 | 0.1835 |
| 0.098 | -0.9667 | -0.868 | -0.7033 | 0.1896 |
| 0.0985 | -0.9665 | -0.8673 | -0.7017 | 0.1957 |
| 0.099 | -0.9664 | -0.8666 | -0.7002 | 0.2017 |
| 0.0995 | -0.9662 | -0.866 | -0.6987 | 0.2078 |
| 0.1 | -0.966 | -0.8653 | -0.6972 | 0.2139 |
