# Supplementary material for: Analysis of sinusoidal post-buckling deformation of horizontal coiled tubing with initial residual bending
Source: PLoS One. 2024 May 14;19(5):e0301610. doi: 10.1371/journal.pone.0301610 (PMC11093391; doi:10.1371/journal.pone.0301610)
Supplement: S1 File — (ZIP) [file pone.0301610.s001.zip › The values used to build graphs - Fig 8 (a).docx]

## The values used to build graphs

The minimal data set of the original data for plotting curves in Fig 8 (a) is as follows:

| x-axis | ‾*L*_0_ = 25 | ‾*L*_0_ = 20 | ‾*L*_0_ = 15 | ‾*L*_0_ = 10 |
| --- | --- | --- | --- | --- |
| 0 | -0.0233 | -0.0361 | -0.063 | -0.1346 |
| 0.0005 | -0.2018 | -0.2503 | -0.3276 | -0.4639 |
| 0.001 | -0.346 | -0.4138 | -0.5108 | -0.6497 |
| 0.0015 | -0.4618 | -0.5376 | -0.6365 | -0.7536 |
| 0.002 | -0.5544 | -0.631 | -0.7227 | -0.8124 |
| 0.0025 | -0.6283 | -0.7016 | -0.7824 | -0.8459 |
| 0.003 | -0.6874 | -0.7552 | -0.8242 | -0.8648 |
| 0.0035 | -0.7349 | -0.7962 | -0.8539 | -0.8751 |
| 0.004 | -0.7731 | -0.8278 | -0.8754 | -0.8798 |
| 0.0045 | -0.8041 | -0.8525 | -0.891 | -0.881 |
| 0.005 | -0.8294 | -0.8719 | -0.9024 | -0.8798 |
| 0.0055 | -0.8502 | -0.8874 | -0.9109 | -0.8769 |
| 0.006 | -0.8674 | -0.8997 | -0.9171 | -0.8728 |
| 0.0065 | -0.8817 | -0.9097 | -0.9217 | -0.8678 |
| 0.007 | -0.8936 | -0.9178 | -0.9249 | -0.8622 |
| 0.0075 | -0.9037 | -0.9244 | -0.9272 | -0.8561 |
| 0.008 | -0.9122 | -0.9298 | -0.9287 | -0.8496 |
| 0.0085 | -0.9195 | -0.9342 | -0.9295 | -0.8428 |
| 0.009 | -0.9257 | -0.9379 | -0.9299 | -0.8358 |
| 0.0095 | -0.931 | -0.9409 | -0.9298 | -0.8285 |
| 0.01 | -0.9356 | -0.9434 | -0.9295 | -0.8211 |
| 0.0105 | -0.9396 | -0.9454 | -0.9288 | -0.8136 |
| 0.011 | -0.943 | -0.9471 | -0.9279 | -0.806 |
| 0.0115 | -0.946 | -0.9484 | -0.9268 | -0.7982 |
| 0.012 | -0.9486 | -0.9495 | -0.9256 | -0.7904 |
| 0.0125 | -0.9508 | -0.9503 | -0.9242 | -0.7825 |
| 0.013 | -0.9528 | -0.951 | -0.9227 | -0.7746 |
| 0.0135 | -0.9545 | -0.9514 | -0.9211 | -0.7666 |
| 0.014 | -0.956 | -0.9517 | -0.9194 | -0.7586 |
| 0.0145 | -0.9574 | -0.9519 | -0.9177 | -0.7506 |
| 0.015 | -0.9585 | -0.952 | -0.9158 | -0.7425 |
| 0.0155 | -0.9595 | -0.952 | -0.9139 | -0.7344 |
| 0.016 | -0.9604 | -0.9518 | -0.912 | -0.7262 |
| 0.0165 | -0.9611 | -0.9516 | -0.91 | -0.7181 |
| 0.017 | -0.9618 | -0.9514 | -0.9079 | -0.7099 |
| 0.0175 | -0.9623 | -0.951 | -0.9059 | -0.7017 |
| 0.018 | -0.9628 | -0.9506 | -0.9038 | -0.6935 |
| 0.0185 | -0.9632 | -0.9502 | -0.9016 | -0.6853 |
| 0.019 | -0.9635 | -0.9497 | -0.8995 | -0.677 |
| 0.0195 | -0.9637 | -0.9492 | -0.8973 | -0.6688 |
| 0.02 | -0.9639 | -0.9486 | -0.8951 | -0.6605 |
| 0.0205 | -0.9641 | -0.948 | -0.8929 | -0.6522 |
| 0.021 | -0.9642 | -0.9474 | -0.8906 | -0.644 |
| 0.0215 | -0.9642 | -0.9467 | -0.8883 | -0.6357 |
| 0.022 | -0.9643 | -0.946 | -0.8861 | -0.6274 |
| 0.0225 | -0.9642 | -0.9453 | -0.8838 | -0.6191 |
| 0.023 | -0.9642 | -0.9446 | -0.8815 | -0.6108 |
| 0.0235 | -0.9641 | -0.9439 | -0.8792 | -0.6025 |
| 0.024 | -0.964 | -0.9431 | -0.8768 | -0.5941 |
| 0.0245 | -0.9639 | -0.9423 | -0.8745 | -0.5858 |
| 0.025 | -0.9637 | -0.9415 | -0.8722 | -0.5775 |
| 0.0255 | -0.9636 | -0.9407 | -0.8698 | -0.5692 |
| 0.026 | -0.9634 | -0.9399 | -0.8675 | -0.5608 |
| 0.0265 | -0.9631 | -0.939 | -0.8651 | -0.5525 |
| 0.027 | -0.9629 | -0.9382 | -0.8627 | -0.5441 |
| 0.0275 | -0.9627 | -0.9373 | -0.8603 | -0.5358 |
| 0.028 | -0.9624 | -0.9364 | -0.858 | -0.5274 |
| 0.0285 | -0.9621 | -0.9356 | -0.8556 | -0.5191 |
| 0.029 | -0.9618 | -0.9347 | -0.8532 | -0.5107 |
| 0.0295 | -0.9615 | -0.9338 | -0.8508 | -0.5024 |
| 0.03 | -0.9612 | -0.9329 | -0.8484 | -0.494 |
| 0.0305 | -0.9609 | -0.932 | -0.846 | -0.4856 |
| 0.031 | -0.9606 | -0.9311 | -0.8436 | -0.4773 |
| 0.0315 | -0.9602 | -0.9301 | -0.8412 | -0.4689 |
| 0.032 | -0.9599 | -0.9292 | -0.8387 | -0.4605 |
| 0.0325 | -0.9595 | -0.9283 | -0.8363 | -0.4521 |
| 0.033 | -0.9591 | -0.9273 | -0.8339 | -0.4438 |
| 0.0335 | -0.9588 | -0.9264 | -0.8315 | -0.4354 |
| 0.034 | -0.9584 | -0.9254 | -0.829 | -0.427 |
| 0.0345 | -0.958 | -0.9245 | -0.8266 | -0.4186 |
| 0.035 | -0.9576 | -0.9235 | -0.8242 | -0.4102 |
| 0.0355 | -0.9572 | -0.9226 | -0.8217 | -0.4018 |
| 0.036 | -0.9568 | -0.9216 | -0.8193 | -0.3935 |
| 0.0365 | -0.9564 | -0.9206 | -0.8169 | -0.3851 |
| 0.037 | -0.956 | -0.9197 | -0.8144 | -0.3767 |
| 0.0375 | -0.9555 | -0.9187 | -0.812 | -0.3683 |
| 0.038 | -0.9551 | -0.9177 | -0.8095 | -0.3599 |
| 0.0385 | -0.9547 | -0.9167 | -0.8071 | -0.3515 |
| 0.039 | -0.9543 | -0.9158 | -0.8046 | -0.3431 |
| 0.0395 | -0.9538 | -0.9148 | -0.8022 | -0.3347 |
| 0.04 | -0.9534 | -0.9138 | -0.7997 | -0.3263 |
| 0.0405 | -0.9529 | -0.9128 | -0.7973 | -0.3179 |
| 0.041 | -0.9525 | -0.9118 | -0.7948 | -0.3095 |
| 0.0415 | -0.952 | -0.9108 | -0.7923 | -0.3011 |
| 0.042 | -0.9516 | -0.9098 | -0.7899 | -0.2927 |
| 0.0425 | -0.9511 | -0.9088 | -0.7874 | -0.2843 |
| 0.043 | -0.9507 | -0.9078 | -0.785 | -0.2759 |
| 0.0435 | -0.9502 | -0.9068 | -0.7825 | -0.2674 |
| 0.044 | -0.9497 | -0.9058 | -0.78 | -0.259 |
| 0.0445 | -0.9493 | -0.9048 | -0.7776 | -0.2506 |
| 0.045 | -0.9488 | -0.9038 | -0.7751 | -0.2422 |
| 0.0455 | -0.9483 | -0.9028 | -0.7727 | -0.2338 |
| 0.046 | -0.9478 | -0.9018 | -0.7702 | -0.2254 |
| 0.0465 | -0.9474 | -0.9008 | -0.7677 | -0.217 |
| 0.047 | -0.9469 | -0.8998 | -0.7653 | -0.2085 |
| 0.0475 | -0.9464 | -0.8988 | -0.7628 | -0.2001 |
| 0.048 | -0.9459 | -0.8977 | -0.7603 | -0.1917 |
| 0.0485 | -0.9454 | -0.8967 | -0.7578 | -0.1833 |
| 0.049 | -0.945 | -0.8957 | -0.7554 | -0.1748 |
| 0.0495 | -0.9445 | -0.8947 | -0.7529 | -0.1664 |
| 0.05 | -0.944 | -0.8937 | -0.7504 | -0.158 |
| 0.0505 | -0.9435 | -0.8927 | -0.748 | -0.1496 |
| 0.051 | -0.943 | -0.8916 | -0.7455 | -0.1411 |
| 0.0515 | -0.9425 | -0.8906 | -0.743 | -0.1327 |
| 0.052 | -0.942 | -0.8896 | -0.7405 | -0.1243 |
| 0.0525 | -0.9415 | -0.8886 | -0.738 | -0.1159 |
| 0.053 | -0.941 | -0.8875 | -0.7356 | -0.1074 |
| 0.0535 | -0.9405 | -0.8865 | -0.7331 | -0.099 |
| 0.054 | -0.94 | -0.8855 | -0.7306 | -0.0906 |
| 0.0545 | -0.9395 | -0.8845 | -0.7281 | -0.0821 |
| 0.055 | -0.939 | -0.8834 | -0.7257 | -0.0737 |
| 0.0555 | -0.9385 | -0.8824 | -0.7232 | -0.0652 |
| 0.056 | -0.938 | -0.8814 | -0.7207 | -0.0568 |
| 0.0565 | -0.9375 | -0.8804 | -0.7182 | -0.0484 |
| 0.057 | -0.937 | -0.8793 | -0.7157 | -0.0399 |
| 0.0575 | -0.9365 | -0.8783 | -0.7133 | -0.0315 |
| 0.058 | -0.936 | -0.8773 | -0.7108 | -0.023 |
| 0.0585 | -0.9355 | -0.8762 | -0.7083 | -0.0146 |
| 0.059 | -0.935 | -0.8752 | -0.7058 | -0.0062 |
| 0.0595 | -0.9345 | -0.8742 | -0.7033 | 0.0023 |
| 0.06 | -0.934 | -0.8732 | -0.7008 | 0.0107 |
| 0.0605 | -0.9335 | -0.8721 | -0.6984 | 0.0192 |
| 0.061 | -0.933 | -0.8711 | -0.6959 | 0.0276 |
| 0.0615 | -0.9325 | -0.8701 | -0.6934 | 0.0361 |
| 0.062 | -0.9319 | -0.869 | -0.6909 | 0.0445 |
| 0.0625 | -0.9314 | -0.868 | -0.6884 | 0.053 |
| 0.063 | -0.9309 | -0.867 | -0.6859 | 0.0614 |
| 0.0635 | -0.9304 | -0.8659 | -0.6834 | 0.0699 |
| 0.064 | -0.9299 | -0.8649 | -0.681 | 0.0783 |
| 0.0645 | -0.9294 | -0.8638 | -0.6785 | 0.0868 |
| 0.065 | -0.9289 | -0.8628 | -0.676 | 0.0952 |
| 0.0655 | -0.9283 | -0.8618 | -0.6735 | 0.1037 |
| 0.066 | -0.9278 | -0.8607 | -0.671 | 0.1122 |
| 0.0665 | -0.9273 | -0.8597 | -0.6685 | 0.1206 |
| 0.067 | -0.9268 | -0.8587 | -0.666 | 0.1291 |
| 0.0675 | -0.9263 | -0.8576 | -0.6635 | 0.1375 |
| 0.068 | -0.9258 | -0.8566 | -0.6611 | 0.146 |
| 0.0685 | -0.9252 | -0.8555 | -0.6586 | 0.1545 |
| 0.069 | -0.9247 | -0.8545 | -0.6561 | 0.1629 |
| 0.0695 | -0.9242 | -0.8535 | -0.6536 | 0.1714 |
| 0.07 | -0.9237 | -0.8524 | -0.6511 | 0.1798 |
| 0.0705 | -0.9232 | -0.8514 | -0.6486 | 0.1883 |
| 0.071 | -0.9226 | -0.8503 | -0.6461 | 0.1968 |
| 0.0715 | -0.9221 | -0.8493 | -0.6436 | 0.2052 |
| 0.072 | -0.9216 | -0.8483 | -0.6411 | 0.2137 |
| 0.0725 | -0.9211 | -0.8472 | -0.6386 | 0.2222 |
| 0.073 | -0.9206 | -0.8462 | -0.6361 | 0.2307 |
| 0.0735 | -0.92 | -0.8451 | -0.6336 | 0.2391 |
| 0.074 | -0.9195 | -0.8441 | -0.6312 | 0.2476 |
| 0.0745 | -0.919 | -0.8431 | -0.6287 | 0.2561 |
| 0.075 | -0.9185 | -0.842 | -0.6262 | 0.2645 |
| 0.0755 | -0.9179 | -0.841 | -0.6237 | 0.273 |
| 0.076 | -0.9174 | -0.8399 | -0.6212 | 0.2815 |
| 0.0765 | -0.9169 | -0.8389 | -0.6187 | 0.29 |
| 0.077 | -0.9164 | -0.8378 | -0.6162 | 0.2984 |
| 0.0775 | -0.9158 | -0.8368 | -0.6137 | 0.3069 |
| 0.078 | -0.9153 | -0.8358 | -0.6112 | 0.3154 |
| 0.0785 | -0.9148 | -0.8347 | -0.6087 | 0.3239 |
| 0.079 | -0.9143 | -0.8337 | -0.6062 | 0.3324 |
| 0.0795 | -0.9137 | -0.8326 | -0.6037 | 0.3408 |
| 0.08 | -0.9132 | -0.8316 | -0.6012 | 0.3493 |
| 0.0805 | -0.9127 | -0.8305 | -0.5987 | 0.3578 |
| 0.081 | -0.9122 | -0.8295 | -0.5962 | 0.3663 |
| 0.0815 | -0.9116 | -0.8284 | -0.5937 | 0.3748 |
| 0.082 | -0.9111 | -0.8274 | -0.5912 | 0.3833 |
| 0.0825 | -0.9106 | -0.8264 | -0.5887 | 0.3918 |
| 0.083 | -0.9101 | -0.8253 | -0.5862 | 0.4002 |
| 0.0835 | -0.9095 | -0.8243 | -0.5837 | 0.4087 |
| 0.084 | -0.909 | -0.8232 | -0.5812 | 0.4172 |
| 0.0845 | -0.9085 | -0.8222 | -0.5787 | 0.4257 |
| 0.085 | -0.9079 | -0.8211 | -0.5762 | 0.4342 |
| 0.0855 | -0.9074 | -0.8201 | -0.5737 | 0.4427 |
| 0.086 | -0.9069 | -0.819 | -0.5712 | 0.4512 |
| 0.0865 | -0.9064 | -0.818 | -0.5687 | 0.4597 |
| 0.087 | -0.9058 | -0.8169 | -0.5662 | 0.4682 |
| 0.0875 | -0.9053 | -0.8159 | -0.5637 | 0.4767 |
| 0.088 | -0.9048 | -0.8148 | -0.5612 | 0.4852 |
| 0.0885 | -0.9042 | -0.8138 | -0.5587 | 0.4937 |
| 0.089 | -0.9037 | -0.8127 | -0.5562 | 0.5022 |
| 0.0895 | -0.9032 | -0.8117 | -0.5537 | 0.5107 |
| 0.09 | -0.9027 | -0.8107 | -0.5512 | 0.5192 |
| 0.0905 | -0.9021 | -0.8096 | -0.5487 | 0.5277 |
| 0.091 | -0.9016 | -0.8086 | -0.5462 | 0.5362 |
| 0.0915 | -0.9011 | -0.8075 | -0.5437 | 0.5447 |
| 0.092 | -0.9005 | -0.8065 | -0.5412 | 0.5532 |
| 0.0925 | -0.9 | -0.8054 | -0.5387 | 0.5617 |
| 0.093 | -0.8995 | -0.8044 | -0.5362 | 0.5702 |
| 0.0935 | -0.8989 | -0.8033 | -0.5337 | 0.5787 |
| 0.094 | -0.8984 | -0.8023 | -0.5312 | 0.5872 |
| 0.0945 | -0.8979 | -0.8012 | -0.5287 | 0.5957 |
| 0.095 | -0.8974 | -0.8002 | -0.5262 | 0.6042 |
| 0.0955 | -0.8968 | -0.7991 | -0.5237 | 0.6127 |
| 0.096 | -0.8963 | -0.7981 | -0.5212 | 0.6212 |
| 0.0965 | -0.8958 | -0.797 | -0.5187 | 0.6298 |
| 0.097 | -0.8952 | -0.796 | -0.5162 | 0.6383 |
| 0.0975 | -0.8947 | -0.7949 | -0.5137 | 0.6468 |
| 0.098 | -0.8942 | -0.7939 | -0.5112 | 0.6553 |
| 0.0985 | -0.8936 | -0.7928 | -0.5087 | 0.6638 |
| 0.099 | -0.8931 | -0.7918 | -0.5062 | 0.6723 |
| 0.0995 | -0.8926 | -0.7907 | -0.5037 | 0.6808 |
| 0.1 | -0.892 | -0.7897 | -0.5012 | 0.6894 |
