# Supplementary material for: Analysis of sinusoidal post-buckling deformation of horizontal coiled tubing with initial residual bending
Source: PLoS One. 2024 May 14;19(5):e0301610. doi: 10.1371/journal.pone.0301610 (PMC11093391; doi:10.1371/journal.pone.0301610)
Supplement: S1 File — (ZIP) [file pone.0301610.s001.zip › The values used to build graphs - Fig 7 (c).docx]

## The values used to build graphs

The minimal data set of the original data for plotting curves in Fig 7 (c) is as follows:

| x-axis | *A*_0_ = 0.2 | *A*_0_ = 0.4 | *A*_0_ = 0.6 | *A*_0_ = 0.8 |
| --- | --- | --- | --- | --- |
| 0 | -0.0015 | -0.0058 | -0.0128 | -0.0221 |
| 0.0005 | -0.0509 | -0.055 | -0.0616 | -0.0703 |
| 0.001 | -0.0978 | -0.1017 | -0.1079 | -0.1161 |
| 0.0015 | -0.1424 | -0.146 | -0.1518 | -0.1595 |
| 0.002 | -0.1846 | -0.1881 | -0.1935 | -0.2008 |
| 0.0025 | -0.2247 | -0.2279 | -0.2331 | -0.2398 |
| 0.003 | -0.2627 | -0.2657 | -0.2705 | -0.2768 |
| 0.0035 | -0.2987 | -0.3015 | -0.306 | -0.3119 |
| 0.004 | -0.3328 | -0.3355 | -0.3396 | -0.3451 |
| 0.0045 | -0.3651 | -0.3676 | -0.3715 | -0.3765 |
| 0.005 | -0.3957 | -0.3979 | -0.4016 | -0.4062 |
| 0.0055 | -0.4246 | -0.4267 | -0.43 | -0.4343 |
| 0.006 | -0.4519 | -0.4539 | -0.457 | -0.4609 |
| 0.0065 | -0.4778 | -0.4796 | -0.4825 | -0.4861 |
| 0.007 | -0.5023 | -0.5039 | -0.5065 | -0.5098 |
| 0.0075 | -0.5254 | -0.5269 | -0.5293 | -0.5323 |
| 0.008 | -0.5473 | -0.5487 | -0.5509 | -0.5536 |
| 0.0085 | -0.568 | -0.5692 | -0.5712 | -0.5737 |
| 0.009 | -0.5875 | -0.5887 | -0.5905 | -0.5926 |
| 0.0095 | -0.606 | -0.6071 | -0.6087 | -0.6106 |
| 0.01 | -0.6235 | -0.6245 | -0.6259 | -0.6276 |
| 0.0105 | -0.6401 | -0.6409 | -0.6422 | -0.6436 |
| 0.011 | -0.6558 | -0.6565 | -0.6576 | -0.6588 |
| 0.0115 | -0.6706 | -0.6712 | -0.6722 | -0.6732 |
| 0.012 | -0.6846 | -0.6852 | -0.686 | -0.6868 |
| 0.0125 | -0.6979 | -0.6984 | -0.699 | -0.6996 |
| 0.013 | -0.7105 | -0.7109 | -0.7114 | -0.7118 |
| 0.0135 | -0.7224 | -0.7227 | -0.7231 | -0.7233 |
| 0.014 | -0.7337 | -0.7339 | -0.7342 | -0.7343 |
| 0.0145 | -0.7444 | -0.7446 | -0.7447 | -0.7446 |
| 0.015 | -0.7546 | -0.7547 | -0.7547 | -0.7544 |
| 0.0155 | -0.7642 | -0.7642 | -0.7641 | -0.7637 |
| 0.016 | -0.7734 | -0.7733 | -0.7731 | -0.7726 |
| 0.0165 | -0.7821 | -0.7819 | -0.7816 | -0.7809 |
| 0.017 | -0.7903 | -0.7901 | -0.7897 | -0.7889 |
| 0.0175 | -0.7981 | -0.7979 | -0.7974 | -0.7964 |
| 0.018 | -0.8056 | -0.8053 | -0.8047 | -0.8036 |
| 0.0185 | -0.8127 | -0.8123 | -0.8116 | -0.8104 |
| 0.019 | -0.8194 | -0.819 | -0.8182 | -0.8169 |
| 0.0195 | -0.8258 | -0.8254 | -0.8245 | -0.8231 |
| 0.02 | -0.8319 | -0.8314 | -0.8305 | -0.8289 |
| 0.0205 | -0.8378 | -0.8372 | -0.8362 | -0.8345 |
| 0.021 | -0.8433 | -0.8427 | -0.8416 | -0.8399 |
| 0.0215 | -0.8486 | -0.848 | -0.8468 | -0.8449 |
| 0.022 | -0.8537 | -0.853 | -0.8517 | -0.8498 |
| 0.0225 | -0.8585 | -0.8577 | -0.8564 | -0.8544 |
| 0.023 | -0.8631 | -0.8623 | -0.8609 | -0.8588 |
| 0.0235 | -0.8675 | -0.8667 | -0.8652 | -0.863 |
| 0.024 | -0.8717 | -0.8708 | -0.8693 | -0.867 |
| 0.0245 | -0.8757 | -0.8748 | -0.8732 | -0.8708 |
| 0.025 | -0.8796 | -0.8786 | -0.8769 | -0.8744 |
| 0.0255 | -0.8832 | -0.8823 | -0.8805 | -0.8779 |
| 0.026 | -0.8868 | -0.8857 | -0.8839 | -0.8813 |
| 0.0265 | -0.8901 | -0.8891 | -0.8872 | -0.8845 |
| 0.027 | -0.8934 | -0.8923 | -0.8904 | -0.8875 |
| 0.0275 | -0.8965 | -0.8953 | -0.8934 | -0.8905 |
| 0.028 | -0.8995 | -0.8983 | -0.8963 | -0.8933 |
| 0.0285 | -0.9023 | -0.9011 | -0.899 | -0.8959 |
| 0.029 | -0.9051 | -0.9038 | -0.9017 | -0.8985 |
| 0.0295 | -0.9077 | -0.9064 | -0.9042 | -0.901 |
| 0.03 | -0.9102 | -0.9089 | -0.9067 | -0.9033 |
| 0.0305 | -0.9126 | -0.9113 | -0.909 | -0.9056 |
| 0.031 | -0.915 | -0.9136 | -0.9112 | -0.9078 |
| 0.0315 | -0.9172 | -0.9158 | -0.9134 | -0.9099 |
| 0.032 | -0.9194 | -0.918 | -0.9155 | -0.9119 |
| 0.0325 | -0.9215 | -0.92 | -0.9175 | -0.9138 |
| 0.033 | -0.9235 | -0.922 | -0.9194 | -0.9157 |
| 0.0335 | -0.9254 | -0.9239 | -0.9213 | -0.9174 |
| 0.034 | -0.9273 | -0.9257 | -0.923 | -0.9192 |
| 0.0345 | -0.9291 | -0.9275 | -0.9248 | -0.9208 |
| 0.035 | -0.9308 | -0.9292 | -0.9264 | -0.9224 |
| 0.0355 | -0.9325 | -0.9308 | -0.928 | -0.9239 |
| 0.036 | -0.9341 | -0.9324 | -0.9295 | -0.9254 |
| 0.0365 | -0.9356 | -0.9339 | -0.931 | -0.9268 |
| 0.037 | -0.9371 | -0.9354 | -0.9324 | -0.9281 |
| 0.0375 | -0.9386 | -0.9368 | -0.9338 | -0.9295 |
| 0.038 | -0.94 | -0.9382 | -0.9351 | -0.9307 |
| 0.0385 | -0.9414 | -0.9395 | -0.9364 | -0.9319 |
| 0.039 | -0.9427 | -0.9408 | -0.9376 | -0.9331 |
| 0.0395 | -0.9439 | -0.942 | -0.9388 | -0.9342 |
| 0.04 | -0.9452 | -0.9432 | -0.94 | -0.9353 |
| 0.0405 | -0.9463 | -0.9444 | -0.9411 | -0.9364 |
| 0.041 | -0.9475 | -0.9455 | -0.9422 | -0.9374 |
| 0.0415 | -0.9486 | -0.9466 | -0.9432 | -0.9383 |
| 0.042 | -0.9497 | -0.9477 | -0.9442 | -0.9393 |
| 0.0425 | -0.9507 | -0.9487 | -0.9452 | -0.9402 |
| 0.043 | -0.9517 | -0.9497 | -0.9461 | -0.9411 |
| 0.0435 | -0.9527 | -0.9506 | -0.947 | -0.9419 |
| 0.044 | -0.9537 | -0.9515 | -0.9479 | -0.9427 |
| 0.0445 | -0.9546 | -0.9524 | -0.9488 | -0.9435 |
| 0.045 | -0.9555 | -0.9533 | -0.9496 | -0.9443 |
| 0.0455 | -0.9564 | -0.9541 | -0.9504 | -0.945 |
| 0.046 | -0.9572 | -0.955 | -0.9512 | -0.9457 |
| 0.0465 | -0.958 | -0.9558 | -0.9519 | -0.9464 |
| 0.047 | -0.9588 | -0.9565 | -0.9526 | -0.9471 |
| 0.0475 | -0.9596 | -0.9573 | -0.9533 | -0.9477 |
| 0.048 | -0.9604 | -0.958 | -0.954 | -0.9483 |
| 0.0485 | -0.9611 | -0.9587 | -0.9547 | -0.9489 |
| 0.049 | -0.9618 | -0.9594 | -0.9553 | -0.9495 |
| 0.0495 | -0.9625 | -0.96 | -0.9559 | -0.9501 |
| 0.05 | -0.9632 | -0.9607 | -0.9565 | -0.9506 |
| 0.0505 | -0.9638 | -0.9613 | -0.9571 | -0.9511 |
| 0.051 | -0.9644 | -0.9619 | -0.9577 | -0.9516 |
| 0.0515 | -0.9651 | -0.9625 | -0.9582 | -0.9521 |
| 0.052 | -0.9657 | -0.9631 | -0.9588 | -0.9526 |
| 0.0525 | -0.9662 | -0.9637 | -0.9593 | -0.9531 |
| 0.053 | -0.9668 | -0.9642 | -0.9598 | -0.9535 |
| 0.0535 | -0.9674 | -0.9647 | -0.9603 | -0.9539 |
| 0.054 | -0.9679 | -0.9652 | -0.9607 | -0.9543 |
| 0.0545 | -0.9684 | -0.9657 | -0.9612 | -0.9547 |
| 0.055 | -0.969 | -0.9662 | -0.9616 | -0.9551 |
| 0.0555 | -0.9695 | -0.9667 | -0.9621 | -0.9555 |
| 0.056 | -0.9699 | -0.9672 | -0.9625 | -0.9558 |
| 0.0565 | -0.9704 | -0.9676 | -0.9629 | -0.9562 |
| 0.057 | -0.9709 | -0.9681 | -0.9633 | -0.9565 |
| 0.0575 | -0.9713 | -0.9685 | -0.9637 | -0.9569 |
| 0.058 | -0.9718 | -0.9689 | -0.964 | -0.9572 |
| 0.0585 | -0.9722 | -0.9693 | -0.9644 | -0.9575 |
| 0.059 | -0.9726 | -0.9697 | -0.9648 | -0.9578 |
| 0.0595 | -0.973 | -0.9701 | -0.9651 | -0.958 |
| 0.06 | -0.9734 | -0.9705 | -0.9654 | -0.9583 |
| 0.0605 | -0.9738 | -0.9708 | -0.9658 | -0.9586 |
| 0.061 | -0.9742 | -0.9712 | -0.9661 | -0.9588 |
| 0.0615 | -0.9746 | -0.9715 | -0.9664 | -0.9591 |
| 0.062 | -0.975 | -0.9719 | -0.9667 | -0.9593 |
| 0.0625 | -0.9753 | -0.9722 | -0.967 | -0.9596 |
| 0.063 | -0.9757 | -0.9725 | -0.9672 | -0.9598 |
| 0.0635 | -0.976 | -0.9728 | -0.9675 | -0.96 |
| 0.064 | -0.9763 | -0.9731 | -0.9678 | -0.9602 |
| 0.0645 | -0.9767 | -0.9734 | -0.968 | -0.9604 |
| 0.065 | -0.977 | -0.9737 | -0.9683 | -0.9606 |
| 0.0655 | -0.9773 | -0.974 | -0.9685 | -0.9608 |
| 0.066 | -0.9776 | -0.9743 | -0.9688 | -0.9609 |
| 0.0665 | -0.9779 | -0.9746 | -0.969 | -0.9611 |
| 0.067 | -0.9782 | -0.9748 | -0.9692 | -0.9613 |
| 0.0675 | -0.9785 | -0.9751 | -0.9694 | -0.9614 |
| 0.068 | -0.9788 | -0.9754 | -0.9697 | -0.9616 |
| 0.0685 | -0.979 | -0.9756 | -0.9699 | -0.9617 |
| 0.069 | -0.9793 | -0.9758 | -0.9701 | -0.9619 |
| 0.0695 | -0.9796 | -0.9761 | -0.9702 | -0.962 |
| 0.07 | -0.9798 | -0.9763 | -0.9704 | -0.9621 |
| 0.0705 | -0.9801 | -0.9765 | -0.9706 | -0.9623 |
| 0.071 | -0.9803 | -0.9768 | -0.9708 | -0.9624 |
| 0.0715 | -0.9805 | -0.977 | -0.971 | -0.9625 |
| 0.072 | -0.9808 | -0.9772 | -0.9711 | -0.9626 |
| 0.0725 | -0.981 | -0.9774 | -0.9713 | -0.9627 |
| 0.073 | -0.9812 | -0.9776 | -0.9715 | -0.9628 |
| 0.0735 | -0.9815 | -0.9778 | -0.9716 | -0.9629 |
| 0.074 | -0.9817 | -0.978 | -0.9718 | -0.963 |
| 0.0745 | -0.9819 | -0.9782 | -0.9719 | -0.9631 |
| 0.075 | -0.9821 | -0.9783 | -0.972 | -0.9632 |
| 0.0755 | -0.9823 | -0.9785 | -0.9722 | -0.9632 |
| 0.076 | -0.9825 | -0.9787 | -0.9723 | -0.9633 |
| 0.0765 | -0.9827 | -0.9789 | -0.9724 | -0.9634 |
| 0.077 | -0.9829 | -0.979 | -0.9726 | -0.9635 |
| 0.0775 | -0.9831 | -0.9792 | -0.9727 | -0.9635 |
| 0.078 | -0.9833 | -0.9794 | -0.9728 | -0.9636 |
| 0.0785 | -0.9834 | -0.9795 | -0.9729 | -0.9636 |
| 0.079 | -0.9836 | -0.9797 | -0.973 | -0.9637 |
| 0.0795 | -0.9838 | -0.9798 | -0.9731 | -0.9637 |
| 0.08 | -0.984 | -0.98 | -0.9733 | -0.9638 |
| 0.0805 | -0.9841 | -0.9801 | -0.9734 | -0.9638 |
| 0.081 | -0.9843 | -0.9802 | -0.9734 | -0.9639 |
| 0.0815 | -0.9845 | -0.9804 | -0.9735 | -0.9639 |
| 0.082 | -0.9846 | -0.9805 | -0.9736 | -0.9639 |
| 0.0825 | -0.9848 | -0.9806 | -0.9737 | -0.964 |
| 0.083 | -0.9849 | -0.9808 | -0.9738 | -0.964 |
| 0.0835 | -0.9851 | -0.9809 | -0.9739 | -0.964 |
| 0.084 | -0.9852 | -0.981 | -0.974 | -0.964 |
| 0.0845 | -0.9854 | -0.9811 | -0.9741 | -0.9641 |
| 0.085 | -0.9855 | -0.9813 | -0.9741 | -0.9641 |
| 0.0855 | -0.9857 | -0.9814 | -0.9742 | -0.9641 |
| 0.086 | -0.9858 | -0.9815 | -0.9743 | -0.9641 |
| 0.0865 | -0.9859 | -0.9816 | -0.9743 | -0.9641 |
| 0.087 | -0.9861 | -0.9817 | -0.9744 | -0.9641 |
| 0.0875 | -0.9862 | -0.9818 | -0.9745 | -0.9641 |
| 0.088 | -0.9863 | -0.9819 | -0.9745 | -0.9641 |
| 0.0885 | -0.9865 | -0.982 | -0.9746 | -0.9641 |
| 0.089 | -0.9866 | -0.9821 | -0.9746 | -0.9641 |
| 0.0895 | -0.9867 | -0.9822 | -0.9747 | -0.9641 |
| 0.09 | -0.9868 | -0.9823 | -0.9748 | -0.9641 |
| 0.0905 | -0.9869 | -0.9824 | -0.9748 | -0.9641 |
| 0.091 | -0.9871 | -0.9825 | -0.9749 | -0.9641 |
| 0.0915 | -0.9872 | -0.9826 | -0.9749 | -0.9641 |
| 0.092 | -0.9873 | -0.9827 | -0.9749 | -0.9641 |
| 0.0925 | -0.9874 | -0.9827 | -0.975 | -0.9641 |
| 0.093 | -0.9875 | -0.9828 | -0.975 | -0.964 |
| 0.0935 | -0.9876 | -0.9829 | -0.9751 | -0.964 |
| 0.094 | -0.9877 | -0.983 | -0.9751 | -0.964 |
| 0.0945 | -0.9878 | -0.9831 | -0.9751 | -0.964 |
| 0.095 | -0.9879 | -0.9831 | -0.9752 | -0.9639 |
| 0.0955 | -0.988 | -0.9832 | -0.9752 | -0.9639 |
| 0.096 | -0.9881 | -0.9833 | -0.9752 | -0.9639 |
| 0.0965 | -0.9882 | -0.9834 | -0.9753 | -0.9639 |
| 0.097 | -0.9883 | -0.9834 | -0.9753 | -0.9638 |
| 0.0975 | -0.9884 | -0.9835 | -0.9753 | -0.9638 |
| 0.098 | -0.9885 | -0.9836 | -0.9753 | -0.9638 |
| 0.0985 | -0.9886 | -0.9836 | -0.9754 | -0.9637 |
| 0.099 | -0.9887 | -0.9837 | -0.9754 | -0.9637 |
| 0.0995 | -0.9887 | -0.9838 | -0.9754 | -0.9637 |
| 0.1 | -0.9888 | -0.9838 | -0.9754 | -0.9636 |
