# Supplementary material for: Analysis of sinusoidal post-buckling deformation of horizontal coiled tubing with initial residual bending
Source: PLoS One. 2024 May 14;19(5):e0301610. doi: 10.1371/journal.pone.0301610 (PMC11093391; doi:10.1371/journal.pone.0301610)
Supplement: S1 File — (ZIP) [file pone.0301610.s001.zip › The values used to build graphs - Fig 7 (a).docx]

## The values used to build graphs

The minimal data set of the original data for plotting curves in Fig 7 (a) is as follows:

| x-axis | ‾*L*_0_ = 100 | ‾*L*_0_ = 200 | ‾*L*_0_ = 500 | ‾*L*_0_ = 1000 |
| --- | --- | --- | --- | --- |
| 0 | -0.0015 | -0.00037 | -5.9E-05 | -1.5E-05 |
| 0.0005 | -0.0509 | -0.0254 | -0.0102 | -0.0051 |
| 0.001 | -0.0978 | -0.0498 | -0.0202 | -0.0101 |
| 0.0015 | -0.1424 | -0.0736 | -0.0301 | -0.0151 |
| 0.002 | -0.1846 | -0.0968 | -0.0398 | -0.0201 |
| 0.0025 | -0.2247 | -0.1194 | -0.0495 | -0.0251 |
| 0.003 | -0.2627 | -0.1414 | -0.0591 | -0.03 |
| 0.0035 | -0.2987 | -0.1629 | -0.0686 | -0.0349 |
| 0.004 | -0.3328 | -0.1838 | -0.078 | -0.0398 |
| 0.0045 | -0.3651 | -0.2041 | -0.0873 | -0.0447 |
| 0.005 | -0.3957 | -0.2239 | -0.0966 | -0.0495 |
| 0.0055 | -0.4246 | -0.2432 | -0.1057 | -0.0543 |
| 0.006 | -0.4519 | -0.262 | -0.1147 | -0.0591 |
| 0.0065 | -0.4778 | -0.2802 | -0.1236 | -0.0639 |
| 0.007 | -0.5023 | -0.298 | -0.1324 | -0.0686 |
| 0.0075 | -0.5254 | -0.3153 | -0.1412 | -0.0733 |
| 0.008 | -0.5473 | -0.3321 | -0.1498 | -0.078 |
| 0.0085 | -0.568 | -0.3485 | -0.1584 | -0.0827 |
| 0.009 | -0.5875 | -0.3645 | -0.1669 | -0.0873 |
| 0.0095 | -0.606 | -0.38 | -0.1752 | -0.0919 |
| 0.01 | -0.6235 | -0.3951 | -0.1835 | -0.0965 |
| 0.0105 | -0.6401 | -0.4098 | -0.1917 | -0.1011 |
| 0.011 | -0.6558 | -0.424 | -0.1998 | -0.1056 |
| 0.0115 | -0.6706 | -0.4379 | -0.2079 | -0.1101 |
| 0.012 | -0.6846 | -0.4514 | -0.2158 | -0.1146 |
| 0.0125 | -0.6979 | -0.4646 | -0.2237 | -0.1191 |
| 0.013 | -0.7105 | -0.4773 | -0.2315 | -0.1236 |
| 0.0135 | -0.7224 | -0.4897 | -0.2391 | -0.128 |
| 0.014 | -0.7337 | -0.5018 | -0.2468 | -0.1324 |
| 0.0145 | -0.7444 | -0.5136 | -0.2543 | -0.1368 |
| 0.015 | -0.7546 | -0.525 | -0.2617 | -0.1411 |
| 0.0155 | -0.7642 | -0.5361 | -0.2691 | -0.1455 |
| 0.016 | -0.7734 | -0.5469 | -0.2764 | -0.1498 |
| 0.0165 | -0.7821 | -0.5574 | -0.2836 | -0.1541 |
| 0.017 | -0.7903 | -0.5676 | -0.2907 | -0.1583 |
| 0.0175 | -0.7981 | -0.5776 | -0.2978 | -0.1626 |
| 0.018 | -0.8056 | -0.5872 | -0.3048 | -0.1668 |
| 0.0185 | -0.8127 | -0.5966 | -0.3117 | -0.171 |
| 0.019 | -0.8194 | -0.6058 | -0.3185 | -0.1752 |
| 0.0195 | -0.8258 | -0.6147 | -0.3253 | -0.1794 |
| 0.02 | -0.8319 | -0.6233 | -0.332 | -0.1835 |
| 0.0205 | -0.8378 | -0.6317 | -0.3386 | -0.1876 |
| 0.021 | -0.8433 | -0.6399 | -0.3451 | -0.1917 |
| 0.0215 | -0.8486 | -0.6478 | -0.3516 | -0.1958 |
| 0.022 | -0.8537 | -0.6556 | -0.358 | -0.1998 |
| 0.0225 | -0.8585 | -0.6631 | -0.3643 | -0.2038 |
| 0.023 | -0.8631 | -0.6704 | -0.3706 | -0.2078 |
| 0.0235 | -0.8675 | -0.6775 | -0.3767 | -0.2118 |
| 0.024 | -0.8717 | -0.6845 | -0.3829 | -0.2158 |
| 0.0245 | -0.8757 | -0.6912 | -0.3889 | -0.2197 |
| 0.025 | -0.8796 | -0.6978 | -0.3949 | -0.2236 |
| 0.0255 | -0.8832 | -0.7042 | -0.4008 | -0.2275 |
| 0.026 | -0.8868 | -0.7104 | -0.4067 | -0.2314 |
| 0.0265 | -0.8901 | -0.7164 | -0.4125 | -0.2353 |
| 0.027 | -0.8934 | -0.7223 | -0.4182 | -0.2391 |
| 0.0275 | -0.8965 | -0.7281 | -0.4239 | -0.2429 |
| 0.028 | -0.8995 | -0.7337 | -0.4295 | -0.2467 |
| 0.0285 | -0.9023 | -0.7391 | -0.435 | -0.2505 |
| 0.029 | -0.9051 | -0.7444 | -0.4405 | -0.2543 |
| 0.0295 | -0.9077 | -0.7495 | -0.4459 | -0.258 |
| 0.03 | -0.9102 | -0.7546 | -0.4513 | -0.2617 |
| 0.0305 | -0.9126 | -0.7594 | -0.4566 | -0.2654 |
| 0.031 | -0.915 | -0.7642 | -0.4618 | -0.2691 |
| 0.0315 | -0.9172 | -0.7689 | -0.467 | -0.2727 |
| 0.032 | -0.9194 | -0.7734 | -0.4721 | -0.2764 |
| 0.0325 | -0.9215 | -0.7778 | -0.4772 | -0.28 |
| 0.033 | -0.9235 | -0.7821 | -0.4822 | -0.2836 |
| 0.0335 | -0.9254 | -0.7863 | -0.4872 | -0.2872 |
| 0.034 | -0.9273 | -0.7903 | -0.4921 | -0.2907 |
| 0.0345 | -0.9291 | -0.7943 | -0.4969 | -0.2943 |
| 0.035 | -0.9308 | -0.7982 | -0.5017 | -0.2978 |
| 0.0355 | -0.9325 | -0.802 | -0.5064 | -0.3013 |
| 0.036 | -0.9341 | -0.8057 | -0.5111 | -0.3048 |
| 0.0365 | -0.9356 | -0.8093 | -0.5158 | -0.3082 |
| 0.037 | -0.9371 | -0.8128 | -0.5204 | -0.3117 |
| 0.0375 | -0.9386 | -0.8162 | -0.5249 | -0.3151 |
| 0.038 | -0.94 | -0.8195 | -0.5294 | -0.3185 |
| 0.0385 | -0.9414 | -0.8228 | -0.5338 | -0.3219 |
| 0.039 | -0.9427 | -0.8259 | -0.5382 | -0.3253 |
| 0.0395 | -0.9439 | -0.829 | -0.5425 | -0.3286 |
| 0.04 | -0.9452 | -0.8321 | -0.5468 | -0.3319 |
| 0.0405 | -0.9463 | -0.835 | -0.5511 | -0.3352 |
| 0.041 | -0.9475 | -0.8379 | -0.5552 | -0.3385 |
| 0.0415 | -0.9486 | -0.8407 | -0.5594 | -0.3418 |
| 0.042 | -0.9497 | -0.8435 | -0.5635 | -0.3451 |
| 0.0425 | -0.9507 | -0.8462 | -0.5675 | -0.3483 |
| 0.043 | -0.9517 | -0.8488 | -0.5716 | -0.3516 |
| 0.0435 | -0.9527 | -0.8513 | -0.5755 | -0.3548 |
| 0.044 | -0.9537 | -0.8538 | -0.5794 | -0.3579 |
| 0.0445 | -0.9546 | -0.8563 | -0.5833 | -0.3611 |
| 0.045 | -0.9555 | -0.8587 | -0.5871 | -0.3643 |
| 0.0455 | -0.9564 | -0.861 | -0.5909 | -0.3674 |
| 0.046 | -0.9572 | -0.8633 | -0.5947 | -0.3705 |
| 0.0465 | -0.958 | -0.8655 | -0.5984 | -0.3736 |
| 0.047 | -0.9588 | -0.8677 | -0.6021 | -0.3767 |
| 0.0475 | -0.9596 | -0.8698 | -0.6057 | -0.3798 |
| 0.048 | -0.9604 | -0.8719 | -0.6093 | -0.3828 |
| 0.0485 | -0.9611 | -0.8739 | -0.6128 | -0.3859 |
| 0.049 | -0.9618 | -0.8759 | -0.6163 | -0.3889 |
| 0.0495 | -0.9625 | -0.8779 | -0.6198 | -0.3919 |
| 0.05 | -0.9632 | -0.8798 | -0.6232 | -0.3949 |
| 0.0505 | -0.9638 | -0.8817 | -0.6266 | -0.3979 |
| 0.051 | -0.9644 | -0.8835 | -0.63 | -0.4008 |
| 0.0515 | -0.9651 | -0.8853 | -0.6333 | -0.4037 |
| 0.052 | -0.9657 | -0.887 | -0.6366 | -0.4067 |
| 0.0525 | -0.9662 | -0.8887 | -0.6398 | -0.4096 |
| 0.053 | -0.9668 | -0.8904 | -0.643 | -0.4125 |
| 0.0535 | -0.9674 | -0.892 | -0.6462 | -0.4153 |
| 0.054 | -0.9679 | -0.8937 | -0.6493 | -0.4182 |
| 0.0545 | -0.9684 | -0.8952 | -0.6524 | -0.421 |
| 0.055 | -0.969 | -0.8968 | -0.6555 | -0.4239 |
| 0.0555 | -0.9695 | -0.8983 | -0.6586 | -0.4267 |
| 0.056 | -0.9699 | -0.8997 | -0.6616 | -0.4295 |
| 0.0565 | -0.9704 | -0.9012 | -0.6645 | -0.4322 |
| 0.057 | -0.9709 | -0.9026 | -0.6675 | -0.435 |
| 0.0575 | -0.9713 | -0.904 | -0.6704 | -0.4377 |
| 0.058 | -0.9718 | -0.9054 | -0.6733 | -0.4405 |
| 0.0585 | -0.9722 | -0.9067 | -0.6761 | -0.4432 |
| 0.059 | -0.9726 | -0.908 | -0.6789 | -0.4459 |
| 0.0595 | -0.973 | -0.9093 | -0.6817 | -0.4486 |
| 0.06 | -0.9734 | -0.9105 | -0.6844 | -0.4513 |
| 0.0605 | -0.9738 | -0.9118 | -0.6872 | -0.4539 |
| 0.061 | -0.9742 | -0.913 | -0.6899 | -0.4566 |
| 0.0615 | -0.9746 | -0.9142 | -0.6925 | -0.4592 |
| 0.062 | -0.975 | -0.9153 | -0.6951 | -0.4618 |
| 0.0625 | -0.9753 | -0.9165 | -0.6978 | -0.4644 |
| 0.063 | -0.9757 | -0.9176 | -0.7003 | -0.467 |
| 0.0635 | -0.976 | -0.9187 | -0.7029 | -0.4696 |
| 0.064 | -0.9763 | -0.9198 | -0.7054 | -0.4721 |
| 0.0645 | -0.9767 | -0.9208 | -0.7079 | -0.4746 |
| 0.065 | -0.977 | -0.9218 | -0.7104 | -0.4772 |
| 0.0655 | -0.9773 | -0.9229 | -0.7128 | -0.4797 |
| 0.066 | -0.9776 | -0.9239 | -0.7152 | -0.4822 |
| 0.0665 | -0.9779 | -0.9248 | -0.7176 | -0.4847 |
| 0.067 | -0.9782 | -0.9258 | -0.72 | -0.4871 |
| 0.0675 | -0.9785 | -0.9267 | -0.7223 | -0.4896 |
| 0.068 | -0.9788 | -0.9277 | -0.7246 | -0.492 |
| 0.0685 | -0.979 | -0.9286 | -0.7269 | -0.4945 |
| 0.069 | -0.9793 | -0.9295 | -0.7292 | -0.4969 |
| 0.0695 | -0.9796 | -0.9303 | -0.7314 | -0.4993 |
| 0.07 | -0.9798 | -0.9312 | -0.7336 | -0.5017 |
| 0.0705 | -0.9801 | -0.9321 | -0.7358 | -0.5041 |
| 0.071 | -0.9803 | -0.9329 | -0.738 | -0.5064 |
| 0.0715 | -0.9805 | -0.9337 | -0.7401 | -0.5088 |
| 0.072 | -0.9808 | -0.9345 | -0.7423 | -0.5111 |
| 0.0725 | -0.981 | -0.9353 | -0.7444 | -0.5134 |
| 0.073 | -0.9812 | -0.9361 | -0.7465 | -0.5158 |
| 0.0735 | -0.9815 | -0.9368 | -0.7485 | -0.5181 |
| 0.074 | -0.9817 | -0.9376 | -0.7505 | -0.5203 |
| 0.0745 | -0.9819 | -0.9383 | -0.7526 | -0.5226 |
| 0.075 | -0.9821 | -0.939 | -0.7546 | -0.5249 |
| 0.0755 | -0.9823 | -0.9397 | -0.7565 | -0.5271 |
| 0.076 | -0.9825 | -0.9404 | -0.7585 | -0.5294 |
| 0.0765 | -0.9827 | -0.9411 | -0.7604 | -0.5316 |
| 0.077 | -0.9829 | -0.9418 | -0.7623 | -0.5338 |
| 0.0775 | -0.9831 | -0.9425 | -0.7642 | -0.536 |
| 0.078 | -0.9833 | -0.9431 | -0.7661 | -0.5382 |
| 0.0785 | -0.9834 | -0.9438 | -0.7679 | -0.5403 |
| 0.079 | -0.9836 | -0.9444 | -0.7698 | -0.5425 |
| 0.0795 | -0.9838 | -0.945 | -0.7716 | -0.5447 |
| 0.08 | -0.984 | -0.9456 | -0.7734 | -0.5468 |
| 0.0805 | -0.9841 | -0.9462 | -0.7752 | -0.5489 |
| 0.081 | -0.9843 | -0.9468 | -0.7769 | -0.551 |
| 0.0815 | -0.9845 | -0.9474 | -0.7787 | -0.5531 |
| 0.082 | -0.9846 | -0.948 | -0.7804 | -0.5552 |
| 0.0825 | -0.9848 | -0.9486 | -0.7821 | -0.5573 |
| 0.083 | -0.9849 | -0.9491 | -0.7838 | -0.5594 |
| 0.0835 | -0.9851 | -0.9497 | -0.7854 | -0.5614 |
| 0.084 | -0.9852 | -0.9502 | -0.7871 | -0.5635 |
| 0.0845 | -0.9854 | -0.9507 | -0.7887 | -0.5655 |
| 0.085 | -0.9855 | -0.9512 | -0.7903 | -0.5675 |
| 0.0855 | -0.9857 | -0.9518 | -0.792 | -0.5695 |
| 0.086 | -0.9858 | -0.9523 | -0.7935 | -0.5715 |
| 0.0865 | -0.9859 | -0.9528 | -0.7951 | -0.5735 |
| 0.087 | -0.9861 | -0.9533 | -0.7967 | -0.5755 |
| 0.0875 | -0.9862 | -0.9537 | -0.7982 | -0.5775 |
| 0.088 | -0.9863 | -0.9542 | -0.7997 | -0.5794 |
| 0.0885 | -0.9865 | -0.9547 | -0.8012 | -0.5814 |
| 0.089 | -0.9866 | -0.9551 | -0.8027 | -0.5833 |
| 0.0895 | -0.9867 | -0.9556 | -0.8042 | -0.5852 |
| 0.09 | -0.9868 | -0.956 | -0.8057 | -0.5871 |
| 0.0905 | -0.9869 | -0.9565 | -0.8071 | -0.589 |
| 0.091 | -0.9871 | -0.9569 | -0.8086 | -0.5909 |
| 0.0915 | -0.9872 | -0.9574 | -0.81 | -0.5928 |
| 0.092 | -0.9873 | -0.9578 | -0.8114 | -0.5947 |
| 0.0925 | -0.9874 | -0.9582 | -0.8128 | -0.5965 |
| 0.093 | -0.9875 | -0.9586 | -0.8142 | -0.5984 |
| 0.0935 | -0.9876 | -0.959 | -0.8155 | -0.6002 |
| 0.094 | -0.9877 | -0.9594 | -0.8169 | -0.6021 |
| 0.0945 | -0.9878 | -0.9598 | -0.8182 | -0.6039 |
| 0.095 | -0.9879 | -0.9602 | -0.8195 | -0.6057 |
| 0.0955 | -0.988 | -0.9606 | -0.8209 | -0.6075 |
| 0.096 | -0.9881 | -0.9609 | -0.8222 | -0.6093 |
| 0.0965 | -0.9882 | -0.9613 | -0.8234 | -0.611 |
| 0.097 | -0.9883 | -0.9617 | -0.8247 | -0.6128 |
| 0.0975 | -0.9884 | -0.962 | -0.826 | -0.6146 |
| 0.098 | -0.9885 | -0.9624 | -0.8272 | -0.6163 |
| 0.0985 | -0.9886 | -0.9627 | -0.8285 | -0.6181 |
| 0.099 | -0.9887 | -0.9631 | -0.8297 | -0.6198 |
| 0.0995 | -0.9887 | -0.9634 | -0.8309 | -0.6215 |
| 0.1 | -0.9888 | -0.9638 | -0.8321 | -0.6232 |
