# Supplementary material for: Analysis of sinusoidal post-buckling deformation of horizontal coiled tubing with initial residual bending
Source: PLoS One. 2024 May 14;19(5):e0301610. doi: 10.1371/journal.pone.0301610 (PMC11093391; doi:10.1371/journal.pone.0301610)
Supplement: S1 File — (ZIP) [file pone.0301610.s001.zip › The values used to build graphs - Fig 6.docx]

## The values used to build graphs

The minimal data set of the original data for plotting curves in Fig 6 is as follows:

| x-axis | Wu (1995) | ‾*L*_0_ = 0.2 | ‾*L*_0_ = 0.3 | ‾*L*_0_ = 0.4 |
| --- | --- | --- | --- | --- |
| 0 | 1.0752 | 1.01854 | 1.00822 | 1.00462 |
| 0.001 | 1.12997 | 1.0446 | 1.02539 | 1.01743 |
| 0.002 | 1.18715 | 1.0713 | 1.04284 | 1.03041 |
| 0.003 | 1.24669 | 1.09863 | 1.06058 | 1.04355 |
| 0.004 | 1.30851 | 1.12659 | 1.07861 | 1.05684 |
| 0.005 | 1.37252 | 1.15517 | 1.09691 | 1.0703 |
| 0.006 | 1.43861 | 1.18438 | 1.1155 | 1.08392 |
| 0.007 | 1.50669 | 1.21419 | 1.13437 | 1.09769 |
| 0.008 | 1.57663 | 1.24461 | 1.15351 | 1.11163 |
| 0.009 | 1.64833 | 1.27561 | 1.17294 | 1.12572 |
| 0.01 | 1.72167 | 1.30721 | 1.19263 | 1.13997 |
| 0.011 | 1.79653 | 1.33937 | 1.2126 | 1.15438 |
| 0.012 | 1.8728 | 1.37209 | 1.23284 | 1.16894 |
| 0.013 | 1.95037 | 1.40536 | 1.25335 | 1.18366 |
| 0.014 | 2.02915 | 1.43917 | 1.27412 | 1.19853 |
| 0.015 | 2.10903 | 1.4735 | 1.29515 | 1.21356 |
| 0.016 | 2.18992 | 1.50833 | 1.31644 | 1.22873 |
| 0.017 | 2.27174 | 1.54367 | 1.33798 | 1.24406 |
| 0.018 | 2.3544 | 1.57948 | 1.35978 | 1.25954 |
| 0.019 | 2.43784 | 1.61575 | 1.38182 | 1.27516 |
| 0.02 | 2.52197 | 1.65249 | 1.40411 | 1.29094 |
| 0.021 | 2.60675 | 1.68965 | 1.42663 | 1.30685 |
| 0.022 | 2.6921 | 1.72725 | 1.4494 | 1.32292 |
| 0.023 | 2.77798 | 1.76525 | 1.47239 | 1.33912 |
| 0.024 | 2.86433 | 1.80365 | 1.49561 | 1.35547 |
| 0.025 | 2.95111 | 1.84243 | 1.51906 | 1.37196 |
| 0.026 | 3.03828 | 1.88158 | 1.54273 | 1.38858 |
| 0.027 | 3.12579 | 1.92109 | 1.56661 | 1.40535 |
| 0.028 | 3.21362 | 1.96094 | 1.59071 | 1.42224 |
| 0.029 | 3.30172 | 2.00113 | 1.61502 | 1.43928 |
| 0.03 | 3.39007 | 2.04163 | 1.63952 | 1.45644 |
| 0.031 | 3.47864 | 2.08244 | 1.66423 | 1.47374 |
| 0.032 | 3.56741 | 2.12354 | 1.68913 | 1.49116 |
| 0.033 | 3.65635 | 2.16493 | 1.71423 | 1.50871 |
| 0.034 | 3.74544 | 2.20659 | 1.73951 | 1.52639 |
| 0.035 | 3.83465 | 2.24851 | 1.76497 | 1.54419 |
| 0.036 | 3.92398 | 2.29069 | 1.79061 | 1.56211 |
| 0.037 | 4.01341 | 2.33311 | 1.81643 | 1.58015 |
| 0.038 | 4.10291 | 2.37576 | 1.84242 | 1.59831 |
| 0.039 | 4.19247 | 2.41863 | 1.86857 | 1.61659 |
| 0.04 | 4.28208 | 2.46173 | 1.89489 | 1.63498 |
| 0.041 | 4.37173 | 2.50503 | 1.92137 | 1.65348 |
| 0.042 | 4.46141 | 2.54852 | 1.948 | 1.6721 |
| 0.043 | 4.5511 | 2.59221 | 1.97478 | 1.69082 |
| 0.044 | 4.64079 | 2.63608 | 2.00172 | 1.70966 |
| 0.045 | 4.73048 | 2.68013 | 2.02879 | 1.7286 |
| 0.046 | 4.82016 | 2.72435 | 2.05601 | 1.74764 |
| 0.047 | 4.90981 | 2.76873 | 2.08337 | 1.76679 |
| 0.048 | 4.99943 | 2.81327 | 2.11085 | 1.78603 |
| 0.049 | 5.08902 | 2.85796 | 2.13847 | 1.80538 |
| 0.05 | 5.17856 | 2.90279 | 2.16622 | 1.82483 |
| 0.051 | 5.26806 | 2.94777 | 2.19409 | 1.84437 |
| 0.052 | 5.3575 | 2.99287 | 2.22208 | 1.864 |
| 0.053 | 5.44687 | 3.0381 | 2.25019 | 1.88373 |
| 0.054 | 5.53619 | 3.08346 | 2.27842 | 1.90355 |
| 0.055 | 5.62543 | 3.12894 | 2.30675 | 1.92345 |
| 0.056 | 5.71459 | 3.17453 | 2.3352 | 1.94345 |
| 0.057 | 5.80368 | 3.22023 | 2.36375 | 1.96353 |
| 0.058 | 5.89268 | 3.26603 | 2.39241 | 1.9837 |
| 0.059 | 5.98159 | 3.31194 | 2.42117 | 2.00395 |
| 0.06 | 6.07041 | 3.35795 | 2.45002 | 2.02428 |
| 0.061 | 6.15914 | 3.40404 | 2.47898 | 2.04469 |
| 0.062 | 6.24777 | 3.45023 | 2.50802 | 2.06518 |
| 0.063 | 6.3363 | 3.49651 | 2.53716 | 2.08574 |
| 0.064 | 6.42473 | 3.54287 | 2.56638 | 2.10639 |
| 0.065 | 6.51305 | 3.58931 | 2.5957 | 2.1271 |
| 0.066 | 6.60126 | 3.63583 | 2.62509 | 2.14789 |
| 0.067 | 6.68936 | 3.68242 | 2.65457 | 2.16876 |
| 0.068 | 6.77735 | 3.72909 | 2.68413 | 2.18969 |
| 0.069 | 6.86522 | 3.77582 | 2.71377 | 2.21069 |
| 0.07 | 6.95297 | 3.82262 | 2.74348 | 2.23176 |
| 0.071 | 7.0406 | 3.86948 | 2.77327 | 2.25289 |
| 0.072 | 7.12811 | 3.91641 | 2.80312 | 2.27409 |
| 0.073 | 7.2155 | 3.9634 | 2.83305 | 2.29535 |
| 0.074 | 7.30277 | 4.01044 | 2.86305 | 2.31668 |
| 0.075 | 7.3899 | 4.05754 | 2.89312 | 2.33807 |
| 0.076 | 7.47691 | 4.10469 | 2.92325 | 2.35952 |
| 0.077 | 7.56379 | 4.1519 | 2.95344 | 2.38102 |
| 0.078 | 7.65054 | 4.19915 | 2.9837 | 2.40259 |
| 0.079 | 7.73716 | 4.24645 | 3.01402 | 2.42421 |
| 0.08 | 7.82364 | 4.2938 | 3.04439 | 2.44589 |
| 0.081 | 7.90999 | 4.34119 | 3.07483 | 2.46762 |
| 0.082 | 7.9962 | 4.38863 | 3.10532 | 2.48941 |
| 0.083 | 8.08227 | 4.4361 | 3.13586 | 2.51125 |
| 0.084 | 8.16821 | 4.48362 | 3.16646 | 2.53314 |
| 0.085 | 8.25401 | 4.53117 | 3.19711 | 2.55508 |
| 0.086 | 8.33967 | 4.57876 | 3.22781 | 2.57707 |
| 0.087 | 8.42518 | 4.62639 | 3.25856 | 2.59911 |
| 0.088 | 8.51056 | 4.67405 | 3.28936 | 2.6212 |
| 0.089 | 8.59579 | 4.72174 | 3.3202 | 2.64333 |
| 0.09 | 8.68088 | 4.76947 | 3.3511 | 2.66552 |
| 0.091 | 8.76582 | 4.81722 | 3.38203 | 2.68774 |
| 0.092 | 8.85062 | 4.86501 | 3.41301 | 2.71001 |
| 0.093 | 8.93528 | 4.91282 | 3.44404 | 2.73233 |
| 0.094 | 9.01978 | 4.96066 | 3.4751 | 2.75468 |
| 0.095 | 9.10414 | 5.00853 | 3.50621 | 2.77708 |
| 0.096 | 9.18836 | 5.05642 | 3.53736 | 2.79952 |
| 0.097 | 9.27242 | 5.10434 | 3.56854 | 2.822 |
| 0.098 | 9.35633 | 5.15228 | 3.59977 | 2.84452 |
| 0.099 | 9.4401 | 5.20025 | 3.63103 | 2.86708 |
| 0.1 | 9.52371 | 5.24823 | 3.66232 | 2.88968 |
| 0.101 | 9.60718 | 5.29624 | 3.69366 | 2.91231 |
| 0.102 | 9.69049 | 5.34427 | 3.72502 | 2.93499 |
| 0.103 | 9.77365 | 5.39231 | 3.75642 | 2.95769 |
| 0.104 | 9.85666 | 5.44038 | 3.78786 | 2.98044 |
| 0.105 | 9.93952 | 5.48846 | 3.81933 | 3.00321 |
| 0.106 | 10.02223 | 5.53656 | 3.85082 | 3.02602 |
| 0.107 | 10.10478 | 5.58468 | 3.88235 | 3.04887 |
| 0.108 | 10.18717 | 5.63281 | 3.91391 | 3.07175 |
| 0.109 | 10.26942 | 5.68096 | 3.9455 | 3.09466 |
| 0.11 | 10.3515 | 5.72913 | 3.97712 | 3.1176 |
| 0.111 | 10.43344 | 5.7773 | 4.00876 | 3.14057 |
| 0.112 | 10.51521 | 5.8255 | 4.04044 | 3.16357 |
| 0.113 | 10.59684 | 5.8737 | 4.07214 | 3.18661 |
| 0.114 | 10.6783 | 5.92192 | 4.10386 | 3.20967 |
| 0.115 | 10.75961 | 5.97015 | 4.13562 | 3.23276 |
| 0.116 | 10.84076 | 6.01839 | 4.16739 | 3.25588 |
| 0.117 | 10.92176 | 6.06664 | 4.1992 | 3.27903 |
| 0.118 | 11.0026 | 6.1149 | 4.23102 | 3.3022 |
| 0.119 | 11.08328 | 6.16317 | 4.26287 | 3.32541 |
| 0.12 | 11.1638 | 6.21145 | 4.29474 | 3.34863 |
| 0.121 | 11.24417 | 6.25974 | 4.32664 | 3.37189 |
| 0.122 | 11.32437 | 6.30804 | 4.35856 | 3.39517 |
| 0.123 | 11.40442 | 6.35635 | 4.3905 | 3.41847 |
| 0.124 | 11.48431 | 6.40467 | 4.42246 | 3.4418 |
| 0.125 | 11.56404 | 6.45299 | 4.45444 | 3.46516 |
| 0.126 | 11.64361 | 6.50132 | 4.48644 | 3.48853 |
| 0.127 | 11.72302 | 6.54966 | 4.51846 | 3.51193 |
| 0.128 | 11.80227 | 6.598 | 4.5505 | 3.53536 |
| 0.129 | 11.88136 | 6.64635 | 4.58256 | 3.5588 |
| 0.13 | 11.96029 | 6.6947 | 4.61464 | 3.58227 |
| 0.131 | 12.03906 | 6.74306 | 4.64674 | 3.60576 |
| 0.132 | 12.11767 | 6.79143 | 4.67885 | 3.62927 |
| 0.133 | 12.19612 | 6.8398 | 4.71099 | 3.65281 |
| 0.134 | 12.27441 | 6.88817 | 4.74314 | 3.67636 |
| 0.135 | 12.35254 | 6.93655 | 4.7753 | 3.69993 |
| 0.136 | 12.4305 | 6.98494 | 4.80748 | 3.72353 |
| 0.137 | 12.50831 | 7.03332 | 4.83968 | 3.74714 |
| 0.138 | 12.58595 | 7.08171 | 4.8719 | 3.77077 |
| 0.139 | 12.66343 | 7.1301 | 4.90413 | 3.79443 |
| 0.14 | 12.74075 | 7.1785 | 4.93637 | 3.8181 |
| 0.141 | 12.8179 | 7.2269 | 4.96863 | 3.84178 |
| 0.142 | 12.8949 | 7.2753 | 5.00091 | 3.86549 |
| 0.143 | 12.97173 | 7.3237 | 5.0332 | 3.88921 |
| 0.144 | 13.0484 | 7.3721 | 5.0655 | 3.91296 |
| 0.145 | 13.1249 | 7.42051 | 5.09781 | 3.93672 |
| 0.146 | 13.20125 | 7.46891 | 5.13014 | 3.96049 |
| 0.147 | 13.27743 | 7.51732 | 5.16249 | 3.98428 |
| 0.148 | 13.35344 | 7.56573 | 5.19484 | 4.00809 |
| 0.149 | 13.4293 | 7.61414 | 5.22721 | 4.03192 |
| 0.15 | 13.50499 | 7.66255 | 5.25959 | 4.05576 |
