# Supplementary material for: Analysis of sinusoidal post-buckling deformation of horizontal coiled tubing with initial residual bending
Source: PLoS One. 2024 May 14;19(5):e0301610. doi: 10.1371/journal.pone.0301610 (PMC11093391; doi:10.1371/journal.pone.0301610)
Supplement: S1 File — (ZIP) [file pone.0301610.s001.zip › The values used to build graphs - Fig 5 (b).docx]

## The values used to build graphs

The minimal data set of the original data for plotting curves in Fig 5 (b) is as follows:

| x-axis | Wu (1995) | *A*_0_ = 0.2 | *A*_0_ = 0.35 | *A*_0_ = 0.5 |
| --- | --- | --- | --- | --- |
| 0 | 1 | 1.0752 | 1.23545 | 1.48042 |
| 0.001 | 1.05205 | 1.12997 | 1.29453 | 1.54143 |
| 0.002 | 1.10666 | 1.18715 | 1.35556 | 1.60338 |
| 0.003 | 1.16381 | 1.24669 | 1.41842 | 1.66615 |
| 0.004 | 1.22345 | 1.30851 | 1.483 | 1.7296 |
| 0.005 | 1.28552 | 1.37252 | 1.54917 | 1.79362 |
| 0.006 | 1.34993 | 1.43861 | 1.61681 | 1.85809 |
| 0.007 | 1.4166 | 1.50669 | 1.6858 | 1.92288 |
| 0.008 | 1.48543 | 1.57663 | 1.75602 | 1.98791 |
| 0.009 | 1.55631 | 1.64833 | 1.82734 | 2.05308 |
| 0.01 | 1.62912 | 1.72167 | 1.89967 | 2.11829 |
| 0.011 | 1.70377 | 1.79653 | 1.97288 | 2.18346 |
| 0.012 | 1.78014 | 1.8728 | 2.04687 | 2.24851 |
| 0.013 | 1.85811 | 1.95037 | 2.12156 | 2.31337 |
| 0.014 | 1.93758 | 2.02915 | 2.19685 | 2.37798 |
| 0.015 | 2.01845 | 2.10903 | 2.27266 | 2.44228 |
| 0.016 | 2.10062 | 2.18992 | 2.3489 | 2.50621 |
| 0.017 | 2.184 | 2.27174 | 2.42552 | 2.56972 |
| 0.018 | 2.2685 | 2.3544 | 2.50244 | 2.63277 |
| 0.019 | 2.35404 | 2.43784 | 2.57961 | 2.6953 |
| 0.02 | 2.44054 | 2.52197 | 2.65696 | 2.75729 |
| 0.021 | 2.52794 | 2.60675 | 2.73445 | 2.8187 |
| 0.022 | 2.61616 | 2.6921 | 2.81203 | 2.87949 |
| 0.023 | 2.70515 | 2.77798 | 2.88965 | 2.93963 |
| 0.024 | 2.79486 | 2.86433 | 2.96728 | 2.9991 |
| 0.025 | 2.88522 | 2.95111 | 3.04488 | 3.05787 |
| 0.026 | 2.9762 | 3.03828 | 3.12241 | 3.11591 |
| 0.027 | 3.06774 | 3.12579 | 3.19984 | 3.17321 |
| 0.028 | 3.15982 | 3.21362 | 3.27716 | 3.22975 |
| 0.029 | 3.25239 | 3.30172 | 3.35432 | 3.2855 |
| 0.03 | 3.34542 | 3.39007 | 3.4313 | 3.34045 |
| 0.031 | 3.43888 | 3.47864 | 3.50809 | 3.39459 |
| 0.032 | 3.53274 | 3.56741 | 3.58467 | 3.44789 |
| 0.033 | 3.62697 | 3.65635 | 3.661 | 3.50035 |
| 0.034 | 3.72155 | 3.74544 | 3.73709 | 3.55195 |
| 0.035 | 3.81646 | 3.83465 | 3.8129 | 3.60268 |
| 0.036 | 3.91167 | 3.92398 | 3.88843 | 3.65254 |
| 0.037 | 4.00717 | 4.01341 | 3.96366 | 3.7015 |
| 0.038 | 4.10294 | 4.10291 | 4.03858 | 3.74957 |
| 0.039 | 4.19897 | 4.19247 | 4.11318 | 3.79673 |
| 0.04 | 4.29523 | 4.28208 | 4.18744 | 3.84297 |
| 0.041 | 4.39171 | 4.37173 | 4.26136 | 3.88829 |
| 0.042 | 4.48841 | 4.46141 | 4.33493 | 3.93267 |
| 0.043 | 4.58531 | 4.5511 | 4.40813 | 3.97612 |
| 0.044 | 4.6824 | 4.64079 | 4.48096 | 4.01863 |
| 0.045 | 4.77966 | 4.73048 | 4.55341 | 4.06019 |
| 0.046 | 4.87709 | 4.82016 | 4.62548 | 4.10079 |
| 0.047 | 4.97469 | 4.90981 | 4.69715 | 4.14044 |
| 0.048 | 5.07243 | 4.99943 | 4.76842 | 4.17911 |
| 0.049 | 5.17031 | 5.08902 | 4.83929 | 4.21682 |
| 0.05 | 5.26834 | 5.17856 | 4.90975 | 4.25356 |
| 0.051 | 5.36649 | 5.26806 | 4.97979 | 4.28932 |
| 0.052 | 5.46476 | 5.3575 | 5.04941 | 4.32409 |
| 0.053 | 5.56315 | 5.44687 | 5.11861 | 4.35788 |
| 0.054 | 5.66165 | 5.53619 | 5.18737 | 4.39069 |
| 0.055 | 5.76026 | 5.62543 | 5.2557 | 4.4225 |
| 0.056 | 5.85896 | 5.71459 | 5.3236 | 4.45332 |
| 0.057 | 5.95777 | 5.80368 | 5.39105 | 4.48314 |
| 0.058 | 6.05666 | 5.89268 | 5.45806 | 4.51196 |
| 0.059 | 6.15565 | 5.98159 | 5.52461 | 4.53977 |
| 0.06 | 6.25472 | 6.07041 | 5.59072 | 4.56659 |
| 0.061 | 6.35386 | 6.15914 | 5.65638 | 4.59239 |
| 0.062 | 6.45309 | 6.24777 | 5.72157 | 4.61718 |
| 0.063 | 6.55239 | 6.3363 | 5.78631 | 4.64097 |
| 0.064 | 6.65176 | 6.42473 | 5.85059 | 4.66374 |
| 0.065 | 6.75119 | 6.51305 | 5.9144 | 4.68549 |
| 0.066 | 6.85069 | 6.60126 | 5.97774 | 4.70622 |
| 0.067 | 6.95026 | 6.68936 | 6.04062 | 4.72594 |
| 0.068 | 7.04988 | 6.77735 | 6.10303 | 4.74464 |
| 0.069 | 7.14956 | 6.86522 | 6.16496 | 4.76231 |
| 0.07 | 7.2493 | 6.95297 | 6.22642 | 4.77896 |
| 0.071 | 7.34909 | 7.0406 | 6.2874 | 4.79458 |
| 0.072 | 7.44893 | 7.12811 | 6.34791 | 4.80918 |
| 0.073 | 7.54882 | 7.2155 | 6.40794 | 4.82275 |
| 0.074 | 7.64876 | 7.30277 | 6.46748 | 4.83529 |
| 0.075 | 7.74874 | 7.3899 | 6.52655 | 4.8468 |
| 0.076 | 7.84877 | 7.47691 | 6.58513 | 4.85728 |
| 0.077 | 7.94884 | 7.56379 | 6.64323 | 4.86672 |
| 0.078 | 8.04895 | 7.65054 | 6.70084 | 4.87514 |
| 0.079 | 8.14911 | 7.73716 | 6.75796 | 4.88251 |
| 0.08 | 8.2493 | 7.82364 | 6.8146 | 4.88885 |
| 0.081 | 8.34953 | 7.90999 | 6.87074 | 4.89416 |
| 0.082 | 8.44979 | 7.9962 | 6.9264 | 4.89843 |
| 0.083 | 8.55009 | 8.08227 | 6.98156 | 4.90165 |
| 0.084 | 8.65042 | 8.16821 | 7.03624 | 4.90384 |
| 0.085 | 8.75079 | 8.25401 | 7.09042 | 4.90499 |
| 0.086 | 8.85118 | 8.33967 | 7.1441 | 4.9051 |
| 0.087 | 8.95161 | 8.42518 | 7.19729 | 4.90417 |
| 0.088 | 9.05207 | 8.51056 | 7.24999 | 4.90219 |
| 0.089 | 9.15256 | 8.59579 | 7.30218 | 4.89917 |
| 0.09 | 9.25307 | 8.68088 | 7.35388 | 4.89511 |
| 0.091 | 9.35361 | 8.76582 | 7.40509 | 4.89001 |
| 0.092 | 9.45418 | 8.85062 | 7.45579 | 4.88386 |
| 0.093 | 9.55478 | 8.93528 | 7.506 | 4.87666 |
| 0.094 | 9.6554 | 9.01978 | 7.5557 | 4.86842 |
| 0.095 | 9.75604 | 9.10414 | 7.60491 | 4.85913 |
| 0.096 | 9.85671 | 9.18836 | 7.65361 | 4.84879 |
| 0.097 | 9.9574 | 9.27242 | 7.70181 | 4.83741 |
| 0.098 | 10.05811 | 9.35633 | 7.74951 | 4.82497 |
| 0.099 | 10.15884 | 9.4401 | 7.79671 | 4.81149 |
| 0.1 | 10.2596 | 9.52371 | 7.8434 | 4.79696 |
| 0.101 | 10.36037 | 9.60718 | 7.88959 | 4.78138 |
| 0.102 | 10.46117 | 9.69049 | 7.93527 | 4.76475 |
| 0.103 | 10.56199 | 9.77365 | 7.98045 | 4.74707 |
| 0.104 | 10.66282 | 9.85666 | 8.02513 | 4.72834 |
| 0.105 | 10.76367 | 9.93952 | 8.0693 | 4.70856 |
| 0.106 | 10.86455 | 10.02223 | 8.11296 | 4.68772 |
| 0.107 | 10.96543 | 10.10478 | 8.15612 | 4.66584 |
| 0.108 | 11.06634 | 10.18717 | 8.19876 | 4.6429 |
| 0.109 | 11.16726 | 10.26942 | 8.24091 | 4.61891 |
| 0.11 | 11.26821 | 10.3515 | 8.28254 | 4.59386 |
| 0.111 | 11.36916 | 10.43344 | 8.32367 | 4.56776 |
| 0.112 | 11.47013 | 10.51521 | 8.36429 | 4.54061 |
| 0.113 | 11.57112 | 10.59684 | 8.4044 | 4.5124 |
| 0.114 | 11.67212 | 10.6783 | 8.444 | 4.48314 |
| 0.115 | 11.77314 | 10.75961 | 8.48309 | 4.45282 |
| 0.116 | 11.87417 | 10.84076 | 8.52167 | 4.42145 |
| 0.117 | 11.97522 | 10.92176 | 8.55974 | 4.38902 |
| 0.118 | 12.07628 | 11.0026 | 8.5973 | 4.35554 |
| 0.119 | 12.17735 | 11.08328 | 8.63435 | 4.321 |
| 0.12 | 12.27844 | 11.1638 | 8.67089 | 4.28541 |
| 0.121 | 12.37954 | 11.24417 | 8.70692 | 4.24875 |
| 0.122 | 12.48065 | 11.32437 | 8.74244 | 4.21104 |
| 0.123 | 12.58178 | 11.40442 | 8.77744 | 4.17228 |
| 0.124 | 12.68291 | 11.48431 | 8.81194 | 4.13245 |
| 0.125 | 12.78406 | 11.56404 | 8.84592 | 4.09157 |
| 0.126 | 12.88522 | 11.64361 | 8.87939 | 4.04963 |
| 0.127 | 12.9864 | 11.72302 | 8.91234 | 4.00664 |
| 0.128 | 13.08758 | 11.80227 | 8.94479 | 3.96258 |
| 0.129 | 13.18877 | 11.88136 | 8.97672 | 3.91747 |
| 0.13 | 13.28998 | 11.96029 | 9.00814 | 3.87129 |
| 0.131 | 13.3912 | 12.03906 | 9.03904 | 3.82406 |
| 0.132 | 13.49242 | 12.11767 | 9.06943 | 3.77577 |
| 0.133 | 13.59366 | 12.19612 | 9.09931 | 3.72642 |
| 0.134 | 13.69491 | 12.27441 | 9.12867 | 3.67601 |
| 0.135 | 13.79616 | 12.35254 | 9.15752 | 3.62454 |
| 0.136 | 13.89743 | 12.4305 | 9.18586 | 3.57201 |
| 0.137 | 13.99871 | 12.50831 | 9.21368 | 3.51842 |
| 0.138 | 14.09999 | 12.58595 | 9.24098 | 3.46377 |
| 0.139 | 14.20129 | 12.66343 | 9.26777 | 3.40805 |
| 0.14 | 14.3026 | 12.74075 | 9.29405 | 3.35128 |
| 0.141 | 14.40391 | 12.8179 | 9.31981 | 3.29345 |
| 0.142 | 14.50523 | 12.8949 | 9.34505 | 3.23455 |
| 0.143 | 14.60656 | 12.97173 | 9.36978 | 3.1746 |
| 0.144 | 14.7079 | 13.0484 | 9.39399 | 3.11358 |
| 0.145 | 14.80925 | 13.1249 | 9.41769 | 3.0515 |
| 0.146 | 14.91061 | 13.20125 | 9.44087 | 2.98836 |
| 0.147 | 15.01197 | 13.27743 | 9.46354 | 2.92415 |
| 0.148 | 15.11335 | 13.35344 | 9.48569 | 2.85889 |
| 0.149 | 15.21473 | 13.4293 | 9.50732 | 2.79256 |
| 0.15 | 15.31612 | 13.50499 | 9.52844 | 2.72517 |
