# Supplementary material for: Analysis of sinusoidal post-buckling deformation of horizontal coiled tubing with initial residual bending
Source: PLoS One. 2024 May 14;19(5):e0301610. doi: 10.1371/journal.pone.0301610 (PMC11093391; doi:10.1371/journal.pone.0301610)
Supplement: S1 File — (ZIP) [file pone.0301610.s001.zip › The values used to build graphs - Fig 5 (a).docx]

## The values used to build graphs

The minimal data set of the original data for plotting curves in Fig 5 (a) is as follows:

| x-axis | Wu (1995) | *A*_0_ = 0.2 | *A*_0_ = 0.3 | *A*_0_ = 0.4 |
| --- | --- | --- | --- | --- |
| 0 | 1 | 1.00012 | 1.00026 | 1.00047 |
| 0.001 | 1.05205 | 1.05217 | 1.05232 | 1.05253 |
| 0.002 | 1.10666 | 1.10679 | 1.10695 | 1.10717 |
| 0.003 | 1.16381 | 1.16394 | 1.16411 | 1.16433 |
| 0.004 | 1.22345 | 1.22359 | 1.22376 | 1.22399 |
| 0.005 | 1.28552 | 1.28566 | 1.28583 | 1.28607 |
| 0.006 | 1.34993 | 1.35007 | 1.35025 | 1.35049 |
| 0.007 | 1.4166 | 1.41675 | 1.41693 | 1.41718 |
| 0.008 | 1.48543 | 1.48558 | 1.48576 | 1.48601 |
| 0.009 | 1.55631 | 1.55645 | 1.55664 | 1.5569 |
| 0.01 | 1.62912 | 1.62927 | 1.62946 | 1.62972 |
| 0.011 | 1.70377 | 1.70392 | 1.70411 | 1.70437 |
| 0.012 | 1.78014 | 1.78029 | 1.78048 | 1.78074 |
| 0.013 | 1.85811 | 1.85826 | 1.85845 | 1.85871 |
| 0.014 | 1.93758 | 1.93773 | 1.93792 | 1.93818 |
| 0.015 | 2.01845 | 2.0186 | 2.01879 | 2.01905 |
| 0.016 | 2.10062 | 2.10077 | 2.10096 | 2.10121 |
| 0.017 | 2.184 | 2.18415 | 2.18433 | 2.18458 |
| 0.018 | 2.2685 | 2.26865 | 2.26882 | 2.26907 |
| 0.019 | 2.35404 | 2.35418 | 2.35436 | 2.3546 |
| 0.02 | 2.44054 | 2.44068 | 2.44085 | 2.44109 |
| 0.021 | 2.52794 | 2.52807 | 2.52824 | 2.52847 |
| 0.022 | 2.61616 | 2.61629 | 2.61645 | 2.61668 |
| 0.023 | 2.70515 | 2.70528 | 2.70543 | 2.70565 |
| 0.024 | 2.79486 | 2.79498 | 2.79513 | 2.79533 |
| 0.025 | 2.88522 | 2.88533 | 2.88548 | 2.88567 |
| 0.026 | 2.9762 | 2.97631 | 2.97644 | 2.97663 |
| 0.027 | 3.06774 | 3.06785 | 3.06797 | 3.06815 |
| 0.028 | 3.15982 | 3.15992 | 3.16004 | 3.1602 |
| 0.029 | 3.25239 | 3.25248 | 3.25259 | 3.25275 |
| 0.03 | 3.34542 | 3.3455 | 3.34561 | 3.34575 |
| 0.031 | 3.43888 | 3.43895 | 3.43905 | 3.43918 |
| 0.032 | 3.53274 | 3.5328 | 3.53289 | 3.533 |
| 0.033 | 3.62697 | 3.62703 | 3.6271 | 3.6272 |
| 0.034 | 3.72155 | 3.7216 | 3.72166 | 3.72175 |
| 0.035 | 3.81646 | 3.8165 | 3.81655 | 3.81662 |
| 0.036 | 3.91167 | 3.9117 | 3.91174 | 3.9118 |
| 0.037 | 4.00717 | 4.00719 | 4.00722 | 4.00726 |
| 0.038 | 4.10294 | 4.10296 | 4.10297 | 4.103 |
| 0.039 | 4.19897 | 4.19897 | 4.19898 | 4.19898 |
| 0.04 | 4.29523 | 4.29522 | 4.29521 | 4.2952 |
| 0.041 | 4.39171 | 4.3917 | 4.39168 | 4.39165 |
| 0.042 | 4.48841 | 4.48839 | 4.48835 | 4.4883 |
| 0.043 | 4.58531 | 4.58527 | 4.58522 | 4.58515 |
| 0.044 | 4.6824 | 4.68235 | 4.68229 | 4.6822 |
| 0.045 | 4.77966 | 4.7796 | 4.77952 | 4.77941 |
| 0.046 | 4.87709 | 4.87702 | 4.87693 | 4.8768 |
| 0.047 | 4.97469 | 4.9746 | 4.97449 | 4.97434 |
| 0.048 | 5.07243 | 5.07233 | 5.07221 | 5.07203 |
| 0.049 | 5.17031 | 5.1702 | 5.17006 | 5.16987 |
| 0.05 | 5.26834 | 5.26821 | 5.26806 | 5.26784 |
| 0.051 | 5.36649 | 5.36635 | 5.36618 | 5.36593 |
| 0.052 | 5.46476 | 5.46461 | 5.46442 | 5.46415 |
| 0.053 | 5.56315 | 5.56298 | 5.56278 | 5.56248 |
| 0.054 | 5.66165 | 5.66147 | 5.66125 | 5.66093 |
| 0.055 | 5.76026 | 5.76006 | 5.75982 | 5.75948 |
| 0.056 | 5.85896 | 5.85876 | 5.85849 | 5.85812 |
| 0.057 | 5.95777 | 5.95754 | 5.95726 | 5.95687 |
| 0.058 | 6.05666 | 6.05643 | 6.05613 | 6.0557 |
| 0.059 | 6.15565 | 6.15539 | 6.15507 | 6.15462 |
| 0.06 | 6.25472 | 6.25444 | 6.2541 | 6.25363 |
| 0.061 | 6.35386 | 6.35358 | 6.35322 | 6.35271 |
| 0.062 | 6.45309 | 6.45278 | 6.4524 | 6.45187 |
| 0.063 | 6.55239 | 6.55207 | 6.55166 | 6.5511 |
| 0.064 | 6.65176 | 6.65142 | 6.65099 | 6.6504 |
| 0.065 | 6.75119 | 6.75084 | 6.75039 | 6.74977 |
| 0.066 | 6.85069 | 6.85032 | 6.84985 | 6.8492 |
| 0.067 | 6.95026 | 6.94987 | 6.94938 | 6.94869 |
| 0.068 | 7.04988 | 7.04947 | 7.04896 | 7.04824 |
| 0.069 | 7.14956 | 7.14913 | 7.1486 | 7.14785 |
| 0.07 | 7.2493 | 7.24885 | 7.24829 | 7.24751 |
| 0.071 | 7.34909 | 7.34862 | 7.34804 | 7.34722 |
| 0.072 | 7.44893 | 7.44844 | 7.44784 | 7.44699 |
| 0.073 | 7.54882 | 7.54832 | 7.54768 | 7.5468 |
| 0.074 | 7.64876 | 7.64823 | 7.64758 | 7.64666 |
| 0.075 | 7.74874 | 7.7482 | 7.74752 | 7.74656 |
| 0.076 | 7.84877 | 7.84821 | 7.8475 | 7.84651 |
| 0.077 | 7.94884 | 7.94826 | 7.94752 | 7.94649 |
| 0.078 | 8.04895 | 8.04835 | 8.04759 | 8.04652 |
| 0.079 | 8.14911 | 8.14848 | 8.14769 | 8.14659 |
| 0.08 | 8.2493 | 8.24865 | 8.24784 | 8.2467 |
| 0.081 | 8.34953 | 8.34885 | 8.34801 | 8.34684 |
| 0.082 | 8.44979 | 8.4491 | 8.44823 | 8.44701 |
| 0.083 | 8.55009 | 8.54937 | 8.54848 | 8.54722 |
| 0.084 | 8.65042 | 8.64968 | 8.64876 | 8.64746 |
| 0.085 | 8.75079 | 8.75003 | 8.74907 | 8.74774 |
| 0.086 | 8.85118 | 8.8504 | 8.84942 | 8.84804 |
| 0.087 | 8.95161 | 8.95081 | 8.94979 | 8.94838 |
| 0.088 | 9.05207 | 9.05124 | 9.0502 | 9.04874 |
| 0.089 | 9.15256 | 9.1517 | 9.15063 | 9.14913 |
| 0.09 | 9.25307 | 9.25219 | 9.25109 | 9.24955 |
| 0.091 | 9.35361 | 9.35271 | 9.35158 | 9.34999 |
| 0.092 | 9.45418 | 9.45325 | 9.45209 | 9.45046 |
| 0.093 | 9.55478 | 9.55382 | 9.55263 | 9.55095 |
| 0.094 | 9.6554 | 9.65442 | 9.65319 | 9.65147 |
| 0.095 | 9.75604 | 9.75503 | 9.75378 | 9.75201 |
| 0.096 | 9.85671 | 9.85567 | 9.85438 | 9.85258 |
| 0.097 | 9.9574 | 9.95634 | 9.95502 | 9.95316 |
| 0.098 | 10.05811 | 10.05702 | 10.05567 | 10.05377 |
| 0.099 | 10.15884 | 10.15773 | 10.15634 | 10.15439 |
| 0.1 | 10.2596 | 10.25846 | 10.25704 | 10.25504 |
| 0.101 | 10.36037 | 10.35921 | 10.35775 | 10.35571 |
| 0.102 | 10.46117 | 10.45998 | 10.45849 | 10.45639 |
| 0.103 | 10.56199 | 10.56076 | 10.55924 | 10.5571 |
| 0.104 | 10.66282 | 10.66157 | 10.66001 | 10.65782 |
| 0.105 | 10.76367 | 10.7624 | 10.7608 | 10.75856 |
| 0.106 | 10.86455 | 10.86324 | 10.86161 | 10.85932 |
| 0.107 | 10.96543 | 10.9641 | 10.96243 | 10.96009 |
| 0.108 | 11.06634 | 11.06498 | 11.06327 | 11.06088 |
| 0.109 | 11.16726 | 11.16587 | 11.16413 | 11.16169 |
| 0.11 | 11.26821 | 11.26678 | 11.265 | 11.26251 |
| 0.111 | 11.36916 | 11.36771 | 11.36589 | 11.36334 |
| 0.112 | 11.47013 | 11.46865 | 11.46679 | 11.46419 |
| 0.113 | 11.57112 | 11.56961 | 11.56771 | 11.56506 |
| 0.114 | 11.67212 | 11.67058 | 11.66865 | 11.66594 |
| 0.115 | 11.77314 | 11.77157 | 11.76959 | 11.76683 |
| 0.116 | 11.87417 | 11.87257 | 11.87056 | 11.86774 |
| 0.117 | 11.97522 | 11.97358 | 11.97153 | 11.96866 |
| 0.118 | 12.07628 | 12.07461 | 12.07252 | 12.06959 |
| 0.119 | 12.17735 | 12.17565 | 12.17352 | 12.17053 |
| 0.12 | 12.27844 | 12.2767 | 12.27453 | 12.27149 |
| 0.121 | 12.37954 | 12.37777 | 12.37556 | 12.37246 |
| 0.122 | 12.48065 | 12.47885 | 12.4766 | 12.47344 |
| 0.123 | 12.58178 | 12.57994 | 12.57765 | 12.57443 |
| 0.124 | 12.68291 | 12.68104 | 12.67871 | 12.67543 |
| 0.125 | 12.78406 | 12.78216 | 12.77978 | 12.77645 |
| 0.126 | 12.88522 | 12.88329 | 12.88086 | 12.87747 |
| 0.127 | 12.9864 | 12.98442 | 12.98196 | 12.97851 |
| 0.128 | 13.08758 | 13.08557 | 13.08307 | 13.07955 |
| 0.129 | 13.18877 | 13.18673 | 13.18418 | 13.18061 |
| 0.13 | 13.28998 | 13.2879 | 13.28531 | 13.28167 |
| 0.131 | 13.3912 | 13.38908 | 13.38644 | 13.38274 |
| 0.132 | 13.49242 | 13.49028 | 13.48759 | 13.48383 |
| 0.133 | 13.59366 | 13.59148 | 13.58875 | 13.58492 |
| 0.134 | 13.69491 | 13.69269 | 13.68991 | 13.68602 |
| 0.135 | 13.79616 | 13.79391 | 13.79109 | 13.78713 |
| 0.136 | 13.89743 | 13.89514 | 13.89227 | 13.88825 |
| 0.137 | 13.99871 | 13.99638 | 13.99346 | 13.98938 |
| 0.138 | 14.09999 | 14.09763 | 14.09467 | 14.09052 |
| 0.139 | 14.20129 | 14.19889 | 14.19588 | 14.19166 |
| 0.14 | 14.3026 | 14.30015 | 14.2971 | 14.29282 |
| 0.141 | 14.40391 | 14.40143 | 14.39832 | 14.39398 |
| 0.142 | 14.50523 | 14.50271 | 14.49956 | 14.49515 |
| 0.143 | 14.60656 | 14.604 | 14.6008 | 14.59632 |
| 0.144 | 14.7079 | 14.7053 | 14.70206 | 14.69751 |
| 0.145 | 14.80925 | 14.80661 | 14.80332 | 14.7987 |
| 0.146 | 14.91061 | 14.90793 | 14.90458 | 14.89989 |
| 0.147 | 15.01197 | 15.00926 | 15.00586 | 15.0011 |
| 0.148 | 15.11335 | 15.11059 | 15.10714 | 15.10231 |
| 0.149 | 15.21473 | 15.21193 | 15.20843 | 15.20353 |
| 0.15 | 15.31612 | 15.31328 | 15.30973 | 15.30476 |
