# Supplementary material for: Analysis of sinusoidal post-buckling deformation of horizontal coiled tubing with initial residual bending
Source: PLoS One. 2024 May 14;19(5):e0301610. doi: 10.1371/journal.pone.0301610 (PMC11093391; doi:10.1371/journal.pone.0301610)
Supplement: S1 File — (ZIP) [file pone.0301610.s001.zip › The values used to build graphs - Fig 4 (c).docx]

## The values used to build graphs

The minimal data set of the original data for plotting curves in Fig 4 (c) is as follows:

| x-axis | m=2 | m=20 | m=40 | m=50 |
| --- | --- | --- | --- | --- |
| 0 | 1.00131 | 1.13539 | 1.55271 | 1.79623 |
| 0.0002 | 1.03576 | 1.17289 | 1.59287 | 1.83004 |
| 0.0004 | 1.07135 | 1.2114 | 1.63335 | 1.86358 |
| 0.0006 | 1.10807 | 1.2509 | 1.6741 | 1.89682 |
| 0.0008 | 1.14593 | 1.29138 | 1.71509 | 1.92973 |
| 0.001 | 1.18489 | 1.3328 | 1.75627 | 1.9623 |
| 0.0012 | 1.22495 | 1.37512 | 1.79762 | 1.99448 |
| 0.0014 | 1.26609 | 1.41833 | 1.83909 | 2.02627 |
| 0.0016 | 1.30829 | 1.46238 | 1.88065 | 2.05763 |
| 0.0018 | 1.35151 | 1.50725 | 1.92228 | 2.08854 |
| 0.002 | 1.39573 | 1.55289 | 1.96394 | 2.11899 |
| 0.0022 | 1.44092 | 1.59928 | 2.00561 | 2.14896 |
| 0.0024 | 1.48706 | 1.64637 | 2.04724 | 2.17842 |
| 0.0026 | 1.5341 | 1.69414 | 2.08883 | 2.20736 |
| 0.0028 | 1.58202 | 1.74254 | 2.13034 | 2.23577 |
| 0.003 | 1.63079 | 1.79155 | 2.17176 | 2.26363 |
| 0.0032 | 1.68037 | 1.84112 | 2.21305 | 2.29092 |
| 0.0034 | 1.73072 | 1.89124 | 2.25419 | 2.31763 |
| 0.0036 | 1.78182 | 1.94186 | 2.29518 | 2.34375 |
| 0.0038 | 1.83362 | 1.99295 | 2.33598 | 2.36927 |
| 0.004 | 1.88611 | 2.04449 | 2.37659 | 2.39417 |
| 0.0042 | 1.93925 | 2.09645 | 2.41697 | 2.41845 |
| 0.0044 | 1.99301 | 2.1488 | 2.45713 | 2.44209 |
| 0.0046 | 2.04736 | 2.20152 | 2.49703 | 2.46509 |
| 0.0048 | 2.10227 | 2.25457 | 2.53668 | 2.48744 |
| 0.005 | 2.15771 | 2.30794 | 2.57605 | 2.50912 |
| 0.0052 | 2.21366 | 2.36161 | 2.61513 | 2.53013 |
| 0.0054 | 2.27009 | 2.41554 | 2.65391 | 2.55047 |
| 0.0056 | 2.32699 | 2.46973 | 2.69238 | 2.57012 |
| 0.0058 | 2.38432 | 2.52416 | 2.73053 | 2.58908 |
| 0.006 | 2.44207 | 2.57879 | 2.76835 | 2.60734 |
| 0.0062 | 2.50021 | 2.63363 | 2.80583 | 2.6249 |
| 0.0064 | 2.55873 | 2.68864 | 2.84295 | 2.64175 |
| 0.0066 | 2.6176 | 2.74383 | 2.87972 | 2.65789 |
| 0.0068 | 2.67681 | 2.79916 | 2.91612 | 2.6733 |
| 0.007 | 2.73635 | 2.85463 | 2.95215 | 2.68799 |
| 0.0072 | 2.79619 | 2.91022 | 2.98779 | 2.70195 |
| 0.0074 | 2.85632 | 2.96593 | 3.02305 | 2.71517 |
| 0.0076 | 2.91673 | 3.02174 | 3.05791 | 2.72766 |
| 0.0078 | 2.97741 | 3.07764 | 3.09237 | 2.7394 |
| 0.008 | 3.03833 | 3.13361 | 3.12643 | 2.7504 |
| 0.0082 | 3.0995 | 3.18966 | 3.16007 | 2.76065 |
| 0.0084 | 3.16089 | 3.24576 | 3.19329 | 2.77015 |
| 0.0086 | 3.2225 | 3.30192 | 3.2261 | 2.77889 |
| 0.0088 | 3.28432 | 3.35811 | 3.25847 | 2.78687 |
| 0.009 | 3.34634 | 3.41435 | 3.29042 | 2.79408 |
| 0.0092 | 3.40854 | 3.47061 | 3.32193 | 2.80054 |
| 0.0094 | 3.47093 | 3.52689 | 3.353 | 2.80622 |
| 0.0096 | 3.53349 | 3.58318 | 3.38363 | 2.81113 |
| 0.0098 | 3.59621 | 3.63948 | 3.41381 | 2.81527 |
| 0.01 | 3.65909 | 3.69578 | 3.44354 | 2.81864 |
| 0.0102 | 3.72212 | 3.75208 | 3.47281 | 2.82123 |
| 0.0104 | 3.78529 | 3.80837 | 3.50163 | 2.82303 |
| 0.0106 | 3.8486 | 3.86464 | 3.52999 | 2.82406 |
| 0.0108 | 3.91204 | 3.92089 | 3.55789 | 2.8243 |
| 0.011 | 3.97561 | 3.97712 | 3.58532 | 2.82376 |
| 0.0112 | 4.0393 | 4.03332 | 3.61228 | 2.82243 |
| 0.0114 | 4.1031 | 4.08948 | 3.63878 | 2.82031 |
| 0.0116 | 4.16702 | 4.14561 | 3.6648 | 2.8174 |
| 0.0118 | 4.23104 | 4.2017 | 3.69034 | 2.81369 |
| 0.012 | 4.29517 | 4.25775 | 3.71541 | 2.80919 |
| 0.0122 | 4.35939 | 4.31374 | 3.74 | 2.8039 |
| 0.0124 | 4.42371 | 4.36969 | 3.76411 | 2.79781 |
| 0.0126 | 4.48811 | 4.42558 | 3.78773 | 2.79092 |
| 0.0128 | 4.55261 | 4.48141 | 3.81087 | 2.78324 |
| 0.013 | 4.61719 | 4.53718 | 3.83353 | 2.77475 |
| 0.0132 | 4.68185 | 4.5929 | 3.85569 | 2.76546 |
| 0.0134 | 4.74659 | 4.64854 | 3.87737 | 2.75536 |
| 0.0136 | 4.8114 | 4.70412 | 3.89855 | 2.74447 |
| 0.0138 | 4.87628 | 4.75963 | 3.91924 | 2.73276 |
| 0.014 | 4.94123 | 4.81507 | 3.93944 | 2.72026 |
| 0.0142 | 5.00625 | 4.87043 | 3.95914 | 2.70694 |
| 0.0144 | 5.07134 | 4.92571 | 3.97835 | 2.69282 |
| 0.0146 | 5.13649 | 4.98092 | 3.99705 | 2.67788 |
| 0.0148 | 5.20169 | 5.03605 | 4.01526 | 2.66214 |
| 0.015 | 5.26696 | 5.09109 | 4.03297 | 2.64558 |
| 0.0152 | 5.33228 | 5.14606 | 4.05017 | 2.62822 |
| 0.0154 | 5.39765 | 5.20093 | 4.06688 | 2.61004 |
| 0.0156 | 5.46308 | 5.25572 | 4.08307 | 2.59105 |
| 0.0158 | 5.52856 | 5.31042 | 4.09877 | 2.57124 |
| 0.016 | 5.59408 | 5.36504 | 4.11396 | 2.55062 |
| 0.0162 | 5.65965 | 5.41956 | 4.12864 | 2.52919 |
| 0.0164 | 5.72527 | 5.47398 | 4.14281 | 2.50693 |
| 0.0166 | 5.79094 | 5.52832 | 4.15648 | 2.48387 |
| 0.0168 | 5.85664 | 5.58256 | 4.16964 | 2.45998 |
| 0.017 | 5.92239 | 5.6367 | 4.18229 | 2.43528 |
| 0.0172 | 5.98818 | 5.69075 | 4.19442 | 2.40975 |
| 0.0174 | 6.054 | 5.74469 | 4.20605 | 2.38341 |
| 0.0176 | 6.11987 | 5.79854 | 4.21716 | 2.35625 |
| 0.0178 | 6.18577 | 5.85229 | 4.22776 | 2.32827 |
| 0.018 | 6.2517 | 5.90594 | 4.23785 | 2.29946 |
| 0.0182 | 6.31767 | 5.95948 | 4.24742 | 2.26984 |
| 0.0184 | 6.38367 | 6.01292 | 4.25648 | 2.2394 |
| 0.0186 | 6.44971 | 6.06626 | 4.26503 | 2.20813 |
| 0.0188 | 6.51577 | 6.11949 | 4.27305 | 2.17604 |
| 0.019 | 6.58187 | 6.17261 | 4.28057 | 2.14312 |
| 0.0192 | 6.648 | 6.22563 | 4.28756 | 2.10939 |
| 0.0194 | 6.71415 | 6.27854 | 4.29404 | 2.07483 |
| 0.0196 | 6.78034 | 6.33135 | 4.29999 | 2.03944 |
| 0.0198 | 6.84655 | 6.38404 | 4.30543 | 2.00323 |
| 0.02 | 6.91278 | 6.43663 | 4.31036 | 1.96619 |
| 0.0202 | 6.97904 | 6.48911 | 4.31476 | 1.92833 |
| 0.0204 | 7.04533 | 6.54147 | 4.31864 | 1.88965 |
| 0.0206 | 7.11164 | 6.59373 | 4.322 | 1.85013 |
| 0.0208 | 7.17798 | 6.64587 | 4.32484 | 1.80979 |
| 0.021 | 7.24434 | 6.6979 | 4.32716 | 1.76863 |
| 0.0212 | 7.31072 | 6.74982 | 4.32896 | 1.72663 |
| 0.0214 | 7.37712 | 6.80162 | 4.33023 | 1.68381 |
| 0.0216 | 7.44354 | 6.85331 | 4.33099 | 1.64016 |
| 0.0218 | 7.50998 | 6.90488 | 4.33122 | 1.59568 |
| 0.022 | 7.57645 | 6.95634 | 4.33093 | 1.55038 |
| 0.0222 | 7.64293 | 7.00769 | 4.33011 | 1.50424 |
| 0.0224 | 7.70943 | 7.05892 | 4.32877 | 1.45728 |
| 0.0226 | 7.77595 | 7.11003 | 4.32691 | 1.40948 |
| 0.0228 | 7.84249 | 7.16103 | 4.32452 | 1.36086 |
| 0.023 | 7.90905 | 7.21191 | 4.32161 | 1.31141 |
| 0.0232 | 7.97562 | 7.26267 | 4.31817 | 1.26112 |
| 0.0234 | 8.04221 | 7.31331 | 4.31421 | 1.21001 |
| 0.0236 | 8.10882 | 7.36384 | 4.30972 | 1.15807 |
| 0.0238 | 8.17544 | 7.41424 | 4.3047 | 1.10529 |
| 0.024 | 8.24208 | 7.46453 | 4.29916 | 1.05168 |
| 0.0242 | 8.30873 | 7.5147 | 4.29309 | 0.99725 |
| 0.0244 | 8.3754 | 7.56475 | 4.2865 | 0.94198 |
| 0.0246 | 8.44208 | 7.61468 | 4.27938 | 0.88588 |
| 0.0248 | 8.50878 | 7.66448 | 4.27173 | 0.82894 |
| 0.025 | 8.57549 | 7.71417 | 4.26356 | 0.77118 |
| 0.0252 | 8.64222 | 7.76374 | 4.25485 | 0.71258 |
| 0.0254 | 8.70895 | 7.81318 | 4.24562 | 0.65315 |
| 0.0256 | 8.7757 | 7.86251 | 4.23586 | 0.59289 |
| 0.0258 | 8.84247 | 7.91171 | 4.22558 | 0.53179 |
| 0.026 | 8.90924 | 7.96079 | 4.21476 | 0.46986 |
| 0.0262 | 8.97603 | 8.00975 | 4.20342 | 0.4071 |
| 0.0264 | 9.04283 | 8.05859 | 4.19154 | 0.3435 |
| 0.0266 | 9.10964 | 8.1073 | 4.17914 | 0.27907 |
| 0.0268 | 9.17646 | 8.15589 | 4.16621 | 0.21381 |
| 0.027 | 9.24329 | 8.20436 | 4.15275 | 0.14771 |
| 0.0272 | 9.31014 | 8.2527 | 4.13876 | 0.08078 |
| 0.0274 | 9.37699 | 8.30092 | 4.12423 | 0.01301 |
| 0.0276 | 9.44385 | 8.34902 | 4.10918 | -0.05559 |
| 0.0278 | 9.51073 | 8.39699 | 4.0936 | -0.12503 |
| 0.028 | 9.57761 | 8.44484 | 4.07749 | -0.1953 |
| 0.0282 | 9.64451 | 8.49256 | 4.06085 | -0.2664 |
| 0.0284 | 9.71141 | 8.54016 | 4.04368 | -0.33834 |
| 0.0286 | 9.77832 | 8.58763 | 4.02597 | -0.41112 |
| 0.0288 | 9.84524 | 8.63498 | 4.00774 | -0.48473 |
| 0.029 | 9.91217 | 8.6822 | 3.98897 | -0.55918 |
| 0.0292 | 9.97911 | 8.7293 | 3.96967 | -0.63446 |
| 0.0294 | 10.04606 | 8.77627 | 3.94984 | -0.71058 |
| 0.0296 | 10.11301 | 8.82312 | 3.92948 | -0.78754 |
| 0.0298 | 10.17998 | 8.86984 | 3.90859 | -0.86533 |
| 0.03 | 10.24695 | 8.91644 | 3.88717 | -0.94396 |
| 0.0302 | 10.31393 | 8.96291 | 3.86521 | -1.02342 |
| 0.0304 | 10.38092 | 9.00925 | 3.84272 | -1.10372 |
| 0.0306 | 10.44791 | 9.05547 | 3.8197 | -1.18486 |
| 0.0308 | 10.51492 | 9.10156 | 3.79615 | -1.26683 |
| 0.031 | 10.58192 | 9.14752 | 3.77206 | -1.34964 |
| 0.0312 | 10.64894 | 9.19336 | 3.74745 | -1.43329 |
| 0.0314 | 10.71596 | 9.23906 | 3.72229 | -1.51777 |
| 0.0316 | 10.783 | 9.28465 | 3.69661 | -1.60309 |
| 0.0318 | 10.85003 | 9.3301 | 3.67039 | -1.68925 |
| 0.032 | 10.91708 | 9.37543 | 3.64364 | -1.77625 |
| 0.0322 | 10.98413 | 9.42063 | 3.61636 | -1.86408 |
| 0.0324 | 11.05118 | 9.4657 | 3.58854 | -1.95276 |
| 0.0326 | 11.11824 | 9.51065 | 3.56019 | -2.04227 |
| 0.0328 | 11.18531 | 9.55546 | 3.53131 | -2.13261 |
| 0.033 | 11.25239 | 9.60015 | 3.50189 | -2.2238 |
| 0.0332 | 11.31947 | 9.64471 | 3.47194 | -2.31582 |
| 0.0334 | 11.38655 | 9.68915 | 3.44146 | -2.40869 |
| 0.0336 | 11.45364 | 9.73345 | 3.41044 | -2.50239 |
| 0.0338 | 11.52074 | 9.77763 | 3.37888 | -2.59692 |
| 0.034 | 11.58784 | 9.82167 | 3.3468 | -2.6923 |
| 0.0342 | 11.65495 | 9.86559 | 3.31418 | -2.78852 |
| 0.0344 | 11.72207 | 9.90938 | 3.28102 | -2.88557 |
| 0.0346 | 11.78918 | 9.95304 | 3.24733 | -2.98346 |
| 0.0348 | 11.85631 | 9.99658 | 3.2131 | -3.0822 |
| 0.035 | 11.92343 | 10.03998 | 3.17834 | -3.18177 |
| 0.0352 | 11.99057 | 10.08326 | 3.14305 | -3.28218 |
| 0.0354 | 12.0577 | 10.1264 | 3.10722 | -3.38343 |
| 0.0356 | 12.12485 | 10.16942 | 3.07086 | -3.48551 |
| 0.0358 | 12.19199 | 10.2123 | 3.03396 | -3.58844 |
| 0.036 | 12.25915 | 10.25506 | 2.99653 | -3.69221 |
| 0.0362 | 12.3263 | 10.29769 | 2.95856 | -3.79681 |
| 0.0364 | 12.39346 | 10.34019 | 2.92005 | -3.90226 |
| 0.0366 | 12.46063 | 10.38256 | 2.88101 | -4.00854 |
| 0.0368 | 12.52779 | 10.4248 | 2.84144 | -4.11567 |
| 0.037 | 12.59497 | 10.46691 | 2.80133 | -4.22363 |
| 0.0372 | 12.66214 | 10.50889 | 2.76068 | -4.33244 |
| 0.0374 | 12.72932 | 10.55074 | 2.7195 | -4.44208 |
| 0.0376 | 12.79651 | 10.59246 | 2.67778 | -4.55257 |
| 0.0378 | 12.8637 | 10.63405 | 2.63553 | -4.66389 |
| 0.038 | 12.93089 | 10.67551 | 2.59274 | -4.77606 |
| 0.0382 | 12.99809 | 10.71684 | 2.54942 | -4.88906 |
| 0.0384 | 13.06529 | 10.75804 | 2.50556 | -5.00291 |
| 0.0386 | 13.13249 | 10.79911 | 2.46116 | -5.11759 |
| 0.0388 | 13.1997 | 10.84005 | 2.41623 | -5.23312 |
| 0.039 | 13.26691 | 10.88086 | 2.37076 | -5.34949 |
| 0.0392 | 13.33412 | 10.92153 | 2.32476 | -5.46669 |
| 0.0394 | 13.40134 | 10.96208 | 2.27822 | -5.58474 |
| 0.0396 | 13.46856 | 11.0025 | 2.23114 | -5.70363 |
| 0.0398 | 13.53578 | 11.04279 | 2.18352 | -5.82336 |
| 0.04 | 13.60301 | 11.08294 | 2.13537 | -5.94393 |
| 0.0402 | 13.67024 | 11.12297 | 2.08669 | -6.06534 |
| 0.0404 | 13.73747 | 11.16286 | 2.03746 | -6.1876 |
| 0.0406 | 13.80471 | 11.20262 | 1.98771 | -6.31069 |
| 0.0408 | 13.87194 | 11.24226 | 1.93741 | -6.43462 |
| 0.041 | 13.93919 | 11.28176 | 1.88658 | -6.5594 |
| 0.0412 | 14.00643 | 11.32113 | 1.83521 | -6.68502 |
| 0.0414 | 14.07368 | 11.36037 | 1.7833 | -6.81148 |
| 0.0416 | 14.14093 | 11.39948 | 1.73085 | -6.93878 |
| 0.0418 | 14.20818 | 11.43845 | 1.67787 | -7.06692 |
| 0.042 | 14.27544 | 11.4773 | 1.62436 | -7.1959 |
| 0.0422 | 14.3427 | 11.51601 | 1.5703 | -7.32573 |
| 0.0424 | 14.40996 | 11.5546 | 1.51571 | -7.45639 |
| 0.0426 | 14.47722 | 11.59305 | 1.46058 | -7.5879 |
| 0.0428 | 14.54449 | 11.63137 | 1.40491 | -7.72025 |
| 0.043 | 14.61176 | 11.66956 | 1.34871 | -7.85344 |
| 0.0432 | 14.67903 | 11.70762 | 1.29197 | -7.98748 |
| 0.0434 | 14.7463 | 11.74555 | 1.23469 | -8.12235 |
| 0.0436 | 14.81358 | 11.78334 | 1.17687 | -8.25807 |
| 0.0438 | 14.88085 | 11.821 | 1.11852 | -8.39463 |
| 0.044 | 14.94814 | 11.85854 | 1.05963 | -8.53203 |
| 0.0442 | 15.01542 | 11.89594 | 1.0002 | -8.67027 |
| 0.0444 | 15.0827 | 11.9332 | 0.94023 | -8.80936 |
| 0.0446 | 15.14999 | 11.97034 | 0.87973 | -8.94929 |
| 0.0448 | 15.21728 | 12.00734 | 0.81868 | -9.09006 |
| 0.045 | 15.28457 | 12.04422 | 0.7571 | -9.23167 |
| 0.0452 | 15.35186 | 12.08096 | 0.69499 | -9.37413 |
| 0.0454 | 15.41916 | 12.11757 | 0.63233 | -9.51743 |
| 0.0456 | 15.48646 | 12.15405 | 0.56914 | -9.66157 |
| 0.0458 | 15.55376 | 12.19039 | 0.50541 | -9.80655 |
| 0.046 | 15.62106 | 12.2266 | 0.44114 | -9.95238 |
| 0.0462 | 15.68836 | 12.26269 | 0.37633 | -10.0991 |
| 0.0464 | 15.75567 | 12.29863 | 0.31098 | -10.2466 |
| 0.0466 | 15.82297 | 12.33445 | 0.2451 | -10.3949 |
| 0.0468 | 15.89028 | 12.37014 | 0.17868 | -10.5441 |
| 0.047 | 15.95759 | 12.40569 | 0.11172 | -10.6942 |
| 0.0472 | 16.02491 | 12.44111 | 0.04422 | -10.845 |
| 0.0474 | 16.09222 | 12.4764 | -0.02382 | -10.9968 |
| 0.0476 | 16.15954 | 12.51155 | -0.0924 | -11.1493 |
| 0.0478 | 16.22685 | 12.54658 | -0.16151 | -11.3028 |
| 0.048 | 16.29417 | 12.58147 | -0.23116 | -11.457 |
| 0.0482 | 16.36149 | 12.61623 | -0.30135 | -11.6121 |
| 0.0484 | 16.42882 | 12.65086 | -0.37208 | -11.7681 |
| 0.0486 | 16.49614 | 12.68535 | -0.44335 | -11.9248 |
| 0.0488 | 16.56347 | 12.71971 | -0.51516 | -12.0825 |
| 0.049 | 16.63079 | 12.75394 | -0.58751 | -12.2409 |
| 0.0492 | 16.69812 | 12.78804 | -0.66039 | -12.4003 |
| 0.0494 | 16.76545 | 12.822 | -0.73381 | -12.5604 |
| 0.0496 | 16.83279 | 12.85583 | -0.80778 | -12.7214 |
| 0.0498 | 16.90012 | 12.88953 | -0.88228 | -12.8833 |
| 0.05 | 16.96745 | 12.9231 | -0.95732 | -13.046 |
