# Supplementary material for: Analysis of sinusoidal post-buckling deformation of horizontal coiled tubing with initial residual bending
Source: PLoS One. 2024 May 14;19(5):e0301610. doi: 10.1371/journal.pone.0301610 (PMC11093391; doi:10.1371/journal.pone.0301610)
Supplement: S1 File — (ZIP) [file pone.0301610.s001.zip › The values used to build graphs - Fig 4 (b).docx]

## The values used to build graphs

The minimal data set of the original data for plotting curves in Fig 4 (b) is as follows:

| x-axis | m=2 | m=20 | m=40 | Wu (1995) |
| --- | --- | --- | --- | --- |
| 0 | 1.00047 | 1.04781 | 1.19703 | 1 |
| 0.001 | 1.10717 | 1.15794 | 1.31555 | 1.10666 |
| 0.002 | 1.22399 | 1.27777 | 1.44196 | 1.22345 |
| 0.003 | 1.3505 | 1.40669 | 1.57537 | 1.34993 |
| 0.004 | 1.48602 | 1.54395 | 1.7148 | 1.48543 |
| 0.005 | 1.62972 | 1.68865 | 1.85931 | 1.62912 |
| 0.006 | 1.78074 | 1.83989 | 2.00799 | 1.78014 |
| 0.007 | 1.93818 | 1.99678 | 2.16003 | 1.93758 |
| 0.008 | 2.10121 | 2.15852 | 2.3147 | 2.10062 |
| 0.009 | 2.26908 | 2.32437 | 2.47139 | 2.2685 |
| 0.01 | 2.44109 | 2.49369 | 2.62956 | 2.44054 |
| 0.011 | 2.61668 | 2.66593 | 2.78875 | 2.61616 |
| 0.012 | 2.79534 | 2.84062 | 2.94858 | 2.79486 |
| 0.013 | 2.97663 | 3.01736 | 3.10873 | 2.9762 |
| 0.014 | 3.16021 | 3.19581 | 3.26893 | 3.15982 |
| 0.015 | 3.34575 | 3.37569 | 3.42894 | 3.34542 |
| 0.016 | 3.53301 | 3.55674 | 3.58856 | 3.53274 |
| 0.017 | 3.72175 | 3.73878 | 3.74764 | 3.72155 |
| 0.018 | 3.9118 | 3.92161 | 3.90602 | 3.91167 |
| 0.019 | 4.103 | 4.1051 | 4.06359 | 4.10294 |
| 0.02 | 4.29521 | 4.28912 | 4.22024 | 4.29523 |
| 0.021 | 4.48831 | 4.47355 | 4.37588 | 4.48841 |
| 0.022 | 4.6822 | 4.65831 | 4.53044 | 4.6824 |
| 0.023 | 4.8768 | 4.84331 | 4.68385 | 4.87709 |
| 0.024 | 5.07204 | 5.02849 | 4.83604 | 5.07243 |
| 0.025 | 5.26784 | 5.21377 | 4.98698 | 5.26834 |
| 0.026 | 5.46416 | 5.39912 | 5.1366 | 5.46476 |
| 0.027 | 5.66093 | 5.58448 | 5.28488 | 5.66165 |
| 0.028 | 5.85813 | 5.76981 | 5.43178 | 5.85896 |
| 0.029 | 6.05571 | 5.95507 | 5.57726 | 6.05666 |
| 0.03 | 6.25363 | 6.14024 | 5.7213 | 6.25472 |
| 0.031 | 6.45187 | 6.32527 | 5.86387 | 6.45309 |
| 0.032 | 6.6504 | 6.51016 | 6.00496 | 6.65176 |
| 0.033 | 6.8492 | 6.69488 | 6.14454 | 6.85069 |
| 0.034 | 7.04824 | 6.87939 | 6.28259 | 7.04988 |
| 0.035 | 7.24751 | 7.0637 | 6.4191 | 7.2493 |
| 0.036 | 7.44699 | 7.24777 | 6.55405 | 7.44893 |
| 0.037 | 7.64666 | 7.4316 | 6.68744 | 7.64876 |
| 0.038 | 7.84651 | 7.61518 | 6.81924 | 7.84877 |
| 0.039 | 8.04653 | 7.79848 | 6.94945 | 8.04895 |
| 0.04 | 8.2467 | 7.9815 | 7.07806 | 8.2493 |
| 0.041 | 8.44702 | 8.16423 | 7.20505 | 8.44979 |
| 0.042 | 8.64747 | 8.34666 | 7.33043 | 8.65042 |
| 0.043 | 8.84805 | 8.52878 | 7.45418 | 8.85118 |
| 0.044 | 9.04874 | 8.71059 | 7.57629 | 9.05207 |
| 0.045 | 9.24955 | 8.89207 | 7.69676 | 9.25307 |
| 0.046 | 9.45047 | 9.07322 | 7.81559 | 9.45418 |
| 0.047 | 9.65148 | 9.25403 | 7.93276 | 9.6554 |
| 0.048 | 9.85258 | 9.4345 | 8.04827 | 9.85671 |
| 0.049 | 10.05377 | 9.61463 | 8.16212 | 10.05811 |
| 0.05 | 10.25505 | 9.7944 | 8.27431 | 10.2596 |
| 0.051 | 0.66779 | 0.66779 | 0.66779 | 0.66779 |
| 0.052 | 0.67596 | 0.67596 | 0.67596 | 0.67596 |
| 0.053 | 0.68411 | 0.68411 | 0.68411 | 0.68411 |
| 0.054 | 0.69223 | 0.69223 | 0.69223 | 0.69223 |
| 0.055 | 0.70032 | 0.70032 | 0.70032 | 0.70032 |
| 0.056 | 0.70838 | 0.70838 | 0.70838 | 0.70838 |
| 0.057 | 0.71643 | 0.71643 | 0.71643 | 0.71643 |
| 0.058 | 0.72445 | 0.72445 | 0.72445 | 0.72445 |
| 0.059 | 0.73245 | 0.73245 | 0.73245 | 0.73245 |
| 0.06 | 0.74042 | 0.74042 | 0.74042 | 0.74042 |
| 0.061 | 0.74838 | 0.74838 | 0.74838 | 0.74838 |
| 0.062 | 0.75632 | 0.75632 | 0.75632 | 0.75632 |
| 0.063 | 0.76425 | 0.76425 | 0.76425 | 0.76425 |
| 0.064 | 0.77215 | 0.77215 | 0.77215 | 0.77215 |
| 0.065 | 0.78004 | 0.78004 | 0.78004 | 0.78004 |
| 0.066 | 0.78791 | 0.78791 | 0.78791 | 0.78791 |
| 0.067 | 0.79577 | 0.79577 | 0.79577 | 0.79577 |
| 0.068 | 0.80361 | 0.80361 | 0.80361 | 0.80361 |
| 0.069 | 0.81145 | 0.81145 | 0.81145 | 0.81145 |
| 0.07 | 0.81926 | 0.81926 | 0.81926 | 0.81926 |
| 0.071 | 0.82707 | 0.82707 | 0.82707 | 0.82707 |
| 0.072 | 0.83486 | 0.83486 | 0.83486 | 0.83486 |
| 0.073 | 0.84265 | 0.84265 | 0.84265 | 0.84265 |
| 0.074 | 0.85042 | 0.85042 | 0.85042 | 0.85042 |
| 0.075 | 0.85818 | 0.85818 | 0.85818 | 0.85818 |
| 0.076 | 0.86593 | 0.86593 | 0.86593 | 0.86593 |
| 0.077 | 0.87368 | 0.87368 | 0.87368 | 0.87368 |
| 0.078 | 0.88141 | 0.88141 | 0.88141 | 0.88141 |
| 0.079 | 0.88914 | 0.88914 | 0.88914 | 0.88914 |
| 0.08 | 0.89686 | 0.89686 | 0.89686 | 0.89686 |
| 0.081 | 0.90457 | 0.90457 | 0.90457 | 0.90457 |
| 0.082 | 0.91227 | 0.91227 | 0.91227 | 0.91227 |
| 0.083 | 0.91997 | 0.91997 | 0.91997 | 0.91997 |
| 0.084 | 0.92766 | 0.92766 | 0.92766 | 0.92766 |
| 0.085 | 0.93535 | 0.93535 | 0.93535 | 0.93535 |
| 0.086 | 0.94303 | 0.94303 | 0.94303 | 0.94303 |
| 0.087 | 0.9507 | 0.9507 | 0.9507 | 0.9507 |
| 0.088 | 0.95837 | 0.95837 | 0.95837 | 0.95837 |
| 0.089 | 0.96603 | 0.96603 | 0.96603 | 0.96603 |
| 0.09 | 0.97369 | 0.97369 | 0.97369 | 0.97369 |
| 0.091 | 0.98135 | 0.98135 | 0.98135 | 0.98135 |
| 0.092 | 0.989 | 0.989 | 0.989 | 0.989 |
| 0.093 | 0.99665 | 0.99665 | 0.99665 | 0.99665 |
| 0.094 | 1.0043 | 1.0043 | 1.0043 | 1.0043 |
| 0.095 | 1.01194 | 1.01194 | 1.01194 | 1.01194 |
| 0.096 | 1.01958 | 1.01958 | 1.01958 | 1.01958 |
| 0.097 | 1.02721 | 1.02721 | 1.02721 | 1.02721 |
| 0.098 | 1.03485 | 1.03485 | 1.03485 | 1.03485 |
| 0.099 | 1.04248 | 1.04248 | 1.04248 | 1.04248 |
| 0.1 | 1.05011 | 1.05011 | 1.05011 | 1.05011 |
| 0.101 | 1.05773 | 1.05773 | 1.05773 | 1.05773 |
| 0.102 | 1.06536 | 1.06536 | 1.06536 | 1.06536 |
| 0.103 | 1.07298 | 1.07298 | 1.07298 | 1.07298 |
| 0.104 | 1.0806 | 1.0806 | 1.0806 | 1.0806 |
| 0.105 | 1.08822 | 1.08822 | 1.08822 | 1.08822 |
| 0.106 | 1.09584 | 1.09584 | 1.09584 | 1.09584 |
| 0.107 | 1.10346 | 1.10346 | 1.10346 | 1.10346 |
| 0.108 | 1.11108 | 1.11108 | 1.11108 | 1.11108 |
| 0.109 | 1.1187 | 1.1187 | 1.1187 | 1.1187 |
| 0.11 | 1.12631 | 1.12631 | 1.12631 | 1.12631 |
| 0.111 | 1.13393 | 1.13393 | 1.13393 | 1.13393 |
| 0.112 | 1.14155 | 1.14155 | 1.14155 | 1.14155 |
| 0.113 | 1.14916 | 1.14916 | 1.14916 | 1.14916 |
| 0.114 | 1.15678 | 1.15678 | 1.15678 | 1.15678 |
| 0.115 | 1.16439 | 1.16439 | 1.16439 | 1.16439 |
| 0.116 | 1.17201 | 1.17201 | 1.17201 | 1.17201 |
| 0.117 | 1.17963 | 1.17963 | 1.17963 | 1.17963 |
| 0.118 | 1.18724 | 1.18724 | 1.18724 | 1.18724 |
| 0.119 | 1.19486 | 1.19486 | 1.19486 | 1.19486 |
| 0.12 | 1.20248 | 1.20248 | 1.20248 | 1.20248 |
| 0.121 | 1.2101 | 1.2101 | 1.2101 | 1.2101 |
| 0.122 | 1.21771 | 1.21771 | 1.21771 | 1.21771 |
| 0.123 | 1.22533 | 1.22533 | 1.22533 | 1.22533 |
| 0.124 | 1.23295 | 1.23295 | 1.23295 | 1.23295 |
| 0.125 | 1.24058 | 1.24058 | 1.24058 | 1.24058 |
| 0.126 | 1.2482 | 1.2482 | 1.2482 | 1.2482 |
| 0.127 | 1.25582 | 1.25582 | 1.25582 | 1.25582 |
| 0.128 | 1.26345 | 1.26345 | 1.26345 | 1.26345 |
| 0.129 | 1.27107 | 1.27107 | 1.27107 | 1.27107 |
| 0.13 | 1.2787 | 1.2787 | 1.2787 | 1.2787 |
| 0.131 | 1.28633 | 1.28633 | 1.28633 | 1.28633 |
| 0.132 | 1.29396 | 1.29396 | 1.29396 | 1.29396 |
| 0.133 | 1.30159 | 1.30159 | 1.30159 | 1.30159 |
| 0.134 | 1.30922 | 1.30922 | 1.30922 | 1.30922 |
| 0.135 | 1.31686 | 1.31686 | 1.31686 | 1.31686 |
| 0.136 | 1.32449 | 1.32449 | 1.32449 | 1.32449 |
| 0.137 | 1.33213 | 1.33213 | 1.33213 | 1.33213 |
| 0.138 | 1.33977 | 1.33977 | 1.33977 | 1.33977 |
| 0.139 | 1.34741 | 1.34741 | 1.34741 | 1.34741 |
| 0.14 | 1.35506 | 1.35506 | 1.35506 | 1.35506 |
| 0.141 | 1.3627 | 1.3627 | 1.3627 | 1.3627 |
| 0.142 | 1.37035 | 1.37035 | 1.37035 | 1.37035 |
| 0.143 | 1.378 | 1.378 | 1.378 | 1.378 |
| 0.144 | 1.38565 | 1.38565 | 1.38565 | 1.38565 |
| 0.145 | 1.3933 | 1.3933 | 1.3933 | 1.3933 |
| 0.146 | 1.40096 | 1.40096 | 1.40096 | 1.40096 |
| 0.147 | 1.40861 | 1.40861 | 1.40861 | 1.40861 |
| 0.148 | 1.41627 | 1.41627 | 1.41627 | 1.41627 |
| 0.149 | 1.42393 | 1.42393 | 1.42393 | 1.42393 |
| 0.15 | 1.4316 | 1.4316 | 1.4316 | 1.4316 |
| 0.151 | 1.43926 | 1.43926 | 1.43926 | 1.43926 |
| 0.152 | 1.44693 | 1.44693 | 1.44693 | 1.44693 |
| 0.153 | 1.4546 | 1.4546 | 1.4546 | 1.4546 |
| 0.154 | 1.46227 | 1.46227 | 1.46227 | 1.46227 |
| 0.155 | 1.46994 | 1.46994 | 1.46994 | 1.46994 |
| 0.156 | 1.47762 | 1.47762 | 1.47762 | 1.47762 |
| 0.157 | 1.4853 | 1.4853 | 1.4853 | 1.4853 |
| 0.158 | 1.49298 | 1.49298 | 1.49298 | 1.49298 |
| 0.159 | 1.50066 | 1.50066 | 1.50066 | 1.50066 |
| 0.16 | 1.50835 | 1.50835 | 1.50835 | 1.50835 |
| 0.161 | 1.51604 | 1.51604 | 1.51604 | 1.51604 |
| 0.162 | 1.52373 | 1.52373 | 1.52373 | 1.52373 |
| 0.163 | 1.53142 | 1.53142 | 1.53142 | 1.53142 |
| 0.164 | 1.53911 | 1.53911 | 1.53911 | 1.53911 |
| 0.165 | 1.54681 | 1.54681 | 1.54681 | 1.54681 |
| 0.166 | 1.55451 | 1.55451 | 1.55451 | 1.55451 |
| 0.167 | 1.56221 | 1.56221 | 1.56221 | 1.56221 |
| 0.168 | 1.56992 | 1.56992 | 1.56992 | 1.56992 |
| 0.169 | 1.57762 | 1.57762 | 1.57762 | 1.57762 |
| 0.17 | 1.58533 | 1.58533 | 1.58533 | 1.58533 |
| 0.171 | 1.59304 | 1.59304 | 1.59304 | 1.59304 |
| 0.172 | 1.60076 | 1.60076 | 1.60076 | 1.60076 |
| 0.173 | 1.60848 | 1.60848 | 1.60848 | 1.60848 |
| 0.174 | 1.61619 | 1.61619 | 1.61619 | 1.61619 |
| 0.175 | 1.62392 | 1.62392 | 1.62392 | 1.62392 |
| 0.176 | 1.63164 | 1.63164 | 1.63164 | 1.63164 |
| 0.177 | 1.63937 | 1.63937 | 1.63937 | 1.63937 |
| 0.178 | 1.6471 | 1.6471 | 1.6471 | 1.6471 |
| 0.179 | 1.65483 | 1.65483 | 1.65483 | 1.65483 |
| 0.18 | 1.66256 | 1.66256 | 1.66256 | 1.66256 |
| 0.181 | 1.6703 | 1.6703 | 1.6703 | 1.6703 |
| 0.182 | 1.67803 | 1.67803 | 1.67803 | 1.67803 |
| 0.183 | 1.68578 | 1.68578 | 1.68578 | 1.68578 |
| 0.184 | 1.69352 | 1.69352 | 1.69352 | 1.69352 |
| 0.185 | 1.70127 | 1.70127 | 1.70127 | 1.70127 |
| 0.186 | 1.70901 | 1.70901 | 1.70901 | 1.70901 |
| 0.187 | 1.71677 | 1.71677 | 1.71677 | 1.71677 |
| 0.188 | 1.72452 | 1.72452 | 1.72452 | 1.72452 |
| 0.189 | 1.73227 | 1.73227 | 1.73227 | 1.73227 |
| 0.19 | 1.74003 | 1.74003 | 1.74003 | 1.74003 |
| 0.191 | 1.74779 | 1.74779 | 1.74779 | 1.74779 |
| 0.192 | 1.75556 | 1.75556 | 1.75556 | 1.75556 |
| 0.193 | 1.76332 | 1.76332 | 1.76332 | 1.76332 |
| 0.194 | 1.77109 | 1.77109 | 1.77109 | 1.77109 |
| 0.195 | 1.77886 | 1.77886 | 1.77886 | 1.77886 |
| 0.196 | 1.78663 | 1.78663 | 1.78663 | 1.78663 |
| 0.197 | 1.79441 | 1.79441 | 1.79441 | 1.79441 |
| 0.198 | 1.80219 | 1.80219 | 1.80219 | 1.80219 |
| 0.199 | 1.80997 | 1.80997 | 1.80997 | 1.80997 |
| 0.2 | 1.81775 | 1.81775 | 1.81775 | 1.81775 |
