# Supplementary material for: Analysis of sinusoidal post-buckling deformation of horizontal coiled tubing with initial residual bending
Source: PLoS One. 2024 May 14;19(5):e0301610. doi: 10.1371/journal.pone.0301610 (PMC11093391; doi:10.1371/journal.pone.0301610)
Supplement: S1 File — (ZIP) [file pone.0301610.s001.zip › The values used to build graphs - Fig 4 (a).docx]

## The values used to build graphs

The minimal data set of the original data for plotting curves in Fig 4 (a) is as follows:

| x-axis | m=2 | m=20 | m=40 | Wu (1995) |
| --- | --- | --- | --- | --- |
| 0 | 1.00012 | 1.01184 | 1.04781 | 1 |
| 0.0002 | 1.01032 | 1.02214 | 1.05838 | 1.0102 |
| 0.0004 | 1.02063 | 1.03254 | 1.06905 | 1.02051 |
| 0.0006 | 1.03104 | 1.04304 | 1.07981 | 1.03092 |
| 0.0008 | 1.04155 | 1.05364 | 1.09068 | 1.04143 |
| 0.001 | 1.05217 | 1.06435 | 1.10164 | 1.05205 |
| 0.0012 | 1.06289 | 1.07515 | 1.11271 | 1.06277 |
| 0.0014 | 1.07371 | 1.08606 | 1.12387 | 1.07359 |
| 0.0016 | 1.08464 | 1.09707 | 1.13513 | 1.08451 |
| 0.0018 | 1.09566 | 1.10818 | 1.14649 | 1.09553 |
| 0.002 | 1.10679 | 1.1194 | 1.15794 | 1.10666 |
| 0.0022 | 1.11802 | 1.13071 | 1.1695 | 1.11789 |
| 0.0024 | 1.12935 | 1.14212 | 1.18115 | 1.12922 |
| 0.0026 | 1.14078 | 1.15364 | 1.1929 | 1.14065 |
| 0.0028 | 1.15231 | 1.16525 | 1.20474 | 1.15218 |
| 0.003 | 1.16394 | 1.17696 | 1.21668 | 1.16381 |
| 0.0032 | 1.17568 | 1.18877 | 1.22871 | 1.17554 |
| 0.0034 | 1.18751 | 1.20068 | 1.24083 | 1.18737 |
| 0.0036 | 1.19944 | 1.21268 | 1.25305 | 1.1993 |
| 0.0038 | 1.21146 | 1.22478 | 1.26536 | 1.21133 |
| 0.004 | 1.22359 | 1.23698 | 1.27777 | 1.22345 |
| 0.0042 | 1.23581 | 1.24927 | 1.29026 | 1.23567 |
| 0.0044 | 1.24813 | 1.26166 | 1.30285 | 1.24799 |
| 0.0046 | 1.26054 | 1.27415 | 1.31552 | 1.26041 |
| 0.0048 | 1.27305 | 1.28672 | 1.32828 | 1.27292 |
| 0.005 | 1.28566 | 1.29939 | 1.34114 | 1.28552 |
| 0.0052 | 1.29836 | 1.31215 | 1.35408 | 1.29822 |
| 0.0054 | 1.31115 | 1.32501 | 1.3671 | 1.31101 |
| 0.0056 | 1.32403 | 1.33795 | 1.38021 | 1.32389 |
| 0.0058 | 1.33701 | 1.35099 | 1.39341 | 1.33687 |
| 0.006 | 1.35007 | 1.36411 | 1.40669 | 1.34993 |
| 0.0062 | 1.36323 | 1.37733 | 1.42006 | 1.36309 |
| 0.0064 | 1.37648 | 1.39063 | 1.4335 | 1.37633 |
| 0.0066 | 1.38981 | 1.40401 | 1.44703 | 1.38967 |
| 0.0068 | 1.40324 | 1.41749 | 1.46064 | 1.40309 |
| 0.007 | 1.41675 | 1.43105 | 1.47433 | 1.4166 |
| 0.0072 | 1.43034 | 1.44469 | 1.4881 | 1.4302 |
| 0.0074 | 1.44403 | 1.45842 | 1.50195 | 1.44388 |
| 0.0076 | 1.45779 | 1.47223 | 1.51587 | 1.45765 |
| 0.0078 | 1.47164 | 1.48612 | 1.52987 | 1.4715 |
| 0.008 | 1.48558 | 1.50009 | 1.54395 | 1.48543 |
| 0.0082 | 1.49959 | 1.51415 | 1.5581 | 1.49944 |
| 0.0084 | 1.51369 | 1.52828 | 1.57232 | 1.51354 |
| 0.0086 | 1.52786 | 1.54249 | 1.58662 | 1.52772 |
| 0.0088 | 1.54212 | 1.55678 | 1.60098 | 1.54197 |
| 0.009 | 1.55645 | 1.57115 | 1.61542 | 1.55631 |
| 0.0092 | 1.57087 | 1.58559 | 1.62993 | 1.57072 |
| 0.0094 | 1.58536 | 1.6001 | 1.64451 | 1.58521 |
| 0.0096 | 1.59992 | 1.61469 | 1.65915 | 1.59977 |
| 0.0098 | 1.61456 | 1.62936 | 1.67387 | 1.61441 |
| 0.01 | 1.62927 | 1.64409 | 1.68865 | 1.62912 |
| 0.0102 | 1.64406 | 1.6589 | 1.70349 | 1.64391 |
| 0.0104 | 1.65892 | 1.67377 | 1.7184 | 1.65877 |
| 0.0106 | 1.67385 | 1.68872 | 1.73337 | 1.6737 |
| 0.0108 | 1.68885 | 1.70373 | 1.74841 | 1.6887 |
| 0.011 | 1.70392 | 1.71882 | 1.76351 | 1.70377 |
| 0.0112 | 1.71906 | 1.73396 | 1.77866 | 1.71891 |
| 0.0114 | 1.73427 | 1.74918 | 1.79388 | 1.73412 |
| 0.0116 | 1.74954 | 1.76446 | 1.80916 | 1.74939 |
| 0.0118 | 1.76488 | 1.7798 | 1.82449 | 1.76473 |
| 0.012 | 1.78029 | 1.79521 | 1.83989 | 1.78014 |
| 0.0122 | 1.79576 | 1.81068 | 1.85533 | 1.79561 |
| 0.0124 | 1.81129 | 1.82621 | 1.87084 | 1.81114 |
| 0.0126 | 1.82689 | 1.8418 | 1.8864 | 1.82673 |
| 0.0128 | 1.84254 | 1.85745 | 1.90201 | 1.84239 |
| 0.013 | 1.85826 | 1.87316 | 1.91768 | 1.85811 |
| 0.0132 | 1.87404 | 1.88893 | 1.9334 | 1.87389 |
| 0.0134 | 1.88987 | 1.90476 | 1.94917 | 1.88972 |
| 0.0136 | 1.90577 | 1.92064 | 1.96499 | 1.90562 |
| 0.0138 | 1.92172 | 1.93657 | 1.98086 | 1.92157 |
| 0.014 | 1.93773 | 1.95257 | 1.99678 | 1.93758 |
| 0.0142 | 1.9538 | 1.96861 | 2.01275 | 1.95365 |
| 0.0144 | 1.96992 | 1.98471 | 2.02877 | 1.96977 |
| 0.0146 | 1.98609 | 2.00086 | 2.04483 | 1.98594 |
| 0.0148 | 2.00232 | 2.01707 | 2.06094 | 2.00217 |
| 0.015 | 2.0186 | 2.03332 | 2.07709 | 2.01845 |
| 0.0152 | 2.03493 | 2.04963 | 2.09329 | 2.03479 |
| 0.0154 | 2.05132 | 2.06598 | 2.10953 | 2.05117 |
| 0.0156 | 2.06775 | 2.08238 | 2.12582 | 2.06761 |
| 0.0158 | 2.08424 | 2.09883 | 2.14215 | 2.08409 |
| 0.016 | 2.10077 | 2.11533 | 2.15852 | 2.10062 |
| 0.0162 | 2.11735 | 2.13188 | 2.17493 | 2.11721 |
| 0.0164 | 2.13398 | 2.14847 | 2.19138 | 2.13384 |
| 0.0166 | 2.15066 | 2.1651 | 2.20787 | 2.15051 |
| 0.0168 | 2.16738 | 2.18178 | 2.2244 | 2.16723 |
| 0.017 | 2.18415 | 2.1985 | 2.24097 | 2.184 |
| 0.0172 | 2.20096 | 2.21527 | 2.25758 | 2.20082 |
| 0.0174 | 2.21782 | 2.23208 | 2.27422 | 2.21767 |
| 0.0176 | 2.23472 | 2.24893 | 2.2909 | 2.23457 |
| 0.0178 | 2.25166 | 2.26582 | 2.30762 | 2.25152 |
| 0.018 | 2.26865 | 2.28275 | 2.32437 | 2.2685 |
| 0.0182 | 2.28567 | 2.29972 | 2.34115 | 2.28553 |
| 0.0184 | 2.30274 | 2.31673 | 2.35797 | 2.3026 |
| 0.0186 | 2.31985 | 2.33378 | 2.37483 | 2.31971 |
| 0.0188 | 2.337 | 2.35087 | 2.39171 | 2.33685 |
| 0.019 | 2.35418 | 2.368 | 2.40863 | 2.35404 |
| 0.0192 | 2.37141 | 2.38516 | 2.42558 | 2.37127 |
| 0.0194 | 2.38867 | 2.40236 | 2.44256 | 2.38853 |
| 0.0196 | 2.40597 | 2.41959 | 2.45958 | 2.40583 |
| 0.0198 | 2.42331 | 2.43686 | 2.47662 | 2.42317 |
| 0.02 | 2.44068 | 2.45416 | 2.49369 | 2.44054 |
| 0.0202 | 2.45809 | 2.4715 | 2.51079 | 2.45795 |
| 0.0204 | 2.47553 | 2.48887 | 2.52792 | 2.4754 |
| 0.0206 | 2.49301 | 2.50627 | 2.54508 | 2.49288 |
| 0.0208 | 2.51053 | 2.52371 | 2.56227 | 2.51039 |
| 0.021 | 2.52807 | 2.54118 | 2.57948 | 2.52794 |
| 0.0212 | 2.54565 | 2.55868 | 2.59672 | 2.54552 |
| 0.0214 | 2.56327 | 2.57621 | 2.61398 | 2.56313 |
| 0.0216 | 2.58091 | 2.59377 | 2.63127 | 2.58078 |
| 0.0218 | 2.59859 | 2.61136 | 2.64859 | 2.59846 |
| 0.022 | 2.61629 | 2.62898 | 2.66593 | 2.61616 |
| 0.0222 | 2.63403 | 2.64663 | 2.6833 | 2.6339 |
| 0.0224 | 2.6518 | 2.66431 | 2.70069 | 2.65167 |
| 0.0226 | 2.6696 | 2.68202 | 2.7181 | 2.66947 |
| 0.0228 | 2.68742 | 2.69975 | 2.73554 | 2.6873 |
| 0.023 | 2.70528 | 2.71751 | 2.753 | 2.70515 |
| 0.0232 | 2.72316 | 2.7353 | 2.77048 | 2.72304 |
| 0.0234 | 2.74108 | 2.75312 | 2.78798 | 2.74095 |
| 0.0236 | 2.75902 | 2.77096 | 2.80551 | 2.75889 |
| 0.0238 | 2.77698 | 2.78883 | 2.82306 | 2.77686 |
| 0.024 | 2.79498 | 2.80672 | 2.84062 | 2.79486 |
| 0.0242 | 2.813 | 2.82464 | 2.85821 | 2.81288 |
| 0.0244 | 2.83104 | 2.84258 | 2.87582 | 2.83093 |
| 0.0246 | 2.84912 | 2.86055 | 2.89345 | 2.849 |
| 0.0248 | 2.86721 | 2.87854 | 2.91109 | 2.8671 |
| 0.025 | 2.88533 | 2.89655 | 2.92876 | 2.88522 |
| 0.0252 | 2.90348 | 2.91459 | 2.94644 | 2.90337 |
| 0.0254 | 2.92165 | 2.93264 | 2.96415 | 2.92154 |
| 0.0256 | 2.93985 | 2.95073 | 2.98187 | 2.93974 |
| 0.0258 | 2.95806 | 2.96883 | 2.99961 | 2.95795 |
| 0.026 | 2.97631 | 2.98695 | 3.01736 | 2.9762 |
| 0.0262 | 2.99457 | 3.0051 | 3.03514 | 2.99446 |
| 0.0264 | 3.01286 | 3.02327 | 3.05293 | 3.01275 |
| 0.0266 | 3.03116 | 3.04146 | 3.07073 | 3.03106 |
| 0.0268 | 3.04949 | 3.05967 | 3.08856 | 3.04939 |
| 0.027 | 3.06785 | 3.07789 | 3.10639 | 3.06774 |
| 0.0272 | 3.08622 | 3.09614 | 3.12425 | 3.08612 |
| 0.0274 | 3.10461 | 3.11441 | 3.14212 | 3.10451 |
| 0.0276 | 3.12303 | 3.1327 | 3.16 | 3.12293 |
| 0.0278 | 3.14146 | 3.151 | 3.1779 | 3.14136 |
| 0.028 | 3.15992 | 3.16933 | 3.19581 | 3.15982 |
| 0.0282 | 3.17839 | 3.18767 | 3.21374 | 3.1783 |
| 0.0284 | 3.19689 | 3.20603 | 3.23168 | 3.19679 |
| 0.0286 | 3.2154 | 3.22441 | 3.24964 | 3.21531 |
| 0.0288 | 3.23393 | 3.24281 | 3.26761 | 3.23384 |
| 0.029 | 3.25248 | 3.26122 | 3.28559 | 3.25239 |
| 0.0292 | 3.27105 | 3.27965 | 3.30358 | 3.27096 |
| 0.0294 | 3.28964 | 3.2981 | 3.32159 | 3.28955 |
| 0.0296 | 3.30824 | 3.31656 | 3.33961 | 3.30815 |
| 0.0298 | 3.32686 | 3.33504 | 3.35764 | 3.32678 |
| 0.03 | 3.3455 | 3.35354 | 3.37569 | 3.34542 |
| 0.0302 | 3.36416 | 3.37205 | 3.39374 | 3.36408 |
| 0.0304 | 3.38283 | 3.39058 | 3.41181 | 3.38275 |
| 0.0306 | 3.40152 | 3.40912 | 3.42989 | 3.40145 |
| 0.0308 | 3.42023 | 3.42768 | 3.44798 | 3.42015 |
| 0.031 | 3.43895 | 3.44625 | 3.46608 | 3.43888 |
| 0.0312 | 3.45769 | 3.46484 | 3.48419 | 3.45762 |
| 0.0314 | 3.47645 | 3.48344 | 3.50232 | 3.47637 |
| 0.0316 | 3.49522 | 3.50206 | 3.52045 | 3.49515 |
| 0.0318 | 3.514 | 3.52069 | 3.53859 | 3.51393 |
| 0.032 | 3.5328 | 3.53933 | 3.55674 | 3.53274 |
| 0.0322 | 3.55162 | 3.55799 | 3.57491 | 3.55155 |
| 0.0324 | 3.57045 | 3.57666 | 3.59308 | 3.57038 |
| 0.0326 | 3.58929 | 3.59534 | 3.61126 | 3.58923 |
| 0.0328 | 3.60815 | 3.61404 | 3.62945 | 3.60809 |
| 0.033 | 3.62703 | 3.63275 | 3.64765 | 3.62697 |
| 0.0332 | 3.64591 | 3.65147 | 3.66586 | 3.64586 |
| 0.0334 | 3.66482 | 3.67021 | 3.68408 | 3.66476 |
| 0.0336 | 3.68373 | 3.68896 | 3.7023 | 3.68368 |
| 0.0338 | 3.70266 | 3.70772 | 3.72053 | 3.7026 |
| 0.034 | 3.7216 | 3.72649 | 3.73878 | 3.72155 |
| 0.0342 | 3.74055 | 3.74527 | 3.75703 | 3.7405 |
| 0.0344 | 3.75952 | 3.76407 | 3.77528 | 3.75947 |
| 0.0346 | 3.7785 | 3.78288 | 3.79355 | 3.77845 |
| 0.0348 | 3.79749 | 3.80169 | 3.81182 | 3.79745 |
| 0.035 | 3.8165 | 3.82052 | 3.8301 | 3.81646 |
| 0.0352 | 3.83552 | 3.83936 | 3.84839 | 3.83547 |
| 0.0354 | 3.85454 | 3.85821 | 3.86669 | 3.85451 |
| 0.0356 | 3.87359 | 3.87708 | 3.88499 | 3.87355 |
| 0.0358 | 3.89264 | 3.89595 | 3.9033 | 3.8926 |
| 0.036 | 3.9117 | 3.91483 | 3.92161 | 3.91167 |
| 0.0362 | 3.93078 | 3.93372 | 3.93993 | 3.93075 |
| 0.0364 | 3.94987 | 3.95263 | 3.95826 | 3.94984 |
| 0.0366 | 3.96896 | 3.97154 | 3.9766 | 3.96894 |
| 0.0368 | 3.98807 | 3.99046 | 3.99494 | 3.98805 |
| 0.037 | 4.00719 | 4.00939 | 4.01328 | 4.00717 |
| 0.0372 | 4.02633 | 4.02834 | 4.03164 | 4.0263 |
| 0.0374 | 4.04547 | 4.04729 | 4.04999 | 4.04545 |
| 0.0376 | 4.06462 | 4.06625 | 4.06836 | 4.0646 |
| 0.0378 | 4.08378 | 4.08522 | 4.08673 | 4.08377 |
| 0.038 | 4.10296 | 4.1042 | 4.1051 | 4.10294 |
| 0.0382 | 4.12214 | 4.12318 | 4.12348 | 4.12213 |
| 0.0384 | 4.14133 | 4.14218 | 4.14187 | 4.14132 |
| 0.0386 | 4.16054 | 4.16119 | 4.16026 | 4.16053 |
| 0.0388 | 4.17975 | 4.1802 | 4.17865 | 4.17974 |
| 0.039 | 4.19897 | 4.19922 | 4.19705 | 4.19897 |
| 0.0392 | 4.2182 | 4.21825 | 4.21545 | 4.2182 |
| 0.0394 | 4.23744 | 4.23729 | 4.23386 | 4.23744 |
| 0.0396 | 4.25669 | 4.25634 | 4.25228 | 4.2567 |
| 0.0398 | 4.27595 | 4.27539 | 4.27069 | 4.27596 |
| 0.04 | 4.29522 | 4.29446 | 4.28912 | 4.29523 |
| 0.0402 | 4.3145 | 4.31353 | 4.30754 | 4.31451 |
| 0.0404 | 4.33379 | 4.3326 | 4.32597 | 4.3338 |
| 0.0406 | 4.35308 | 4.35169 | 4.34441 | 4.35309 |
| 0.0408 | 4.37239 | 4.37078 | 4.36285 | 4.3724 |
| 0.041 | 4.3917 | 4.38988 | 4.38129 | 4.39171 |
| 0.0412 | 4.41102 | 4.40899 | 4.39973 | 4.41104 |
| 0.0414 | 4.43035 | 4.42811 | 4.41818 | 4.43037 |
| 0.0416 | 4.44969 | 4.44723 | 4.43664 | 4.44971 |
| 0.0418 | 4.46903 | 4.46636 | 4.45509 | 4.46906 |
| 0.042 | 4.48839 | 4.48549 | 4.47355 | 4.48841 |
| 0.0422 | 4.50775 | 4.50464 | 4.49201 | 4.50778 |
| 0.0424 | 4.52712 | 4.52378 | 4.51048 | 4.52715 |
| 0.0426 | 4.5465 | 4.54294 | 4.52895 | 4.54653 |
| 0.0428 | 4.56588 | 4.5621 | 4.54742 | 4.56592 |
| 0.043 | 4.58527 | 4.58127 | 4.5659 | 4.58531 |
| 0.0432 | 4.60467 | 4.60045 | 4.58437 | 4.60471 |
| 0.0434 | 4.62408 | 4.61963 | 4.60285 | 4.62412 |
| 0.0436 | 4.6435 | 4.63882 | 4.62134 | 4.64354 |
| 0.0438 | 4.66292 | 4.65801 | 4.63982 | 4.66297 |
| 0.044 | 4.68235 | 4.67721 | 4.65831 | 4.6824 |
| 0.0442 | 4.70178 | 4.69642 | 4.6768 | 4.70184 |
| 0.0444 | 4.72123 | 4.71563 | 4.69529 | 4.72128 |
| 0.0446 | 4.74068 | 4.73484 | 4.71379 | 4.74073 |
| 0.0448 | 4.76014 | 4.75407 | 4.73229 | 4.76019 |
| 0.045 | 4.7796 | 4.7733 | 4.75078 | 4.77966 |
| 0.0452 | 4.79907 | 4.79253 | 4.76929 | 4.79913 |
| 0.0454 | 4.81855 | 4.81177 | 4.78779 | 4.81861 |
| 0.0456 | 4.83803 | 4.83102 | 4.8063 | 4.8381 |
| 0.0458 | 4.85752 | 4.85027 | 4.8248 | 4.85759 |
| 0.046 | 4.87702 | 4.86952 | 4.84331 | 4.87709 |
| 0.0462 | 4.89652 | 4.88878 | 4.86182 | 4.8966 |
| 0.0464 | 4.91603 | 4.90805 | 4.88033 | 4.91611 |
| 0.0466 | 4.93555 | 4.92732 | 4.89885 | 4.93563 |
| 0.0468 | 4.95507 | 4.9466 | 4.91736 | 4.95515 |
| 0.047 | 4.9746 | 4.96588 | 4.93588 | 4.97469 |
| 0.0472 | 4.99413 | 4.98516 | 4.9544 | 4.99422 |
| 0.0474 | 5.01367 | 5.00446 | 4.97292 | 5.01376 |
| 0.0476 | 5.03322 | 5.02375 | 4.99144 | 5.03331 |
| 0.0478 | 5.05277 | 5.04305 | 5.00996 | 5.05287 |
| 0.048 | 5.07233 | 5.06236 | 5.02849 | 5.07243 |
| 0.0482 | 5.09189 | 5.08167 | 5.04701 | 5.09199 |
| 0.0484 | 5.11146 | 5.10098 | 5.06554 | 5.11157 |
| 0.0486 | 5.13104 | 5.1203 | 5.08406 | 5.13114 |
| 0.0488 | 5.15062 | 5.13962 | 5.10259 | 5.15073 |
| 0.049 | 5.1702 | 5.15895 | 5.12112 | 5.17031 |
| 0.0492 | 5.18979 | 5.17828 | 5.13965 | 5.18991 |
| 0.0494 | 5.20939 | 5.19762 | 5.15818 | 5.20951 |
| 0.0496 | 5.22899 | 5.21696 | 5.17671 | 5.22911 |
| 0.0498 | 5.2486 | 5.2363 | 5.19524 | 5.24872 |
| 0.05 | 5.26821 | 5.25565 | 5.21377 | 5.26834 |
