# Supplementary material for: Analysis of sinusoidal post-buckling deformation of horizontal coiled tubing with initial residual bending
Source: PLoS One. 2024 May 14;19(5):e0301610. doi: 10.1371/journal.pone.0301610 (PMC11093391; doi:10.1371/journal.pone.0301610)
Supplement: S1 File — (ZIP) [file pone.0301610.s001.zip › The values used to build graphs - Fig 3.docx]

## The values used to build graphs

The minimal data set of the original data for plotting curves in Fig 3 is as follows:

| x-axis | ‾*L*_0_ = 100 | ‾*L*_0_ = 200 | ‾*L*_0_ = 500 | ‾*L*_0_ = 1000 |
| --- | --- | --- | --- | --- |
| 0 | 0.03143 | 0.01571 | 0.00628 | 0.00314 |
| 0.001 | 0.26888 | 0.187 | 0.11711 | 0.08254 |
| 0.002 | 0.38859 | 0.26735 | 0.16634 | 0.11698 |
| 0.003 | 0.48747 | 0.33141 | 0.20471 | 0.14362 |
| 0.004 | 0.57678 | 0.38743 | 0.23755 | 0.16625 |
| 0.005 | 0.66077 | 0.43858 | 0.26692 | 0.18633 |
| 0.006 | 0.74157 | 0.48645 | 0.29387 | 0.20463 |
| 0.007 | 0.82039 | 0.53201 | 0.31902 | 0.22159 |
| 0.008 | 0.89797 | 0.57584 | 0.34276 | 0.23748 |
| 0.009 | 0.9748 | 0.61836 | 0.36539 | 0.25253 |
| 0.01 | 1.05121 | 0.65988 | 0.3871 | 0.26686 |
| 0.011 | 1.12741 | 0.7006 | 0.40805 | 0.28059 |
| 0.012 | 1.20358 | 0.7407 | 0.42834 | 0.29381 |
| 0.013 | 1.2798 | 0.78031 | 0.44808 | 0.30658 |
| 0.014 | 1.35616 | 0.81954 | 0.46733 | 0.31896 |
| 0.015 | 1.4327 | 0.85845 | 0.48617 | 0.33099 |
| 0.016 | 1.50946 | 0.89713 | 0.50463 | 0.34271 |
| 0.017 | 1.58645 | 0.93562 | 0.52277 | 0.35415 |
| 0.018 | 1.66368 | 0.97396 | 0.54062 | 0.36534 |
| 0.019 | 1.74115 | 1.0122 | 0.55821 | 0.3763 |
| 0.02 | 1.81887 | 1.05037 | 0.57557 | 0.38706 |
| 0.021 | 1.89683 | 1.08849 | 0.59272 | 0.39762 |
| 0.022 | 1.97502 | 1.12658 | 0.60969 | 0.408 |
| 0.023 | 2.05344 | 1.16466 | 0.62648 | 0.41822 |
| 0.024 | 2.13207 | 1.20274 | 0.64312 | 0.4283 |
| 0.025 | 2.2109 | 1.24084 | 0.65962 | 0.43823 |
| 0.026 | 2.28993 | 1.27897 | 0.676 | 0.44803 |
| 0.027 | 2.36914 | 1.31713 | 0.69226 | 0.45772 |
| 0.028 | 2.44853 | 1.35532 | 0.70842 | 0.46729 |
| 0.029 | 2.52809 | 1.39357 | 0.72448 | 0.47676 |
| 0.03 | 2.6078 | 1.43186 | 0.74046 | 0.48613 |
| 0.031 | 2.68766 | 1.47021 | 0.75636 | 0.4954 |
| 0.032 | 2.76766 | 1.50862 | 0.77218 | 0.50459 |
| 0.033 | 2.84779 | 1.54708 | 0.78795 | 0.5137 |
| 0.034 | 2.92805 | 1.5856 | 0.80365 | 0.52273 |
| 0.035 | 3.00842 | 1.62419 | 0.8193 | 0.53169 |
| 0.036 | 3.08891 | 1.66283 | 0.8349 | 0.54058 |
| 0.037 | 3.1695 | 1.70154 | 0.85045 | 0.54941 |
| 0.038 | 3.25019 | 1.7403 | 0.86597 | 0.55818 |
| 0.039 | 3.33097 | 1.77913 | 0.88145 | 0.56688 |
| 0.04 | 3.41185 | 1.81802 | 0.89689 | 0.57553 |
| 0.041 | 3.4928 | 1.85697 | 0.91231 | 0.58413 |
| 0.042 | 3.57384 | 1.89598 | 0.9277 | 0.59269 |
| 0.043 | 3.65495 | 1.93504 | 0.94306 | 0.60119 |
| 0.044 | 3.73614 | 1.97417 | 0.9584 | 0.60965 |
| 0.045 | 3.81739 | 2.01335 | 0.97373 | 0.61807 |
| 0.046 | 3.89871 | 2.05258 | 0.98904 | 0.62644 |
| 0.047 | 3.98009 | 2.09187 | 1.00433 | 0.63478 |
| 0.048 | 4.06153 | 2.13121 | 1.01961 | 0.64309 |
| 0.049 | 4.14302 | 2.1706 | 1.03488 | 0.65135 |
| 0.05 | 4.22457 | 2.21004 | 1.05014 | 0.65959 |
| 0.051 | 4.30616 | 2.24953 | 1.06539 | 0.66779 |
| 0.052 | 4.38781 | 2.28906 | 1.08064 | 0.67596 |
| 0.053 | 4.4695 | 2.32865 | 1.09588 | 0.68411 |
| 0.054 | 4.55123 | 2.36828 | 1.11111 | 0.69223 |
| 0.055 | 4.63301 | 2.40795 | 1.12635 | 0.70032 |
| 0.056 | 4.71482 | 2.44766 | 1.14158 | 0.70838 |
| 0.057 | 4.79668 | 2.48742 | 1.15681 | 0.71643 |
| 0.058 | 4.87857 | 2.52722 | 1.17204 | 0.72445 |
| 0.059 | 4.9605 | 2.56705 | 1.18728 | 0.73245 |
| 0.06 | 5.04246 | 2.60693 | 1.20251 | 0.74042 |
| 0.061 | 5.12445 | 2.64684 | 1.21775 | 0.74838 |
| 0.062 | 5.20647 | 2.68679 | 1.23299 | 0.75632 |
| 0.063 | 5.28852 | 2.72677 | 1.24823 | 0.76425 |
| 0.064 | 5.37061 | 2.76678 | 1.26348 | 0.77215 |
| 0.065 | 5.45272 | 2.80683 | 1.27873 | 0.78004 |
| 0.066 | 5.53485 | 2.84691 | 1.29399 | 0.78791 |
| 0.067 | 5.61701 | 2.88703 | 1.30926 | 0.79577 |
| 0.068 | 5.6992 | 2.92717 | 1.32453 | 0.80361 |
| 0.069 | 5.78141 | 2.96734 | 1.33981 | 0.81145 |
| 0.07 | 5.86364 | 3.00754 | 1.35509 | 0.81926 |
| 0.071 | 5.94589 | 3.04777 | 1.37038 | 0.82707 |
| 0.072 | 6.02817 | 3.08803 | 1.38568 | 0.83486 |
| 0.073 | 6.11046 | 3.12831 | 1.40099 | 0.84265 |
| 0.074 | 6.19278 | 3.16862 | 1.4163 | 0.85042 |
| 0.075 | 6.27511 | 3.20895 | 1.43163 | 0.85818 |
| 0.076 | 6.35747 | 3.24931 | 1.44696 | 0.86593 |
| 0.077 | 6.43984 | 3.28969 | 1.4623 | 0.87368 |
| 0.078 | 6.52223 | 3.33009 | 1.47765 | 0.88141 |
| 0.079 | 6.60463 | 3.37052 | 1.49301 | 0.88914 |
| 0.08 | 6.68705 | 3.41096 | 1.50838 | 0.89686 |
| 0.081 | 6.76949 | 3.45143 | 1.52376 | 0.90457 |
| 0.082 | 6.85194 | 3.49192 | 1.53915 | 0.91227 |
| 0.083 | 6.93441 | 3.53243 | 1.55454 | 0.91997 |
| 0.084 | 7.01689 | 3.57296 | 1.56995 | 0.92766 |
| 0.085 | 7.09938 | 3.6135 | 1.58537 | 0.93535 |
| 0.086 | 7.18189 | 3.65407 | 1.60079 | 0.94303 |
| 0.087 | 7.26441 | 3.69465 | 1.61623 | 0.9507 |
| 0.088 | 7.34695 | 3.73525 | 1.63167 | 0.95837 |
| 0.089 | 7.4295 | 3.77587 | 1.64713 | 0.96603 |
| 0.09 | 7.51205 | 3.8165 | 1.66259 | 0.97369 |
| 0.091 | 7.59463 | 3.85715 | 1.67807 | 0.98135 |
| 0.092 | 7.67721 | 3.89782 | 1.69355 | 0.989 |
| 0.093 | 7.7598 | 3.9385 | 1.70905 | 0.99665 |
| 0.094 | 7.84241 | 3.9792 | 1.72455 | 1.0043 |
| 0.095 | 7.92502 | 4.01991 | 1.74007 | 1.01194 |
| 0.096 | 8.00765 | 4.06064 | 1.75559 | 1.01958 |
| 0.097 | 8.09028 | 4.10137 | 1.77112 | 1.02721 |
| 0.098 | 8.17293 | 4.14213 | 1.78667 | 1.03485 |
| 0.099 | 8.25558 | 4.18289 | 1.80222 | 1.04248 |
| 0.1 | 8.33824 | 4.22367 | 1.81778 | 1.05011 |
| 0.101 | 8.42092 | 4.26447 | 1.83336 | 1.05773 |
| 0.102 | 8.5036 | 4.30527 | 1.84894 | 1.06536 |
| 0.103 | 8.58629 | 4.34609 | 1.86453 | 1.07298 |
| 0.104 | 8.66899 | 4.38691 | 1.88013 | 1.0806 |
| 0.105 | 8.7517 | 4.42775 | 1.89574 | 1.08822 |
| 0.106 | 8.83441 | 4.4686 | 1.91136 | 1.09584 |
| 0.107 | 8.91714 | 4.50946 | 1.92699 | 1.10346 |
| 0.108 | 8.99987 | 4.55034 | 1.94262 | 1.11108 |
| 0.109 | 9.08261 | 4.59122 | 1.95827 | 1.1187 |
| 0.11 | 9.16535 | 4.63211 | 1.97393 | 1.12631 |
| 0.111 | 9.24811 | 4.67302 | 1.98959 | 1.13393 |
| 0.112 | 9.33087 | 4.71393 | 2.00527 | 1.14155 |
| 0.113 | 9.41364 | 4.75485 | 2.02095 | 1.14916 |
| 0.114 | 9.49641 | 4.79578 | 2.03664 | 1.15678 |
| 0.115 | 9.5792 | 4.83672 | 2.05234 | 1.16439 |
| 0.116 | 9.66199 | 4.87767 | 2.06805 | 1.17201 |
| 0.117 | 9.74478 | 4.91863 | 2.08376 | 1.17963 |
| 0.118 | 9.82758 | 4.9596 | 2.09949 | 1.18724 |
| 0.119 | 9.91039 | 5.00058 | 2.11522 | 1.19486 |
| 0.12 | 9.99321 | 5.04156 | 2.13097 | 1.20248 |
| 0.121 | 10.07603 | 5.08255 | 2.14672 | 1.2101 |
| 0.122 | 10.15885 | 5.12355 | 2.16247 | 1.21771 |
| 0.123 | 10.24168 | 5.16456 | 2.17824 | 1.22533 |
| 0.124 | 10.32452 | 5.20557 | 2.19401 | 1.23295 |
| 0.125 | 10.40737 | 5.2466 | 2.2098 | 1.24058 |
| 0.126 | 10.49022 | 5.28763 | 2.22559 | 1.2482 |
| 0.127 | 10.57307 | 5.32867 | 2.24138 | 1.25582 |
| 0.128 | 10.65593 | 5.36971 | 2.25719 | 1.26345 |
| 0.129 | 10.7388 | 5.41076 | 2.273 | 1.27107 |
| 0.13 | 10.82167 | 5.45182 | 2.28882 | 1.2787 |
| 0.131 | 10.90454 | 5.49288 | 2.30465 | 1.28633 |
| 0.132 | 10.98742 | 5.53395 | 2.32049 | 1.29396 |
| 0.133 | 11.07031 | 5.57503 | 2.33633 | 1.30159 |
| 0.134 | 11.1532 | 5.61611 | 2.35218 | 1.30922 |
| 0.135 | 11.2361 | 5.6572 | 2.36803 | 1.31686 |
| 0.136 | 11.319 | 5.6983 | 2.3839 | 1.32449 |
| 0.137 | 11.4019 | 5.7394 | 2.39977 | 1.33213 |
| 0.138 | 11.48481 | 5.78051 | 2.41565 | 1.33977 |
| 0.139 | 11.56773 | 5.82162 | 2.43153 | 1.34741 |
| 0.14 | 11.65065 | 5.86274 | 2.44742 | 1.35506 |
| 0.141 | 11.73357 | 5.90387 | 2.46332 | 1.3627 |
| 0.142 | 11.8165 | 5.94499 | 2.47922 | 1.37035 |
| 0.143 | 11.89943 | 5.98613 | 2.49513 | 1.378 |
| 0.144 | 11.98237 | 6.02727 | 2.51105 | 1.38565 |
| 0.145 | 12.06531 | 6.06841 | 2.52697 | 1.3933 |
| 0.146 | 12.14826 | 6.10957 | 2.5429 | 1.40096 |
| 0.147 | 12.23121 | 6.15072 | 2.55884 | 1.40861 |
| 0.148 | 12.31416 | 6.19188 | 2.57478 | 1.41627 |
| 0.149 | 12.39712 | 6.23305 | 2.59073 | 1.42393 |
| 0.15 | 12.48008 | 6.27421 | 2.60668 | 1.4316 |
| 0.151 | 12.56305 | 6.31539 | 2.62264 | 1.43926 |
| 0.152 | 12.64602 | 6.35657 | 2.63861 | 1.44693 |
| 0.153 | 12.72899 | 6.39775 | 2.65458 | 1.4546 |
| 0.154 | 12.81197 | 6.43894 | 2.67056 | 1.46227 |
| 0.155 | 12.89495 | 6.48013 | 2.68654 | 1.46994 |
| 0.156 | 12.97793 | 6.52133 | 2.70253 | 1.47762 |
| 0.157 | 13.06092 | 6.56253 | 2.71852 | 1.4853 |
| 0.158 | 13.14391 | 6.60373 | 2.73452 | 1.49298 |
| 0.159 | 13.22691 | 6.64494 | 2.75053 | 1.50066 |
| 0.16 | 13.30991 | 6.68615 | 2.76654 | 1.50835 |
| 0.161 | 13.39291 | 6.72737 | 2.78255 | 1.51604 |
| 0.162 | 13.47592 | 6.76859 | 2.79858 | 1.52373 |
| 0.163 | 13.55893 | 6.80981 | 2.8146 | 1.53142 |
| 0.164 | 13.64194 | 6.85104 | 2.83063 | 1.53911 |
| 0.165 | 13.72496 | 6.89227 | 2.84667 | 1.54681 |
| 0.166 | 13.80798 | 6.93351 | 2.86271 | 1.55451 |
| 0.167 | 13.89101 | 6.97475 | 2.87876 | 1.56221 |
| 0.168 | 13.97403 | 7.01599 | 2.89481 | 1.56992 |
| 0.169 | 14.05706 | 7.05723 | 2.91086 | 1.57762 |
| 0.17 | 14.1401 | 7.09848 | 2.92692 | 1.58533 |
| 0.171 | 14.22314 | 7.13973 | 2.94299 | 1.59304 |
| 0.172 | 14.30618 | 7.18099 | 2.95906 | 1.60076 |
| 0.173 | 14.38922 | 7.22225 | 2.97513 | 1.60848 |
| 0.174 | 14.47227 | 7.26351 | 2.99121 | 1.61619 |
| 0.175 | 14.55532 | 7.30478 | 3.0073 | 1.62392 |
| 0.176 | 14.63837 | 7.34605 | 3.02339 | 1.63164 |
| 0.177 | 14.72143 | 7.38732 | 3.03948 | 1.63937 |
| 0.178 | 14.80449 | 7.42859 | 3.05557 | 1.6471 |
| 0.179 | 14.88755 | 7.46987 | 3.07168 | 1.65483 |
| 0.18 | 14.97061 | 7.51115 | 3.08778 | 1.66256 |
| 0.181 | 15.05368 | 7.55244 | 3.10389 | 1.6703 |
| 0.182 | 15.13675 | 7.59372 | 3.12 | 1.67803 |
| 0.183 | 15.21983 | 7.63501 | 3.13612 | 1.68578 |
| 0.184 | 15.30291 | 7.67631 | 3.15224 | 1.69352 |
| 0.185 | 15.38599 | 7.7176 | 3.16837 | 1.70127 |
| 0.186 | 15.46907 | 7.7589 | 3.1845 | 1.70901 |
| 0.187 | 15.55216 | 7.8002 | 3.20063 | 1.71677 |
| 0.188 | 15.63524 | 7.8415 | 3.21677 | 1.72452 |
| 0.189 | 15.71834 | 7.88281 | 3.23291 | 1.73227 |
| 0.19 | 15.80143 | 7.92412 | 3.24906 | 1.74003 |
| 0.191 | 15.88453 | 7.96543 | 3.26521 | 1.74779 |
| 0.192 | 15.96763 | 8.00674 | 3.28136 | 1.75556 |
| 0.193 | 16.05073 | 8.04806 | 3.29752 | 1.76332 |
| 0.194 | 16.13384 | 8.08938 | 3.31368 | 1.77109 |
| 0.195 | 16.21695 | 8.1307 | 3.32984 | 1.77886 |
| 0.196 | 16.30006 | 8.17202 | 3.34601 | 1.78663 |
| 0.197 | 16.38317 | 8.21335 | 3.36218 | 1.79441 |
| 0.198 | 16.46629 | 8.25468 | 3.37836 | 1.80219 |
| 0.199 | 16.54941 | 8.29601 | 3.39453 | 1.80997 |
| 0.2 | 16.63253 | 8.33734 | 3.41071 | 1.81775 |
