# Supplementary material for: Analysis of sinusoidal post-buckling deformation of horizontal coiled tubing with initial residual bending
Source: PLoS One. 2024 May 14;19(5):e0301610. doi: 10.1371/journal.pone.0301610 (PMC11093391; doi:10.1371/journal.pone.0301610)
Supplement: S1 File — (ZIP) [file pone.0301610.s001.zip › The values used to build graphs - Fig 2.docx]

## The values used to build graphs

The minimal data set of the original data for plotting curves in Fig 2 is as follows:

| x-axis | Wu (1995) | m=10 | m=20 | m=30 |
| --- | --- | --- | --- | --- |
| 0 | 0 | 0.15854 | 0.32599 | 0.51197 |
| 0.001 | 0.26684 | 0.3146 | 0.43408 | 0.59956 |
| 0.002 | 0.38704 | 0.42473 | 0.52795 | 0.68255 |
| 0.003 | 0.48611 | 0.51953 | 0.61454 | 0.76272 |
| 0.004 | 0.57552 | 0.60664 | 0.69693 | 0.84115 |
| 0.005 | 0.65958 | 0.68934 | 0.77673 | 0.91849 |
| 0.006 | 0.74041 | 0.76934 | 0.85492 | 0.99517 |
| 0.007 | 0.81925 | 0.84765 | 0.93212 | 1.07151 |
| 0.008 | 0.89685 | 0.92492 | 1.00873 | 1.14769 |
| 0.009 | 0.97368 | 1.00157 | 1.08502 | 1.22385 |
| 0.01 | 1.05009 | 1.07789 | 1.16119 | 1.30011 |
| 0.011 | 1.1263 | 1.15407 | 1.23737 | 1.37651 |
| 0.012 | 1.20247 | 1.23024 | 1.31365 | 1.4531 |
| 0.013 | 1.27869 | 1.3065 | 1.39008 | 1.52991 |
| 0.014 | 1.35505 | 1.38292 | 1.46671 | 1.60696 |
| 0.015 | 1.43158 | 1.45954 | 1.54357 | 1.68426 |
| 0.016 | 1.50834 | 1.53637 | 1.62066 | 1.7618 |
| 0.017 | 1.58532 | 1.61344 | 1.698 | 1.83958 |
| 0.018 | 1.66255 | 1.69076 | 1.77559 | 1.9176 |
| 0.019 | 1.74002 | 1.76832 | 1.85341 | 1.99585 |
| 0.02 | 1.81774 | 1.84612 | 1.93147 | 2.07432 |
| 0.021 | 1.89569 | 1.92417 | 2.00976 | 2.153 |
| 0.022 | 1.97388 | 2.00244 | 2.08827 | 2.23188 |
| 0.023 | 2.05229 | 2.08093 | 2.16699 | 2.31096 |
| 0.024 | 2.13092 | 2.15963 | 2.24591 | 2.39022 |
| 0.025 | 2.20975 | 2.23853 | 2.32502 | 2.46965 |
| 0.026 | 2.28878 | 2.31763 | 2.40432 | 2.54924 |
| 0.027 | 2.36799 | 2.3969 | 2.48378 | 2.62899 |
| 0.028 | 2.44737 | 2.47635 | 2.5634 | 2.70889 |
| 0.029 | 2.52693 | 2.55596 | 2.64318 | 2.78892 |
| 0.03 | 2.60664 | 2.63573 | 2.7231 | 2.86908 |
| 0.031 | 2.68649 | 2.71563 | 2.80316 | 2.94937 |
| 0.032 | 2.76649 | 2.79568 | 2.88335 | 3.02977 |
| 0.033 | 2.84662 | 2.87586 | 2.96366 | 3.11028 |
| 0.034 | 2.92688 | 2.95616 | 3.04408 | 3.1909 |
| 0.035 | 3.00725 | 3.03657 | 3.12461 | 3.27161 |
| 0.036 | 3.08773 | 3.11709 | 3.20525 | 3.35242 |
| 0.037 | 3.16832 | 3.19772 | 3.28598 | 3.43331 |
| 0.038 | 3.24901 | 3.27844 | 3.3668 | 3.51429 |
| 0.039 | 3.3298 | 3.35926 | 3.44771 | 3.59534 |
| 0.04 | 3.41067 | 3.44016 | 3.5287 | 3.67647 |
| 0.041 | 3.49162 | 3.52115 | 3.60977 | 3.75767 |
| 0.042 | 3.57266 | 3.60221 | 3.69092 | 3.83894 |
| 0.043 | 3.65377 | 3.68335 | 3.77213 | 3.92027 |
| 0.044 | 3.73496 | 3.76456 | 3.85341 | 4.00166 |
| 0.045 | 3.81621 | 3.84583 | 3.93476 | 4.08311 |
| 0.046 | 3.89752 | 3.92717 | 4.01616 | 4.16462 |
| 0.047 | 3.9789 | 4.00857 | 4.09762 | 4.24618 |
| 0.048 | 4.06034 | 4.09003 | 4.17914 | 4.32778 |
| 0.049 | 4.14183 | 4.17154 | 4.2607 | 4.40944 |
| 0.05 | 4.22338 | 4.2531 | 4.34232 | 4.49114 |
| 0.051 | 4.30497 | 4.33472 | 4.42398 | 4.57288 |
| 0.052 | 4.38662 | 4.41638 | 4.50569 | 4.65466 |
| 0.053 | 4.46831 | 4.49808 | 4.58745 | 4.73649 |
| 0.054 | 4.55004 | 4.57983 | 4.66924 | 4.81835 |
| 0.055 | 4.63181 | 4.66162 | 4.75107 | 4.90025 |
| 0.056 | 4.71363 | 4.74345 | 4.83294 | 4.98218 |
| 0.057 | 4.79548 | 4.82532 | 4.91485 | 5.06415 |
| 0.058 | 4.87738 | 4.90722 | 4.99679 | 5.14614 |
| 0.059 | 4.9593 | 4.98916 | 5.07876 | 5.22817 |
| 0.06 | 5.04126 | 5.07113 | 5.16077 | 5.31023 |
| 0.061 | 5.12325 | 5.15313 | 5.2428 | 5.39232 |
| 0.062 | 5.20528 | 5.23517 | 5.32487 | 5.47443 |
| 0.063 | 5.28733 | 5.31723 | 5.40696 | 5.55657 |
| 0.064 | 5.36941 | 5.39932 | 5.48908 | 5.63874 |
| 0.065 | 5.45152 | 5.48144 | 5.57122 | 5.72093 |
| 0.066 | 5.53365 | 5.56359 | 5.6534 | 5.80314 |
| 0.067 | 5.61582 | 5.64575 | 5.73559 | 5.88537 |
| 0.068 | 5.698 | 5.72795 | 5.81781 | 5.96763 |
| 0.069 | 5.78021 | 5.81017 | 5.90005 | 6.04991 |
| 0.07 | 5.86244 | 5.8924 | 5.98231 | 6.13221 |
| 0.071 | 5.9447 | 5.97467 | 6.06459 | 6.21452 |
| 0.072 | 6.02697 | 6.05695 | 6.1469 | 6.29686 |
| 0.073 | 6.10927 | 6.13925 | 6.22922 | 6.37922 |
| 0.074 | 6.19158 | 6.22157 | 6.31156 | 6.46159 |
| 0.075 | 6.27391 | 6.30391 | 6.39392 | 6.54398 |
| 0.076 | 6.35627 | 6.38627 | 6.4763 | 6.62639 |
| 0.077 | 6.43864 | 6.46865 | 6.55869 | 6.70881 |
| 0.078 | 6.52103 | 6.55104 | 6.6411 | 6.79125 |
| 0.079 | 6.60343 | 6.63345 | 6.72353 | 6.8737 |
| 0.08 | 6.68585 | 6.71588 | 6.80597 | 6.95617 |
| 0.081 | 6.76829 | 6.79832 | 6.88843 | 7.03865 |
| 0.082 | 6.85074 | 6.88078 | 6.9709 | 7.12115 |
| 0.083 | 6.93321 | 6.96325 | 7.05339 | 7.20365 |
| 0.084 | 7.01569 | 7.04574 | 7.13589 | 7.28618 |
| 0.085 | 7.09818 | 7.12823 | 7.2184 | 7.36871 |
| 0.086 | 7.18069 | 7.21075 | 7.30093 | 7.45126 |
| 0.087 | 7.26321 | 7.29327 | 7.38347 | 7.53382 |
| 0.088 | 7.34575 | 7.37581 | 7.46602 | 7.61639 |
| 0.089 | 7.42829 | 7.45836 | 7.54858 | 7.69897 |
| 0.09 | 7.51085 | 7.54093 | 7.63116 | 7.78157 |
| 0.091 | 7.59342 | 7.6235 | 7.71374 | 7.86417 |
| 0.092 | 7.676 | 7.70609 | 7.79634 | 7.94679 |
| 0.093 | 7.7586 | 7.78868 | 7.87895 | 8.02941 |
| 0.094 | 7.8412 | 7.87129 | 7.96157 | 8.11205 |
| 0.095 | 7.92382 | 7.95391 | 8.04419 | 8.19469 |
| 0.096 | 8.00644 | 8.03654 | 8.12683 | 8.27734 |
| 0.097 | 8.08908 | 8.11918 | 8.20948 | 8.36001 |
| 0.098 | 8.17172 | 8.20182 | 8.29214 | 8.44268 |
| 0.099 | 8.25438 | 8.28448 | 8.3748 | 8.52536 |
| 0.1 | 8.33704 | 8.36715 | 8.45748 | 8.60805 |
| 0.101 | 8.41971 | 8.44982 | 8.54017 | 8.69075 |
| 0.102 | 8.50239 | 8.53251 | 8.62286 | 8.77346 |
| 0.103 | 8.58509 | 8.6152 | 8.70556 | 8.85617 |
| 0.104 | 8.66778 | 8.6979 | 8.78827 | 8.9389 |
| 0.105 | 8.75049 | 8.78062 | 8.87099 | 9.02163 |
| 0.106 | 8.83321 | 8.86333 | 8.95372 | 9.10437 |
| 0.107 | 8.91593 | 8.94606 | 9.03645 | 9.18711 |
| 0.108 | 8.99866 | 9.02879 | 9.11919 | 9.26987 |
| 0.109 | 9.0814 | 9.11154 | 9.20194 | 9.35263 |
| 0.11 | 9.16415 | 9.19428 | 9.2847 | 9.4354 |
| 0.111 | 9.2469 | 9.27704 | 9.36746 | 9.51817 |
| 0.112 | 9.32966 | 9.3598 | 9.45023 | 9.60095 |
| 0.113 | 9.41243 | 9.44258 | 9.53301 | 9.68374 |
| 0.114 | 9.49521 | 9.52535 | 9.61579 | 9.76653 |
| 0.115 | 9.57799 | 9.60814 | 9.69858 | 9.84933 |
| 0.116 | 9.66078 | 9.69093 | 9.78138 | 9.93214 |
| 0.117 | 9.74357 | 9.77373 | 9.86418 | 10.01495 |
| 0.118 | 9.82638 | 9.85653 | 9.94699 | 10.09777 |
| 0.119 | 9.90918 | 9.93934 | 10.02981 | 10.1806 |
| 0.12 | 9.992 | 10.02216 | 10.11263 | 10.26343 |
| 0.121 | 10.07482 | 10.10498 | 10.19546 | 10.34627 |
| 0.122 | 10.15765 | 10.18781 | 10.27829 | 10.42911 |
| 0.123 | 10.24048 | 10.27064 | 10.36113 | 10.51196 |
| 0.124 | 10.32332 | 10.35348 | 10.44398 | 10.59481 |
| 0.125 | 10.40616 | 10.43633 | 10.52683 | 10.67767 |
| 0.126 | 10.48901 | 10.51918 | 10.60968 | 10.76054 |
| 0.127 | 10.57186 | 10.60203 | 10.69254 | 10.8434 |
| 0.128 | 10.65472 | 10.6849 | 10.77541 | 10.92628 |
| 0.129 | 10.73759 | 10.76776 | 10.85828 | 11.00916 |
| 0.13 | 10.82046 | 10.85063 | 10.94116 | 11.09204 |
| 0.131 | 10.90334 | 10.93351 | 11.02404 | 11.17493 |
| 0.132 | 10.98622 | 11.01639 | 11.10693 | 11.25783 |
| 0.133 | 11.0691 | 11.09928 | 11.18982 | 11.34073 |
| 0.134 | 11.15199 | 11.18218 | 11.27272 | 11.42363 |
| 0.135 | 11.23489 | 11.26507 | 11.35562 | 11.50654 |
| 0.136 | 11.31779 | 11.34797 | 11.43853 | 11.58945 |
| 0.137 | 11.4007 | 11.43088 | 11.52144 | 11.67237 |
| 0.138 | 11.48361 | 11.51379 | 11.60435 | 11.75529 |
| 0.139 | 11.56652 | 11.59671 | 11.68727 | 11.83822 |
| 0.14 | 11.64944 | 11.67963 | 11.7702 | 11.92115 |
| 0.141 | 11.73237 | 11.76256 | 11.85313 | 12.00409 |
| 0.142 | 11.81529 | 11.84548 | 11.93606 | 12.08703 |
| 0.143 | 11.89823 | 11.92842 | 12.019 | 12.16997 |
| 0.144 | 11.98116 | 12.01136 | 12.10194 | 12.25292 |
| 0.145 | 12.0641 | 12.0943 | 12.18489 | 12.33587 |
| 0.146 | 12.14705 | 12.17725 | 12.26784 | 12.41883 |
| 0.147 | 12.23 | 12.2602 | 12.35079 | 12.50179 |
| 0.148 | 12.31295 | 12.34315 | 12.43375 | 12.58475 |
| 0.149 | 12.39591 | 12.42611 | 12.51671 | 12.66772 |
| 0.15 | 12.47887 | 12.50907 | 12.59968 | 12.75069 |
| 0.151 | 12.56184 | 12.59204 | 12.68265 | 12.83367 |
| 0.152 | 12.64481 | 12.67501 | 12.76562 | 12.91665 |
| 0.153 | 12.72778 | 12.75799 | 12.8486 | 12.99963 |
| 0.154 | 12.81076 | 12.84096 | 12.93158 | 13.08262 |
| 0.155 | 12.89374 | 12.92395 | 13.01457 | 13.16561 |
| 0.156 | 12.97673 | 13.00693 | 13.09756 | 13.2486 |
| 0.157 | 13.05971 | 13.08992 | 13.18055 | 13.3316 |
| 0.158 | 13.14271 | 13.17292 | 13.26355 | 13.4146 |
| 0.159 | 13.2257 | 13.25591 | 13.34655 | 13.49761 |
| 0.16 | 13.3087 | 13.33891 | 13.42955 | 13.58062 |
| 0.161 | 13.39171 | 13.42192 | 13.51256 | 13.66363 |
| 0.162 | 13.47471 | 13.50493 | 13.59557 | 13.74664 |
| 0.163 | 13.55772 | 13.58794 | 13.67858 | 13.82966 |
| 0.164 | 13.64074 | 13.67095 | 13.7616 | 13.91268 |
| 0.165 | 13.72375 | 13.75397 | 13.84462 | 13.99571 |
| 0.166 | 13.80677 | 13.83699 | 13.92764 | 14.07874 |
| 0.167 | 13.8898 | 13.92002 | 14.01067 | 14.16177 |
| 0.168 | 13.97283 | 14.00304 | 14.0937 | 14.24481 |
| 0.169 | 14.05586 | 14.08608 | 14.17674 | 14.32784 |
| 0.17 | 14.13889 | 14.16911 | 14.25977 | 14.41089 |
| 0.171 | 14.22193 | 14.25215 | 14.34282 | 14.49393 |
| 0.172 | 14.30497 | 14.33519 | 14.42586 | 14.57698 |
| 0.173 | 14.38801 | 14.41823 | 14.50891 | 14.66003 |
| 0.174 | 14.47106 | 14.50128 | 14.59196 | 14.74308 |
| 0.175 | 14.55411 | 14.58433 | 14.67501 | 14.82614 |
| 0.176 | 14.63716 | 14.66739 | 14.75807 | 14.9092 |
| 0.177 | 14.72022 | 14.75044 | 14.84113 | 14.99227 |
| 0.178 | 14.80328 | 14.8335 | 14.92419 | 15.07533 |
| 0.179 | 14.88634 | 14.91657 | 15.00725 | 15.1584 |
| 0.18 | 14.9694 | 14.99963 | 15.09032 | 15.24147 |
| 0.181 | 15.05247 | 15.0827 | 15.17339 | 15.32455 |
| 0.182 | 15.13554 | 15.16578 | 15.25647 | 15.40763 |
| 0.183 | 15.21862 | 15.24885 | 15.33955 | 15.49071 |
| 0.184 | 15.3017 | 15.33193 | 15.42263 | 15.57379 |
| 0.185 | 15.38478 | 15.41501 | 15.50571 | 15.65688 |
| 0.186 | 15.46786 | 15.49809 | 15.5888 | 15.73997 |
| 0.187 | 15.55095 | 15.58118 | 15.67189 | 15.82306 |
| 0.188 | 15.63403 | 15.66427 | 15.75498 | 15.90616 |
| 0.189 | 15.71713 | 15.74736 | 15.83807 | 15.98926 |
| 0.19 | 15.80022 | 15.83046 | 15.92117 | 16.07236 |
| 0.191 | 15.88332 | 15.91356 | 16.00427 | 16.15546 |
| 0.192 | 15.96642 | 15.99666 | 16.08737 | 16.23857 |
| 0.193 | 16.04952 | 16.07976 | 16.17048 | 16.32168 |
| 0.194 | 16.13263 | 16.16287 | 16.25359 | 16.40479 |
| 0.195 | 16.21574 | 16.24598 | 16.3367 | 16.4879 |
| 0.196 | 16.29885 | 16.32909 | 16.41981 | 16.57102 |
| 0.197 | 16.38196 | 16.4122 | 16.50293 | 16.65414 |
| 0.198 | 16.46508 | 16.49532 | 16.58605 | 16.73726 |
| 0.199 | 16.5482 | 16.57844 | 16.66917 | 16.82039 |
| 0.2 | 16.63132 | 16.66156 | 16.7523 | 16.90352 |
