# Supplementary material for: Analysis of sinusoidal post-buckling deformation of horizontal coiled tubing with initial residual bending
Source: PLoS One. 2024 May 14;19(5):e0301610. doi: 10.1371/journal.pone.0301610 (PMC11093391; doi:10.1371/journal.pone.0301610)
Supplement: S1 File — (ZIP) [file pone.0301610.s001.zip › The values used to build graphs - Fig 13.docx]

## The values used to build graphs

The minimal data set of the original data for plotting curves in Fig 13 is as follows:

| x-axis | ‾*v* = 0.02 | ‾*v* = 0.04 | ‾*v* = 0.06 | ‾*v* = 0.08 |
| --- | --- | --- | --- | --- |
| 0 | 0.8171 | 0.8171 | 0.8171 | 0.8171 |
| 0.05 | 1.13669 | 2.09052 | 3.68023 | 5.90585 |
| 0.1 | 2.09298 | 5.90089 | 12.24736 | 21.13253 |
| 0.15 | 3.67853 | 12.21858 | 26.45189 | 46.37878 |
| 0.2 | 5.88102 | 20.99448 | 46.1834 | 81.44833 |
| 0.25 | 8.68333 | 32.16038 | 71.28852 | 126.0686 |
| 0.3 | 12.06367 | 45.62947 | 101.5721 | 179.8927 |
| 0.35 | 15.99579 | 61.29707 | 136.7987 | 242.5022 |
| 0.4 | 20.4491 | 79.04136 | 176.6944 | 313.4104 |
| 0.45 | 25.389 | 98.72443 | 220.9493 | 392.0662 |
| 0.5 | 30.77709 | 120.1933 | 269.2192 | 477.858 |
| 0.55 | 36.57148 | 143.281 | 321.1288 | 570.1189 |
| 0.6 | 42.72715 | 167.8081 | 376.2748 | 668.1318 |
| 0.65 | 49.19623 | 193.5839 | 434.2284 | 771.1347 |
| 0.7 | 55.92844 | 220.4082 | 494.539 | 878.327 |
| 0.75 | 62.87146 | 248.0722 | 556.7379 | 988.8753 |
| 0.8 | 69.97131 | 276.3611 | 620.3416 | 1101.92 |
| 0.85 | 77.1728 | 305.0549 | 684.8556 | 1216.583 |
| 0.9 | 84.41996 | 333.9305 | 749.7783 | 1331.972 |
| 0.95 | 91.65643 | 362.7634 | 814.6052 | 1447.191 |
| 1 | 98.82597 | 391.3296 | 878.8322 | 1561.344 |
| 1.05 | 105.8729 | 419.407 | 941.9601 | 1673.544 |
| 1.1 | 112.7423 | 446.7772 | 1003.498 | 1782.917 |
| 1.15 | 119.3809 | 473.2276 | 1062.968 | 1888.615 |
| 1.2 | 125.737 | 498.5525 | 1119.907 | 1989.815 |
| 1.25 | 131.7612 | 522.555 | 1173.873 | 2085.73 |
| 1.3 | 137.4067 | 545.0486 | 1224.447 | 2175.616 |
| 1.35 | 142.6297 | 565.8583 | 1271.234 | 2258.773 |
| 1.4 | 147.3894 | 584.8225 | 1313.872 | 2334.555 |
| 1.45 | 151.6489 | 601.7936 | 1352.029 | 2402.372 |
| 1.5 | 155.3751 | 616.6398 | 1385.409 | 2461.699 |
| 1.55 | 158.539 | 629.2456 | 1413.751 | 2512.072 |
| 1.6 | 161.116 | 639.5131 | 1436.836 | 2553.101 |
| 1.65 | 163.0861 | 647.3623 | 1454.484 | 2584.467 |
| 1.7 | 164.4339 | 652.7324 | 1466.557 | 2605.927 |
| 1.75 | 165.149 | 655.5815 | 1472.963 | 2617.312 |
| 1.8 | 165.2258 | 655.8876 | 1473.651 | 2618.535 |
| 1.85 | 164.6637 | 653.6481 | 1468.616 | 2609.586 |
| 1.9 | 163.4671 | 648.8806 | 1457.897 | 2590.535 |
| 1.95 | 161.6453 | 641.6221 | 1441.578 | 2561.529 |
| 2 | 159.2125 | 631.929 | 1419.784 | 2522.795 |
| 2.05 | 156.1875 | 619.8767 | 1392.687 | 2474.634 |
| 2.1 | 152.5939 | 605.5589 | 1360.495 | 2417.419 |
| 2.15 | 148.4597 | 589.0869 | 1323.46 | 2351.596 |
| 2.2 | 143.8169 | 570.5886 | 1281.87 | 2277.676 |
| 2.25 | 138.7017 | 550.2081 | 1236.047 | 2196.234 |
| 2.3 | 133.1538 | 528.1035 | 1186.348 | 2107.903 |
| 2.35 | 127.2164 | 504.4469 | 1133.16 | 2013.369 |
| 2.4 | 120.9356 | 479.4221 | 1076.895 | 1913.368 |
| 2.45 | 114.3602 | 453.2237 | 1017.992 | 1808.678 |
| 2.5 | 107.5414 | 426.0553 | 956.9078 | 1700.111 |
| 2.55 | 100.5322 | 398.128 | 894.1175 | 1588.511 |
| 2.6 | 93.38711 | 369.6591 | 830.1092 | 1474.747 |
| 2.65 | 86.1616 | 340.8699 | 765.3806 | 1359.703 |
| 2.7 | 78.91186 | 311.984 | 700.4347 | 1244.272 |
| 2.75 | 71.69427 | 283.2261 | 635.7766 | 1129.353 |
| 2.8 | 64.56493 | 254.8197 | 571.9088 | 1015.839 |
| 2.85 | 57.57925 | 226.9857 | 509.3278 | 904.6116 |
| 2.9 | 50.79155 | 199.9404 | 448.5201 | 796.5359 |
| 2.95 | 44.25458 | 173.8941 | 389.9584 | 692.4521 |
| 3 | 38.01915 | 149.0492 | 334.0979 | 593.1693 |
| 3.05 | 32.13374 | 125.5988 | 281.3729 | 499.4592 |
| 3.1 | 26.64409 | 103.7253 | 232.1932 | 412.0504 |
| 3.15 | 21.59287 | 83.59874 | 186.9411 | 331.6222 |
| 3.2 | 17.01935 | 65.37549 | 145.9685 | 258.8 |
| 3.25 | 12.95908 | 49.19723 | 109.5937 | 194.1499 |
| 3.3 | 9.44361 | 35.18974 | 78.09966 | 138.1743 |
| 3.35 | 6.50027 | 23.4619 | 51.73108 | 91.30843 |
| 3.4 | 4.15193 | 14.10487 | 30.69299 | 53.91663 |
| 3.45 | 2.41686 | 7.1914 | 15.1489 | 26.28955 |
| 3.5 | 1.30853 | 2.77521 | 5.21966 | 8.64194 |
| 3.55 | 0.83556 | 0.89065 | 0.98246 | 1.111 |
| 3.6 | 1.00163 | 1.55236 | 2.47023 | 3.75526 |
| 3.65 | 1.80544 | 4.75519 | 9.6714 | 16.55418 |
| 3.7 | 3.24076 | 10.47426 | 22.53 | 39.40825 |
| 3.75 | 5.29641 | 18.66509 | 40.94607 | 72.13982 |
| 3.8 | 7.95643 | 29.26403 | 64.77645 | 114.4945 |
| 3.85 | 11.20014 | 42.18869 | 93.8359 | 166.1429 |
| 3.9 | 15.00232 | 57.33858 | 127.8985 | 226.6836 |
| 3.95 | 19.33343 | 74.59595 | 166.6995 | 295.6461 |
| 4 | 24.15979 | 93.82665 | 209.9373 | 372.4941 |
| 4.05 | 29.4439 | 114.8812 | 257.2756 | 456.6303 |
| 4.1 | 35.14468 | 137.5959 | 308.3467 | 547.4008 |
| 4.15 | 41.21782 | 161.7942 | 362.7534 | 644.0997 |
| 4.2 | 47.6161 | 187.288 | 420.0728 | 745.9755 |
| 4.25 | 54.28981 | 213.8791 | 479.8593 | 852.2362 |
| 4.3 | 61.18705 | 241.3608 | 541.6482 | 962.0558 |
| 4.35 | 68.25422 | 269.5195 | 604.9592 | 1074.58 |
| 4.4 | 75.43638 | 298.1362 | 669.3 | 1188.936 |
| 4.45 | 82.6777 | 326.9886 | 734.1705 | 1304.232 |
| 4.5 | 89.92188 | 355.8523 | 799.0665 | 1419.574 |
| 4.55 | 97.11262 | 384.503 | 863.4835 | 1534.064 |
| 4.6 | 104.194 | 412.7179 | 926.9206 | 1646.813 |
| 4.65 | 111.111 | 440.2777 | 988.8848 | 1756.944 |
| 4.7 | 117.8098 | 466.9681 | 1048.894 | 1863.601 |
| 4.75 | 124.2384 | 492.5817 | 1106.483 | 1965.955 |
| 4.8 | 130.3467 | 516.9194 | 1161.202 | 2063.21 |
| 4.85 | 136.0874 | 539.7919 | 1212.628 | 2154.61 |
| 4.9 | 141.4157 | 561.0214 | 1260.359 | 2239.444 |
| 4.95 | 146.2902 | 580.443 | 1304.026 | 2317.054 |
| 5 | 150.6731 | 597.9056 | 1343.288 | 2386.836 |
| 5.05 | 154.5302 | 613.2734 | 1377.84 | 2448.246 |
| 5.1 | 157.8316 | 626.4271 | 1407.414 | 2500.809 |
| 5.15 | 160.5516 | 637.2643 | 1431.78 | 2544.115 |
| 5.2 | 162.669 | 645.7008 | 1450.748 | 2577.828 |
| 5.25 | 164.1675 | 651.6711 | 1464.171 | 2601.685 |
| 5.3 | 165.0353 | 655.1286 | 1471.945 | 2615.502 |
| 5.35 | 165.2657 | 656.0466 | 1474.009 | 2619.17 |
| 5.4 | 164.8569 | 654.4179 | 1470.347 | 2612.662 |
| 5.45 | 163.8121 | 650.2551 | 1460.988 | 2596.027 |
| 5.5 | 162.1394 | 643.5906 | 1446.003 | 2569.395 |
| 5.55 | 159.8518 | 634.4762 | 1425.511 | 2532.974 |
| 5.6 | 156.9671 | 622.9829 | 1399.67 | 2487.046 |
| 5.65 | 153.5078 | 609.1998 | 1368.681 | 2431.968 |
| 5.7 | 149.5006 | 593.2342 | 1332.785 | 2368.169 |
| 5.75 | 144.9769 | 575.2102 | 1292.261 | 2296.144 |
| 5.8 | 139.9716 | 555.268 | 1247.424 | 2216.453 |
| 5.85 | 134.5239 | 533.5624 | 1198.622 | 2129.717 |
| 5.9 | 128.6759 | 510.2623 | 1146.235 | 2036.608 |
| 5.95 | 122.4733 | 485.5489 | 1090.671 | 1937.851 |
| 6 | 115.9641 | 459.6141 | 1032.36 | 1834.214 |
| 6.05 | 109.199 | 432.6597 | 971.757 | 1726.502 |
| 6.1 | 102.2307 | 404.8952 | 909.3324 | 1615.553 |
| 6.15 | 95.11315 | 376.5363 | 845.5716 | 1502.229 |
| 6.2 | 87.90184 | 347.8037 | 780.9702 | 1387.411 |
| 6.25 | 80.65278 | 318.9205 | 716.0305 | 1271.991 |
| 6.3 | 73.42233 | 290.1114 | 651.2573 | 1156.868 |
| 6.35 | 66.26669 | 261.6003 | 587.154 | 1042.935 |
| 6.4 | 59.2415 | 233.6088 | 524.219 | 931.0782 |
| 6.45 | 52.40135 | 206.3546 | 462.9415 | 822.1676 |
| 6.5 | 45.79942 | 180.0495 | 403.798 | 717.0497 |
| 6.55 | 39.48703 | 154.8979 | 347.248 | 616.5415 |
| 6.6 | 33.51325 | 131.0955 | 293.7313 | 521.4244 |
| 6.65 | 27.9245 | 108.8271 | 243.6639 | 432.4377 |
| 6.7 | 22.76424 | 88.26605 | 197.435 | 350.2733 |
| 6.75 | 18.07256 | 69.57201 | 155.4038 | 275.5698 |
| 6.8 | 13.88595 | 52.89036 | 117.8973 | 208.908 |
| 6.85 | 10.23693 | 38.35076 | 85.20681 | 150.8061 |
| 6.9 | 7.15388 | 26.06624 | 57.58662 | 101.7157 |
| 6.95 | 4.66075 | 16.13229 | 35.25138 | 62.01844 |
| 7 | 2.77693 | 8.62613 | 18.37472 | 32.02292 |
| 7.05 | 1.51706 | 3.60611 | 7.08783 | 11.96231 |
| 7.1 | 0.89093 | 1.11125 | 1.47845 | 1.99255 |
| 7.15 | 0.9034 | 1.16095 | 1.59019 | 2.19114 |
| 7.2 | 1.55438 | 3.75481 | 7.42218 | 12.55655 |
| 7.25 | 2.83881 | 8.87269 | 18.92907 | 33.00819 |
| 7.3 | 4.74671 | 16.47478 | 36.02143 | 63.38708 |
| 7.35 | 7.26324 | 26.50201 | 58.56638 | 103.4571 |
| 7.4 | 10.36885 | 38.87641 | 86.38867 | 152.9067 |
| 7.45 | 14.0394 | 53.50181 | 119.272 | 211.3515 |
| 7.5 | 18.24636 | 70.26451 | 156.9608 | 278.3371 |
| 7.55 | 22.95702 | 89.03421 | 199.1621 | 353.343 |
| 7.6 | 28.13478 | 109.665 | 245.5477 | 435.7859 |
| 7.65 | 33.73938 | 131.9965 | 295.7572 | 525.025 |
| 7.7 | 39.72727 | 155.8551 | 349.4002 | 620.3666 |
| 7.75 | 46.05189 | 181.0554 | 406.0597 | 721.0695 |
| 7.8 | 52.66408 | 207.4015 | 465.2952 | 826.351 |
| 7.85 | 59.51246 | 234.6885 | 526.6464 | 935.3925 |
| 7.9 | 66.54378 | 262.7043 | 589.6362 | 1047.346 |
| 7.95 | 73.70338 | 291.2312 | 653.775 | 1161.343 |
| 8 | 80.93561 | 320.0474 | 718.5642 | 1276.495 |
| 8.05 | 88.18425 | 348.9289 | 783.5002 | 1391.908 |
| 8.1 | 95.39295 | 377.6512 | 848.0781 | 1506.684 |
| 8.15 | 102.5057 | 405.9909 | 911.796 | 1619.932 |
| 8.2 | 109.4671 | 433.7279 | 974.1586 | 1730.771 |
| 8.25 | 116.2232 | 460.6464 | 1034.681 | 1838.339 |
| 8.3 | 122.7213 | 486.5372 | 1092.893 | 1941.801 |
| 8.35 | 128.9111 | 511.1991 | 1148.341 | 2040.351 |
| 8.4 | 134.7442 | 534.4403 | 1200.596 | 2133.224 |
| 8.45 | 140.1755 | 556.0801 | 1249.249 | 2219.699 |
| 8.5 | 145.1626 | 575.9503 | 1293.925 | 2299.101 |
| 8.55 | 149.6669 | 593.8966 | 1334.274 | 2370.815 |
| 8.6 | 153.6532 | 609.7792 | 1369.984 | 2434.283 |
| 8.65 | 157.0906 | 623.4748 | 1400.776 | 2489.012 |
| 8.7 | 159.9524 | 634.8769 | 1426.412 | 2534.575 |
| 8.75 | 162.2163 | 643.8969 | 1446.692 | 2570.62 |
| 8.8 | 163.8647 | 650.4646 | 1461.459 | 2596.864 |
| 8.85 | 164.8848 | 654.529 | 1470.597 | 2613.106 |
| 8.9 | 165.2687 | 656.0585 | 1474.036 | 2619.218 |
| 8.95 | 165.0134 | 655.0412 | 1471.748 | 2615.153 |
| 9 | 164.1208 | 651.485 | 1463.753 | 2600.942 |
| 9.05 | 162.5979 | 645.4176 | 1450.111 | 2576.696 |
| 9.1 | 160.4566 | 636.886 | 1430.929 | 2542.604 |
| 9.15 | 157.7135 | 625.9567 | 1406.356 | 2498.93 |
| 9.2 | 154.3899 | 612.7147 | 1376.584 | 2446.014 |
| 9.25 | 150.5117 | 597.2627 | 1341.842 | 2384.267 |
| 9.3 | 146.109 | 579.7211 | 1302.403 | 2314.169 |
| 9.35 | 141.216 | 560.226 | 1258.571 | 2236.266 |
| 9.4 | 135.8709 | 538.9292 | 1210.688 | 2151.163 |
| 9.45 | 130.115 | 515.9961 | 1159.127 | 2059.521 |
| 9.5 | 123.9933 | 491.6051 | 1104.287 | 1962.052 |
| 9.55 | 117.5532 | 465.9457 | 1046.596 | 1859.516 |
| 9.6 | 110.8449 | 439.2174 | 986.5009 | 1752.707 |
| 9.65 | 103.9205 | 411.628 | 924.4701 | 1642.458 |
| 9.7 | 96.83377 | 383.3919 | 860.9855 | 1529.625 |
| 9.75 | 89.6399 | 354.7288 | 796.5404 | 1415.084 |
| 9.8 | 82.39477 | 325.8613 | 731.6359 | 1299.727 |
| 9.85 | 75.15471 | 297.0139 | 666.7766 | 1184.451 |
| 9.9 | 67.97599 | 268.4109 | 602.4666 | 1070.15 |
| 9.95 | 60.91443 | 240.2745 | 539.2059 | 957.715 |
| 10 | 54.02491 | 212.8236 | 477.4862 | 848.0184 |
| 10.05 | 47.36099 | 186.2715 | 417.7873 | 741.9135 |
| 10.1 | 40.97447 | 160.8246 | 360.5733 | 640.2251 |
| 10.15 | 34.91499 | 136.6807 | 306.289 | 543.7436 |
| 10.2 | 29.22966 | 114.0275 | 255.3563 | 453.2191 |
| 10.25 | 23.96266 | 93.04118 | 208.1712 | 369.3553 |
| 10.3 | 19.15494 | 73.88476 | 165.1005 | 292.8041 |
| 10.35 | 14.84386 | 56.7072 | 126.4789 | 224.1606 |
| 10.4 | 11.06294 | 41.64202 | 92.60679 | 163.9584 |
| 10.45 | 7.84156 | 28.80634 | 63.74738 | 112.6654 |
| 10.5 | 5.20476 | 18.29992 | 40.12503 | 70.68055 |
| 10.55 | 3.17304 | 10.20445 | 21.92337 | 38.33006 |
| 10.6 | 1.76219 | 4.58284 | 9.2839 | 15.86545 |
| 10.65 | 0.98317 | 1.47881 | 2.30486 | 3.46136 |
| 10.7 | 0.84204 | 0.91647 | 1.04052 | 1.21419 |
| 10.75 | 1.3399 | 2.90021 | 5.5007 | 9.14144 |
| 10.8 | 2.47287 | 7.41459 | 15.65073 | 27.18146 |
| 10.85 | 4.23216 | 14.42453 | 31.4117 | 55.19403 |
| 10.9 | 6.60408 | 23.87554 | 52.6611 | 92.96138 |
| 10.95 | 9.5702 | 35.69414 | 79.23374 | 140.1899 |
| 11 | 13.10746 | 49.78848 | 110.9231 | 196.5126 |
| 11.05 | 17.18838 | 66.04898 | 147.4827 | 261.4914 |
| 11.1 | 21.78123 | 84.34925 | 188.6285 | 334.6213 |
| 11.15 | 26.85031 | 104.547 | 234.0406 | 415.3339 |
| 11.2 | 32.35622 | 126.4853 | 283.366 | 503.0017 |
| 11.25 | 38.25616 | 149.9936 | 336.2212 | 596.9431 |
| 11.3 | 44.50428 | 174.889 | 392.1954 | 696.428 |
| 11.35 | 51.052 | 200.9782 | 450.8533 | 800.6829 |
| 11.4 | 57.84843 | 228.0582 | 511.7392 | 908.8975 |
| 11.45 | 64.84074 | 255.9187 | 574.3796 | 1020.23 |
| 11.5 | 71.97457 | 284.3429 | 638.2876 | 1133.816 |
| 11.55 | 79.19447 | 313.11 | 702.9664 | 1248.772 |
| 11.6 | 86.44431 | 341.9963 | 767.9132 | 1364.204 |
| 11.65 | 93.66774 | 370.7773 | 832.6232 | 1479.215 |
| 11.7 | 100.8086 | 399.2292 | 896.5932 | 1592.911 |
| 11.75 | 107.8114 | 427.1308 | 959.326 | 1704.409 |
| 11.8 | 114.6217 | 454.2653 | 1020.334 | 1812.84 |
| 11.85 | 121.1865 | 480.4217 | 1079.143 | 1917.363 |
| 11.9 | 127.4548 | 505.3967 | 1135.296 | 2017.165 |
| 11.95 | 133.3778 | 528.9962 | 1188.355 | 2111.47 |
| 12 | 138.9096 | 551.0366 | 1237.91 | 2199.544 |
| 12.05 | 144.0071 | 571.3466 | 1283.574 | 2280.704 |
| 12.1 | 148.6307 | 589.7684 | 1324.992 | 2354.319 |
| 12.15 | 152.7445 | 606.1587 | 1361.844 | 2419.815 |
| 12.2 | 156.3164 | 620.3901 | 1393.841 | 2476.685 |
| 12.25 | 159.3187 | 632.352 | 1420.735 | 2524.485 |
| 12.3 | 161.728 | 641.9514 | 1442.318 | 2562.845 |
| 12.35 | 163.5256 | 649.1136 | 1458.421 | 2591.466 |
| 12.4 | 164.6976 | 653.7831 | 1468.92 | 2610.125 |
| 12.45 | 165.2348 | 655.9234 | 1473.732 | 2618.678 |
| 12.5 | 165.133 | 655.518 | 1472.82 | 2617.058 |
| 12.55 | 164.3931 | 652.5699 | 1466.192 | 2605.277 |
| 12.6 | 163.0208 | 647.1022 | 1453.899 | 2583.428 |
| 12.65 | 161.0267 | 639.1574 | 1436.036 | 2551.68 |
| 12.7 | 158.4264 | 628.7971 | 1412.742 | 2510.28 |
| 12.75 | 155.2401 | 616.1019 | 1384.199 | 2459.549 |
| 12.8 | 151.4925 | 601.1705 | 1350.628 | 2399.883 |
| 12.85 | 147.2128 | 584.1191 | 1312.291 | 2331.744 |
| 12.9 | 142.4343 | 565.0801 | 1269.485 | 2255.663 |
| 12.95 | 137.1941 | 544.2015 | 1222.542 | 2172.231 |
| 13 | 131.533 | 521.6457 | 1171.829 | 2082.097 |
| 13.05 | 125.4949 | 497.588 | 1117.739 | 1985.961 |
| 13.1 | 119.1268 | 472.2155 | 1060.692 | 1884.57 |
| 13.15 | 112.4783 | 445.7253 | 1001.133 | 1778.713 |
| 13.2 | 105.6009 | 418.3234 | 939.5239 | 1669.214 |
| 13.25 | 98.5482 | 390.2229 | 876.3439 | 1556.922 |
| 13.3 | 91.375 | 361.6421 | 812.084 | 1442.71 |
| 13.35 | 84.13705 | 332.8033 | 747.2439 | 1327.468 |
| 13.4 | 76.89063 | 303.9305 | 682.3277 | 1212.09 |
| 13.45 | 69.69205 | 275.2484 | 617.8399 | 1097.474 |
| 13.5 | 62.59729 | 246.9798 | 554.2818 | 984.5099 |
| 13.55 | 55.6615 | 219.3445 | 492.1476 | 874.0766 |
| 13.6 | 48.93858 | 192.5573 | 431.9202 | 767.0324 |
| 13.65 | 42.4808 | 166.8265 | 374.0679 | 664.2093 |
| 13.7 | 36.33835 | 142.352 | 319.0403 | 566.4068 |
| 13.75 | 30.55898 | 119.3242 | 267.2652 | 474.3851 |
| 13.8 | 25.18761 | 97.922 | 219.1451 | 388.8596 |
| 13.85 | 20.266 | 78.3118 | 175.0541 | 310.495 |
| 13.9 | 15.8324 | 60.64604 | 135.3349 | 239.9006 |
| 13.95 | 11.92127 | 45.06204 | 100.2963 | 177.6252 |
| 14 | 8.56301 | 31.68096 | 70.2106 | 124.1528 |
| 14.05 | 5.78372 | 20.60679 | 45.31174 | 79.89909 |
| 14.1 | 3.60501 | 11.92564 | 25.79325 | 45.20816 |
| 14.15 | 2.04381 | 5.70498 | 11.80687 | 20.34963 |
| 14.2 | 1.11226 | 1.99316 | 3.46132 | 5.51677 |
| 14.25 | 0.81759 | 0.81904 | 0.82146 | 0.82485 |
| 14.3 | 1.1621 | 2.19175 | 3.90783 | 6.31037 |
| 14.35 | 2.14311 | 6.10062 | 12.69643 | 21.93068 |
| 14.4 | 3.75299 | 12.51526 | 27.11895 | 47.56436 |
| 14.45 | 5.97923 | 21.38581 | 47.06325 | 83.01212 |
| 14.5 | 8.80453 | 32.64331 | 72.37432 | 127.9984 |
| 14.55 | 12.20692 | 46.20025 | 102.8554 | 182.1736 |
| 14.6 | 16.15997 | 61.95125 | 138.2695 | 245.1164 |
| 14.65 | 20.63294 | 79.77388 | 178.3414 | 316.3376 |
| 14.7 | 25.59107 | 99.52958 | 222.7595 | 395.2836 |
| 14.75 | 30.99581 | 121.0648 | 271.1786 | 481.3407 |
| 14.8 | 36.80517 | 144.2121 | 323.2223 | 573.8398 |
| 14.85 | 42.97397 | 168.7916 | 378.486 | 672.0619 |
| 14.9 | 49.45428 | 194.6121 | 436.5401 | 775.2435 |
| 14.95 | 56.19571 | 221.4731 | 496.9333 | 882.5825 |
| 15 | 63.14587 | 249.1656 | 559.1962 | 993.2444 |
