# Supplementary material for: Analysis of sinusoidal post-buckling deformation of horizontal coiled tubing with initial residual bending
Source: PLoS One. 2024 May 14;19(5):e0301610. doi: 10.1371/journal.pone.0301610 (PMC11093391; doi:10.1371/journal.pone.0301610)
Supplement: S1 File — (ZIP) [file pone.0301610.s001.zip › The values used to build graphs - Fig 12.docx]

## The values used to build graphs

The minimal data set of the original data for plotting curves in Fig 12 is as follows:

| x-axis | ‾*L*_z_ = 0.005 | ‾*L*_z_ = 0.01 | ‾*L*_z_ = 0.015 | ‾*L*_z_ = 0.02 |
| --- | --- | --- | --- | --- |
| 0 | 0.78769 | 0.8171 | 1.07334 | 1.54361 |
| 0.05 | 0.81317 | 0.83862 | 1.09082 | 1.55788 |
| 0.1 | 0.88938 | 0.90301 | 1.14314 | 1.60062 |
| 0.15 | 1.01564 | 1.00977 | 1.22994 | 1.67156 |
| 0.2 | 1.19085 | 1.15806 | 1.35063 | 1.77027 |
| 0.25 | 1.41347 | 1.34675 | 1.50439 | 1.89616 |
| 0.3 | 1.68154 | 1.57437 | 1.69015 | 2.04847 |
| 0.35 | 1.99269 | 1.83915 | 1.90665 | 2.22628 |
| 0.4 | 2.34419 | 2.13903 | 2.1524 | 2.42851 |
| 0.45 | 2.73294 | 2.4717 | 2.42573 | 2.65393 |
| 0.5 | 3.15553 | 2.83456 | 2.72475 | 2.90119 |
| 0.55 | 3.60823 | 3.2248 | 3.04742 | 3.16879 |
| 0.6 | 4.08706 | 3.63939 | 3.39152 | 3.45509 |
| 0.65 | 4.5878 | 4.07511 | 3.75469 | 3.75837 |
| 0.7 | 5.10605 | 4.52858 | 4.13445 | 4.07678 |
| 0.75 | 5.63723 | 4.99628 | 4.52817 | 4.40837 |
| 0.8 | 6.17669 | 5.47457 | 4.93316 | 4.75114 |
| 0.85 | 6.71966 | 5.95973 | 5.34663 | 5.10298 |
| 0.9 | 7.26137 | 6.44799 | 5.76574 | 5.46176 |
| 0.95 | 7.79704 | 6.93557 | 6.18759 | 5.82527 |
| 1 | 8.32197 | 7.41867 | 6.60929 | 6.19129 |
| 1.05 | 8.83152 | 7.89352 | 7.02793 | 6.55758 |
| 1.1 | 9.32121 | 8.35645 | 7.44063 | 6.92189 |
| 1.15 | 9.78673 | 8.80384 | 7.84452 | 7.28197 |
| 1.2 | 10.22399 | 9.23222 | 8.23684 | 7.63562 |
| 1.25 | 10.62912 | 9.63825 | 8.61487 | 7.98066 |
| 1.3 | 10.99856 | 10.01877 | 8.97599 | 8.31494 |
| 1.35 | 11.32905 | 10.37083 | 9.31771 | 8.63642 |
| 1.4 | 11.6177 | 10.69168 | 9.63767 | 8.9431 |
| 1.45 | 11.86195 | 10.97881 | 9.93365 | 9.23309 |
| 1.5 | 12.05965 | 11.23001 | 10.20361 | 9.50459 |
| 1.55 | 12.20906 | 11.4433 | 10.44568 | 9.75591 |
| 1.6 | 12.30887 | 11.61704 | 10.65817 | 9.98549 |
| 1.65 | 12.35819 | 11.74985 | 10.83962 | 10.19191 |
| 1.7 | 12.3566 | 11.84072 | 10.98877 | 10.37389 |
| 1.75 | 12.3041 | 11.88893 | 11.10459 | 10.53029 |
| 1.8 | 12.20116 | 11.89411 | 11.18626 | 10.66014 |
| 1.85 | 12.04869 | 11.85622 | 11.23324 | 10.76263 |
| 1.9 | 11.84802 | 11.77554 | 11.24518 | 10.83712 |
| 1.95 | 11.60093 | 11.65272 | 11.22202 | 10.88315 |
| 2 | 11.30959 | 11.48871 | 11.1639 | 10.90043 |
| 2.05 | 10.97657 | 11.28478 | 11.07123 | 10.88886 |
| 2.1 | 10.6048 | 11.04252 | 10.94466 | 10.8485 |
| 2.15 | 10.19755 | 10.76383 | 10.78506 | 10.77961 |
| 2.2 | 9.75842 | 10.45086 | 10.59353 | 10.68261 |
| 2.25 | 9.29126 | 10.10606 | 10.37141 | 10.55812 |
| 2.3 | 8.8002 | 9.73211 | 10.12024 | 10.4069 |
| 2.35 | 8.28955 | 9.33193 | 9.84175 | 10.2299 |
| 2.4 | 7.76382 | 8.90862 | 9.53786 | 10.02821 |
| 2.45 | 7.22763 | 8.46548 | 9.2107 | 9.8031 |
| 2.5 | 6.6857 | 8.00596 | 8.86251 | 9.55596 |
| 2.55 | 6.14281 | 7.53364 | 8.4957 | 9.28833 |
| 2.6 | 5.60374 | 7.05218 | 8.11282 | 9.00186 |
| 2.65 | 5.07322 | 6.56534 | 7.7165 | 8.69834 |
| 2.7 | 4.55594 | 6.07689 | 7.30948 | 8.37963 |
| 2.75 | 4.05644 | 5.59064 | 6.89458 | 8.04772 |
| 2.8 | 3.57912 | 5.11036 | 6.47464 | 7.70464 |
| 2.85 | 3.12819 | 4.63978 | 6.05258 | 7.35253 |
| 2.9 | 2.70761 | 4.18257 | 5.63129 | 6.99354 |
| 2.95 | 2.32109 | 3.74227 | 5.21367 | 6.62989 |
| 3 | 1.97203 | 3.3223 | 4.80261 | 6.26382 |
| 3.05 | 1.66349 | 2.92592 | 4.40092 | 5.89756 |
| 3.1 | 1.3982 | 2.55622 | 4.01137 | 5.53338 |
| 3.15 | 1.17849 | 2.21606 | 3.63664 | 5.17349 |
| 3.2 | 1.0063 | 1.90807 | 3.2793 | 4.8201 |
| 3.25 | 0.88314 | 1.63466 | 2.9418 | 4.47537 |
| 3.3 | 0.81009 | 1.39795 | 2.62646 | 4.1414 |
| 3.35 | 0.78779 | 1.19976 | 2.33544 | 3.82023 |
| 3.4 | 0.81645 | 1.04164 | 2.07074 | 3.51381 |
| 3.45 | 0.89581 | 0.92482 | 1.83416 | 3.224 |
| 3.5 | 1.02517 | 0.85019 | 1.62734 | 2.95257 |
| 3.55 | 1.2034 | 0.81835 | 1.45169 | 2.70117 |
| 3.6 | 1.42892 | 0.82953 | 1.3084 | 2.47131 |
| 3.65 | 1.69975 | 0.88365 | 1.19847 | 2.26439 |
| 3.7 | 2.01351 | 0.98029 | 1.12264 | 2.08166 |
| 3.75 | 2.36743 | 1.1187 | 1.08143 | 1.92423 |
| 3.8 | 2.75841 | 1.29781 | 1.07513 | 1.79304 |
| 3.85 | 3.18299 | 1.51622 | 1.10377 | 1.6889 |
| 3.9 | 3.63744 | 1.77225 | 1.16717 | 1.61243 |
| 3.95 | 4.11777 | 2.0639 | 1.26488 | 1.56409 |
| 4 | 4.61973 | 2.38892 | 1.39625 | 1.54417 |
| 4.05 | 5.13892 | 2.74477 | 1.56036 | 1.5528 |
| 4.1 | 5.67076 | 3.1287 | 1.7561 | 1.58991 |
| 4.15 | 6.21058 | 3.53773 | 1.98212 | 1.6553 |
| 4.2 | 6.75361 | 3.96868 | 2.23689 | 1.74856 |
| 4.25 | 7.29508 | 4.41821 | 2.51864 | 1.86913 |
| 4.3 | 7.83022 | 4.88281 | 2.82546 | 2.01628 |
| 4.35 | 8.35432 | 5.35889 | 3.15523 | 2.18912 |
| 4.4 | 8.86276 | 5.84274 | 3.50569 | 2.38661 |
| 4.45 | 9.35107 | 6.33061 | 3.87444 | 2.60755 |
| 4.5 | 9.81494 | 6.8187 | 4.25895 | 2.85061 |
| 4.55 | 10.25029 | 7.30321 | 4.65658 | 3.1143 |
| 4.6 | 10.65329 | 7.78039 | 5.06459 | 3.39704 |
| 4.65 | 11.02039 | 8.24651 | 5.48017 | 3.69711 |
| 4.7 | 11.34835 | 8.69796 | 5.90047 | 4.01267 |
| 4.75 | 11.63429 | 9.13122 | 6.32259 | 4.34181 |
| 4.8 | 11.87569 | 9.54291 | 6.74363 | 4.68253 |
| 4.85 | 12.07042 | 9.92984 | 7.16068 | 5.03275 |
| 4.9 | 12.21676 | 10.289 | 7.57087 | 5.39033 |
| 4.95 | 12.31344 | 10.61758 | 7.97136 | 5.75308 |
| 5 | 12.35959 | 10.91303 | 8.3594 | 6.11878 |
| 5.05 | 12.3548 | 11.17305 | 8.7323 | 6.4852 |
| 5.1 | 12.29913 | 11.39561 | 9.08748 | 6.85007 |
| 5.15 | 12.19307 | 11.57899 | 9.4225 | 7.21117 |
| 5.2 | 12.03754 | 11.72174 | 9.73502 | 7.56627 |
| 5.25 | 11.83391 | 11.82276 | 10.0229 | 7.91317 |
| 5.3 | 11.58399 | 11.88127 | 10.28413 | 8.24975 |
| 5.35 | 11.28996 | 11.8968 | 10.51692 | 8.57393 |
| 5.4 | 10.95443 | 11.86924 | 10.71963 | 8.88369 |
| 5.45 | 10.58034 | 11.7988 | 10.89088 | 9.17713 |
| 5.5 | 10.171 | 11.68603 | 11.02947 | 9.45242 |
| 5.55 | 9.73 | 11.53181 | 11.13445 | 9.70786 |
| 5.6 | 9.26122 | 11.33734 | 11.20508 | 9.94186 |
| 5.65 | 8.76881 | 11.10413 | 11.24088 | 10.15297 |
| 5.7 | 8.25708 | 10.834 | 11.24159 | 10.33988 |
| 5.75 | 7.73056 | 10.52905 | 11.20722 | 10.50142 |
| 5.8 | 7.19387 | 10.19166 | 11.138 | 10.6366 |
| 5.85 | 6.65174 | 9.82446 | 11.03441 | 10.74455 |
| 5.9 | 6.10895 | 9.4303 | 10.89717 | 10.82463 |
| 5.95 | 5.57027 | 9.01225 | 10.72723 | 10.87632 |
| 6 | 5.04045 | 8.57357 | 10.52577 | 10.8993 |
| 6.05 | 4.52414 | 8.11767 | 10.29418 | 10.89344 |
| 6.1 | 4.0259 | 7.64809 | 10.03407 | 10.85876 |
| 6.15 | 3.55012 | 7.16849 | 9.74724 | 10.79548 |
| 6.2 | 3.10097 | 6.68259 | 9.43568 | 10.70401 |
| 6.25 | 2.68241 | 6.19418 | 9.10153 | 10.5849 |
| 6.3 | 2.29814 | 5.70706 | 8.74712 | 10.43889 |
| 6.35 | 1.95152 | 5.225 | 8.3749 | 10.26691 |
| 6.4 | 1.64561 | 4.75175 | 7.98742 | 10.07002 |
| 6.45 | 1.38311 | 4.291 | 7.58738 | 9.84944 |
| 6.5 | 1.16632 | 3.84632 | 7.17753 | 9.60654 |
| 6.55 | 0.99715 | 3.42116 | 6.76071 | 9.34283 |
| 6.6 | 0.87709 | 3.01883 | 6.33978 | 9.05995 |
| 6.65 | 0.8072 | 2.64245 | 5.91764 | 8.75966 |
| 6.7 | 0.78809 | 2.29494 | 5.49721 | 8.4438 |
| 6.75 | 0.81993 | 1.979 | 5.08138 | 8.11434 |
| 6.8 | 0.90244 | 1.69708 | 4.67301 | 7.77331 |
| 6.85 | 1.0349 | 1.45137 | 4.27491 | 7.42281 |
| 6.9 | 1.21613 | 1.24377 | 3.88981 | 7.06501 |
| 6.95 | 1.44455 | 1.0759 | 3.52036 | 6.70211 |
| 7 | 1.71814 | 0.94906 | 3.16911 | 6.33633 |
| 7.05 | 2.03449 | 0.86423 | 2.83845 | 5.96994 |
| 7.1 | 2.39082 | 0.82207 | 2.53066 | 5.60516 |
| 7.15 | 2.784 | 0.82291 | 2.24784 | 5.24425 |
| 7.2 | 3.21056 | 0.86674 | 1.99195 | 4.8894 |
| 7.25 | 3.66675 | 0.95323 | 1.76472 | 4.54279 |
| 7.3 | 4.14856 | 1.08169 | 1.56772 | 4.20653 |
| 7.35 | 4.65173 | 1.25113 | 1.4023 | 3.88267 |
| 7.4 | 5.17185 | 1.46025 | 1.26958 | 3.57318 |
| 7.45 | 5.70433 | 1.70741 | 1.17048 | 3.27994 |
| 7.5 | 6.24448 | 1.9907 | 1.10568 | 3.00474 |
| 7.55 | 6.78756 | 2.30792 | 1.07561 | 2.74925 |
| 7.6 | 7.32877 | 2.65661 | 1.08049 | 2.51501 |
| 7.65 | 7.86336 | 3.03406 | 1.12028 | 2.30345 |
| 7.7 | 8.38661 | 3.43734 | 1.19471 | 2.11584 |
| 7.75 | 8.89392 | 3.86332 | 1.30327 | 1.95332 |
| 7.8 | 9.38082 | 4.3087 | 1.44521 | 1.81687 |
| 7.85 | 9.84303 | 4.77001 | 1.61957 | 1.70733 |
| 7.9 | 10.27647 | 5.24366 | 1.82515 | 1.62534 |
| 7.95 | 10.67733 | 5.72599 | 2.06054 | 1.5714 |
| 8 | 11.04207 | 6.21324 | 2.32413 | 1.54585 |
| 8.05 | 11.36749 | 6.70162 | 2.61412 | 1.54882 |
| 8.1 | 11.65071 | 7.18734 | 2.92851 | 1.58032 |
| 8.15 | 11.88925 | 7.66662 | 3.26515 | 1.64014 |
| 8.2 | 12.081 | 8.13573 | 3.62173 | 1.72792 |
| 8.25 | 12.22427 | 8.59103 | 3.99581 | 1.84314 |
| 8.3 | 12.31781 | 9.02897 | 4.3848 | 1.9851 |
| 8.35 | 12.36078 | 9.44615 | 4.78605 | 2.15293 |
| 8.4 | 12.35281 | 9.83931 | 5.19679 | 2.34564 |
| 8.45 | 12.29397 | 10.2054 | 5.61419 | 2.56204 |
| 8.5 | 12.18478 | 10.54157 | 6.03539 | 2.80083 |
| 8.55 | 12.0262 | 10.8452 | 6.45748 | 3.06057 |
| 8.6 | 11.81963 | 11.11393 | 6.87756 | 3.33967 |
| 8.65 | 11.56688 | 11.34566 | 7.29273 | 3.63645 |
| 8.7 | 11.27018 | 11.53859 | 7.70012 | 3.94909 |
| 8.75 | 10.93214 | 11.69121 | 8.09693 | 4.27571 |
| 8.8 | 10.55575 | 11.80235 | 8.48041 | 4.6143 |
| 8.85 | 10.14432 | 11.87112 | 8.84792 | 4.96281 |
| 8.9 | 9.70146 | 11.897 | 9.19692 | 5.3191 |
| 8.95 | 9.23109 | 11.87979 | 9.52498 | 5.681 |
| 9 | 8.73734 | 11.81961 | 9.82986 | 6.0463 |
| 9.05 | 8.22455 | 11.71695 | 10.10942 | 6.41275 |
| 9.1 | 7.69726 | 11.57258 | 10.36174 | 6.77811 |
| 9.15 | 7.16009 | 11.38765 | 10.58508 | 7.14013 |
| 9.2 | 6.61778 | 11.1636 | 10.77787 | 7.49659 |
| 9.25 | 6.07511 | 10.90216 | 10.9388 | 7.84529 |
| 9.3 | 5.53684 | 10.60537 | 11.06674 | 8.18408 |
| 9.35 | 5.00773 | 10.27554 | 11.1608 | 8.51087 |
| 9.4 | 4.49242 | 9.91525 | 11.22033 | 8.82364 |
| 9.45 | 3.99546 | 9.5273 | 11.24493 | 9.12045 |
| 9.5 | 3.52122 | 9.1147 | 11.23441 | 9.39947 |
| 9.55 | 3.07387 | 8.68067 | 11.18886 | 9.65897 |
| 9.6 | 2.65735 | 8.22858 | 11.10858 | 9.89733 |
| 9.65 | 2.27533 | 7.76196 | 10.99414 | 10.11308 |
| 9.7 | 1.93117 | 7.28442 | 10.84633 | 10.30488 |
| 9.75 | 1.6279 | 6.7997 | 10.66617 | 10.47152 |
| 9.8 | 1.3682 | 6.31155 | 10.45492 | 10.61199 |
| 9.85 | 1.15433 | 5.82377 | 10.21403 | 10.72539 |
| 9.9 | 0.98819 | 5.34015 | 9.94517 | 10.81103 |
| 9.95 | 0.87124 | 4.86445 | 9.6502 | 10.86836 |
| 10 | 0.80451 | 4.40036 | 9.33118 | 10.89704 |
| 10.05 | 0.78859 | 3.9515 | 8.9903 | 10.89689 |
| 10.1 | 0.82361 | 3.52134 | 8.62992 | 10.8679 |
| 10.15 | 0.90927 | 3.11323 | 8.25253 | 10.81026 |
| 10.2 | 1.04481 | 2.73034 | 7.86074 | 10.72432 |
| 10.25 | 1.22905 | 2.37564 | 7.45726 | 10.61062 |
| 10.3 | 1.46035 | 2.05188 | 7.04487 | 10.46987 |
| 10.35 | 1.73669 | 1.76158 | 6.62642 | 10.30295 |
| 10.4 | 2.05563 | 1.50699 | 6.20478 | 10.1109 |
| 10.45 | 2.41436 | 1.29007 | 5.78288 | 9.8949 |
| 10.5 | 2.80973 | 1.11253 | 5.3636 | 9.6563 |
| 10.55 | 3.23825 | 0.97573 | 4.94985 | 9.39658 |
| 10.6 | 3.69617 | 0.88074 | 4.54446 | 9.11736 |
| 10.65 | 4.17943 | 0.82829 | 4.15022 | 8.82037 |
| 10.7 | 4.6838 | 0.81878 | 3.76985 | 8.50744 |
| 10.75 | 5.20482 | 0.8523 | 3.40595 | 8.18051 |
| 10.8 | 5.73792 | 0.92859 | 3.06102 | 7.8416 |
| 10.85 | 6.2784 | 1.04704 | 2.73744 | 7.49281 |
| 10.9 | 6.82149 | 1.20675 | 2.43741 | 7.13628 |
| 10.95 | 7.36243 | 1.40647 | 2.163 | 6.77421 |
| 11 | 7.89645 | 1.64466 | 1.91609 | 6.40882 |
| 11.05 | 8.41884 | 1.91946 | 1.69837 | 6.04237 |
| 11.1 | 8.925 | 2.22874 | 1.51133 | 5.6771 |
| 11.15 | 9.41049 | 2.57011 | 1.35625 | 5.31525 |
| 11.2 | 9.87101 | 2.94091 | 1.2342 | 4.95903 |
| 11.25 | 10.30252 | 3.33826 | 1.146 | 4.61061 |
| 11.3 | 10.70122 | 3.75909 | 1.09227 | 4.27214 |
| 11.35 | 11.0636 | 4.20011 | 1.07337 | 3.94566 |
| 11.4 | 11.38646 | 4.65791 | 1.08943 | 3.63318 |
| 11.45 | 11.66696 | 5.12894 | 1.14033 | 3.33658 |
| 11.5 | 11.90263 | 5.60952 | 1.22574 | 3.05768 |
| 11.55 | 12.09139 | 6.09593 | 1.34507 | 2.79816 |
| 11.6 | 12.23159 | 6.58439 | 1.49749 | 2.5596 |
| 11.65 | 12.32198 | 7.07109 | 1.68198 | 2.34344 |
| 11.7 | 12.36178 | 7.55226 | 1.89725 | 2.151 |
| 11.75 | 12.35062 | 8.02416 | 2.14184 | 1.98343 |
| 11.8 | 12.28862 | 8.4831 | 2.41408 | 1.84176 |
| 11.85 | 12.1763 | 8.92553 | 2.7121 | 1.72683 |
| 11.9 | 12.01468 | 9.34799 | 3.03384 | 1.63935 |
| 11.95 | 11.80516 | 9.74721 | 3.37712 | 1.57983 |
| 12 | 11.54959 | 10.12008 | 3.73957 | 1.54864 |
| 12.05 | 11.25023 | 10.46368 | 4.1187 | 1.54597 |
| 12.1 | 10.9097 | 10.77536 | 4.5119 | 1.57183 |
| 12.15 | 10.53102 | 11.05267 | 4.91649 | 1.62607 |
| 12.2 | 10.11751 | 11.29346 | 5.32967 | 1.70836 |
| 12.25 | 9.67282 | 11.49586 | 5.7486 | 1.8182 |
| 12.3 | 9.20086 | 11.65829 | 6.1704 | 1.95492 |
| 12.35 | 8.70579 | 11.77949 | 6.59216 | 2.11772 |
| 12.4 | 8.19197 | 11.8585 | 7.01099 | 2.30559 |
| 12.45 | 7.66392 | 11.89472 | 7.42398 | 2.5174 |
| 12.5 | 7.12629 | 11.88786 | 7.82829 | 2.75187 |
| 12.55 | 6.58382 | 11.83797 | 8.22113 | 3.00759 |
| 12.6 | 6.04128 | 11.74545 | 8.59979 | 3.28299 |
| 12.65 | 5.50345 | 11.61102 | 8.96165 | 3.57641 |
| 12.7 | 4.97506 | 11.43571 | 9.30421 | 3.88606 |
| 12.75 | 4.46077 | 11.22091 | 9.6251 | 4.21007 |
| 12.8 | 3.9651 | 10.96827 | 9.9221 | 4.54645 |
| 12.85 | 3.49242 | 10.67978 | 10.19315 | 4.89317 |
| 12.9 | 3.04689 | 10.35766 | 10.43639 | 5.24809 |
| 12.95 | 2.63242 | 10.00444 | 10.65012 | 5.60906 |
| 13 | 2.25267 | 9.62287 | 10.83286 | 5.97386 |
| 13.05 | 1.91099 | 9.2159 | 10.98334 | 6.34026 |
| 13.1 | 1.61037 | 8.78672 | 11.10053 | 6.70601 |
| 13.15 | 1.35346 | 8.33865 | 11.18361 | 7.06887 |
| 13.2 | 1.14253 | 7.8752 | 11.23201 | 7.42661 |
| 13.25 | 0.97943 | 7.39995 | 11.24538 | 7.77702 |
| 13.3 | 0.86559 | 6.91661 | 11.22365 | 8.11793 |
| 13.35 | 0.80203 | 6.42893 | 11.16694 | 8.44726 |
| 13.4 | 0.78929 | 5.94072 | 11.07567 | 8.76296 |
| 13.45 | 0.82749 | 5.45575 | 10.95046 | 9.06308 |
| 13.5 | 0.91629 | 4.97781 | 10.79219 | 9.34576 |
| 13.55 | 1.05492 | 4.5106 | 10.60194 | 9.60925 |
| 13.6 | 1.24215 | 4.05776 | 10.38104 | 9.85192 |
| 13.65 | 1.47633 | 3.6228 | 10.13101 | 10.07226 |
| 13.7 | 1.7554 | 3.2091 | 9.85359 | 10.26889 |
| 13.75 | 2.07692 | 2.81987 | 9.55071 | 10.4406 |
| 13.8 | 2.43804 | 2.45813 | 9.22444 | 10.58632 |
| 13.85 | 2.83558 | 2.1267 | 8.87706 | 10.70513 |
| 13.9 | 3.26606 | 1.82815 | 8.51097 | 10.79631 |
| 13.95 | 3.72568 | 1.56478 | 8.12869 | 10.85928 |
| 14 | 4.21039 | 1.33865 | 7.73286 | 10.89365 |
| 14.05 | 4.71593 | 1.15151 | 7.32623 | 10.89921 |
| 14.1 | 5.23784 | 1.00482 | 6.91159 | 10.87592 |
| 14.15 | 5.77154 | 0.8997 | 6.4918 | 10.82392 |
| 14.2 | 6.31232 | 0.83698 | 6.06977 | 10.74354 |
| 14.25 | 6.85542 | 0.81714 | 5.64839 | 10.63529 |
| 14.3 | 7.39607 | 0.84033 | 5.23056 | 10.49983 |
| 14.35 | 7.92949 | 0.90638 | 4.81918 | 10.33801 |
| 14.4 | 8.451 | 1.01478 | 4.41705 | 10.15083 |
| 14.45 | 8.956 | 1.16468 | 4.02696 | 9.93947 |
| 14.5 | 9.44005 | 1.35492 | 3.65157 | 9.70523 |
| 14.55 | 9.89888 | 1.58402 | 3.29348 | 9.44957 |
| 14.6 | 10.32845 | 1.8502 | 2.95512 | 9.17408 |
| 14.65 | 10.72498 | 2.15141 | 2.63884 | 8.88045 |
| 14.7 | 11.08497 | 2.4853 | 2.34679 | 8.57053 |
| 14.75 | 11.40526 | 2.84929 | 2.08097 | 8.24621 |
| 14.8 | 11.68303 | 3.24054 | 1.84322 | 7.90951 |
| 14.85 | 11.91582 | 3.65601 | 1.63516 | 7.5625 |
| 14.9 | 12.10159 | 4.09249 | 1.45821 | 7.20733 |
| 14.95 | 12.23871 | 4.54659 | 1.31359 | 6.84618 |
| 15 | 12.32596 | 5.01476 | 1.20228 | 6.48127 |
