# Supplementary material for: Analysis of sinusoidal post-buckling deformation of horizontal coiled tubing with initial residual bending
Source: PLoS One. 2024 May 14;19(5):e0301610. doi: 10.1371/journal.pone.0301610 (PMC11093391; doi:10.1371/journal.pone.0301610)
Supplement: S1 File — (ZIP) [file pone.0301610.s001.zip › The values used to build graphs - Fig 11 (b).docx]

## The values used to build graphs

The minimal data set of the original data for plotting curves in Fig 11 (b) is as follows:

| x-axis | m=5 | m=15 | m=25 | m=35 |
| --- | --- | --- | --- | --- |
| 0 | 1.55616 | 1.66074 | 1.88614 | 2.26146 |
| 0.05 | 1.77327 | 1.89123 | 2.14367 | 2.56015 |
| 0.1 | 2.42329 | 2.58133 | 2.91477 | 3.45461 |
| 0.15 | 3.50223 | 3.72689 | 4.19501 | 4.94 |
| 0.2 | 5.00351 | 5.32107 | 5.97704 | 7.0083 |
| 0.25 | 6.91794 | 7.35432 | 8.25064 | 9.64833 |
| 0.3 | 9.23383 | 9.81448 | 11.00274 | 12.84582 |
| 0.35 | 11.93702 | 12.68681 | 14.21755 | 16.58348 |
| 0.4 | 15.01097 | 15.95413 | 17.87659 | 20.84113 |
| 0.45 | 18.43691 | 19.59688 | 21.95886 | 25.59574 |
| 0.5 | 22.19388 | 23.59324 | 26.44091 | 30.82161 |
| 0.55 | 26.25892 | 27.9193 | 31.29699 | 36.4905 |
| 0.6 | 30.60716 | 32.54915 | 36.49922 | 42.57175 |
| 0.65 | 35.21202 | 37.45507 | 42.0177 | 49.03248 |
| 0.7 | 40.04535 | 42.60768 | 47.82074 | 55.83777 |
| 0.75 | 45.07757 | 47.97614 | 53.87499 | 62.9508 |
| 0.8 | 50.27792 | 53.52827 | 60.14567 | 70.33312 |
| 0.85 | 55.61458 | 59.23085 | 66.59675 | 77.9448 |
| 0.9 | 61.05491 | 65.04969 | 73.19114 | 85.74465 |
| 0.95 | 66.56563 | 70.94995 | 79.89095 | 93.69049 |
| 1 | 72.11302 | 76.89629 | 86.65767 | 101.7393 |
| 1.05 | 77.66314 | 82.85306 | 93.45241 | 109.8476 |
| 1.1 | 83.18203 | 88.78458 | 100.2361 | 117.9715 |
| 1.15 | 88.63591 | 94.65531 | 106.9697 | 126.0669 |
| 1.2 | 93.9914 | 100.4301 | 113.6146 | 134.0901 |
| 1.25 | 99.21573 | 106.0742 | 120.1324 | 141.9977 |
| 1.3 | 104.2769 | 111.554 | 126.4858 | 149.7469 |
| 1.35 | 109.144 | 116.8364 | 132.6382 | 157.2956 |
| 1.4 | 113.7871 | 121.8899 | 138.5541 | 164.603 |
| 1.45 | 118.1779 | 126.6842 | 144.1997 | 171.6296 |
| 1.5 | 122.2894 | 131.1905 | 149.5423 | 178.3372 |
| 1.55 | 126.0966 | 135.3818 | 154.5512 | 184.6896 |
| 1.6 | 129.576 | 139.233 | 159.1977 | 190.6524 |
| 1.65 | 132.7063 | 142.7209 | 163.4549 | 196.1931 |
| 1.7 | 135.4685 | 145.8246 | 167.2984 | 201.2819 |
| 1.75 | 137.8455 | 148.5256 | 170.7061 | 205.8912 |
| 1.8 | 139.8228 | 150.8076 | 173.6584 | 209.9959 |
| 1.85 | 141.3884 | 152.657 | 176.1382 | 213.5739 |
| 1.9 | 142.5325 | 154.0626 | 178.1312 | 216.6057 |
| 1.95 | 143.2483 | 155.016 | 179.6261 | 219.0749 |
| 2 | 143.5313 | 155.5116 | 180.6142 | 220.9681 |
| 2.05 | 143.3798 | 155.5462 | 181.0898 | 222.2752 |
| 2.1 | 142.7947 | 155.1198 | 181.0502 | 222.9889 |
| 2.15 | 141.7795 | 154.2348 | 180.4956 | 223.1054 |
| 2.2 | 140.3406 | 152.8966 | 179.4292 | 222.6241 |
| 2.25 | 138.4868 | 151.1132 | 177.8571 | 221.5476 |
| 2.3 | 136.2293 | 148.8954 | 175.7885 | 219.8818 |
| 2.35 | 133.582 | 146.2563 | 173.2351 | 217.6356 |
| 2.4 | 130.5612 | 143.2119 | 170.2117 | 214.8212 |
| 2.45 | 127.1853 | 139.7804 | 166.7357 | 211.4539 |
| 2.5 | 123.475 | 135.9824 | 162.8271 | 207.5519 |
| 2.55 | 119.453 | 131.8406 | 158.5084 | 203.1365 |
| 2.6 | 115.144 | 127.3799 | 153.8044 | 198.2314 |
| 2.65 | 110.5743 | 122.6271 | 148.7423 | 192.8633 |
| 2.7 | 105.772 | 117.6106 | 143.3511 | 187.0613 |
| 2.75 | 100.7663 | 112.3605 | 137.6619 | 180.8567 |
| 2.8 | 95.58803 | 106.9083 | 131.7073 | 174.2833 |
| 2.85 | 90.26882 | 101.2867 | 125.5218 | 167.3765 |
| 2.9 | 84.84122 | 95.52939 | 119.1407 | 160.1739 |
| 2.95 | 79.33846 | 89.67085 | 112.6009 | 152.7144 |
| 3 | 73.79421 | 83.74619 | 105.9399 | 145.0385 |
| 3.05 | 68.24241 | 77.79093 | 99.19603 | 137.1876 |
| 3.1 | 62.71702 | 71.84075 | 92.40811 | 129.2043 |
| 3.15 | 57.25184 | 65.93131 | 85.61515 | 121.1318 |
| 3.2 | 51.88032 | 60.098 | 78.85619 | 113.0138 |
| 3.25 | 46.63532 | 54.37577 | 72.17008 | 104.8942 |
| 3.3 | 41.54891 | 48.7989 | 65.59526 | 96.81699 |
| 3.35 | 36.6522 | 43.40078 | 59.16952 | 88.82587 |
| 3.4 | 31.97515 | 38.21376 | 52.92978 | 80.96406 |
| 3.45 | 27.54635 | 33.26888 | 46.9119 | 73.2741 |
| 3.5 | 23.39288 | 28.59577 | 41.15045 | 65.79759 |
| 3.55 | 19.54015 | 24.2224 | 35.67855 | 58.57497 |
| 3.6 | 16.01171 | 20.17495 | 30.52761 | 51.64529 |
| 3.65 | 12.82913 | 16.47766 | 25.72724 | 45.04603 |
| 3.7 | 10.01187 | 13.15267 | 21.305 | 38.81288 |
| 3.75 | 7.57716 | 10.21987 | 17.28629 | 32.97954 |
| 3.8 | 5.53986 | 7.69682 | 13.69421 | 27.57755 |
| 3.85 | 3.91244 | 5.59863 | 10.54936 | 22.6361 |
| 3.9 | 2.70485 | 3.93784 | 7.86982 | 18.18192 |
| 3.95 | 1.92447 | 2.72441 | 5.67098 | 14.23907 |
| 4 | 1.57606 | 1.9656 | 3.96545 | 10.82888 |
| 4.05 | 1.66176 | 1.66594 | 2.76303 | 7.96976 |
| 4.1 | 2.18104 | 1.82723 | 2.07063 | 5.67718 |
| 4.15 | 3.13073 | 2.44851 | 1.89222 | 3.96352 |
| 4.2 | 4.50503 | 3.52606 | 2.22883 | 2.83804 |
| 4.25 | 6.29553 | 5.05342 | 3.07852 | 2.30683 |
| 4.3 | 8.49129 | 7.02145 | 4.43642 | 2.37276 |
| 4.35 | 11.07888 | 9.41839 | 6.29472 | 3.03546 |
| 4.4 | 14.04249 | 12.22987 | 8.64276 | 4.29137 |
| 4.45 | 17.36401 | 15.43907 | 11.46705 | 6.13368 |
| 4.5 | 21.02311 | 19.02678 | 14.75138 | 8.55245 |
| 4.55 | 24.99744 | 22.97152 | 18.47688 | 11.53461 |
| 4.6 | 29.26269 | 27.24968 | 22.62216 | 15.06403 |
| 4.65 | 33.79279 | 31.83564 | 27.16341 | 19.12164 |
| 4.7 | 38.56002 | 36.70194 | 32.07455 | 23.68552 |
| 4.75 | 43.53524 | 41.81945 | 37.32736 | 28.73097 |
| 4.8 | 48.68801 | 47.15752 | 42.89167 | 34.23075 |
| 4.85 | 53.98682 | 52.68417 | 48.73551 | 40.1551 |
| 4.9 | 59.39926 | 58.36631 | 54.82532 | 46.47201 |
| 4.95 | 64.89222 | 64.16991 | 61.12609 | 53.14732 |
| 5 | 70.43208 | 70.06019 | 67.60162 | 60.14492 |
| 5.05 | 75.98496 | 76.00188 | 74.21469 | 67.42698 |
| 5.1 | 81.51688 | 81.95936 | 80.9273 | 74.95411 |
| 5.15 | 86.99398 | 87.89695 | 87.70086 | 82.6856 |
| 5.2 | 92.38275 | 93.77906 | 94.49644 | 90.57963 |
| 5.25 | 97.65019 | 99.57044 | 101.275 | 98.59349 |
| 5.3 | 102.7641 | 105.2364 | 107.9975 | 106.6838 |
| 5.35 | 107.6931 | 110.7429 | 114.6253 | 114.8068 |
| 5.4 | 112.4071 | 116.0571 | 121.1203 | 122.9186 |
| 5.45 | 116.8771 | 121.1469 | 127.4451 | 130.9751 |
| 5.5 | 121.0759 | 125.982 | 133.5634 | 138.9329 |
| 5.55 | 124.9778 | 130.5333 | 139.44 | 146.7488 |
| 5.6 | 128.5587 | 134.7736 | 145.0411 | 154.3806 |
| 5.65 | 131.7968 | 138.6773 | 150.3343 | 161.7868 |
| 5.7 | 134.6723 | 142.2212 | 155.2894 | 168.9274 |
| 5.75 | 137.1676 | 145.3839 | 159.8778 | 175.7637 |
| 5.8 | 139.2673 | 148.1464 | 164.073 | 182.2588 |
| 5.85 | 140.9586 | 150.4923 | 167.8509 | 188.3773 |
| 5.9 | 142.2312 | 152.4074 | 171.1898 | 194.0862 |
| 5.95 | 143.0772 | 153.8802 | 174.0705 | 199.3546 |
| 6 | 143.4915 | 154.902 | 176.4764 | 204.1538 |
| 6.05 | 143.4715 | 155.4665 | 178.3936 | 208.4579 |
| 6.1 | 143.0174 | 155.5704 | 179.8111 | 212.2436 |
| 6.15 | 142.1319 | 155.2131 | 180.7207 | 215.4904 |
| 6.2 | 140.8204 | 154.3968 | 181.1173 | 218.1806 |
| 6.25 | 139.0911 | 153.1262 | 180.9984 | 220.2996 |
| 6.3 | 136.9544 | 151.409 | 180.3649 | 221.8361 |
| 6.35 | 134.4235 | 149.2556 | 179.2203 | 222.7816 |
| 6.4 | 131.5139 | 146.6789 | 177.5713 | 223.1311 |
| 6.45 | 128.2434 | 143.6942 | 175.4273 | 222.8826 |
| 6.5 | 124.632 | 140.3196 | 172.8007 | 222.0376 |
| 6.55 | 120.7019 | 136.5752 | 169.7065 | 220.6005 |
| 6.6 | 116.477 | 132.4835 | 166.1627 | 218.5791 |
| 6.65 | 111.9834 | 128.069 | 162.1896 | 215.9845 |
| 6.7 | 107.2484 | 123.3583 | 157.81 | 212.8307 |
| 6.75 | 102.3011 | 118.3795 | 153.0492 | 209.1347 |
| 6.8 | 97.17175 | 113.1625 | 147.9346 | 204.9166 |
| 6.85 | 91.89179 | 107.7386 | 142.4955 | 200.1993 |
| 6.9 | 86.49352 | 102.1403 | 136.7634 | 195.0083 |
| 6.95 | 81.00997 | 96.4012 | 130.7711 | 189.3718 |
| 7 | 75.47471 | 90.55562 | 124.5531 | 183.3202 |
| 7.05 | 69.9216 | 84.63863 | 118.1452 | 176.8864 |
| 7.1 | 64.38464 | 78.68569 | 111.5843 | 170.1051 |
| 7.15 | 58.89769 | 72.73247 | 104.9081 | 163.0133 |
| 7.2 | 53.49432 | 66.81465 | 98.15494 | 155.6492 |
| 7.25 | 48.2076 | 60.96766 | 91.3637 | 148.0527 |
| 7.3 | 43.06985 | 55.22655 | 84.57343 | 140.2649 |
| 7.35 | 38.11251 | 49.62569 | 77.82314 | 132.3281 |
| 7.4 | 33.36589 | 44.19865 | 71.15165 | 124.2852 |
| 7.45 | 28.85901 | 38.97791 | 64.5973 | 116.1797 |
| 7.5 | 24.61945 | 33.99475 | 58.19776 | 108.0555 |
| 7.55 | 20.67312 | 29.279 | 51.98981 | 99.95657 |
| 7.6 | 17.04416 | 24.8589 | 46.00911 | 91.92673 |
| 7.65 | 13.75474 | 20.76092 | 40.29004 | 84.00943 |
| 7.7 | 10.82498 | 17.00959 | 34.86546 | 76.24751 |
| 7.75 | 8.27279 | 13.62737 | 29.76651 | 68.68295 |
| 7.8 | 6.11378 | 10.6345 | 25.0225 | 61.35666 |
| 7.85 | 4.36113 | 8.0489 | 20.66067 | 54.30829 |
| 7.9 | 3.02557 | 5.88605 | 16.70607 | 47.57593 |
| 7.95 | 2.11526 | 4.15889 | 13.18143 | 41.196 |
| 8 | 1.63575 | 2.87776 | 10.10697 | 35.20299 |
| 8.05 | 1.58999 | 2.05032 | 7.50036 | 29.62931 |
| 8.1 | 1.97825 | 1.68154 | 5.37656 | 24.50509 |
| 8.15 | 2.79815 | 1.77361 | 3.74777 | 19.85803 |
| 8.2 | 4.0447 | 2.32599 | 2.62335 | 15.71324 |
| 8.25 | 5.71026 | 3.33537 | 2.00974 | 12.09314 |
| 8.3 | 7.78466 | 4.79571 | 1.91047 | 9.01728 |
| 8.35 | 10.25521 | 6.69826 | 2.32612 | 6.50229 |
| 8.4 | 13.10682 | 9.03165 | 3.25429 | 4.56177 |
| 8.45 | 16.32205 | 11.7819 | 4.68965 | 3.2062 |
| 8.5 | 19.88124 | 14.93255 | 6.62397 | 2.4429 |
| 8.55 | 23.76263 | 18.46474 | 9.04613 | 2.27601 |
| 8.6 | 27.9425 | 22.35733 | 11.94223 | 2.70642 |
| 8.65 | 32.39529 | 26.58701 | 15.29564 | 3.73181 |
| 8.7 | 37.09375 | 31.12846 | 19.08709 | 5.34663 |
| 8.75 | 42.00917 | 35.95449 | 23.29482 | 7.54216 |
| 8.8 | 47.11148 | 41.03619 | 27.89466 | 10.30653 |
| 8.85 | 52.36948 | 46.34315 | 32.86018 | 13.62481 |
| 8.9 | 57.75099 | 51.84357 | 38.16286 | 17.47906 |
| 8.95 | 63.2231 | 57.50452 | 43.77224 | 21.84844 |
| 9 | 68.75234 | 63.29208 | 49.6561 | 26.70935 |
| 9.05 | 74.30487 | 69.17159 | 55.78063 | 32.03549 |
| 9.1 | 79.84671 | 75.10782 | 62.11063 | 37.79809 |
| 9.15 | 85.34396 | 81.06521 | 68.60973 | 43.96598 |
| 9.2 | 90.76297 | 87.00806 | 75.24059 | 50.50582 |
| 9.25 | 96.07058 | 92.90076 | 81.96509 | 57.38224 |
| 9.3 | 101.2343 | 98.708 | 88.74457 | 64.55806 |
| 9.35 | 106.2225 | 104.395 | 95.54006 | 71.99447 |
| 9.4 | 111.0047 | 109.9276 | 102.3125 | 79.65125 |
| 9.45 | 115.5515 | 115.2727 | 109.023 | 87.48698 |
| 9.5 | 119.8352 | 120.3982 | 115.6328 | 95.45926 |
| 9.55 | 123.8296 | 125.2734 | 122.1041 | 103.525 |
| 9.6 | 127.51 | 129.8691 | 128.3995 | 111.6405 |
| 9.65 | 130.8541 | 134.1578 | 134.483 | 119.7618 |
| 9.7 | 133.8413 | 138.1136 | 140.3194 | 127.8451 |
| 9.75 | 136.4534 | 141.7129 | 145.8752 | 135.8465 |
| 9.8 | 138.6743 | 144.9341 | 151.1184 | 143.7228 |
| 9.85 | 140.4904 | 147.7578 | 156.019 | 151.4313 |
| 9.9 | 141.8907 | 150.1672 | 160.5486 | 158.9302 |
| 9.95 | 142.8665 | 152.1478 | 164.6812 | 166.179 |
| 10 | 143.4118 | 153.6877 | 168.393 | 173.1384 |
| 10.05 | 143.5234 | 154.7776 | 171.6627 | 179.7708 |
| 10.1 | 143.2005 | 155.4111 | 174.4714 | 186.0401 |
| 10.15 | 142.4451 | 155.5843 | 176.803 | 191.9124 |
| 10.2 | 141.2619 | 155.2962 | 178.6441 | 197.3559 |
| 10.25 | 139.658 | 154.5485 | 179.984 | 202.3412 |
| 10.3 | 137.6434 | 153.3457 | 180.8151 | 206.8412 |
| 10.35 | 135.2303 | 151.695 | 181.1325 | 210.8315 |
| 10.4 | 132.4336 | 149.6063 | 180.9345 | 214.2905 |
| 10.45 | 129.2703 | 147.0922 | 180.2221 | 217.1994 |
| 10.5 | 125.7598 | 144.1677 | 178.9995 | 219.5426 |
| 10.55 | 121.9237 | 140.8504 | 177.2738 | 221.3073 |
| 10.6 | 117.7854 | 137.1601 | 175.0547 | 222.4839 |
| 10.65 | 113.3703 | 133.1191 | 172.3552 | 223.066 |
| 10.7 | 108.7053 | 128.7514 | 169.1908 | 223.0506 |
| 10.75 | 103.8192 | 124.0834 | 165.5796 | 222.4376 |
| 10.8 | 98.74166 | 119.143 | 161.5425 | 221.2304 |
| 10.85 | 93.50392 | 113.9599 | 157.1026 | 219.4356 |
| 10.9 | 88.138 | 108.565 | 152.2856 | 217.0629 |
| 10.95 | 82.67674 | 102.9908 | 147.1192 | 214.1251 |
| 11 | 77.15357 | 97.2706 | 141.633 | 210.6381 |
| 11.05 | 71.60228 | 91.43878 | 135.8587 | 206.621 |
| 11.1 | 66.05684 | 85.53026 | 129.8294 | 202.0953 |
| 11.15 | 60.5512 | 79.58045 | 123.5799 | 197.0857 |
| 11.2 | 55.11902 | 73.62499 | 117.1461 | 191.6192 |
| 11.25 | 49.79354 | 67.69958 | 110.565 | 185.7256 |
| 11.3 | 44.60734 | 61.83971 | 103.8745 | 179.4367 |
| 11.35 | 39.59213 | 56.08049 | 97.11295 | 172.7866 |
| 11.4 | 34.7786 | 50.45641 | 90.31932 | 165.8113 |
| 11.45 | 30.19618 | 45.00116 | 83.53265 | 158.5486 |
| 11.5 | 25.8729 | 39.74742 | 76.79195 | 151.0378 |
| 11.55 | 21.83518 | 34.72664 | 70.13598 | 143.3196 |
| 11.6 | 18.10772 | 29.9689 | 63.60299 | 135.4358 |
| 11.65 | 14.71331 | 25.50268 | 57.23052 | 127.4291 |
| 11.7 | 11.6727 | 21.35471 | 51.0552 | 119.3428 |
| 11.75 | 9.00447 | 17.54985 | 45.11251 | 111.2206 |
| 11.8 | 6.72494 | 14.11085 | 39.43659 | 103.1066 |
| 11.85 | 4.84805 | 11.05832 | 34.06006 | 95.04469 |
| 11.9 | 3.38527 | 8.41052 | 29.01379 | 87.0784 |
| 11.95 | 2.34553 | 6.1833 | 24.32679 | 79.25089 |
| 12 | 1.7352 | 4.38999 | 20.02596 | 71.6045 |
| 12.05 | 1.558 | 3.04133 | 16.13601 | 64.18058 |
| 12.1 | 1.81501 | 2.14539 | 12.67928 | 57.01929 |
| 12.15 | 2.50468 | 1.70753 | 9.67563 | 50.15936 |
| 12.2 | 3.62277 | 1.73037 | 7.1423 | 43.63789 |
| 12.25 | 5.16246 | 2.21378 | 5.09384 | 37.49014 |
| 12.3 | 7.11434 | 3.15485 | 3.54201 | 31.74935 |
| 12.35 | 9.46647 | 4.54797 | 2.49573 | 26.44656 |
| 12.4 | 12.20447 | 6.38479 | 1.96099 | 21.61043 |
| 12.45 | 15.31161 | 8.65431 | 1.94088 | 17.2671 |
| 12.5 | 18.7689 | 11.34296 | 2.43551 | 13.44006 |
| 12.55 | 22.55518 | 14.43463 | 3.44203 | 10.14999 |
| 12.6 | 26.64733 | 17.91083 | 4.95467 | 7.41467 |
| 12.65 | 31.0203 | 21.75074 | 6.96474 | 5.24888 |
| 12.7 | 35.64737 | 25.93137 | 9.46069 | 3.66432 |
| 12.75 | 40.50024 | 30.4277 | 12.42821 | 2.66957 |
| 12.8 | 45.54923 | 35.2128 | 15.85024 | 2.26999 |
| 12.85 | 50.76345 | 40.25802 | 19.70713 | 2.46775 |
| 12.9 | 56.11102 | 45.53315 | 23.97674 | 3.26178 |
| 12.95 | 61.55922 | 51.0066 | 28.63454 | 4.64778 |
| 13 | 67.07473 | 56.64558 | 33.65377 | 6.61827 |
| 13.05 | 72.62379 | 62.41633 | 39.0056 | 9.16259 |
| 13.1 | 78.17245 | 68.28427 | 44.6593 | 12.267 |
| 13.15 | 83.68677 | 74.21425 | 50.58237 | 15.91472 |
| 13.2 | 89.13298 | 80.17074 | 56.74079 | 20.08603 |
| 13.25 | 94.47778 | 86.11805 | 63.09917 | 24.75839 |
| 13.3 | 99.68842 | 92.02054 | 69.62097 | 29.90653 |
| 13.35 | 104.733 | 97.84285 | 76.2687 | 35.50264 |
| 13.4 | 109.5807 | 103.5501 | 83.00417 | 41.51645 |
| 13.45 | 114.2018 | 109.108 | 89.78865 | 47.91545 |
| 13.5 | 118.568 | 114.4833 | 96.58315 | 54.66504 |
| 13.55 | 122.6526 | 119.6438 | 103.3486 | 61.72873 |
| 13.6 | 126.4305 | 124.5585 | 110.0461 | 69.0683 |
| 13.65 | 129.8787 | 129.198 | 116.6371 | 76.64408 |
| 13.7 | 132.976 | 133.5344 | 123.0837 | 84.41507 |
| 13.75 | 135.7034 | 137.5418 | 129.3489 | 92.33923 |
| 13.8 | 138.0442 | 141.196 | 135.3967 | 100.3737 |
| 13.85 | 139.9842 | 144.4753 | 141.1921 | 108.475 |
| 13.9 | 141.5113 | 147.3599 | 146.7019 | 116.5993 |
| 13.95 | 142.6163 | 149.8325 | 151.8944 | 124.7027 |
| 14 | 143.2924 | 151.8783 | 156.7397 | 132.7412 |
| 14.05 | 143.5355 | 153.485 | 161.21 | 140.6713 |
| 14.1 | 143.344 | 154.6429 | 165.2794 | 148.4502 |
| 14.15 | 142.7192 | 155.3453 | 168.9246 | 156.0357 |
| 14.2 | 141.6648 | 155.5877 | 172.1247 | 163.3867 |
| 14.25 | 140.1873 | 155.3688 | 174.8611 | 170.4635 |
| 14.3 | 138.2959 | 154.6899 | 177.1181 | 177.2276 |
| 14.35 | 136.0019 | 153.5551 | 178.8828 | 183.6425 |
| 14.4 | 133.3196 | 151.9711 | 180.145 | 189.6734 |
| 14.45 | 130.2653 | 149.9474 | 180.8974 | 195.2877 |
| 14.5 | 126.8578 | 147.4963 | 181.1356 | 200.4549 |
| 14.55 | 123.1178 | 144.6324 | 180.8584 | 205.147 |
| 14.6 | 119.0684 | 141.3728 | 180.0673 | 209.3386 |
| 14.65 | 114.7343 | 137.7372 | 178.7669 | 213.0071 |
| 14.7 | 110.142 | 133.7473 | 176.9646 | 216.1324 |
| 14.75 | 105.3197 | 129.4271 | 174.6708 | 218.6977 |
| 14.8 | 100.2969 | 124.8024 | 171.8988 | 220.6891 |
| 14.85 | 95.1043 | 119.9011 | 168.6645 | 222.0958 |
| 14.9 | 89.77374 | 114.7524 | 164.9864 | 222.9101 |
| 14.95 | 84.33784 | 109.3873 | 160.8859 | 223.1277 |
| 15 | 78.82985 | 103.8379 | 156.3863 | 222.7474 |
| 15.05 | 73.28349 | 98.13748 | 151.5138 | 221.7711 |
| 15.1 | 67.7327 | 92.32021 | 146.2962 | 220.2043 |
| 15.15 | 62.21144 | 86.42096 | 140.7637 | 218.0554 |
| 15.2 | 56.75349 | 80.47507 | 134.948 | 215.3361 |
| 15.25 | 51.39224 | 74.51819 | 128.8826 | 212.061 |
| 15.3 | 46.16049 | 68.58599 | 122.6024 | 208.248 |
| 15.35 | 41.09024 | 62.71403 | 116.1436 | 203.9177 |
| 15.4 | 36.2125 | 56.93747 | 109.5432 | 199.0936 |
| 15.45 | 31.55711 | 51.29093 | 102.8392 | 193.8018 |
| 15.5 | 27.15252 | 45.80822 | 96.07019 | 188.0709 |
| 15.55 | 23.02568 | 40.52218 | 89.27509 | 181.9321 |
| 15.6 | 19.20181 | 35.46447 | 82.49296 | 175.4186 |
| 15.65 | 15.70431 | 30.66538 | 75.76277 | 168.5656 |
| 15.7 | 12.55454 | 26.15363 | 69.12322 | 161.4103 |
| 15.75 | 9.77177 | 21.95624 | 62.61248 | 153.9914 |
| 15.8 | 7.37302 | 18.09836 | 56.26795 | 146.3491 |
| 15.85 | 5.37293 | 14.60306 | 50.12609 | 138.5249 |
| 15.9 | 3.78374 | 11.49127 | 44.22221 | 130.5609 |
| 15.95 | 2.61516 | 8.78162 | 38.59021 | 122.5004 |
| 16 | 1.87434 | 6.49034 | 33.26246 | 114.387 |
| 16.05 | 1.56581 | 4.63113 | 28.26956 | 106.2646 |
| 16.1 | 1.69144 | 3.21512 | 23.6402 | 98.17718 |
| 16.15 | 2.25047 | 2.25079 | 19.40095 | 90.16845 |
| 16.2 | 3.23949 | 1.74392 | 15.57618 | 82.28179 |
| 16.25 | 4.65245 | 1.69752 | 12.18784 | 74.55984 |
| 16.3 | 6.48071 | 2.11189 | 9.2554 | 67.04438 |
| 16.35 | 8.71309 | 2.98454 | 6.7957 | 59.77606 |
| 16.4 | 11.33596 | 4.31024 | 4.82285 | 52.7942 |
| 16.45 | 14.33327 | 6.08107 | 3.34819 | 46.13655 |
| 16.5 | 17.68671 | 8.28643 | 2.38019 | 39.8391 |
| 16.55 | 21.37577 | 10.91311 | 1.92441 | 33.93592 |
| 16.6 | 25.37789 | 13.94539 | 1.98345 | 28.45891 |
| 16.65 | 29.66861 | 17.36512 | 2.55699 | 23.43769 |
| 16.7 | 34.22169 | 21.15183 | 3.64172 | 18.89939 |
| 16.75 | 39.00929 | 25.28285 | 5.23143 | 14.86856 |
| 16.8 | 44.00212 | 29.73345 | 7.31698 | 11.36697 |
| 16.85 | 49.16965 | 34.47698 | 9.8864 | 8.41355 |
| 16.9 | 54.48028 | 39.48503 | 12.92492 | 6.02427 |
| 16.95 | 59.90151 | 44.72762 | 16.41512 | 4.21204 |
| 17 | 65.40019 | 50.17336 | 20.33692 | 2.98664 |
| 17.05 | 70.94267 | 55.78962 | 24.66783 | 2.35471 |
| 17.1 | 76.49504 | 61.54277 | 29.38295 | 2.31966 |
| 17.15 | 82.02333 | 67.39835 | 34.45521 | 2.88167 |
| 17.2 | 87.4937 | 73.32127 | 39.85548 | 4.03772 |
| 17.25 | 92.87268 | 79.27605 | 45.55271 | 5.78154 |
| 17.3 | 98.12734 | 85.22702 | 51.5142 | 8.10373 |
| 17.35 | 103.2255 | 91.13851 | 57.70567 | 10.99172 |
| 17.4 | 108.136 | 96.97509 | 64.09156 | 14.42992 |
| 17.45 | 112.8287 | 102.7018 | 70.63517 | 18.39973 |
| 17.5 | 117.275 | 108.2843 | 77.29889 | 22.8797 |
| 17.55 | 121.4475 | 113.6891 | 84.04442 | 27.84562 |
| 17.6 | 125.3208 | 118.8839 | 90.83297 | 33.27064 |
| 17.65 | 128.8711 | 123.8374 | 97.62554 | 39.12543 |
| 17.7 | 132.0767 | 128.52 | 104.3831 | 45.37833 |
| 17.75 | 134.9179 | 132.9037 | 111.0667 | 51.99555 |
| 17.8 | 137.3774 | 136.962 | 117.638 | 58.9413 |
| 17.85 | 139.44 | 140.6708 | 124.0591 | 66.17801 |
| 17.9 | 141.0932 | 144.0076 | 130.2932 | 73.66656 |
| 17.95 | 142.3269 | 146.9526 | 136.3044 | 81.36642 |
| 18 | 143.1334 | 149.4881 | 142.0581 | 89.23596 |
| 18.05 | 143.5078 | 151.5988 | 147.5212 | 97.23259 |
| 18.1 | 143.4478 | 153.2721 | 152.6622 | 105.3131 |
| 18.15 | 142.9539 | 154.498 | 157.4517 | 113.4336 |
| 18.2 | 142.0289 | 155.2691 | 161.8619 | 121.5504 |
| 18.25 | 140.6787 | 155.5807 | 165.8677 | 129.6193 |
| 18.3 | 138.9114 | 155.4311 | 169.4458 | 137.5968 |
| 18.35 | 136.7379 | 154.8211 | 172.5757 | 145.4397 |
| 18.4 | 134.1715 | 153.7543 | 175.2394 | 153.1055 |
| 18.45 | 131.228 | 152.2372 | 177.4216 | 160.5527 |
| 18.5 | 127.9252 | 150.2789 | 179.1096 | 167.741 |
| 18.55 | 124.2836 | 147.8911 | 180.2939 | 174.6314 |
| 18.6 | 120.3253 | 145.0881 | 180.9675 | 181.1866 |
| 18.65 | 116.0746 | 141.8868 | 181.1265 | 187.3712 |
| 18.7 | 111.5576 | 138.3063 | 180.7702 | 193.1515 |
| 18.75 | 106.8019 | 134.3681 | 179.9005 | 198.4964 |
| 18.8 | 101.8365 | 130.0959 | 178.5224 | 203.3768 |
| 18.85 | 96.69203 | 125.5152 | 176.6438 | 207.7663 |
| 18.9 | 91.39983 | 120.6536 | 174.2756 | 211.6412 |
| 18.95 | 85.99233 | 115.5401 | 171.4315 | 214.9803 |
| 19 | 80.50262 | 110.2055 | 168.1277 | 217.7657 |
| 19.05 | 74.9643 | 104.6816 | 164.3833 | 219.9821 |
| 19.1 | 69.41126 | 99.00172 | 160.2198 | 221.6177 |
| 19.15 | 63.87748 | 93.19979 | 155.6612 | 222.6636 |
| 19.2 | 58.39682 | 87.3106 | 150.7338 | 223.114 |
| 19.25 | 53.00281 | 81.36945 | 145.4658 | 222.9666 |
| 19.3 | 47.72845 | 75.41194 | 139.8877 | 222.2221 |
| 19.35 | 42.606 | 69.47376 | 134.0314 | 220.8846 |
| 19.4 | 37.66678 | 63.5905 | 127.9307 | 218.9613 |
| 19.45 | 32.94102 | 57.7974 | 121.6207 | 216.4627 |
| 19.5 | 28.4576 | 52.12915 | 115.1377 | 213.4022 |
| 19.55 | 24.24394 | 46.61972 | 108.5189 | 209.7966 |
| 19.6 | 20.32582 | 41.3021 | 101.8024 | 205.6652 |
| 19.65 | 16.72718 | 36.20814 | 95.02683 | 201.0305 |
| 19.7 | 13.47003 | 31.36833 | 88.23118 | 195.9177 |
| 19.75 | 10.57428 | 26.81167 | 81.45449 | 190.3543 |
| 19.8 | 8.05763 | 22.56543 | 74.73573 | 184.3707 |
| 19.85 | 5.93547 | 18.65504 | 68.11351 | 177.999 |
| 19.9 | 4.22077 | 15.10391 | 61.62589 | 171.274 |
| 19.95 | 2.92401 | 11.93329 | 55.31015 | 164.232 |
| 20 | 2.05311 | 9.16217 | 49.2026 | 156.9111 |
