# Supplementary material for: Analysis of sinusoidal post-buckling deformation of horizontal coiled tubing with initial residual bending
Source: PLoS One. 2024 May 14;19(5):e0301610. doi: 10.1371/journal.pone.0301610 (PMC11093391; doi:10.1371/journal.pone.0301610)
Supplement: S1 File — (ZIP) [file pone.0301610.s001.zip › The values used to build graphs - Fig 11 (a).docx]

## The values used to build graphs

The minimal data set of the original data for plotting curves in Fig 11 (a) is as follows:

| x-axis | *A*_n_ = 0.2 | *A*_n_ = 0.4 | *A*_n_ = 0.6 | *A*_n_ = 0.8 |
| --- | --- | --- | --- | --- |
| 0 | 0.88502 | 0.88486 | 0.88458 | 0.88419 |
| 0.05 | 0.88773 | 0.89361 | 0.93599 | 1.01476 |
| 0.1 | 0.89583 | 0.91977 | 1.08972 | 1.40524 |
| 0.15 | 0.90925 | 0.9631 | 1.34432 | 2.05192 |
| 0.2 | 0.92786 | 1.02318 | 1.69737 | 2.94865 |
| 0.25 | 0.95148 | 1.09945 | 2.14552 | 4.08693 |
| 0.3 | 0.97989 | 1.19118 | 2.68452 | 5.45595 |
| 0.35 | 1.01281 | 1.2975 | 3.30923 | 7.04271 |
| 0.4 | 1.04994 | 1.4174 | 4.01374 | 8.83215 |
| 0.45 | 1.09093 | 1.54973 | 4.79135 | 10.80729 |
| 0.5 | 1.13537 | 1.69326 | 5.63469 | 12.94937 |
| 0.55 | 1.18286 | 1.8466 | 6.53573 | 15.23805 |
| 0.6 | 1.23294 | 2.00831 | 7.48594 | 17.65162 |
| 0.65 | 1.28514 | 2.17685 | 8.47627 | 20.16716 |
| 0.7 | 1.33896 | 2.35062 | 9.49734 | 22.76078 |
| 0.75 | 1.39388 | 2.52797 | 10.53945 | 25.40787 |
| 0.8 | 1.4494 | 2.70721 | 11.5927 | 28.08329 |
| 0.85 | 1.50497 | 2.88665 | 12.64709 | 30.76165 |
| 0.9 | 1.56007 | 3.06458 | 13.6926 | 33.41752 |
| 0.95 | 1.61419 | 3.2393 | 14.71932 | 36.02569 |
| 1 | 1.6668 | 3.40917 | 15.71749 | 38.5614 |
| 1.05 | 1.7174 | 3.57256 | 16.67764 | 41.00058 |
| 1.1 | 1.76552 | 3.72792 | 17.59064 | 43.32007 |
| 1.15 | 1.81069 | 3.87379 | 18.44783 | 45.49784 |
| 1.2 | 1.8525 | 4.00877 | 19.24108 | 47.51323 |
| 1.25 | 1.89053 | 4.13158 | 19.96283 | 49.34711 |
| 1.3 | 1.92444 | 4.24105 | 20.60625 | 50.98206 |
| 1.35 | 1.95389 | 4.33616 | 21.16523 | 52.40256 |
| 1.4 | 1.97861 | 4.41598 | 21.63445 | 53.59513 |
| 1.45 | 1.99836 | 4.47977 | 22.00946 | 54.54845 |
| 1.5 | 2.01296 | 4.52692 | 22.2867 | 55.25346 |
| 1.55 | 2.02226 | 4.55697 | 22.46354 | 55.70348 |
| 1.6 | 2.02618 | 4.56966 | 22.5383 | 55.89422 |
| 1.65 | 2.02468 | 4.56484 | 22.51027 | 55.82389 |
| 1.7 | 2.01778 | 4.54258 | 22.37971 | 55.49314 |
| 1.75 | 2.00553 | 4.50308 | 22.14786 | 54.90512 |
| 1.8 | 1.98807 | 4.44671 | 21.81693 | 54.06541 |
| 1.85 | 1.96554 | 4.37402 | 21.39006 | 52.98198 |
| 1.9 | 1.93818 | 4.28569 | 20.8713 | 51.66512 |
| 1.95 | 1.90623 | 4.18256 | 20.26557 | 50.12733 |
| 2 | 1.87 | 4.06562 | 19.57864 | 48.3832 |
| 2.05 | 1.82983 | 3.93596 | 18.81701 | 46.4493 |
| 2.1 | 1.78611 | 3.79483 | 17.98793 | 44.34398 |
| 2.15 | 1.73925 | 3.64356 | 17.09927 | 42.08724 |
| 2.2 | 1.6897 | 3.4836 | 16.15946 | 39.70048 |
| 2.25 | 1.63793 | 3.31645 | 15.17743 | 37.20638 |
| 2.3 | 1.58443 | 3.14371 | 14.1625 | 34.62861 |
| 2.35 | 1.5297 | 2.96702 | 13.12431 | 31.99164 |
| 2.4 | 1.47427 | 2.78805 | 12.07272 | 29.32051 |
| 2.45 | 1.41867 | 2.60851 | 11.01771 | 26.64056 |
| 2.5 | 1.36341 | 2.43009 | 9.9693 | 23.97725 |
| 2.55 | 1.30904 | 2.25451 | 8.93744 | 21.35586 |
| 2.6 | 1.25605 | 2.08341 | 7.93194 | 18.80127 |
| 2.65 | 1.20497 | 1.91843 | 6.96234 | 16.33774 |
| 2.7 | 1.15626 | 1.76114 | 6.03785 | 13.98864 |
| 2.75 | 1.1104 | 1.61302 | 5.16724 | 11.77629 |
| 2.8 | 1.06782 | 1.47549 | 4.35878 | 9.72168 |
| 2.85 | 1.02892 | 1.34984 | 3.62015 | 7.84432 |
| 2.9 | 0.99407 | 1.23728 | 2.95836 | 6.16203 |
| 2.95 | 0.96361 | 1.13887 | 2.37969 | 4.69078 |
| 3 | 0.93781 | 1.05554 | 1.88965 | 3.44454 |
| 3.05 | 0.91694 | 0.9881 | 1.49287 | 2.43514 |
| 3.1 | 0.90118 | 0.93717 | 1.19314 | 1.67216 |
| 3.15 | 0.89069 | 0.90324 | 0.99329 | 1.16284 |
| 3.2 | 0.88556 | 0.88663 | 0.89522 | 0.91202 |
| 3.25 | 0.88585 | 0.8875 | 0.89987 | 0.92208 |
| 3.3 | 0.89155 | 0.90585 | 1.00718 | 1.19292 |
| 3.35 | 0.9026 | 0.9415 | 1.21615 | 1.72198 |
| 3.4 | 0.91891 | 0.9941 | 1.52479 | 2.50423 |
| 3.45 | 0.94031 | 1.06317 | 1.93016 | 3.53224 |
| 3.5 | 0.96661 | 1.14804 | 2.42842 | 4.79626 |
| 3.55 | 0.99755 | 1.24791 | 3.01483 | 6.28429 |
| 3.6 | 1.03284 | 1.36182 | 3.68383 | 7.9822 |
| 3.65 | 1.07215 | 1.48871 | 4.42907 | 9.87386 |
| 3.7 | 1.11509 | 1.62736 | 5.24347 | 11.94134 |
| 3.75 | 1.16128 | 1.77646 | 6.1193 | 14.16498 |
| 3.8 | 1.21026 | 1.93459 | 7.04824 | 16.5237 |
| 3.85 | 1.26157 | 2.10025 | 8.02147 | 18.99509 |
| 3.9 | 1.31472 | 2.27187 | 9.02974 | 21.5557 |
| 3.95 | 1.36922 | 2.44782 | 10.0635 | 24.18121 |
| 4 | 1.42453 | 2.62642 | 11.11292 | 26.8467 |
| 4.05 | 1.48014 | 2.80598 | 12.16803 | 29.52687 |
| 4.1 | 1.53552 | 2.9848 | 13.21882 | 32.19627 |
| 4.15 | 1.59014 | 3.16117 | 14.25531 | 34.82957 |
| 4.2 | 1.64349 | 3.33342 | 15.26766 | 37.40175 |
| 4.25 | 1.69505 | 3.49992 | 16.24625 | 39.88842 |
| 4.3 | 1.74434 | 3.65909 | 17.1818 | 42.26595 |
| 4.35 | 1.79088 | 3.80941 | 18.06542 | 44.51178 |
| 4.4 | 1.83425 | 3.94945 | 18.88872 | 46.60458 |
| 4.45 | 1.87401 | 4.07789 | 19.64388 | 48.5245 |
| 4.5 | 1.90981 | 4.1935 | 20.32373 | 50.2533 |
| 4.55 | 1.94129 | 4.2952 | 20.92182 | 51.77457 |
| 4.6 | 1.96815 | 4.382 | 21.43247 | 53.07387 |
| 4.65 | 1.99015 | 4.45309 | 21.85083 | 54.13886 |
| 4.7 | 2.00708 | 4.50779 | 22.17292 | 54.95944 |
| 4.75 | 2.01876 | 4.54558 | 22.39569 | 55.52781 |
| 4.8 | 2.02509 | 4.56611 | 22.51702 | 55.83859 |
| 4.85 | 2.02602 | 4.56917 | 22.53577 | 55.8888 |
| 4.9 | 2.02153 | 4.55475 | 22.45174 | 55.67799 |
| 4.95 | 2.01166 | 4.52297 | 22.26575 | 55.20816 |
| 5 | 1.99651 | 4.47414 | 21.97955 | 54.48375 |
| 5.05 | 1.97623 | 4.40872 | 21.59586 | 53.51166 |
| 5.1 | 1.951 | 4.32733 | 21.11834 | 52.3011 |
| 5.15 | 1.92106 | 4.23075 | 20.5515 | 50.86358 |
| 5.2 | 1.88671 | 4.11989 | 19.90074 | 49.21273 |
| 5.25 | 1.84826 | 3.99581 | 19.17223 | 47.36423 |
| 5.3 | 1.80608 | 3.85969 | 18.37289 | 45.33563 |
| 5.35 | 1.76057 | 3.71281 | 17.51031 | 43.14619 |
| 5.4 | 1.71217 | 3.55657 | 16.59268 | 40.81668 |
| 5.45 | 1.66133 | 3.39246 | 15.62872 | 38.36924 |
| 5.5 | 1.60854 | 3.22204 | 14.62758 | 35.82708 |
| 5.55 | 1.55429 | 3.04692 | 13.59876 | 33.21435 |
| 5.6 | 1.49911 | 2.86877 | 12.55203 | 30.55584 |
| 5.65 | 1.44352 | 2.68927 | 11.49733 | 27.8768 |
| 5.7 | 1.38805 | 2.51014 | 10.44468 | 25.20266 |
| 5.75 | 1.33321 | 2.33307 | 9.40407 | 22.5588 |
| 5.8 | 1.27954 | 2.15975 | 8.38538 | 19.97033 |
| 5.85 | 1.22755 | 1.99183 | 7.39829 | 17.46181 |
| 5.9 | 1.17772 | 1.83088 | 6.45216 | 15.05706 |
| 5.95 | 1.13053 | 1.67846 | 5.55599 | 12.77891 |
| 6 | 1.08643 | 1.53599 | 4.71827 | 10.64899 |
| 6.05 | 1.04583 | 1.40484 | 3.94697 | 8.68751 |
| 6.1 | 1.00913 | 1.28625 | 3.24941 | 6.91309 |
| 6.15 | 0.97667 | 1.18135 | 2.63221 | 5.34259 |
| 6.2 | 0.94875 | 1.09112 | 2.10123 | 3.99091 |
| 6.25 | 0.92565 | 1.01644 | 1.66151 | 2.87088 |
| 6.3 | 0.90759 | 0.95801 | 1.31723 | 1.99313 |
| 6.35 | 0.89473 | 0.91638 | 1.07166 | 1.366 |
| 6.4 | 0.88719 | 0.89195 | 0.92712 | 0.99544 |
| 6.45 | 0.88505 | 0.88494 | 0.885 | 0.88497 |
| 6.5 | 0.88833 | 0.89544 | 0.94569 | 1.03563 |
| 6.55 | 0.897 | 0.92333 | 1.10861 | 1.446 |
| 6.6 | 0.91098 | 0.96836 | 1.37222 | 2.11219 |
| 6.65 | 0.93012 | 1.03008 | 1.73402 | 3.02785 |
| 6.7 | 0.95426 | 1.10793 | 2.19056 | 4.18432 |
| 6.75 | 0.98315 | 1.20116 | 2.73752 | 5.5706 |
| 6.8 | 1.01654 | 1.30888 | 3.36971 | 7.17353 |
| 6.85 | 1.05409 | 1.43008 | 4.08111 | 8.9779 |
| 6.9 | 1.09546 | 1.56359 | 4.86497 | 10.96658 |
| 6.95 | 1.14025 | 1.70816 | 5.71386 | 13.1207 |
| 7 | 1.18804 | 1.8624 | 6.61971 | 15.41979 |
| 7.05 | 1.23837 | 2.02487 | 7.57391 | 17.84204 |
| 7.1 | 1.29076 | 2.194 | 8.56742 | 20.36445 |
| 7.15 | 1.34472 | 2.3682 | 9.59079 | 22.96307 |
| 7.2 | 1.39973 | 2.54582 | 10.63431 | 25.61324 |
| 7.25 | 1.45527 | 2.72516 | 11.68808 | 28.28979 |
| 7.3 | 1.51082 | 2.90452 | 12.74207 | 30.96732 |
| 7.35 | 1.56584 | 3.0822 | 13.7863 | 33.62041 |
| 7.4 | 1.61982 | 3.25651 | 14.81083 | 36.22388 |
| 7.45 | 1.67224 | 3.4258 | 15.80595 | 38.753 |
| 7.5 | 1.72261 | 3.58846 | 16.76221 | 41.18376 |
| 7.55 | 1.77044 | 3.74294 | 17.67052 | 43.4931 |
| 7.6 | 1.81528 | 3.88778 | 18.52226 | 45.65909 |
| 7.65 | 1.8567 | 4.0216 | 19.30934 | 47.66116 |
| 7.7 | 1.89431 | 4.14313 | 20.02429 | 49.48031 |
| 7.75 | 1.92776 | 4.25122 | 20.66032 | 51.09927 |
| 7.8 | 1.95673 | 4.34483 | 21.21139 | 52.50267 |
| 7.85 | 1.98093 | 4.42308 | 21.67227 | 53.67719 |
| 7.9 | 2.00015 | 4.48524 | 22.03858 | 54.61168 |
| 7.95 | 2.01419 | 4.53069 | 22.30684 | 55.29726 |
| 8 | 2.02293 | 4.55902 | 22.47451 | 55.72742 |
| 8.05 | 2.02628 | 4.56996 | 22.53999 | 55.89809 |
| 8.1 | 2.02421 | 4.5634 | 22.50267 | 55.80765 |
| 8.15 | 2.01673 | 4.5394 | 22.36289 | 55.45694 |
| 8.2 | 2.00393 | 4.4982 | 22.12199 | 54.84931 |
| 8.25 | 1.98592 | 4.44018 | 21.78225 | 53.99051 |
| 8.3 | 1.96288 | 4.36589 | 21.34689 | 52.88871 |
| 8.35 | 1.93501 | 4.27604 | 20.82006 | 51.55436 |
| 8.4 | 1.9026 | 4.17149 | 20.20674 | 50.00013 |
| 8.45 | 1.86593 | 4.05322 | 19.51278 | 48.24078 |
| 8.5 | 1.82537 | 3.92236 | 18.74476 | 46.293 |
| 8.55 | 1.7813 | 3.78015 | 17.90996 | 44.17529 |
| 8.6 | 1.73414 | 3.62795 | 17.01632 | 41.90775 |
| 8.65 | 1.68433 | 3.4672 | 16.07232 | 39.51191 |
| 8.7 | 1.63235 | 3.29942 | 15.08693 | 37.01051 |
| 8.75 | 1.5787 | 3.12621 | 14.0695 | 34.4273 |
| 8.8 | 1.52387 | 2.94921 | 13.02969 | 31.78681 |
| 8.85 | 1.4684 | 2.77011 | 11.97738 | 29.11409 |
| 8.9 | 1.41281 | 2.59061 | 10.92255 | 26.43452 |
| 8.95 | 1.35762 | 2.4124 | 9.87523 | 23.77355 |
| 9 | 1.30337 | 2.23719 | 8.84536 | 21.15642 |
| 9.05 | 1.25056 | 2.06663 | 7.84271 | 18.608 |
| 9.1 | 1.1997 | 1.90235 | 6.87682 | 16.15246 |
| 9.15 | 1.15128 | 1.74591 | 5.95684 | 13.81312 |
| 9.2 | 1.10574 | 1.59879 | 5.09151 | 11.61218 |
| 9.25 | 1.06353 | 1.46238 | 4.28906 | 9.57055 |
| 9.3 | 1.02504 | 1.33799 | 3.55709 | 7.70761 |
| 9.35 | 0.99064 | 1.2268 | 2.90257 | 6.04103 |
| 9.4 | 0.96066 | 1.12985 | 2.3317 | 4.58664 |
| 9.45 | 0.93537 | 1.04808 | 1.8499 | 3.35824 |
| 9.5 | 0.91503 | 0.98226 | 1.46175 | 2.36751 |
| 9.55 | 0.89983 | 0.93301 | 1.17094 | 1.62384 |
| 9.6 | 0.8899 | 0.90079 | 0.98022 | 1.13429 |
| 9.65 | 0.88534 | 0.88593 | 0.89141 | 0.90351 |
| 9.7 | 0.8862 | 0.88855 | 0.90535 | 0.93369 |
| 9.75 | 0.89246 | 0.90864 | 1.02191 | 1.22454 |
| 9.8 | 0.90408 | 0.946 | 1.23998 | 1.77331 |
| 9.85 | 0.92093 | 1.00027 | 1.55749 | 2.57478 |
| 9.9 | 0.94286 | 1.07095 | 1.97143 | 3.62135 |
| 9.95 | 0.96966 | 1.15735 | 2.47786 | 4.90307 |
| 10 | 1.00107 | 1.25867 | 3.07198 | 6.40779 |
| 10.05 | 1.03681 | 1.37394 | 3.74814 | 8.12122 |
| 10.1 | 1.07652 | 1.50205 | 4.49993 | 10.02709 |
| 10.15 | 1.11983 | 1.64181 | 5.32021 | 12.10731 |
| 10.2 | 1.16633 | 1.79188 | 6.20118 | 14.34213 |
| 10.25 | 1.21558 | 1.95083 | 7.13449 | 16.71033 |
| 10.3 | 1.2671 | 2.11716 | 8.11127 | 19.18944 |
| 10.35 | 1.32042 | 2.28928 | 9.12225 | 21.75592 |
| 10.4 | 1.37503 | 2.46557 | 10.15783 | 24.3854 |
| 10.45 | 1.4304 | 2.64434 | 11.20817 | 27.05292 |
| 10.5 | 1.48601 | 2.8239 | 12.26331 | 29.73317 |
| 10.55 | 1.54134 | 3.00255 | 13.31321 | 32.40069 |
| 10.6 | 1.59585 | 3.17859 | 14.34792 | 35.03016 |
| 10.65 | 1.64903 | 3.35034 | 15.35761 | 37.59662 |
| 10.7 | 1.70037 | 3.51618 | 16.33269 | 40.0757 |
| 10.75 | 1.74939 | 3.67453 | 17.26391 | 42.44388 |
| 10.8 | 1.79562 | 3.82388 | 18.14241 | 44.67866 |
| 10.85 | 1.83862 | 3.96282 | 18.95987 | 46.75884 |
| 10.9 | 1.87799 | 4.09003 | 19.70851 | 48.66466 |
| 10.95 | 1.91334 | 4.2043 | 20.38123 | 50.37804 |
| 11 | 1.94435 | 4.30455 | 20.97164 | 51.8827 |
| 11.05 | 1.97071 | 4.38982 | 21.47413 | 53.16436 |
| 11.1 | 1.99218 | 4.4593 | 21.88394 | 54.21086 |
| 11.15 | 2.00856 | 4.51233 | 22.19717 | 55.01227 |
| 11.2 | 2.01968 | 4.54841 | 22.41085 | 55.56096 |
| 11.25 | 2.02545 | 4.5672 | 22.52294 | 55.85174 |
| 11.3 | 2.0258 | 4.56852 | 22.53239 | 55.88184 |
| 11.35 | 2.02074 | 4.55235 | 22.43911 | 55.65097 |
| 11.4 | 2.01031 | 4.51885 | 22.24397 | 55.16134 |
| 11.45 | 1.99461 | 4.46834 | 21.94884 | 54.41758 |
| 11.5 | 1.97379 | 4.40129 | 21.55651 | 53.42677 |
| 11.55 | 1.94805 | 4.31835 | 21.07071 | 52.19829 |
| 11.6 | 1.91764 | 4.2203 | 20.49606 | 50.74383 |
| 11.65 | 1.88283 | 4.10807 | 19.83801 | 49.07717 |
| 11.7 | 1.84397 | 3.98273 | 19.1028 | 47.21416 |
| 11.75 | 1.80142 | 3.84547 | 18.29743 | 45.17246 |
| 11.8 | 1.75559 | 3.6976 | 17.42953 | 42.97148 |
| 11.85 | 1.7069 | 3.54051 | 16.50735 | 40.63209 |
| 11.9 | 1.65584 | 3.3757 | 15.53965 | 38.17651 |
| 11.95 | 1.60287 | 3.20473 | 14.5356 | 35.62805 |
| 12 | 1.5485 | 3.02923 | 13.50476 | 33.0109 |
| 12.05 | 1.49325 | 2.85087 | 12.4569 | 30.34992 |
| 12.1 | 1.43765 | 2.67133 | 11.40198 | 27.67035 |
| 12.15 | 1.38222 | 2.49233 | 10.35 | 24.99764 |
| 12.2 | 1.32748 | 2.31557 | 9.31097 | 22.35716 |
| 12.25 | 1.27396 | 2.14271 | 8.29475 | 19.77398 |
| 12.3 | 1.22217 | 1.97541 | 7.31097 | 17.27261 |
| 12.35 | 1.1726 | 1.81525 | 6.369 | 14.87682 |
| 12.4 | 1.12572 | 1.66375 | 5.47777 | 12.60933 |
| 12.45 | 1.08197 | 1.52236 | 4.64573 | 10.49168 |
| 12.5 | 1.04177 | 1.39241 | 3.8808 | 8.54397 |
| 12.55 | 1.0055 | 1.27514 | 3.19024 | 6.78468 |
| 12.6 | 0.9735 | 1.17166 | 2.5806 | 5.23053 |
| 12.65 | 0.94608 | 1.08295 | 2.05767 | 3.89626 |
| 12.7 | 0.92351 | 1.00987 | 1.62642 | 2.79454 |
| 12.75 | 0.90598 | 0.95309 | 1.29094 | 1.93583 |
| 12.8 | 0.89368 | 0.91316 | 1.05442 | 1.32828 |
| 12.85 | 0.88671 | 0.89046 | 0.91909 | 0.97766 |
| 12.9 | 0.88514 | 0.88521 | 0.88626 | 0.88729 |
| 12.95 | 0.889 | 0.89745 | 0.95622 | 1.05804 |
| 13 | 0.89823 | 0.92706 | 1.12832 | 1.48829 |
| 13.05 | 0.91276 | 0.97378 | 1.40092 | 2.17394 |
| 13.1 | 0.93244 | 1.03715 | 1.77144 | 3.10849 |
| 13.15 | 0.95709 | 1.11657 | 2.23634 | 4.28307 |
| 13.2 | 0.98647 | 1.21128 | 2.79123 | 5.68653 |
| 13.25 | 1.02031 | 1.3204 | 3.43083 | 7.30555 |
| 13.3 | 1.05829 | 1.44288 | 4.14907 | 9.12475 |
| 13.35 | 1.10004 | 1.57755 | 4.93912 | 11.12686 |
| 13.4 | 1.14517 | 1.72315 | 5.79349 | 13.29289 |
| 13.45 | 1.19324 | 1.87829 | 6.70407 | 15.60226 |
| 13.5 | 1.24381 | 2.04149 | 7.66221 | 18.03305 |
| 13.55 | 1.29639 | 2.2112 | 8.65881 | 20.56219 |
| 13.6 | 1.35048 | 2.38582 | 9.6844 | 23.16567 |
| 13.65 | 1.40558 | 2.56368 | 10.72926 | 25.81877 |
| 13.7 | 1.46115 | 2.7431 | 11.78346 | 28.4963 |
| 13.75 | 1.51666 | 2.92237 | 12.83698 | 31.17285 |
| 13.8 | 1.5716 | 3.09979 | 13.87983 | 33.82301 |
| 13.85 | 1.62544 | 3.27368 | 14.90211 | 36.42162 |
| 13.9 | 1.67767 | 3.44238 | 15.8941 | 38.94401 |
| 13.95 | 1.72779 | 3.60429 | 16.84639 | 41.36623 |
| 14 | 1.77532 | 3.75787 | 17.74993 | 43.66529 |
| 14.05 | 1.81982 | 3.90167 | 18.59615 | 45.81936 |
| 14.1 | 1.86086 | 4.03431 | 19.37701 | 47.808 |
| 14.15 | 1.89805 | 4.15455 | 20.0851 | 49.61233 |
| 14.2 | 1.93104 | 4.26123 | 20.71369 | 51.2152 |
| 14.25 | 1.95951 | 4.35335 | 21.25681 | 52.60142 |
| 14.3 | 1.9832 | 4.43003 | 21.70931 | 53.75782 |
| 14.35 | 2.00188 | 4.49054 | 22.06689 | 54.67342 |
| 14.4 | 2.01537 | 4.5343 | 22.32616 | 55.33953 |
| 14.45 | 2.02354 | 4.5609 | 22.48464 | 55.74983 |
| 14.5 | 2.02631 | 4.57009 | 22.54085 | 55.90041 |
| 14.55 | 2.02367 | 4.56179 | 22.49424 | 55.78986 |
| 14.6 | 2.01563 | 4.53606 | 22.34525 | 55.41922 |
| 14.65 | 2.00227 | 4.49315 | 22.09531 | 54.792 |
| 14.7 | 1.98372 | 4.43348 | 21.74678 | 53.91417 |
| 14.75 | 1.96016 | 4.3576 | 21.30298 | 52.79406 |
| 14.8 | 1.9318 | 4.26624 | 20.76811 | 51.4423 |
| 14.85 | 1.89892 | 4.16027 | 20.14726 | 49.87172 |
| 14.9 | 1.86183 | 4.04069 | 19.44632 | 48.09724 |
| 14.95 | 1.82088 | 3.90864 | 18.67195 | 46.13569 |
| 15 | 1.77646 | 3.76538 | 17.8315 | 44.00571 |
| 15.05 | 1.729 | 3.61225 | 16.93296 | 41.72751 |
| 15.1 | 1.67894 | 3.45073 | 15.98484 | 39.32271 |
| 15.15 | 1.62676 | 3.28233 | 14.99617 | 36.81416 |
| 15.2 | 1.57295 | 3.10867 | 13.97631 | 34.22566 |
| 15.25 | 1.51803 | 2.93139 | 12.93497 | 31.58178 |
| 15.3 | 1.46252 | 2.75217 | 11.88201 | 28.90763 |
| 15.35 | 1.40695 | 2.57272 | 10.82746 | 26.2286 |
| 15.4 | 1.35184 | 2.39474 | 9.78131 | 23.5701 |
| 15.45 | 1.29771 | 2.21991 | 8.7535 | 20.95739 |
| 15.5 | 1.24509 | 2.04991 | 7.75378 | 18.41527 |
| 15.55 | 1.19447 | 1.88635 | 6.79167 | 15.96786 |
| 15.6 | 1.14632 | 1.73077 | 5.87627 | 13.63841 |
| 15.65 | 1.10112 | 1.58466 | 5.0163 | 11.44902 |
| 15.7 | 1.05928 | 1.4494 | 4.21991 | 9.42049 |
| 15.75 | 1.02121 | 1.32627 | 3.49467 | 7.57206 |
| 15.8 | 0.98726 | 1.21646 | 2.84746 | 5.92128 |
| 15.85 | 0.95776 | 1.12099 | 2.28442 | 4.48383 |
| 15.9 | 0.93299 | 1.04078 | 1.81091 | 3.27336 |
| 15.95 | 0.91318 | 0.97658 | 1.43143 | 2.30135 |
| 16 | 0.89853 | 0.92902 | 1.14956 | 1.57703 |
| 16.05 | 0.88916 | 0.89853 | 0.96799 | 1.10727 |
| 16.1 | 0.88517 | 0.8854 | 0.88844 | 0.89654 |
| 16.15 | 0.8866 | 0.88978 | 0.91168 | 0.94684 |
| 16.2 | 0.89344 | 0.9116 | 1.03747 | 1.25769 |
| 16.25 | 0.90561 | 0.95066 | 1.26462 | 1.82614 |
| 16.3 | 0.92301 | 1.0066 | 1.59098 | 2.64679 |
| 16.35 | 0.94546 | 1.07888 | 2.01345 | 3.71185 |
| 16.4 | 0.97276 | 1.16682 | 2.52802 | 5.01121 |
| 16.45 | 1.00465 | 1.26957 | 3.12979 | 6.53254 |
| 16.5 | 1.04082 | 1.38617 | 3.81307 | 8.26139 |
| 16.55 | 1.08093 | 1.51551 | 4.57135 | 10.18135 |
| 16.6 | 1.12459 | 1.65636 | 5.39744 | 12.27419 |
| 16.65 | 1.17141 | 1.80738 | 6.2835 | 14.52006 |
| 16.7 | 1.22092 | 1.96714 | 7.2211 | 16.89762 |
| 16.75 | 1.27266 | 2.13412 | 8.20136 | 19.3843 |
| 16.8 | 1.32614 | 2.30673 | 9.21496 | 21.95651 |
| 16.85 | 1.38085 | 2.48334 | 10.25228 | 24.58982 |
| 16.9 | 1.43627 | 2.66227 | 11.30347 | 27.25922 |
| 16.95 | 1.49188 | 2.84182 | 12.35854 | 29.93939 |
| 17 | 1.54714 | 3.02028 | 13.40748 | 32.60488 |
| 17.05 | 1.60153 | 3.19596 | 14.44033 | 35.23038 |
| 17.1 | 1.65454 | 3.3672 | 15.44728 | 37.79096 |
| 17.15 | 1.70567 | 3.53236 | 16.41878 | 40.26233 |
| 17.2 | 1.75441 | 3.68987 | 17.34558 | 42.62102 |
| 17.25 | 1.80032 | 3.83825 | 18.21891 | 44.84463 |
| 17.3 | 1.84296 | 3.97608 | 19.03045 | 46.91206 |
| 17.35 | 1.88192 | 4.10205 | 19.77252 | 48.80368 |
| 17.4 | 1.91683 | 4.21497 | 20.43805 | 50.50154 |
| 17.45 | 1.94736 | 4.31376 | 21.02073 | 51.98951 |
| 17.5 | 1.97321 | 4.39748 | 21.51504 | 53.25346 |
| 17.55 | 1.99416 | 4.46535 | 21.91627 | 54.28141 |
| 17.6 | 2.00998 | 4.51671 | 22.22061 | 55.06359 |
| 17.65 | 2.02055 | 4.55107 | 22.42517 | 55.59257 |
| 17.7 | 2.02574 | 4.56812 | 22.52802 | 55.86334 |
| 17.75 | 2.02552 | 4.56769 | 22.52818 | 55.87332 |
| 17.8 | 2.01989 | 4.54978 | 22.42564 | 55.62242 |
| 17.85 | 2.0089 | 4.51456 | 22.22138 | 55.11301 |
| 17.9 | 1.99265 | 4.46238 | 21.91733 | 54.34995 |
| 17.95 | 1.9713 | 4.39371 | 21.51639 | 53.34046 |
| 18 | 1.94506 | 4.30922 | 21.02236 | 52.09414 |
| 18.05 | 1.91416 | 4.20971 | 20.43994 | 50.62282 |
| 18.1 | 1.87891 | 4.09612 | 19.77464 | 48.94045 |
| 18.15 | 1.83964 | 3.96954 | 19.0328 | 47.06302 |
| 18.2 | 1.79673 | 3.83116 | 18.22145 | 45.00835 |
| 18.25 | 1.75057 | 3.6823 | 17.3483 | 42.79595 |
| 18.3 | 1.70161 | 3.52437 | 16.42164 | 40.4468 |
| 18.35 | 1.65032 | 3.35887 | 15.45027 | 37.98323 |
| 18.4 | 1.59718 | 3.18738 | 14.44341 | 35.42861 |
| 18.45 | 1.5427 | 3.01152 | 13.41063 | 32.8072 |
| 18.5 | 1.48739 | 2.83296 | 12.36172 | 30.14388 |
| 18.55 | 1.43178 | 2.6534 | 11.30665 | 27.46393 |
| 18.6 | 1.37639 | 2.47455 | 10.25544 | 24.79281 |
| 18.65 | 1.32176 | 2.2981 | 9.21806 | 22.15585 |
| 18.7 | 1.2684 | 2.12572 | 8.20438 | 19.57811 |
| 18.75 | 1.21683 | 1.95906 | 7.224 | 17.08405 |
| 18.8 | 1.16752 | 1.7997 | 6.28625 | 14.69734 |
| 18.85 | 1.12094 | 1.64915 | 5.40003 | 12.44064 |
| 18.9 | 1.07755 | 1.50884 | 4.57374 | 10.33539 |
| 18.95 | 1.03774 | 1.38011 | 3.81525 | 8.40155 |
| 19 | 1.00191 | 1.26416 | 3.13174 | 6.6575 |
| 19.05 | 0.97038 | 1.16212 | 2.52971 | 5.11978 |
| 19.1 | 0.94347 | 1.07494 | 2.01487 | 3.803 |
| 19.15 | 0.92141 | 1.00345 | 1.59212 | 2.71965 |
| 19.2 | 0.90443 | 0.94833 | 1.26546 | 1.88002 |
| 19.25 | 0.89269 | 0.91011 | 1.038 | 1.29209 |
| 19.3 | 0.88629 | 0.88915 | 0.9119 | 0.96142 |
| 19.35 | 0.88529 | 0.88564 | 0.88836 | 0.89117 |
| 19.4 | 0.88972 | 0.89963 | 0.9676 | 1.08199 |
| 19.45 | 0.89952 | 0.93097 | 1.14886 | 1.53208 |
| 19.5 | 0.91459 | 0.97937 | 1.43043 | 2.23717 |
| 19.55 | 0.93481 | 1.04437 | 1.80963 | 3.19055 |
| 19.6 | 0.95997 | 1.12535 | 2.28286 | 4.38319 |
| 19.65 | 0.98984 | 1.22155 | 2.84563 | 5.80375 |
| 19.7 | 1.02414 | 1.33205 | 3.49259 | 7.43875 |
| 19.75 | 1.06253 | 1.4558 | 4.21761 | 9.27268 |
| 19.8 | 1.10465 | 1.59163 | 5.0138 | 11.28811 |
| 19.85 | 1.15011 | 1.73824 | 5.87359 | 13.46593 |
| 19.9 | 1.19847 | 1.89425 | 6.78883 | 15.78544 |
| 19.95 | 1.24928 | 2.05817 | 7.75082 | 18.22464 |
| 20 | 1.30204 | 2.22845 | 8.75043 | 20.76037 |
