# Supplementary material for: Analysis of sinusoidal post-buckling deformation of horizontal coiled tubing with initial residual bending
Source: PLoS One. 2024 May 14;19(5):e0301610. doi: 10.1371/journal.pone.0301610 (PMC11093391; doi:10.1371/journal.pone.0301610)
Supplement: S1 File — (ZIP) [file pone.0301610.s001.zip › The values used to build graphs - Fig 10.docx]

## The values used to build graphs

The minimal data set of the original data for plotting curves in Fig 10 is as follows:

| x-axis | ‾*L*_0_ = 100 | ‾*L*_0_ = 200 | ‾*L*_0_ = 300 | ‾*L*_0_ = 400 |
| --- | --- | --- | --- | --- |
| 0 | 0.81788 | 0.78758 | 0.83074 | 0.86259 |
| 0.05 | 0.89936 | 0.81312 | 0.84179 | 0.86794 |
| 0.1 | 1.14315 | 0.8895 | 0.87484 | 0.88395 |
| 0.15 | 1.54738 | 1.01605 | 0.92958 | 0.91047 |
| 0.2 | 2.10888 | 1.19167 | 1.00551 | 0.94724 |
| 0.25 | 2.82332 | 1.4148 | 1.10194 | 0.99393 |
| 0.3 | 3.68514 | 1.68349 | 1.21797 | 1.05009 |
| 0.35 | 4.68765 | 1.99536 | 1.35256 | 1.11521 |
| 0.4 | 5.82306 | 2.34767 | 1.50445 | 1.18866 |
| 0.45 | 7.08256 | 2.73732 | 1.67225 | 1.26976 |
| 0.5 | 8.45637 | 3.16089 | 1.85443 | 1.35775 |
| 0.55 | 9.93383 | 3.61465 | 2.04932 | 1.4518 |
| 0.6 | 11.50345 | 4.09459 | 2.25511 | 1.55104 |
| 0.65 | 13.15304 | 4.5965 | 2.46993 | 1.65453 |
| 0.7 | 14.8698 | 5.11596 | 2.69181 | 1.76131 |
| 0.75 | 16.6404 | 5.6484 | 2.91869 | 1.87036 |
| 0.8 | 18.45108 | 6.18913 | 3.14851 | 1.98068 |
| 0.85 | 20.28777 | 6.73339 | 3.37915 | 2.09121 |
| 0.9 | 22.13621 | 7.27639 | 3.6085 | 2.20094 |
| 0.95 | 23.98204 | 7.81335 | 3.83444 | 2.30882 |
| 1 | 25.81092 | 8.33954 | 4.05491 | 2.41385 |
| 1.05 | 27.60864 | 8.85033 | 4.26787 | 2.51504 |
| 1.1 | 29.36124 | 9.34123 | 4.47138 | 2.61144 |
| 1.15 | 31.05509 | 9.8079 | 4.66356 | 2.70215 |
| 1.2 | 32.67704 | 10.24625 | 4.84265 | 2.78632 |
| 1.25 | 34.21448 | 10.6524 | 5.007 | 2.86315 |
| 1.3 | 35.65547 | 11.0228 | 5.15511 | 2.93193 |
| 1.35 | 36.9888 | 11.35416 | 5.28561 | 2.99202 |
| 1.4 | 38.20412 | 11.64358 | 5.39731 | 3.04284 |
| 1.45 | 39.29198 | 11.88851 | 5.48918 | 3.08392 |
| 1.5 | 40.24393 | 12.08679 | 5.56038 | 3.11488 |
| 1.55 | 41.05256 | 12.23668 | 5.61025 | 3.13542 |
| 1.6 | 41.7116 | 12.33684 | 5.63833 | 3.14536 |
| 1.65 | 42.21591 | 12.38641 | 5.64436 | 3.1446 |
| 1.7 | 42.56159 | 12.38495 | 5.6283 | 3.13314 |
| 1.75 | 42.74594 | 12.33246 | 5.59029 | 3.11109 |
| 1.8 | 42.76752 | 12.22941 | 5.53067 | 3.07867 |
| 1.85 | 42.62618 | 12.07671 | 5.45 | 3.03617 |
| 1.9 | 42.32302 | 11.87571 | 5.34901 | 2.984 |
| 1.95 | 41.86038 | 11.62817 | 5.22864 | 2.92264 |
| 2 | 41.24186 | 11.33627 | 5.08999 | 2.85266 |
| 2.05 | 40.47227 | 11.00258 | 4.93432 | 2.77474 |
| 2.1 | 39.5576 | 10.63004 | 4.76309 | 2.68959 |
| 2.15 | 38.50494 | 10.22194 | 4.57784 | 2.59801 |
| 2.2 | 37.32249 | 9.78187 | 4.38029 | 2.50087 |
| 2.25 | 36.01943 | 9.31369 | 4.17226 | 2.39907 |
| 2.3 | 34.60589 | 8.82154 | 3.95564 | 2.29357 |
| 2.35 | 33.09286 | 8.30974 | 3.73244 | 2.18537 |
| 2.4 | 31.49208 | 7.7828 | 3.50469 | 2.07546 |
| 2.45 | 29.81601 | 7.24537 | 3.27449 | 1.96489 |
| 2.5 | 28.07766 | 6.70217 | 3.04396 | 1.8547 |
| 2.55 | 26.29054 | 6.15798 | 2.81522 | 1.74591 |
| 2.6 | 24.46853 | 5.6176 | 2.59035 | 1.63954 |
| 2.65 | 22.6258 | 5.08578 | 2.37143 | 1.5366 |
| 2.7 | 20.77665 | 4.5672 | 2.16047 | 1.43804 |
| 2.75 | 18.93545 | 4.06643 | 1.95939 | 1.3448 |
| 2.8 | 17.11651 | 3.58787 | 1.77006 | 1.25774 |
| 2.85 | 15.33395 | 3.13575 | 1.59421 | 1.17769 |
| 2.9 | 13.60161 | 2.71403 | 1.43344 | 1.10539 |
| 2.95 | 11.93296 | 2.32642 | 1.28924 | 1.04152 |
| 3 | 10.34095 | 1.97635 | 1.16294 | 0.98668 |
| 3.05 | 8.83795 | 1.66688 | 1.05568 | 0.94138 |
| 3.1 | 7.43562 | 1.40075 | 0.96846 | 0.90605 |
| 3.15 | 6.14486 | 1.1803 | 0.90207 | 0.88102 |
| 3.2 | 4.97569 | 1.00746 | 0.85712 | 0.86652 |
| 3.25 | 3.93719 | 0.88375 | 0.83404 | 0.8627 |
| 3.3 | 3.03743 | 0.81027 | 0.83302 | 0.86958 |
| 3.35 | 2.28338 | 0.78767 | 0.85408 | 0.8871 |
| 3.4 | 1.6809 | 0.81613 | 0.89702 | 0.9151 |
| 3.45 | 1.23468 | 0.89541 | 0.96146 | 0.95331 |
| 3.5 | 0.94817 | 1.02481 | 1.04679 | 1.00138 |
| 3.55 | 0.8236 | 1.2032 | 1.15224 | 1.05885 |
| 3.6 | 0.86194 | 1.429 | 1.27684 | 1.12519 |
| 3.65 | 1.06289 | 1.70023 | 1.41944 | 1.19978 |
| 3.7 | 1.42488 | 2.0145 | 1.57873 | 1.28191 |
| 3.75 | 1.94512 | 2.36904 | 1.75327 | 1.37082 |
| 3.8 | 2.61956 | 2.76073 | 1.94143 | 1.46567 |
| 3.85 | 3.44296 | 3.18614 | 2.14149 | 1.56557 |
| 3.9 | 4.40892 | 3.6415 | 2.35162 | 1.66959 |
| 3.95 | 5.50996 | 4.12282 | 2.56989 | 1.77676 |
| 4 | 6.73752 | 4.62586 | 2.7943 | 1.88605 |
| 4.05 | 8.08207 | 5.14619 | 3.02278 | 1.99646 |
| 4.1 | 9.53316 | 5.67924 | 3.25323 | 2.10694 |
| 4.15 | 11.07954 | 6.2203 | 3.48355 | 2.21647 |
| 4.2 | 12.70918 | 6.76461 | 3.7116 | 2.324 |
| 4.25 | 14.40944 | 7.3074 | 3.93532 | 2.42855 |
| 4.3 | 16.1671 | 7.84387 | 4.15262 | 2.52911 |
| 4.35 | 17.96852 | 8.3693 | 4.36153 | 2.62475 |
| 4.4 | 19.79971 | 8.87907 | 4.56012 | 2.71458 |
| 4.45 | 21.64644 | 9.36869 | 4.74657 | 2.79775 |
| 4.5 | 23.49436 | 9.83385 | 4.91917 | 2.87348 |
| 4.55 | 25.32911 | 10.27045 | 5.07633 | 2.94106 |
| 4.6 | 27.13646 | 10.67465 | 5.21661 | 2.99985 |
| 4.65 | 28.90234 | 11.04289 | 5.33871 | 3.04931 |
| 4.7 | 30.61305 | 11.37192 | 5.44153 | 3.08897 |
| 4.75 | 32.25529 | 11.65886 | 5.52411 | 3.11845 |
| 4.8 | 33.8163 | 11.90116 | 5.58569 | 3.13749 |
| 4.85 | 35.28395 | 12.09671 | 5.62571 | 3.14591 |
| 4.9 | 36.64684 | 12.24377 | 5.64381 | 3.14361 |
| 4.95 | 37.89436 | 12.34106 | 5.63981 | 3.13064 |
| 5 | 39.01684 | 12.38771 | 5.61375 | 3.1071 |
| 5.05 | 40.00554 | 12.38331 | 5.56587 | 3.07321 |
| 5.1 | 40.85277 | 12.32791 | 5.49662 | 3.02931 |
| 5.15 | 41.55197 | 12.22199 | 5.40662 | 2.97579 |
| 5.2 | 42.09768 | 12.06648 | 5.29671 | 2.91316 |
| 5.25 | 42.48567 | 11.86275 | 5.16789 | 2.84201 |
| 5.3 | 42.71293 | 11.6126 | 5.02135 | 2.76301 |
| 5.35 | 42.77768 | 11.31823 | 4.85843 | 2.67689 |
| 5.4 | 42.67943 | 10.98223 | 4.68064 | 2.58447 |
| 5.45 | 42.41893 | 10.60757 | 4.48959 | 2.4866 |
| 5.5 | 41.99822 | 10.19753 | 4.28705 | 2.38422 |
| 5.55 | 41.42055 | 9.75574 | 4.07488 | 2.27827 |
| 5.6 | 40.69043 | 9.28607 | 3.85502 | 2.16976 |
| 5.65 | 39.81353 | 8.79267 | 3.62949 | 2.0597 |
| 5.7 | 38.79666 | 8.27988 | 3.40038 | 1.94912 |
| 5.75 | 37.64772 | 7.75222 | 3.16977 | 1.83906 |
| 5.8 | 36.37565 | 7.21432 | 2.93978 | 1.73056 |
| 5.85 | 34.99033 | 6.67094 | 2.71254 | 1.62462 |
| 5.9 | 33.50252 | 6.12684 | 2.49012 | 1.52224 |
| 5.95 | 31.92379 | 5.58682 | 2.27456 | 1.42439 |
| 6 | 30.26641 | 5.05563 | 2.06786 | 1.33198 |
| 6.05 | 28.54325 | 4.53795 | 1.87189 | 1.24587 |
| 6.1 | 26.76771 | 4.03834 | 1.68847 | 1.16688 |
| 6.15 | 24.95357 | 3.56119 | 1.51927 | 1.09574 |
| 6.2 | 23.11494 | 3.1107 | 1.36585 | 1.03313 |
| 6.25 | 21.26609 | 2.69084 | 1.22962 | 0.97962 |
| 6.3 | 19.42139 | 2.3053 | 1.11183 | 0.93572 |
| 6.35 | 17.59517 | 1.95747 | 1.01355 | 0.90184 |
| 6.4 | 15.80161 | 1.65043 | 0.9357 | 0.8783 |
| 6.45 | 14.05465 | 1.38685 | 0.87898 | 0.86532 |
| 6.5 | 12.36786 | 1.16908 | 0.84392 | 0.86303 |
| 6.55 | 10.75432 | 0.99902 | 0.83083 | 0.87143 |
| 6.6 | 9.22659 | 0.87817 | 0.83985 | 0.89046 |
| 6.65 | 7.79651 | 0.8076 | 0.87088 | 0.91993 |
| 6.7 | 6.47519 | 0.78792 | 0.92364 | 0.95958 |
| 6.75 | 5.27291 | 0.81931 | 0.99764 | 1.00902 |
| 6.8 | 4.19898 | 0.90149 | 1.09222 | 1.06779 |
| 6.85 | 3.26175 | 1.03373 | 1.20649 | 1.13535 |
| 6.9 | 2.4685 | 1.21489 | 1.33941 | 1.21106 |
| 6.95 | 1.82539 | 1.44335 | 1.48976 | 1.2942 |
| 7 | 1.3374 | 1.71711 | 1.65616 | 1.38401 |
| 7.05 | 1.00834 | 2.03377 | 1.83709 | 1.47964 |
| 7.1 | 0.84074 | 2.39053 | 2.03087 | 1.58019 |
| 7.15 | 0.83592 | 2.78426 | 2.23574 | 1.68472 |
| 7.2 | 0.99392 | 3.21148 | 2.44981 | 1.79225 |
| 7.25 | 1.31349 | 3.66845 | 2.67112 | 1.90176 |
| 7.3 | 1.79217 | 4.15113 | 2.89763 | 2.01225 |
| 7.35 | 2.42624 | 4.65528 | 3.12727 | 2.12266 |
| 7.4 | 3.21077 | 5.17647 | 3.35792 | 2.23196 |
| 7.45 | 4.13968 | 5.7101 | 3.58747 | 2.33913 |
| 7.5 | 5.20574 | 6.25147 | 3.81381 | 2.44316 |
| 7.55 | 6.40069 | 6.79583 | 4.03487 | 2.54308 |
| 7.6 | 7.71524 | 7.33838 | 4.24861 | 2.63795 |
| 7.65 | 9.13919 | 7.87434 | 4.45307 | 2.72688 |
| 7.7 | 10.66147 | 8.399 | 4.64637 | 2.80903 |
| 7.75 | 12.27028 | 8.90774 | 4.82673 | 2.88364 |
| 7.8 | 13.9531 | 9.39607 | 4.99251 | 2.95 |
| 7.85 | 15.69688 | 9.8597 | 5.14218 | 3.00749 |
| 7.9 | 17.48806 | 10.29454 | 5.27436 | 3.05558 |
| 7.95 | 19.31274 | 10.69677 | 5.38783 | 3.09381 |
| 8 | 21.15675 | 11.06285 | 5.48157 | 3.12182 |
| 8.05 | 23.00575 | 11.38954 | 5.5547 | 3.13935 |
| 8.1 | 24.84539 | 11.67398 | 5.60656 | 3.14623 |
| 8.15 | 26.66137 | 11.91366 | 5.63666 | 3.14241 |
| 8.2 | 28.43958 | 12.10647 | 5.64473 | 3.12792 |
| 8.25 | 30.16621 | 12.25071 | 5.6307 | 3.10289 |
| 8.3 | 31.82785 | 12.34511 | 5.5947 | 3.06755 |
| 8.35 | 33.41157 | 12.38884 | 5.53705 | 3.02225 |
| 8.4 | 34.90509 | 12.38151 | 5.45829 | 2.9674 |
| 8.45 | 36.29678 | 12.32319 | 5.35914 | 2.90352 |
| 8.5 | 37.57583 | 12.2144 | 5.24051 | 2.8312 |
| 8.55 | 38.73231 | 12.05608 | 5.1035 | 2.75113 |
| 8.6 | 39.75722 | 11.84964 | 4.94934 | 2.66407 |
| 8.65 | 40.6426 | 11.59689 | 4.77948 | 2.57081 |
| 8.7 | 41.38157 | 11.30006 | 4.59545 | 2.47225 |
| 8.75 | 41.96838 | 10.96176 | 4.39897 | 2.36929 |
| 8.8 | 42.39848 | 10.58497 | 4.19182 | 2.26292 |
| 8.85 | 42.66852 | 10.17302 | 3.97591 | 2.15412 |
| 8.9 | 42.77641 | 9.72951 | 3.75323 | 2.04393 |
| 8.95 | 42.72129 | 9.25837 | 3.52581 | 1.93336 |
| 9 | 42.50361 | 8.76374 | 3.29576 | 1.82346 |
| 9.05 | 42.12505 | 8.24998 | 3.06517 | 1.71526 |
| 9.1 | 41.58856 | 7.7216 | 2.83617 | 1.60977 |
| 9.15 | 40.8983 | 7.18326 | 2.61086 | 1.50798 |
| 9.2 | 40.05964 | 6.6397 | 2.3913 | 1.41085 |
| 9.25 | 39.07911 | 6.09571 | 2.17952 | 1.31928 |
| 9.3 | 37.96431 | 5.55607 | 1.97746 | 1.23415 |
| 9.35 | 36.72392 | 5.02553 | 1.78697 | 1.15623 |
| 9.4 | 35.36757 | 4.50877 | 1.6098 | 1.08627 |
| 9.45 | 33.90581 | 4.01033 | 1.44759 | 1.02492 |
| 9.5 | 32.34999 | 3.53459 | 1.3018 | 0.97275 |
| 9.55 | 30.7122 | 3.08576 | 1.1738 | 0.93026 |
| 9.6 | 29.00518 | 2.66777 | 1.06474 | 0.89784 |
| 9.65 | 27.24217 | 2.2843 | 0.97563 | 0.8758 |
| 9.7 | 25.43689 | 1.93873 | 0.9073 | 0.86434 |
| 9.75 | 23.60336 | 1.63411 | 0.86036 | 0.86357 |
| 9.8 | 21.75582 | 1.37311 | 0.83524 | 0.8735 |
| 9.85 | 19.90863 | 1.15802 | 0.83219 | 0.89403 |
| 9.9 | 18.07612 | 0.99075 | 0.85122 | 0.92498 |
| 9.95 | 16.27255 | 0.87276 | 0.89216 | 0.96604 |
| 10 | 14.51191 | 0.80509 | 0.95463 | 1.01684 |
| 10.05 | 12.80789 | 0.78834 | 1.03807 | 1.07691 |
| 10.1 | 11.17371 | 0.82265 | 1.1417 | 1.14567 |
| 10.15 | 9.62207 | 0.90773 | 1.26458 | 1.22249 |
| 10.2 | 8.16502 | 1.04281 | 1.40558 | 1.30663 |
| 10.25 | 6.81388 | 1.22673 | 1.56339 | 1.39733 |
| 10.3 | 5.57913 | 1.45785 | 1.73659 | 1.49371 |
| 10.35 | 4.47036 | 1.73414 | 1.92356 | 1.59489 |
| 10.4 | 3.49618 | 2.05318 | 2.1226 | 1.6999 |
| 10.45 | 2.66416 | 2.41214 | 2.33189 | 1.80778 |
| 10.5 | 1.98076 | 2.80789 | 2.54949 | 1.9175 |
| 10.55 | 1.45128 | 3.23692 | 2.77342 | 2.02803 |
| 10.6 | 1.07983 | 3.69547 | 3.00161 | 2.13835 |
| 10.65 | 0.86931 | 4.1795 | 3.23197 | 2.24741 |
| 10.7 | 0.82133 | 4.68476 | 3.46238 | 2.35419 |
| 10.75 | 0.93628 | 5.20678 | 3.69074 | 2.4577 |
| 10.8 | 1.21326 | 5.74098 | 3.91494 | 2.55695 |
| 10.85 | 1.65013 | 6.28266 | 4.13292 | 2.65102 |
| 10.9 | 2.24348 | 6.82704 | 4.34268 | 2.73903 |
| 10.95 | 2.98872 | 7.36934 | 4.5423 | 2.82015 |
| 11 | 3.88005 | 7.90479 | 4.72995 | 2.89363 |
| 11.05 | 4.91056 | 8.42865 | 4.90389 | 2.95876 |
| 11.1 | 6.07225 | 8.93634 | 5.06253 | 3.01494 |
| 11.15 | 7.35609 | 9.42337 | 5.20442 | 3.06165 |
| 11.2 | 8.75211 | 9.88545 | 5.32825 | 3.09843 |
| 11.25 | 10.24948 | 10.31853 | 5.43289 | 3.12496 |
| 11.3 | 11.83656 | 10.71878 | 5.51736 | 3.14098 |
| 11.35 | 13.50104 | 11.08268 | 5.58091 | 3.14634 |
| 11.4 | 15.22997 | 11.40702 | 5.62294 | 3.141 |
| 11.45 | 17.00995 | 11.68896 | 5.64306 | 3.12499 |
| 11.5 | 18.82713 | 11.92601 | 5.6411 | 3.09847 |
| 11.55 | 20.66741 | 12.11607 | 5.61707 | 3.06169 |
| 11.6 | 22.51649 | 12.25748 | 5.57119 | 3.01499 |
| 11.65 | 24.36 | 12.34899 | 5.50388 | 2.95882 |
| 11.7 | 26.18363 | 12.3898 | 5.41577 | 2.8937 |
| 11.75 | 27.9732 | 12.37954 | 5.30765 | 2.82023 |
| 11.8 | 29.71481 | 12.31831 | 5.18053 | 2.73912 |
| 11.85 | 31.39494 | 12.20664 | 5.03556 | 2.65112 |
| 11.9 | 33.00052 | 12.04553 | 4.8741 | 2.55705 |
| 11.95 | 34.51908 | 11.83638 | 4.6976 | 2.4578 |
| 12 | 35.93881 | 11.58103 | 4.5077 | 2.3543 |
| 12.05 | 37.2487 | 11.28175 | 4.30615 | 2.24752 |
| 12.1 | 38.43854 | 10.94116 | 4.09478 | 2.13846 |
| 12.15 | 39.49911 | 10.56226 | 3.87555 | 2.02815 |
| 12.2 | 40.42215 | 10.1484 | 3.65046 | 1.91761 |
| 12.25 | 41.2005 | 9.7032 | 3.42159 | 1.80789 |
| 12.3 | 41.82809 | 9.2306 | 3.19103 | 1.70001 |
| 12.35 | 42.30006 | 8.73475 | 2.9609 | 1.59499 |
| 12.4 | 42.61274 | 8.22002 | 2.73331 | 1.49381 |
| 12.45 | 42.76369 | 7.69095 | 2.51036 | 1.39742 |
| 12.5 | 42.75175 | 7.15218 | 2.29409 | 1.30672 |
| 12.55 | 42.577 | 6.60847 | 2.08648 | 1.22257 |
| 12.6 | 42.2408 | 6.0646 | 1.88945 | 1.14574 |
| 12.65 | 41.74577 | 5.52535 | 1.7048 | 1.07697 |
| 12.7 | 41.09575 | 4.99548 | 1.53422 | 1.0169 |
| 12.75 | 40.2958 | 4.47964 | 1.37929 | 0.96609 |
| 12.8 | 39.35214 | 3.98239 | 1.24142 | 0.92501 |
| 12.85 | 38.27209 | 3.50809 | 1.12188 | 0.89406 |
| 12.9 | 37.06405 | 3.06091 | 1.02177 | 0.87351 |
| 12.95 | 35.73742 | 2.64481 | 0.942 | 0.86358 |
| 13 | 34.30249 | 2.26343 | 0.88331 | 0.86433 |
| 13.05 | 32.77043 | 1.92013 | 0.84623 | 0.87578 |
| 13.1 | 31.15313 | 1.61794 | 0.83111 | 0.89782 |
| 13.15 | 29.46317 | 1.35951 | 0.83809 | 0.93022 |
| 13.2 | 27.71367 | 1.14712 | 0.8671 | 0.97271 |
| 13.25 | 25.91823 | 0.98264 | 0.91788 | 1.02486 |
| 13.3 | 24.0908 | 0.86751 | 0.98995 | 1.0862 |
| 13.35 | 22.24558 | 0.80275 | 1.08266 | 1.15616 |
| 13.4 | 20.39689 | 0.78893 | 1.19516 | 1.23406 |
| 13.45 | 18.55911 | 0.82616 | 1.32641 | 1.3192 |
| 13.5 | 16.7465 | 0.91413 | 1.47521 | 1.41075 |
| 13.55 | 14.97315 | 1.05206 | 1.6402 | 1.50788 |
| 13.6 | 13.25282 | 1.23872 | 1.81985 | 1.60966 |
| 13.65 | 11.59889 | 1.47249 | 2.01252 | 1.71515 |
| 13.7 | 10.0242 | 1.75131 | 2.21645 | 1.82335 |
| 13.75 | 8.54097 | 2.07271 | 2.42975 | 1.93325 |
| 13.8 | 7.16072 | 2.43388 | 2.65047 | 2.04381 |
| 13.85 | 5.89418 | 2.83163 | 2.87659 | 2.15401 |
| 13.9 | 4.75118 | 3.26246 | 3.10603 | 2.26281 |
| 13.95 | 3.74058 | 3.72258 | 3.33668 | 2.36919 |
| 14 | 2.87025 | 4.20795 | 3.56642 | 2.47214 |
| 14.05 | 2.14693 | 4.71428 | 3.79315 | 2.57071 |
| 14.1 | 1.57624 | 5.23713 | 4.01477 | 2.66397 |
| 14.15 | 1.16262 | 5.77189 | 4.22927 | 2.75105 |
| 14.2 | 0.90927 | 6.31385 | 4.43466 | 2.83112 |
| 14.25 | 0.81817 | 6.85825 | 4.62907 | 2.90345 |
| 14.3 | 0.89001 | 7.40028 | 4.8107 | 2.96734 |
| 14.35 | 1.12425 | 7.93519 | 4.97789 | 3.0222 |
| 14.4 | 1.51906 | 8.45825 | 5.1291 | 3.06751 |
| 14.45 | 2.07138 | 8.96487 | 5.26294 | 3.10286 |
| 14.5 | 2.77691 | 9.45058 | 5.37819 | 3.1279 |
| 14.55 | 3.63019 | 9.91111 | 5.47378 | 3.1424 |
| 14.6 | 4.62458 | 10.3424 | 5.54884 | 3.14624 |
| 14.65 | 5.75237 | 10.74066 | 5.60268 | 3.13936 |
| 14.7 | 7.0048 | 11.10238 | 5.6348 | 3.12184 |
| 14.75 | 8.37214 | 11.42437 | 5.64491 | 3.09384 |
| 14.8 | 9.84378 | 11.70379 | 5.63292 | 3.05562 |
| 14.85 | 11.40828 | 11.93819 | 5.59893 | 3.00754 |
| 14.9 | 13.0535 | 12.12551 | 5.54326 | 2.95006 |
| 14.95 | 14.76665 | 12.26409 | 5.46642 | 2.88371 |
| 15 | 16.53444 | 12.35271 | 5.36911 | 2.80911 |
| 15.05 | 18.34314 | 12.39059 | 5.25223 | 2.72696 |
| 15.1 | 20.17869 | 12.37741 | 5.11686 | 2.63804 |
| 15.15 | 22.02683 | 12.31326 | 4.96423 | 2.54318 |
| 15.2 | 23.87322 | 12.19873 | 4.79575 | 2.44327 |
| 15.25 | 25.7035 | 12.03481 | 4.61296 | 2.33924 |
| 15.3 | 27.50345 | 11.82296 | 4.41755 | 2.23207 |
| 15.35 | 29.2591 | 11.56503 | 4.2113 | 2.12277 |
| 15.4 | 30.9568 | 11.26331 | 3.99612 | 2.01236 |
| 15.45 | 32.58336 | 10.92044 | 3.77398 | 1.90188 |
| 15.5 | 34.12613 | 10.53944 | 3.54691 | 1.79236 |
| 15.55 | 35.57314 | 10.12367 | 3.31701 | 1.68483 |
| 15.6 | 36.91314 | 9.6768 | 3.08638 | 1.58029 |
| 15.65 | 38.13571 | 9.20275 | 2.85715 | 1.47974 |
| 15.7 | 39.23136 | 8.7057 | 2.63141 | 1.38411 |
| 15.75 | 40.19156 | 8.19002 | 2.41124 | 1.29429 |
| 15.8 | 41.00885 | 7.66027 | 2.19866 | 1.21114 |
| 15.85 | 41.67689 | 7.12109 | 1.99563 | 1.13542 |
| 15.9 | 42.19047 | 6.57724 | 1.804 | 1.06786 |
| 15.95 | 42.54561 | 6.0335 | 1.62553 | 1.00907 |
| 16 | 42.73955 | 5.49466 | 1.46187 | 0.95962 |
| 16.05 | 42.77078 | 4.96547 | 1.31451 | 0.91997 |
| 16.1 | 42.63906 | 4.45058 | 1.18482 | 0.89048 |
| 16.15 | 42.34541 | 3.95452 | 1.07397 | 0.87144 |
| 16.2 | 41.89211 | 3.48167 | 0.98299 | 0.86303 |
| 16.25 | 41.28269 | 3.03617 | 0.91271 | 0.86532 |
| 16.3 | 40.52188 | 2.62196 | 0.86377 | 0.87828 |
| 16.35 | 39.6156 | 2.24268 | 0.83664 | 0.90181 |
| 16.4 | 38.57089 | 1.90166 | 0.83154 | 0.93568 |
| 16.45 | 37.39587 | 1.60191 | 0.84854 | 0.97957 |
| 16.5 | 36.09967 | 1.34606 | 0.88748 | 1.03307 |
| 16.55 | 34.69236 | 1.13638 | 0.94799 | 1.09567 |
| 16.6 | 33.18489 | 0.97469 | 1.02952 | 1.1668 |
| 16.65 | 31.58897 | 0.86243 | 1.13133 | 1.24579 |
| 16.7 | 29.91699 | 0.80058 | 1.25248 | 1.33189 |
| 16.75 | 28.18195 | 0.78968 | 1.39186 | 1.42429 |
| 16.8 | 26.39733 | 0.82984 | 1.54818 | 1.52214 |
| 16.85 | 24.57699 | 0.9207 | 1.72002 | 1.62451 |
| 16.9 | 22.73509 | 1.06146 | 1.9058 | 1.73045 |
| 16.95 | 20.88592 | 1.25088 | 2.1038 | 1.83895 |
| 17 | 19.04385 | 1.48729 | 2.31222 | 1.94901 |
| 17.05 | 17.2232 | 1.76862 | 2.52914 | 2.05959 |
| 17.1 | 15.4381 | 2.09238 | 2.75257 | 2.16965 |
| 17.15 | 13.70242 | 2.45573 | 2.98045 | 2.27816 |
| 17.2 | 12.02963 | 2.85548 | 3.2107 | 2.38411 |
| 17.25 | 10.43274 | 3.2881 | 3.4412 | 2.4865 |
| 17.3 | 8.92414 | 3.74978 | 3.66983 | 2.58437 |
| 17.35 | 7.51555 | 4.23646 | 3.8945 | 2.6768 |
| 17.4 | 6.2179 | 4.74387 | 4.11314 | 2.76292 |
| 17.45 | 5.04128 | 5.26752 | 4.32375 | 2.84194 |
| 17.5 | 3.99482 | 5.80282 | 4.52438 | 2.91309 |
| 17.55 | 3.08664 | 6.34506 | 4.7132 | 2.97573 |
| 17.6 | 2.3238 | 6.88944 | 4.88847 | 3.02926 |
| 17.65 | 1.71222 | 7.4312 | 5.04859 | 3.07317 |
| 17.7 | 1.25665 | 7.96555 | 5.19208 | 3.10707 |
| 17.75 | 0.96062 | 8.4878 | 5.31763 | 3.13062 |
| 17.8 | 0.82644 | 8.99333 | 5.42407 | 3.14361 |
| 17.85 | 0.85514 | 9.47772 | 5.51044 | 3.14591 |
| 17.9 | 1.0465 | 9.93668 | 5.57594 | 3.13751 |
| 17.95 | 1.39904 | 10.36617 | 5.61998 | 3.11848 |
| 18 | 1.91002 | 10.76243 | 5.64213 | 3.089 |
| 18.05 | 2.57547 | 11.12195 | 5.64221 | 3.04935 |
| 18.1 | 3.39023 | 11.44157 | 5.6202 | 2.9999 |
| 18.15 | 4.34796 | 11.71847 | 5.57632 | 2.94112 |
| 18.2 | 5.44124 | 11.95023 | 5.51097 | 2.87355 |
| 18.25 | 6.66158 | 12.13479 | 5.42474 | 2.79783 |
| 18.3 | 7.99949 | 12.27053 | 5.31842 | 2.71467 |
| 18.35 | 9.44459 | 12.35626 | 5.19301 | 2.62485 |
| 18.4 | 10.98566 | 12.39122 | 5.04964 | 2.52921 |
| 18.45 | 12.61072 | 12.3751 | 4.88963 | 2.42865 |
| 18.5 | 14.30716 | 12.30805 | 4.71445 | 2.32411 |
| 18.55 | 16.06181 | 12.19065 | 4.52572 | 2.21658 |
| 18.6 | 17.86103 | 12.02394 | 4.32516 | 2.10706 |
| 18.65 | 19.69085 | 11.80939 | 4.11462 | 1.99657 |
| 18.7 | 21.53705 | 11.54889 | 3.89603 | 1.88616 |
| 18.75 | 23.3853 | 11.24473 | 3.67139 | 1.77687 |
| 18.8 | 25.22124 | 10.89958 | 3.44278 | 1.6697 |
| 18.85 | 27.0306 | 10.5165 | 3.21229 | 1.56568 |
| 18.9 | 28.79932 | 10.09885 | 2.98203 | 1.46577 |
| 18.95 | 30.51367 | 9.65031 | 2.75412 | 1.37091 |
| 19 | 32.16032 | 9.17482 | 2.53066 | 1.282 |
| 19.05 | 33.72647 | 8.67658 | 2.31369 | 1.19986 |
| 19.1 | 35.19997 | 8.15998 | 2.1052 | 1.12527 |
| 19.15 | 36.56935 | 7.62955 | 1.90712 | 1.05891 |
| 19.2 | 37.82398 | 7.08998 | 1.72125 | 1.00143 |
| 19.25 | 38.9541 | 6.546 | 1.54931 | 0.95335 |
| 19.3 | 39.95093 | 6.00242 | 1.39288 | 0.91513 |
| 19.35 | 40.80673 | 5.46401 | 1.25338 | 0.88712 |
| 19.4 | 41.51484 | 4.93551 | 1.1321 | 0.86959 |
| 19.45 | 42.06976 | 4.42158 | 1.03015 | 0.8627 |
| 19.5 | 42.46718 | 3.92674 | 0.94848 | 0.86651 |
| 19.55 | 42.704 | 3.45534 | 0.88782 | 0.881 |
| 19.6 | 42.77839 | 3.01153 | 0.84873 | 0.90602 |
| 19.65 | 42.68977 | 2.59923 | 0.83158 | 0.94134 |
| 19.7 | 42.43882 | 2.22205 | 0.83653 | 0.98663 |
| 19.75 | 42.0275 | 1.88333 | 0.86351 | 1.04146 |
| 19.8 | 41.45901 | 1.58603 | 0.9123 | 1.10532 |
| 19.85 | 40.73775 | 1.33277 | 0.98243 | 1.17761 |
| 19.9 | 39.86935 | 1.12579 | 1.07327 | 1.25766 |
| 19.95 | 38.86055 | 0.9669 | 1.18399 | 1.34471 |
| 20 | 37.71918 | 0.85751 | 1.31356 | 1.43795 |
